# Mapping of Candidate Genes Involved in Bud Dormancy and Flowering Time in Sweet Cherry (*Prunus avium*)

**DOI:** 10.1371/journal.pone.0143250

**Published:** 2015-11-20

**Authors:** Sophie Castède, José Antonio Campoy, Loïck Le Dantec, José Quero-García, Teresa Barreneche, Bénédicte Wenden, Elisabeth Dirlewanger

**Affiliations:** 1 INRA, UMR 1332 de Biologie du Fruit et Pathologie, F-33140, Villenave d’Ornon, France; 2 Univ. Bordeaux, UMR 1332 de Biologie du Fruit et Pathologie, F-33140, Villenave d’Ornon, France; NARO Institute of Fruit Tree Science, JAPAN

## Abstract

The timing of flowering in perennial plants is crucial for their survival in temperate climates and is regulated by the duration of bud dormancy. Bud dormancy release and bud break depend on the perception of cumulative chilling during endodormancy and heat during the bud development. The objectives of this work were to identify candidate genes involved in dormancy and flowering processes in sweet cherry, their mapping in two mapping progenies ‘Regina’ × ‘Garnet’ and ‘Regina’ × ‘Lapins’, and to select those candidate genes which co-localized with quantitative trait loci (QTLs) associated with temperature requirements for bud dormancy release and flowering. Based on available data on flowering processes in various species, a list of 79 candidate genes was established. The peach and sweet cherry orthologs were identified and primers were designed to amplify sweet cherry candidate gene fragments. Based on the amplified sequences of the three parents of the mapping progenies, SNPs segregations in the progenies were identified. Thirty five candidate genes were genetically mapped in at least one of the two progenies and all were *in silico* mapped. Co-localization between candidate genes and QTLs associated with temperature requirements and flowering date were identified for the first time in sweet cherry. The allelic composition of the candidate genes located in the major QTL for heat requirements and flowering date located on linkage group 4 have a significant effect on these two traits indicating their potential use for breeding programs in sweet cherry to select new varieties adapted to putative future climatic conditions.

## Introduction

The life cycle control of plants is complex, especially for perennial species with repeating cycles under contrasting environments. This is particularly true for flowering as it is one of the most fundamental and complex developmental processes in plants and needs to be synchronized with climatic conditions especially in temperate regions with alternating well-differentiated seasons. As flowering is crucial for survival, buds of perennial species in temperate regions cease growth and become dormant during part of autumn and winter to protect important and delicate tissues from low winter temperatures [1]. This transitional dormancy period is described by Lang [2] to have three characteristic phases, paradormancy, endodormancy and ecodormancy. In paradormancy, internal signals inhibit bud growth during the growing season. In autumn and winter, endodormancy prevents bud growth and requires a certain amount of chilling to make transition to ecodormancy (chilling requirements), during which buds can resume growth when environmental conditions are favorable. Following the endodormancy stage, buds are ecodormant and require a certain amount of heat to allow the flowering process to start (heat requirements). Consequently, dormancy and flowering are linked in perennial species and their proper timing determines the efficiency of the process and the productivity for fruit species.

Recent advances in the understanding of the molecular mechanisms controlling dormancy and flowering in perennials reported that bud dormancy is dynamic rather than a single state, with interactions between intrinsic and environmental signaling cues altering the depth of dormancy [1]. Thus, changes in the environmental signaling may affect the dormancy cycle and consequently the overall plant phenology. Several studies have reported the effect of global warming on winter chilling accumulation of temperate perennial crops during the last century [3, 4] and predicted an upcoming substantial decrease in the production in different perennial crop producing areas [4, 5]. Insufficient chilling temperatures can cause problems related to flower bud abscission and quality, fruit set and quality and vegetative growth and development [6], whereas in cold areas, the increase in temperatures may result in a substantially increased spring frost risk [7, 8].

The flowering process in perennial plants is different from that of annual plants, bud-dormancy release and reset being specific to the perennials. In trees, and fruit trees in particular, flower buds are differentiated in the year preceding the flowering date, but the precise timing for flowering is determined by the temperature response during the dormancy period. On the other hand, in some perennials and most annuals, floral induction and flowering occur during a unique growth cycle and are controlled by similar environmental and endogenous factors. Both in endodormancy and vernalization systems, flowering and flower induction respectively are promoted after plants have been exposed to a certain amount of cold temperature, suggesting similar response mechanisms. In the model plant *Arabidopsis thaliana*, as well as in many other annual plants like wheat and rice, analyses of genes involved in flowering processes led to the identification of four major pathways involved in environmental and endogenous signals transduction: vernalization, photoperiod, gibberellic acid and the autonomous pathways; and a large number of genes were identified to regulate flowering time [9–11]. In *Arabidopsis thaliana*, the MADS-box transcription factor *FLOWERING LOCUS C* (*FLC*) prevents the flowering transition by repressing the floral integrator genes *FLOWERING LOCUS T* (*FT*) and *SUPPRESSOR OF OVEREXPRESSION OF CONSTANS 1* (*SOC1*) [12]. The expression of *FLC* is enhanced by a plant-specific protein encoded by *FRIGIDA* (*FRI*) [13]. Vernalization overcomes the effect of *FRI* by repressing *FLC* expression through complex processes, including an epigenetic mechanism that involves the synthesis of noncoding RNAs and the binding of the Polycomb group (PcG) proteins [14–16]. Among the PcG genes, those coding for the Polycomb repressive complexes 2 (PRC2) subunits, like *CURLY LEAF* (*CLF*), *EMBRYONIC FLOWER2 (EMF2*) and *FERTILIZATION-INDEPENDENT ENDOSPERM (FIE)*, play a major role in the repression of *FLC* [17]. However, vernalization mechanisms may vary between annual plants involving different regulators. For example, in cereals, cold temperatures led to an increase in the expression of the *VERNALIZATION1* and *2* (*VRN1*, *VRN2*) genes [18, 19].

Regarding perennial plants, bud dormancy and flowering have been extensively studied in poplar (*Populus ssp*.), the model woody plant, and a strong involvement of photoperiod, temperature and gibberellic acid was reported [20, 21]. However, alternative mechanisms of flowering regulation were also described in other woody perennials. In particular, dormancy release for several *Rosaceae* trees was shown to rely mainly on temperature sensing mechanisms [22, 23] making them vulnerable to global warming. In *Prunus*, several studies suggest that chilling requirements affect flowering date more strongly than heat requirements: sweet cherry [24, 25], apricot (*Prunus armeniaca* L.) [26], almond (*Prunus amygdalus* L. syn. *Prunus dulcis* M.) [27], and peach (*Prunus persica* L.) [28]. Using highly dense linkage maps [29], quantitative trait loci (QTL) for flowering date, chilling and heat requirements were detected in sweet cherry [25]. In this species, QTLs with major effects for chilling requirements and flowering date were found on linkage group (LG) 4 [25]. They were highly stable between the years of evaluation (three for the chilling requirements and five for the flowering date) and with a narrow confidence interval (less than 1 cM). This result was in agreement with previous works in almond [27], apricot [30–32] and peach [31, 33]. Additionally, a QTL for flowering date co-localizing with the evergrowing (EVG) region was detected on LG1 in peach [33, 34]and sweet cherry [25]. In peach, six tandemly repeated MADS-box genes, named *DORMANCY ASSOCIATED MADS-box1-6* (*DAM1*-*6*) genes, were located in the EVG region and two of them (*DAM5* and *DAM6*) were reported to be the strongest candidate genes (CGs) for the major QTL for chilling requirements at the lower end of LG1 [35–37]. These genes are not expressed in the peach mutant *evergrowing*, which fails to both cease growth and enter dormancy under dormancy-inducing conditions [38]. More recently, histone modifications in the *DAM6* gene were investigated and results suggested the role of epigenetic mechanisms in *DAM6* regulation and bud dormancy release [39]. Modification of *DAM6* chromatin in a similar way to *FLC*, as a consequence of the prolonged exposure to low temperatures [40], suggests the existence of mechanistic similarities between vernalization and bud dormancy processes. Several additional genes were also reported to play an important role in dormancy processes in *Rosaceae* species. In Japanese apricot (*Prunus mume* Sieb. et Zucc.) and in raspberry (*Rubus idaeus* L.) the *SHORT VEGETATIVE PHASE (SVP)* genes are down-regulated during dormancy release [41, 42]. All these results suggest a complex genetic and molecular determinism of dormancy and flowering processes.

Following our work that led to the identification of QTLs for flowering, chilling and heat requirements in sweet cherry [25], we aimed to further investigate these traits and the genes and pathways potentially involved. The main objectives of this work were to identify and map genes involved in the flowering processes. Thus, genes known to be involved in flowering processes in model (*Arabidopsis* and poplar) and related species (peach and blackcurrant) were selected. These genes were then mapped in two sweet cherry mapping progenies and those that co-localized with flowering date QTLs already identified in these progenies were selected. This work provides further information on genetic and molecular determinism of flowering phenology and its findings will help cherry breeders to implement efficient strategies focused on the adaptation to future climatic conditions.

## Materials and Methods

### Plant material

Two intraspecific sweet cherry F1 progenies and their parents were used for genetic analyses.

One progeny, called R×L, of 124 individuals derived from a cross between a German variety, ‘Regina’ and an American variety ‘Lapins’. The other progeny, called R×G, of 117 individuals was derived from a cross between the variety ‘Regina’ and an American variety ‘Garnet’.

In this work, selected varieties, ‘Garnet’, ‘Lapins’ and ‘Regina’ differ for traits involved in flowering phenology. ‘Lapins’ and ‘Garnet’ are considered early flowering varieties (before reference cultivar ‘Burlat’) and ‘Regina’ displays a late flowering phenotype (after ‘Burlat’).

All trees were cultivated in the experimental orchard of INRA-Bordeaux research center located at Toulenne, near Bordeaux, France. The trees were grown on their own roots, on deep loamy soil on the bank of Garonne River at 15 m above sea level, lat. 44.57 N, long. 0.28 W, in a mild winter area with a yearly average rainfall of 825 mm.

R×L and R×G mapping progenies were previously used for a QTL detection study for the three traits, chilling requirements, heat requirements and flowering date [25].

### Sampling and DNA extraction

For genotyping, random samples of 1 g of young leaves were collected from ‘Regina’, ‘Garnet’, ‘Lapins’ and all individuals of both progenies. Samples were frozen with liquid nitrogen, grounded into a fine powder and stored at -80°C. DNA extraction was from 100 mg of frozen leaves powder, with the DNeasy Plant Mini Kit (Qiagen) following manufacturing instructions and stored at -20°C.

### Candidate genes selection

CGs involved in dormancy and flowering processes were selected from already published data on *Prunus* species [25, 35, 43], black currant (*Ribes nigrum* L.) [44], poplar (*Populus trichocarpa)* [20] and *Arabidopsis* [45–48], model species for the study of dormancy or flowering mechanisms (S1 Table). Genes controlling dormancy and vernalization and the different flowering pathways (temperature, photoperiod, gibberellin, autonomous) were analyzed. For each selected gene, the peach orthologous sequence was identified in the Genome Database for Rosaceae (GDR). As the analyses were performed before the release of the second version of the peach sequence, the peach CGs were referred according to the prediction gene code of the Peach genome v1.0 (named ppaxxx) [49]. Following the release of the Peach genome v2.0.a1, the corresponding sequences were identified in this new version (named Prupe.XXX). Sweet cherry orthologs were searched in the sweet cherry ‘Regina’ transcribed sequences [50]. Orthologs were identified using BLASTp on peach and sweet cherry with an E value threshold of 10−8. Bidirectional best hit was used to test each sweet cherry sequence identified against the genome from which the original CG was defined to ensure that it was the accurate ortholog.

### Candidate genes mapping

#### SNP identification

In order to map the CGs, heterozygous SNPs are required for at least one of the parents. Sequence alignments were visualized using the software BioEdit Sequence Alignment Editor [51]. Primers were designed using the software online Primer3Plus (http://www.bioinformatics.nl/cgi-bin/primer3plus/primer3plus.cgi/) (S2 Table). PCR amplifications were performed in a final volume of 36 μl including 0.2 μM of each primer, 200 μM of each dNTP, 1.5 mM MgCl2, 10 ng of DNA of each parent and 0.4 U Taq polymerase (Invitrogen). PCR products were controlled on agarose gel and sent for sequencing to GENOME Express (http://www.beckmangenomics.com/). Heterozygous SNPs in each parent (‘Garnet’, ‘Lapins’ and ‘Regina’) were identified by visual inspection of DNA sequence chromatogram (BioEdit Sequence Alignment Editor).

#### SNP genotyping

Sequences of a minimum of 50 bp up and downstream from the SNP were used for primer design using Sequenom MassARRAY Assay Design 3.1 software with default parameters (S3 Table). The genotyping for the two progenies and their parents was performed according to the iPLEX protocol from Sequenom (http://www.sequenom.com/Home) using the MassARRAY Analyzer 4 System (Sequenom, San Diego, USA) at the Genomic and Sequencing Facility of Bordeaux, France. Data were analyzed with Typer4 software (Sequenom, San Diego, USA).

In order to identify if the SNPs associated amino acid change, the DNA sequences of the CGs were translated into protein using MEGA6 [52].

#### CGs mapping in the two progenies

The CGs were then mapped on the available linkage maps constructed for the two progenies R×L and R×G [25, 29] using JoinMap® 4.0 [53]. A minimum LOD value of 3 and a recombinant threshold of 0.35 were chosen for mapping. Kosambi’s mapping function was used to convert recombination frequency into map distance [54]. The SNP markers were named according to the original full names used in the RosBREED cherry 6K SNP array, including the physical and genetic positions and the targeted genome [55]. To facilitate reading of data, SNP names were contracted as follows: ‘RosBREED_snp_sweet_cherry_Pp1_00493090’ is written ‘Rsweet_1_00493090’. Mapped CGs in sweet cherry progenies were named preceded by the prefix “*Pav”*. The graphical presentation of linkage maps was performed using the MapChart software version 2.2 [56].

CGs that could not be genetically mapped were mapped *in silico* using the peach genome v2.0.a1 sequence. This was performed using the peach physical position of the SNP markers located in the sweet cherry maps flanking each CG. *In silico* mapped CGs were preceded by the prefix “*is”*.

In order to select the CGs located in the QTLs controlling the flowering date identified in the two progenies (R×L and R×G) and in QTLs for chilling and heat requirements identified in R×G [25], the physical positions of the confidence interval of the QTLs was estimated from the physical position of the closest SNP markers from each side of the confidence interval according to the Peach genome v2.0.a1.

### Allelic effect of candidate genes on the phenological traits

Three SNPs were used for haplotype construction, including SNPs located in *NUA*, *EMF2* and *ARP4*. T-tests (P>0.05) were used to determinate significant differences between means of each haplotype. The statistical analyses were performed using the R Commander package of the software R 2.15.0 (R_Development_Core_Team, 2012).

The phenotypic traits were evaluated according to procedures described by Castède *et al*. [25]. The Dynamic model [57] was used to calculate chilling accumulation as the chill portion (CP) and the model proposed by Richardson *et al*. [58] was chosen to calculate the heat accumulation as growing degree hours (GDH). Flowering dates were evaluated when 70% of floral buds reached the full bloom stage in the field.

## Results

### Candidate genes identification

A list of 79 functional CGs was established based on available data on flowering processes (S1 Table). Among these 79 genes, 20 have been shown to be involved in the photoperiod pathway, 25 in the temperature pathway, 15 in dormancy processes, five in the gibberellin pathway, two in the autonomous pathway and three were floral integrators. Following this selection, both peach and sweet cherry orthologous sequences were identified for each gene (S1 Table). Peach CG orthologous sequences were identified using the two versions of the genome sequence (*Prunus Persica* v1.0 and v2.0.a1). Sweet cherry orthologous cDNA sequences were found for each CG (S1 File). Sweet cherry ‘Regina’ transcribed sequences of candidate genes are available in the GDR http://www.rosaceae.org (Accession number tfGDR1019).

### Candidate genes mapping

The primers used to amplify sweet cherry CGs are indicated in S2 Table. For CGs for which no sweet cherry accurate ortholog was found by bidirectional best hit, primers were designed from the peach sequence (S2 File). Sequence of the *DAM6* genomic amplicon from the three parents revealed a strong homology with a cDNA fragment of the ‘Regina’ unigene corresponding to part of the peach *DAM6* gene (S2 File). Amplified sequences were obtained for 76 genes, 67 of them with a single amplified fragment (S3 Table). For all candidate genes, the amplified products from the three parents ‘Regina’, ‘Lapins’ and ‘Garnet’ were sequenced and aligned with the peach orthologous sequence (S2 File). Multiple amplifications were obtained for nine genes, probably due to the presence of multigenic families (S3 Table). SNPs were detected for 39 genes. For two of them (*FRI* and *GH17-39*), two SNPs were identified. Twenty one SNPs were found in intronic regions (within 19 genes), 19 in exons and one in the 5’UTR region. Among the 19 SNPs found in exons, 6 induced nonsynonymous substitutions (S3 Table). Twenty nine SNPs were transitions (19 were C/T polymorphisms while 10 were A/G polymorphisms) and 12 were transversions (4 A/T, 3 G/T, 3 C/G, 2 A/C). *PavVRN2* was placed on the ‘Regina’ linkage map in the R×L progeny but could not be mapped in this parent in the R×G progeny due to an ambiguous genotyping. In addition, for four genes (*FIO1*, *VIL1*, *GH17-39*, *GH17-101*), ambiguous genotyping in both progenies did not allow their mapping.

**Fig 1:**
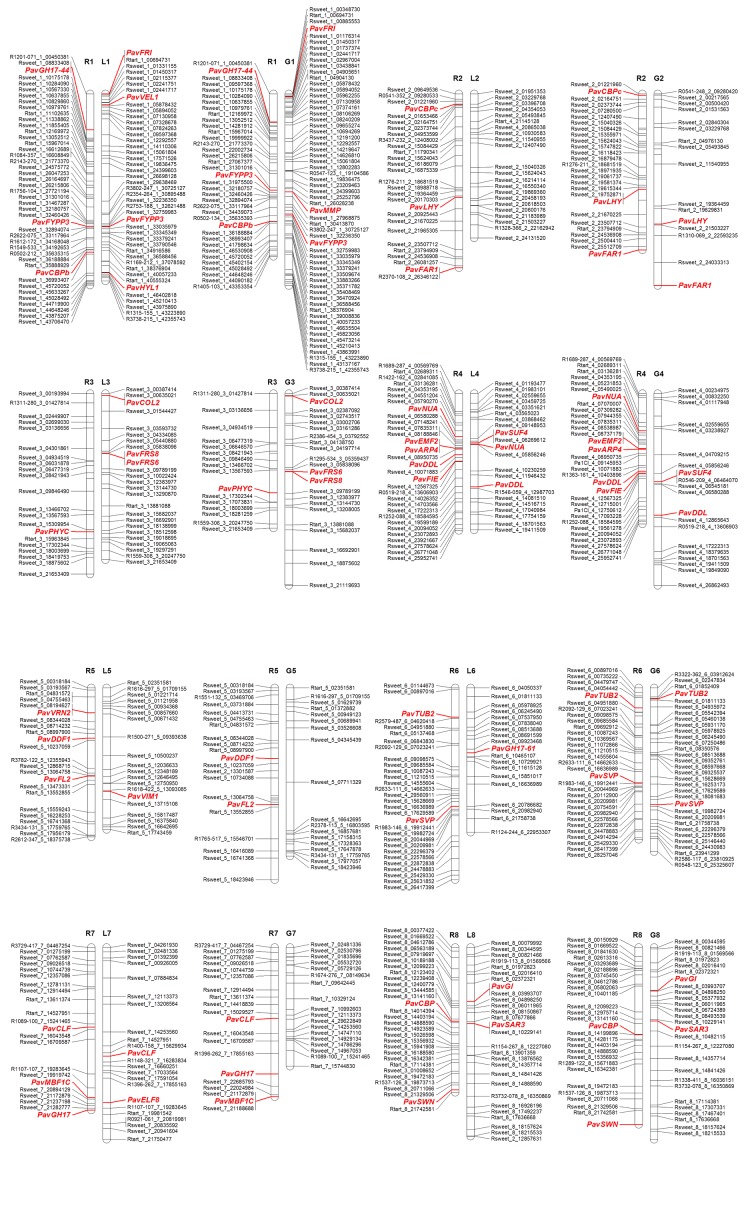
Candidate genes localization in linkage maps constructed from two progenies ‘Regina’ × ‘Garnet’ (R×G) and ‘Regina’ × ‘Lapins’ (R×L). The SNP maps were already reported for R×L [29] and for R×G [25]. The SNP markers are named according to the original full names used in the RosBREED cherry 6K SNP array, including the physical and genetic positions and the targeted genome [55] but with contraction: for example ‘RosBREED_snp_sweet_cherry_Pp1_00493090’ is written ‘Rsweet_1_00493090’. The candidate genes are indicated in red and were genetically mapped using SNP markers described in S3 Table and available in the GDR database.

The ‘Regina’ and ‘Lapins’ linkage maps were constructed based on the segregation analysis in the R×L progeny. Mapped candidate genes are presented in S1 Fig. The ‘Regina’ and ‘Garnet’ linkage maps from the analysis of the R×G progeny are shown in S2 Fig. In total, based on identified SNPs, 35 mapped CGs were distributed on all linkage groups (LG) (Fig 1, S3 Table). Twenty two CGs were mapped in ‘Regina’, 16 in ‘Lapins’ and 14 in ‘Garnet’. Two CGs were mapped in the three parents (*PavFYPP3* and *PavDDL*), seven in ‘Lapins’ and ‘Garnet’ (*PavFRI*, *PavFRS6*, *PavFRS8*, *PavCOL2*, *PavSUF4*, *PavGI*, *PavSAR3*) and two in ‘Regina’ and ‘Garnet’ (*PavFAR1*, *PavCLF*).

They were all mapped in sweet cherry at the same location than their peach orthologs, except one, orthologous of *SUPPRESSOR OF AUXIN RESISTANCE 3* (*PavSAR3*), mapped on LG8 in sweet cherry while it is located on LG2 in peach. Twenty eight genes could not be mapped because no polymorphic SNP was detected among the parents of the mapping progenies. However, for each CG, the physical position of the peach ortholog was determined on the *Prunus persica* v2.0.a1 genome using GDR generic Genome Browser (GBrowse) (S3 Table). Subsequently, all CGs that were not genetically mapped were localized *in silico* in sweet cherry based on the high synteny between sweet cherry and peach as shown in the high density sweet cherry map using Peach genome v1.0 markers [29].

The mapping data are available in S4 Table and in the GDR http://www.rosaceae.org (Accession number tfGDR1019).

### Positioning of CGs within QTL intervals

**Fig 2:**
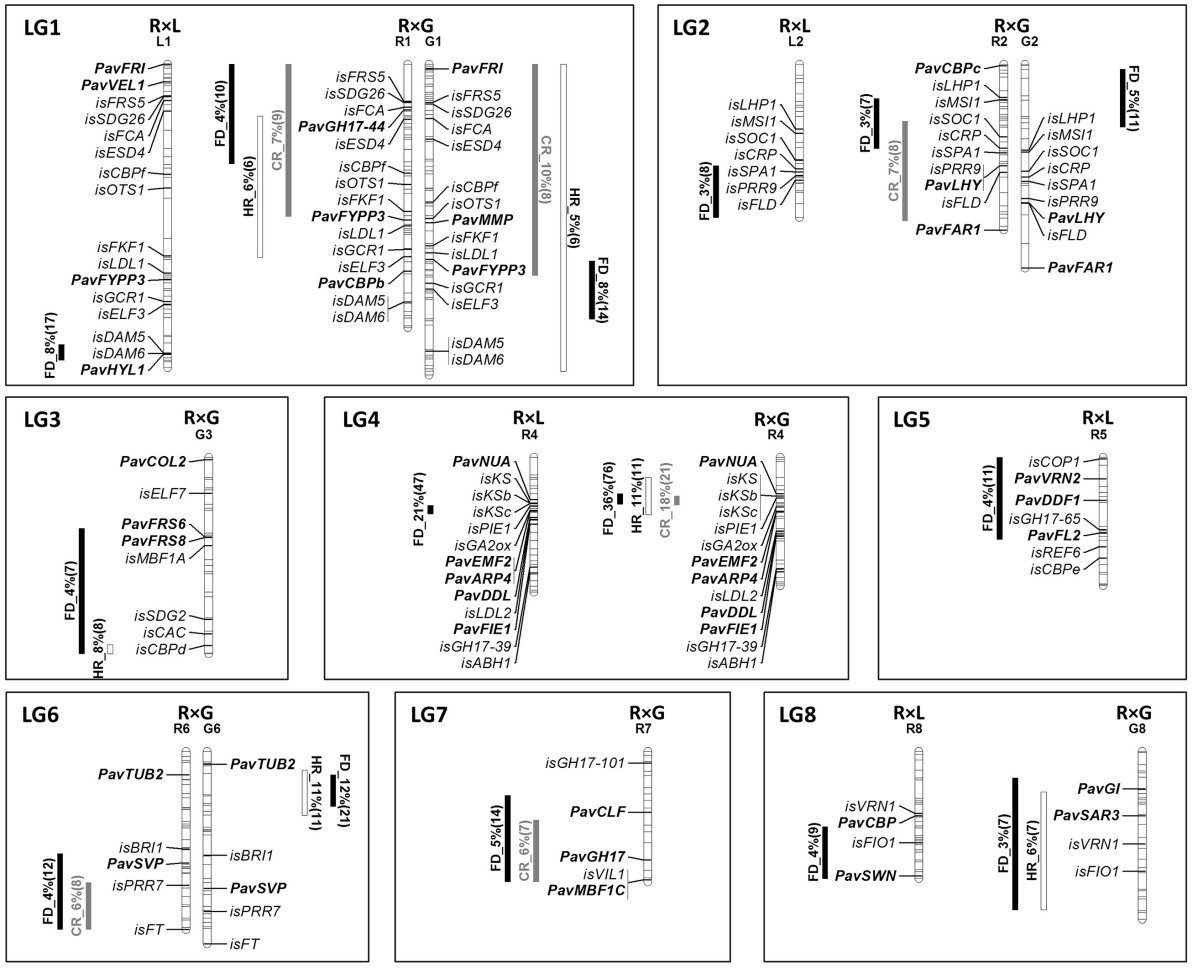
Co-localization between candidate genes and QTLs. QTLs detected in ‘Regina’ × ‘Lapins’ (R×L) (right) in ‘Regina’ × ‘Garnet’ (R×G) (left) progenies are indicated by grey bars for chilling requirements (CR), white bars for heat requirements (HR), and black bars for flowering date (FD). Candidate genes mapped on R×L are indicated on the left, and those mapped on R×G are indicated on the right. Those genetically mapped are in bold and are prefixed with ‘*Pav*’ for *Prunus avium*, and those mapped *in silico* using the Peach genome v2.0.a1 are preceded by the prefix “*is”*.

Among the 79 CGs analyzed in this study, 57 CGs co-localize with QTLs for chilling or heat requirements or for flowering date, 21 CGs being genetically mapped and 38 CGs being *in silico* mapped (Fig 2, S5 Table).

**Fig 3:**
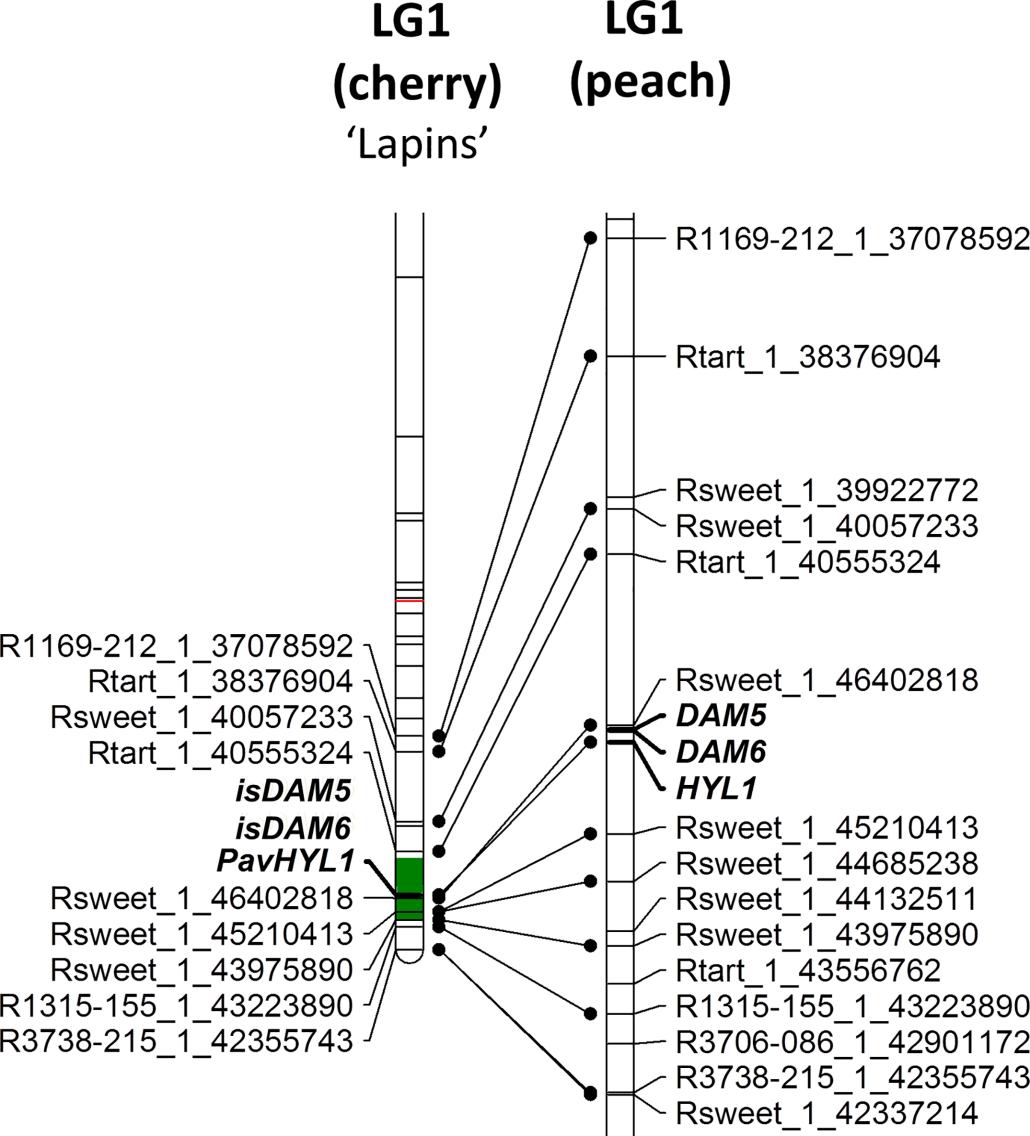
Co-localization between candidate genes and QTL for the flowering date on ‘Lapins’ linkage group 1 and comparison with the peach physical map of the homologous/syntenic region. The region covered by the QTL for the flowering date is indicated in green. The candidate genes are indicated in bold. For peach, the physical position of the candidate genes and of the SNP markers refers to the Peach genome sequence v2.0.a1.

On LG1, the QTL regions for heat and chilling requirements are very large, in particular for the ‘Garnet’ map, covering nearly all the linkage group especially for heat requirements. Consequently, nearly all CGs are located in QTLs (16 among the 18 CGs located in this LG1). The QTL for flowering date detected in ‘Lapins’ is much smaller, covering a region of 5.4 cM (S5 Table, Fig 3). *PavHYL1*, *isDAM5* and *isDAM6* are located in this QTL region, explaining 8% of the variation in the R×L progeny (Fig 3, S5 Table). This genomic region is conserved between sweet cherry and peach (Fig 3).

On LG2, *PavLHY*, *isSOC1*, *isCRP*, *isSPA1*, *isPRR9*, *isFLD* are located within the region of the QTLs for chilling requirements and flowering date, except *PavLHY* located only in the QTL for chilling requirements.

On LG3, *PavFRS6*, *PavFRS8*, *isMBF1*, *isSDG2*, *isCAC*, *isCALd* are located within the region of the QTL for flowering date, and *isCALd* is also localized within the QTL for heat requirements.

**Fig 4:**
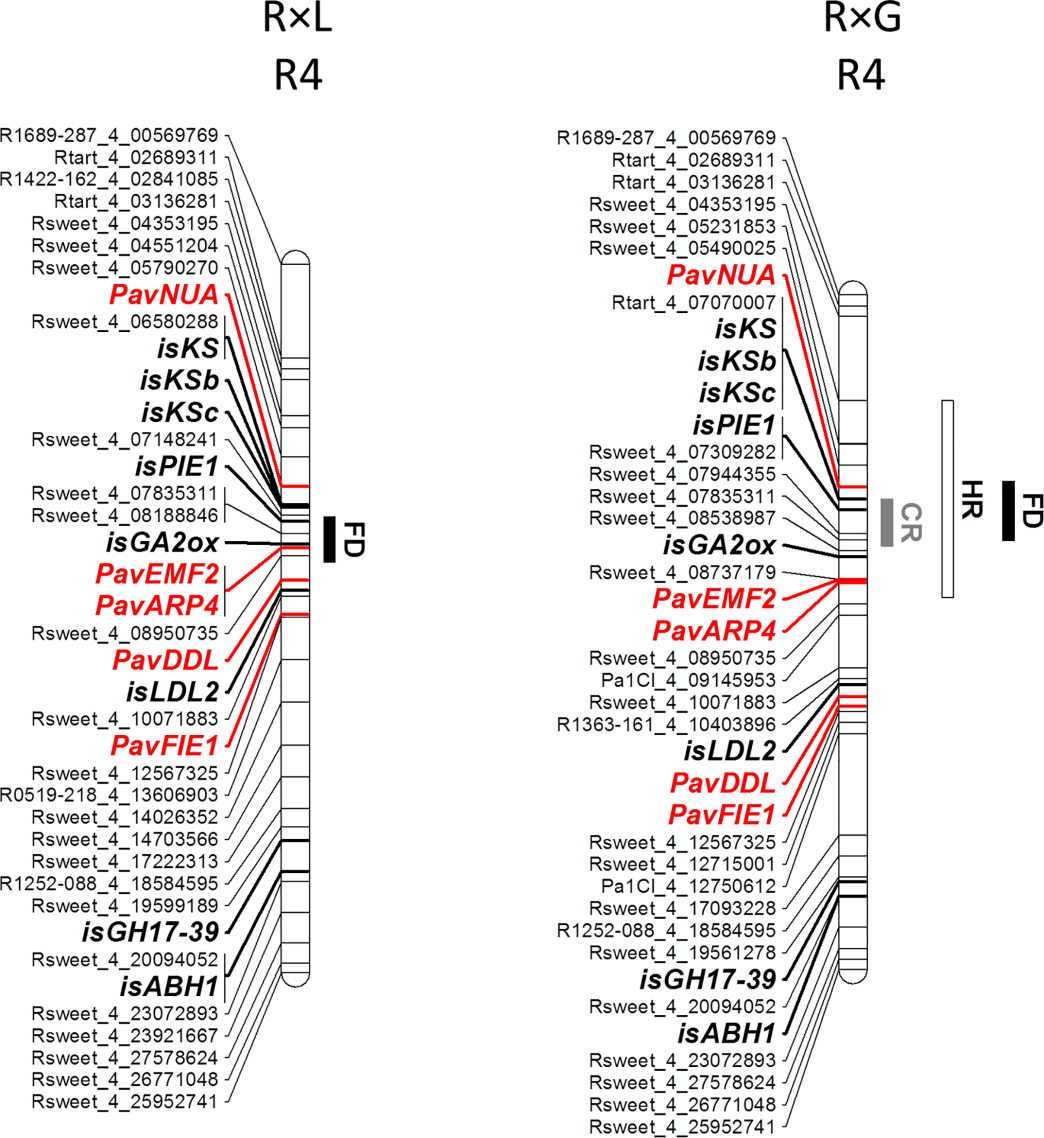
‘Regina’ linkage map of candidate genes on linkage group 4 and QTLs for chilling (CR) and heat requirements (HR) and for flowering date (FD) detected in R×L and R×G progenies. Candidate genes are indicated in bold, those genetically mapped are in red and those mapped *in silico* are in black.

From the 14 CGs located on LG4, 8 are mapped within the small interval of QTLs showing the highest effect for flowering date and for chilling requirements, explaining 36.3% and 17.5% of the phenotypic variation in ‘Regina’ from the R×G progeny, respectively [25]: *PavEMF2*, *PavARP4*, *isGA2ox*, *KAURENE SYNTHASE* (*KS*) CGs (*isKS*, *isKSb*, *isKSc)* and *isPIE1* co-localize with the QTL for flowering date and *isKS*, *isKSb*, *isKSc* and *isPIE1* are also located within the QTL for chilling requirements (Fig 4, S5 Table). All these CGs and *PavNUA* are located within the QTL for heat requirements.

On LG5, *PavVRN2*, *PavDDF1*, *PavFL2*, *isCOP1* and *isGH17-33/65* are located in the QTL region for flowering date.

On LG6, *PavSVP*, *isPRR7* and *isFT* co-localized with the QTL for flowering date and isPRR7 and *isFT* also located in the QTL region for chilling requirements.

On LG7, *PavGH17*, *PavMBF1C* and *isVIL1* located in the QTL region for chilling requirements and flowering date and *PavCLF* located only in the QTL region for flowering date.

On LG8, *PavGI*, *PavSWN*, *PavSAR3*, *isVRN1* and *isFIO1*, co-localized with the QTL for flowering date and the three last CG also for the heat requirements QTL.

### Allelic effect of candidate genes on the phenological traits

The allelic effects of the CGs located in the QTLs with the main effect for chilling and heat requirements, and for flowering date on LG4, were analyzed. Haplotypes were constructed with the three genetically mapped CGs: *PavNUA*, *PavEMF2* and *PavARP4* in R×G and with *PavEMF2* and *PavARP4* in R×L (Table A in S6 Table). The mean values of chilling requirements, heat requirements and flowering date for the two haplotypes were calculated for each year of evaluation. Significant differences between the two haplotypes were found for each year for chilling requirements and flowering date (Table B in S6 Table). For heat requirements, significant differences between the two haplotypes were only found for two of the three years (2010, 2011 but not 2012) (Table B in S6 Table).

The allelic effects of *PavNUA*, *PavEMF2* and *PavARP4* in R×G are significant for all traits and for all years of evaluation, except for heat requirements in 2012 (Table C in S6 Table). The allelic effects of *PavEMF2* and *PavARP4* in R×L progeny are also significant for the flowering date for the 6 years of evaluation. For all traits, the ‘Regina’ haplotype has higher values than the ‘Lapins’ and ‘Garnet’ haplotype. For chilling requirements, differences ranging from 4.17 to 5.32 CP were observed according to the year of evaluation. For heat requirements, the highest difference was observed for P*avEMF2* in 2011: 490 GDH. For flowering date, differences ranging from 1.44 (2011 for R×L) to 3.13 (2008 for R×L) or 4.75 (2009 for R×G) days were observed.

## Discussion

Data on the molecular and genetic control of flowering are scarce in sweet cherry. Consequently, we investigated CGs based on genes involved in dormancy and flowering processes in model plant species such as *Prunus persica*, *Ribes nigrum L*., *Populus trichocarpa*, and *Arabidopsis thaliana*. Starting from a wide list of CGs, we identified and mapped orthologs in sweet cherry and we were able to co-localize a subset of these genes with QTLs for flowering time and chilling and heat requirements.

### Candidate genes mapping

A set of 79 CGs were selected, already reported to be involved in dormancy and flowering processes in several species. Several of them are part of multigenic families (*DAM*, *GH17*, *KS*), increasing the difficulty of designing specific primers to amplify a single CG. For the *DAM* genes, we focused on the *DAM5* and *DAM6* that were shown to be the strongest CGs in peach. Unfortunately no polymorphism was detected within the parental sequences. Concerning the *GH17* family, over 100 putative genes were identified in the *Populus trichocarpa* genome but only a subset of the 10 were analyzed for their effect on dormancy processes [59]. The seven most promising GH17 genes were analyzed in this study, and six primer pairs were designed, the *Populus GH17-33* and *GH17-65* genes being too close to identify sweet cherry orthologs. Among the six *GH17* genes that could be amplified, three were segregating and mapped on different LGs, LG1, LG6 and LG7.

Among all the SNPs detected, 19 SNPs were located in exonic regions. No nonsense mutations were detected but nine of the SNPs induced nonsynonymous substitutions (S3 Table). Three of them induced amino acid polarity changes that may affect the function of the proteins (*CLF*, *VIM1*, *DDL*).

The sequences around the SNPs obtained for each candidate genes (S3 Table) are now available in the GDR and will be used to include these SNPs in the new cherry SNP array that will be constructed with the support of the RosBREED 2 project (https://www.rosbreed.org/). Consequently, it will be soon possible to screen cherry trees for their allelic pattern on these genes.

Thirty four CGs, among the 35 mapped CGs, colocalized with peach orthologs confirming the high synteny between sweet cherry and peach. In addition, these results give good feedback regarding the synteny for the *in silico* mapped CGs, for which no SNPs were detected in the parents of the mapping populations. *PavSAR3* was the only gene that was not mapped in the same LG in cherry (LG8) than in peach (LG2), which may suggest that this gene is present in several copies and the polymorphism was detected only in one locus in sweet cherry. Another hypothesis could be the presence of a translocation between peach and sweet cherry in this region. However, if it is the case, the translocation concerns only a fragment within the two flanking markers Rsweet_8_10229141 and Rsweet_8_10482115, both located on LG8 in peach.

### CG-QTL co-localizations

Most of the analyzed CGs (56 among 79) co-localized within QTLs for chilling or heat requirements, or flowering date (S5 Table). This result can be expected if we consider the wide regions covered by several QTLs (Fig 2). However, the important results are for QTLs with small confidence intervals such as QTLs for the flowering date in LG1 of ‘Lapins’ and in LG4 of ‘Regina’ (Fig 4, S5 Table) covering each 5 and 4 cM, respectively.

At the bottom of LG1, *isDAM5*, *isDAM6* and *PavHYL1* are located within the QTL for flowering date found for the R×L mapping progeny, explaining 8% of the variation of this trait. This region is the same as the *EVG* peach region suggesting a common genetic control between these two *Prunus* species. In peach, *DAM* genes have been proposed as regulators of developmental processes, including seasonal elongation cessation, terminal bud formation [60] and especially dormancy [61]. In particular, *DAM5* and *DAM6* were shown to be strongly regulated by exposure to chilling temperatures over the dormancy period [62].

On LG4, *isKS*, *isKSb*, *isKSc* and *isPIE1* are located within the 4 cM covered by the QTL for chilling requirements detected in ‘Regina’ (S3 Table, Fig 4)’. The QTL for heat requirements covers a larger region (16 cM) that includes *isKS*, *isKSb*, *isKSc* and *isPIE1* but also *PavNUA*, *PavEMF2*, *PavARP4* and *isGA2ox*. For flowering date, a total of eight CGs co-localized with this major QTL in LG4. *PavEMF2*, *PavARP4* and *isGA2ox* mapped within the 3.3 cM QTL region of ‘Regina’ (from the R×L progeny) possibly explaining 21.2% of the phenotypic variation for flowering date, and *isKS*, *isKSb*, *isKSc* and *isPIE1* mapped within the 4.1 cM QTL region of ‘Regina’ (from R×G progeny) possibly explaining 36.3% of the phenotypic variation [25].

These genes are orthologs of genes known to be involved in the temperature pathway (*ARP4*, *EMF2*, *NUA* and *PIE1*) and gibberellins pathway (*KS*, *KSb*, *KSc* and GA2ox). In *Arabidopsis thaliana*, *ARP4*, *EMF2* and *PIE1*, were reported to be involved in epigenetic regulation [17, 63, 64]. GA2ox is an enzyme related to gibberellin inactivation [65]. Increases in gibberellin levels were reported to be necessary for endodormancy release [66]. Transcriptome analysis of Japanese pear flower buds at endodormancy and endodormancy release stages revealed that transcripts of *GA2ox* were up-regulated in ecodormancy [67].

No CG of the photoperiod pathway was found in the major QTL of LG4, which might suggest that photoperiod has little or no effect on flowering time in sweet cherry as it was already reported in several genus of the Rosaceae family: *Malus*, *Pyrus* and *Sorbus* [22, 68]. However, as CGs involved in the photoperiod pathway were mapped in QTLs on other LGs (*ELF3*, *FRS5* and *FYPP3* in LG1, *CRP* in LG2, *FRS6* in LG3, *COP1* in LG5) the effect of the photoperiod on flowering time cannot be excluded. Heide [23] showed a pronounced photoperiod/temperature interaction on the control of growth cessation and resumption in several *Prunus* species including *P*. *avium*.

No CG was located in the QTLs for flowering date in ‘Garnet’ LG2 and LG6 and in the QTL for heat requirements in ‘Garnet’ LG6 suggesting that additional CGs may be involved in the flowering and dormancy processes. Another hypothesis could be that genes not already reported to be involved in dormancy or flowering processes may play a significant role in sweet cherry. However, interestingly in ‘Regina’ LG2, *SOC1* was located *in silico* in the QTL for chilling requirements and for flowering date. This gene was reported to be involved in chilling requirements in apricot [69].

### CGs and sweet cherry breeding programs

The allelic compositions of *PavNUA*, *PavEMF2* and *PavARP4* had a significant effect on the three traits, chilling and heat requirements in the R×G progeny and on flowering date on the two progenies (R×L and R×G). Consequently, we showed that it is possible to select individuals for low/high chilling requirements and early/late flowering time in these progenies by using the SNP markers identified in these CGs. These markers represent a potential tool for breeding programs using marker assisted selection. This selection could be promising for regions where chilling units are not sufficient to fulfill chilling requirements of sweet cherry cultivars. Also, selection for late flowering could help to mitigate spring frost risk in cold winter regions.

## Conclusions

In this study we analyzed a large set of CGs already reported to be involved in flowering and dormancy processes in several species. SNP markers were identified and used to map 39 CGs in sweet cherry progenies. Based on the high synteny between peach and sweet cherry, CGs that were not polymorphic in the parents were mapped *in silico* using the peach genome sequence. We showed the first co-localization between CGs and QTLs for temperature requirements and flowering date in sweet cherry. Most of the selected CGs were located in QTL regions, making them a useful resource for future bud dormancy and flowering network analyses. Those located in QTLs of high effect with a small confidence interval, in the LG4 QTLs for example, are very promising and may be useful to select new varieties adapted to diverse climatic conditions. Moreover, further analyses including expression analyses of CGs located in QTL regions and association genetics could also provide new understanding on processes involved in the control of dormancy and flowering time.

## Supporting Information

S1 Fig Linkage maps of ‘Regina’ and ‘Lapins’ constructed based on the segregation analysis in the R×L progeny. Linkage maps were constructed using JoinMap® 4.0 [53]. A minimum LOD value of 3 and a recombinant threshold of 0.35 were chosen for the mapping. Kosambi’s mapping function was used to convert recombination frequency into map distance [54]. The graphical presentation of linkage maps was performed using the MapChart software version 2.2 [56]. The candidate genes are indicated in red. The genetics distances are indicated in cM.
(TIF)

S2 Fig Linkage maps of ‘Regina’ and ‘Garnet’ constructed based on the segregation analysis in the R×G progeny. Linkage maps were constructed using JoinMap® 4.0 [53]. A minimum LOD value of 3 and a recombinant threshold of 0.35 were chosen for the mapping. Kosambi’s mapping function was used to convert recombination frequency into map distance [54]. The graphical presentation of linkage maps was performed using the MapChart software version 2.2 [56]. The candidate genes are indicated in red. The genetics distances are indicated in cM.
(TIF)

S1 File Sweet cherry cDNA sequences of candidate genes referred as PRUAVXXXXX in S1 Table. (TXT)

S2 File Alignment of candidate genes sequences: peach genomic (ppa), sweet cherry ‘Regina’ cDNA (PRUAV) and PCR sweet cherry genomic amplified fragments of the three parents (Pav for ‘Regina’, ‘Lapins’, and ‘Garnet’). Primers designed on peach are highlighted in red, and those designed on cherry are highlighted in yellow. Sequences used for the genotyping by SEQUENOM are highlighted in green and SNP detected are indicated in pink. K: [G/T]; M: [A/C]; R: [A/G]; S: [C/G]; W: [A/T];Y: [C/T]; N: [A/T/C/G].
(DOCX)

S1 Table List of candidate genes involved in the regulation of dormancy and flowering date in *Prunus* species, black currant (*Ribes nigrum L*.), poplar (*Populus trichocarpa*) and *Arabidopsis thaliana*- Orthologous genes in peach and sweet cherry are indicated (sweet cherry orthologous sequences are available in the Genome Database for Rosaceae (GDR) ^http://www.rosaceae.org/^).
(XLSX)

S2 Table Primers used to amplify sweet cherry candidate genes. (XLSX)

S3 Table Candidate genes mapping in peach and sweet cherry. SNPs used for the genetic mapping in sweet cherry and the genotype for each parent are detailed- The *in silico* position of each candidate gene in the *Prunus persica* physical map is referred according to the Peach genome v2.0.a1. The linkage group and the parent where the candidate gene is genetically mapped are reported for *Prunus avium*.
(XLSX)

S4 Table Candidate genes mapping position. (XLSX)

S5 Table Candidate genes located in QTLs for flowering date detected in 'Regina' ×'Lapins' (R×L) and 'Regina' ×'Garnet' (R×G) progenies and in QTLs for chilling and heat requirements detected in R×G- The physical positions of the confidence interval of the QTLs are referred according to the Peach genome v2.0.a1 based on the physical position of the closest SNP markers**.**
(XLSX)

S6 Table Haplotype analysis of candidate genes spanning in the QTLs on linkage group 4. (A) Candidate genes used to characterize the haplotypes. (B) Mean value of each trait for the two haplotypes in both mapping progenies. Number of observations is indicated in parenthesis. Significant differences between means (P>0,05) are indicated by a star. (C) Effect of the candidate gene allele on the traits.
(XLSX)
