# Supplementary figures and images for: Mapping of Candidate Genes Involved in Bud Dormancy and Flowering Time in Sweet Cherry (Prunus avium)
Source: PLoS One. 2015 Nov 20;10(11):e0143250. doi: 10.1371/journal.pone.0143250 (PMC4654497; doi:10.1371/journal.pone.0143250)

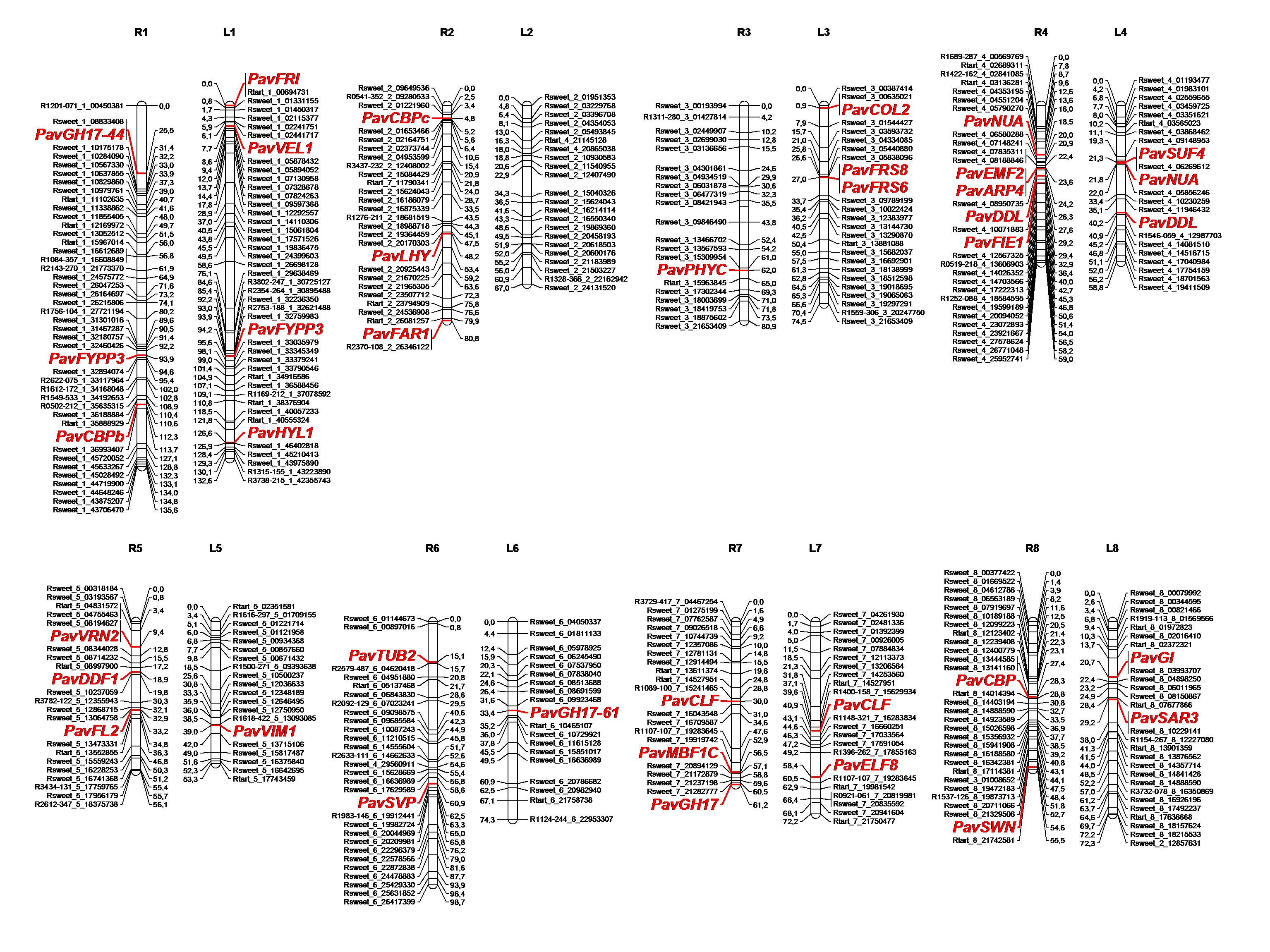

Supplement: S1 Fig — Linkage maps were constructed using JoinMap® 4.0 [53]. A minimum LOD value of 3 and a recombinant threshold of 0.35 were chosen for the mapping. Kosambi’s mapping function was used to convert recombination frequency into map distance [54]. The graphical presentation of linkage maps was performed using the MapChart software version 2.2 [56]. The candidate genes are indicated in red. The genetics distances are indicated in cM. (TIF) [file pone.0143250.s001.tif]

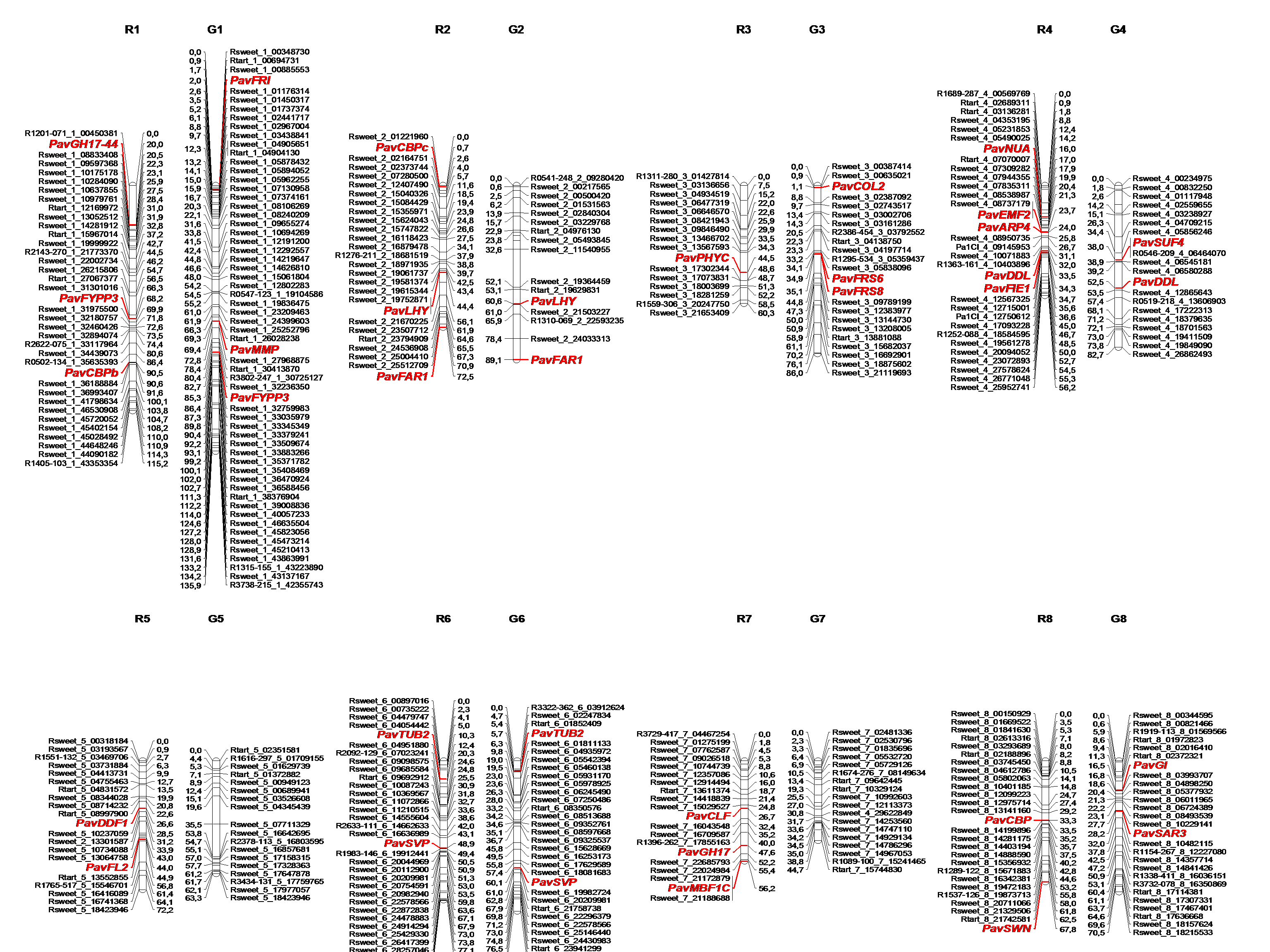

Supplement: S2 Fig — Linkage maps were constructed using JoinMap® 4.0 [53]. A minimum LOD value of 3 and a recombinant threshold of 0.35 were chosen for the mapping. Kosambi’s mapping function was used to convert recombination frequency into map distance [54]. The graphical presentation of linkage maps was performed using the MapChart software version 2.2 [56]. The candidate genes are indicated in red. The genetics distances are indicated in cM. (TIF) [file pone.0143250.s002.tif]
