# Supplementary material for: Mapping of Candidate Genes Involved in Bud Dormancy and Flowering Time in Sweet Cherry (Prunus avium)
Source: PLoS One. 2015 Nov 20;10(11):e0143250. doi: 10.1371/journal.pone.0143250 (PMC4654497; doi:10.1371/journal.pone.0143250)
Supplement: S2 File — Primers designed on peach are highlighted in red, and those designed on cherry are highlighted in yellow. Sequences used for the genotyping by SEQUENOM are highlighted in green and SNP detected are indicated in pink. K: [G/T]; M: [A/C]; R: [A/G]; S: [C/G]; W: [A/T];Y: [C/T]; N: [A/T/C/G]. (DOCX) [file pone.0143250.s004.docx]

**S2 File.** Alignment of candidate genes sequences: peach genomic (ppa) with the position from the beginning of the predicted gene sequence, sweet cherry ‘Regina’ cDNA (PRUAV) and PCR sweet cherry genomic amplified fragments of the three parents (Pav for ‘Regina’, ‘Lapins’, and ‘Garnet’). Primers designed on peach are highlighted in red, and those designed on cherry are highlighted in yellow. Sequences used for the genotyping by SEQUENOM are highlighted in green and SNP detected are indicated in pink. K: [G/T]; M: [A/C]; R: [A/G]; S: [C/G]; W: [A/T];Y: [C/T]; N: [A/T/C/G]

***ARP4***

|  |  | ....|....|....|....|....|....|....|....|....|....|....|....|....|....|....|....|....|....|....|....| |
| --- | --- | --- |
| ppa006685m | 1441 | AGGGAATGCCTATTGGTTGATCCTAAAGAGCATCCAATGCTACTTGCAGAGCCATCTTCTAACACTCAACAACAGAGAGAAAGGTAAGCTAGCAATTATT |
| PRUAV010449 |  | ~~GGAATGCCTATTGGTTGATCCTAAAGAGCATCCAATGCTACTTGCAGAGCCATCTTCTAACACTCAACAACAGAGAGAAAG~~~~~~~~~~~~~~~~~ |
| PavARP4_‘Regina’ |  | ~~~~~~~~~~~~~~~~~~~~~~~~~~~~~~~~~~~~~~~~~~~~~~~~~~~~~~~~~~~ggtgacaAC~~~~CAGAGAG~AAGGTAAGCTAGCAATTCTT |
| PavARP4_‘Lapins’ |  | ~~~~~~~~~~~~~~~~~~~~~~~~~~~~~~~~~~~~~~~~~~~~~~~~~~~~~~~~~~~gcgcACA~~~~~~CAGAGAG~~AGGTAAGCTAGCAATTCTT |
| PavARP4_‘Garnet’ |  | ~~~~~~~~~~~~~~~~~~~~~~~~~~~~~~~~~~~~~~~~~~~~~~~~~~~~~~~~~~~cctgacaaC~~~~CAGAGAG~AAGGTAAGCTAGCAATTCTT |
|  |  |  |
|  |  | ....|....|....|....|....|....|....|....|....|....|....|....|....|....|....|....|....|....|....|....| |
| ppa006685m |  | AAATTTTCTCATTAGCTCAAAATTGTGACTATCTAGTTAAACTAAAATGCTTATGTAATTGTTAATGTATTCTTTTGTGACAAACTTGGACTTCACGGTC |
| PRUAV010449 |  | ~~~~~~~~~~~~~~~~~~~~~~~~~~~~~~~~~~~~~~~~~~~~~~~~~~~~~~~~~~~~~~~~~~~~~~~~~~~~~~~~~~~~~~~~~~~~~~~~~~~~ |
| PavARP4_‘Regina’ |  | AAATTTTCTCATTAGCTCACAATTGTGACTCTCTAGTTAAACCAAAATGCTTATGTAATTGTTAATGTATTCTTTTGTGACAAACATGGACTTCACGGTC |
| PavARP4_‘Lapins’ |  | AAATTTTCTCATTAGCTCACAATTGTGACTCTCTAGTTAAACCAAAATGCTTATGTAATTGTTAATGTATTCTTTTGTGACAAACATGGACTTCACGGTC |
| PavARP4_‘Garnet’ |  | AAATTTTCTCATTAGCTCACAATTGTGACTCTCTAGTTAAACCAAAATGCTTATGTAATTGTTAATGTATTCTTTTGTGACAAACATGGACTTCACGGTC |
|  |  |  |
|  |  | ....|....|....|....|....|....|....|....|....|....|....|....|....|....|....|....|....|....|....|....| |
| ppa006685m |  | AAGGATAAAAACATCCGCCATACAGATAGCTTAAATAAGGGGGTATTTT~GGTTATTAGGGTTGGGTGTGCCATCCTTAAGTTTTTTTCCTCTACTTTCT |
| PRUAV010449 |  | ~~~~~~~~~~~~~~~~~~~~~~~~~~~~~~~~~~~~~~~~~~~~~~~~~~~~~~~~~~~~~~~~~~~~~~~~~~~~~~~~~~~~~~~~~~~~~~~~~~~~ |
| PavARP4_‘Regina’ |  | AAGGATAAAAATATCCACCGTACAGATAGCTTAAATAAGGGGGTTTTTTTGGTTATTAGGGTTGGATCATGTCGGTGGAAATCAAGCATTTTTGCTCTGT |
| PavARP4_‘Lapins’ |  | AAGGATAAAAATATCCACCGTACAGATAGCTTAAATAAGGGGGTTTTTTTGGTTATTAGGGTTGGATCATGTCGGTGGAAATCAAGCATTTTTGCTCTGT |
| PavARP4_‘Garnet’ |  | AAGGATAAAAATATCCACCGTACAGATAGCTTAAATAAGGGGGTTTTTTTGGTTATTAGGGTTGGATCATGTCGGTGGAAATCAAGCATTTTTGCTCTGT |
|  |  |  |
|  |  | ....|....|....|....|....|....|....|....|....|....|....|....|....|....|....|....|....|....|....|....| |
| ppa006685m |  | TTCTGTTTGGATCATGTCAGTGGAAATCAAGCATTTTTGCTCTGTTAAAGGCCGATAGCAGGGAAATCTTGGGAATATAGGGGAACCTTTGCTATATCAG |
| PRUAV010449 |  | ~~~~~~~~~~~~~~~~~~~~~~~~~~~~~~~~~~~~~~~~~~~~~~~~~~~~~~~~~~~~~~~~~~~~~~~~~~~~~~~~~~~~~~~~~~~~~~~~~~~~ |
| PavARP4_‘Regina’ |  | TGAAGGCAGATATCAGGGAAATCTCGGGAATATAGGGGAACCTTTGCTATATCAGGCAAATTTCT~~~~GGGATAGAGGGGAAGAACCACACTAT~~CAG |
| PavARP4_‘Lapins’ |  | TGAAGGCAGATATCAGGGAAATCTCGGGAATATAGGGGAACCTTTGCTATATCAGGCAAATTTCT~~~~GGGATAGAGGGGAAGAACCACACTAT~~CAG |
| PavARP4_‘Garnet’ |  | TGAAGGCAGATATCAGGGAAATCTCGGGAATATAGGGGAACCTTTGCTATATCAGGCAAATTTCT~~~~GGGATAGAGGGGAAGAACCACACTAT~~CAG |
|  |  |  |
|  |  | ....|....|....|....|....|....|....|....|....|....|....|....|....|....|....|....|....|....|....|....| |
| ppa006685m |  | GAAAATTTCTGGGATAGAGGGGAAGAACTTGTTTCAACCCCACTATCAGTATCAGTGTCTTGATATGTTGCATACTGATGTTCCTGTACTATTGTTACAT |
| PRUAV010449 |  | ~~~~~~~~~~~~~~~~~~~~~~~~~~~~~~~~~~~~~~~~~~~~~~~~~~~~~~~~~~~~~~~~~~~~~~~~~~~~~~~~~~~~~~~~~~~~~~~~~~~~ |
| PavARP4_‘Regina’ |  | TATCAGTGTCTTGATTA~~~~~~~~~~~~TGTT~~~~~~~~~~~~~~~~~GCATACTGATGTATTCATT~~~~~~~~ATGTTCCTGTACCATTGTTGCAT |
| PavARP4_‘Lapins’ |  | TATCAGTGTCTTGATTA~~~~~~~~~~~~TGTT~~~~~~~~~~~~~~~~~GCATACTGATGTATTCATT~~~~~~~~ATGTTCCTGTACCATTGTTGCAT |
| PavARP4_‘Garnet’ |  | TATCAGTGTCTTGATTA~~~~~~~~~~~~TGTT~~~~~~~~~~~~~~~~~GCATACTGATGTATTCATT~~~~~~~~ATGTTCCTGTACCATTGTTGCAT |
|  |  |  |
|  |  | ....|....|....|....|....|....|....|....|....|....|....|....|....|....|....|....|....|....|....|....| |
| ppa006685m |  | ACTTTGATTTTGTTGTTTTAAAGTTTTTAACTTGTAATAATTATGCCCAACCCACTTGATAAAAGGTTTTGATATAAATGTTATTATCTGCTCCATCGTT |
| PRUAV010449 |  | ~~~~~~~~~~~~~~~~~~~~~~~~~~~~~~~~~~~~~~~~~~~~~~~~~~~~~~~~~~~~~~~~~~~~~~~~~~~~~~~~~~~~~~~~~~~~~~~~~~~~ |
| PavARP4_‘Regina’ |  | ACTTTGATTTTGTTGTTTTAAAGTTTTTAACTTGTAATAATTATGCCCAACCCACTTGATAAAAGGTTTTGATCTAAATGGTATTATCTGCTCCATCATT |
| PavARP4_‘Lapins’ |  | ACTTTGATTTTGTTGTTTTAAAGTTTTTAACTTGTAATAATTATGCCCAACCCACTGGATAAAAGGTTTTGATCTAAATGGTATTATCTGCTCCATCATT |
| PavARP4_‘Garnet’ |  | ACTTTGATTTTGTTGTTTTAAAGTTTTTAACTTGTAATAATTATGCCCAACCCACTGGATAAAAGGTTTTGATCTAAATGGTATTATCTGCTCCATCATT |
|  |  |  |
|  |  | ....|....|....|....|....|....|....|....|....|....|....|....|....|....|....|....|....|....|....|....| |
| ppa006685m |  | ATTTATGGTTTAATTGGTGTGTCTTTATATTTTTAGTTTTTATGTTAAGTGATTGAGGTTTGTCTTGTACCTGACAGGACGGCAGAGCTTATGTTTGAAA |
| PRUAV010449 |  | ~~~~~~~~~~~~~~~~~~~~~~~~~~~~~~~~~~~~~~~~~~~~~~~~~~~~~~~~~~~~~~~~~~~~~~~~~~~~~GACGGCGGAGCTTATGTTTGAAA |
| PavARP4_‘Regina’ |  | ATTTATGGTTTAATTGGTGTGTCTATATGTTTTTAGTTATTATGTTCAGTGATTAAGGTTTGTCTTGTACCTGACAGGACGGCGGAGCTTATGTTTGAAA |
| PavARP4_‘Lapins’ |  | ATTTATGGTTTAATTGGTGTGTCTATATGTTTTTAGTTATTATGTTCAGTGATTAAGGTTTGTCTTGTACCTGACAGGACGGCGGAGCTTATGTTTGAAA |
| PavARP4_‘Garnet’ |  | ATTTATGGTTTAATTGGTGTGTCTATATGTTTTTAGTTATTATGTTCAGTGATTAAGGTTTGTCTTGTACCTGACAGGACGGCGGAGCTTATGTTTGAAA |
|  |  |  |
|  |  | ....|....|....|....|....|....|....|....|....|....|....|....|....|....|....|....|....|....|....|....| |
| ppa006685m |  | AGTACAAAGTTCCTGCCTTATTTTTGGCCAAGAATGCTGTATGCCCC~TCTTCATCTCATCTCCTTGATTCCAACTTGTAGAACATGGATTAGTATTACA |
| PRUAV010449 |  | AGTACAAAGTTCCTGCCTTATTTTTGGCCAAGAATGCT~~~~~~~~~~~~~~~~~~~~~~~~~~~~~~~~~~~~~~~~~~~~~~~~~~~~~~~~~~~~~~ |
| PavARP4_‘Regina’ |  | AGTACAAAGTTCCTGCCTTATTTTTGGCCAAGAATGCTGTATGTCYCCTCTTCATCTCATCTCCTTGATTCCAACTTGTAGAACATGGATTAGTATTACA |
| PavARP4_‘Lapins’ |  | AGTACAAAGTTCCTGCCTTATTTTTGGCCAAGAATGCTGTATGTCCCCTCTTCATCTCATCTCCTTGATTCCAACTTGTAGAACATGGATTAGTATTACA |
| PavARP4_‘Garnet’ |  | AGTACAAAGTTCCTGCCTTATTTTTGGCCAAGAATGCTGTATGTCCCCTCTTCATCTCATCTCCTTGATTCCAACTTGTAGAACATGGATTAGTATTACA |
|  |  |  |
|  |  | ....|....|....|....|....|....|....|....|....|....|....|....|....|....|....|....|....|....|....|....| |
| ppa006685m |  | GAATCTGTTCTTTTCTTCCTTTTAAAA~TCAGTCTGCAATTGGTGAGTAACTGGAATACTTTTGATGCCAGGTTCTCACATCTTTTGC~ATCAGGCCGTG |
| PRUAV010449 |  | ~~~~~~~~~~~~~~~~~~~~~~~~~~~~~~~~~~~~~~~~~~~~~~~~~~~~~~~~~~~~~~~~~~~~~~~GTTCTCACATCTTTTGC~ATCAGGCCGTG |
| PavARP4_‘Regina’ |  | GAATCTGTTCTTTTGTTCCTTTTAAAAATCAGTCTGCAATTGGTGATTAACTGGAATACTTTTGATGCCAGGTTCTCACATCTTTTGCCATCAGGCAAAA |
| PavARP4_‘Lapins’ |  | GAATCTGTTCTTTTGTTCCTTTTAAAAATCAGTCTGCAATTGGTGATTAACTGGAATACTTTTGATGCCAGGTTCTCACATCTTTTGCCATCAGGCAAA |
| PavARP4_‘Garnet’ |  | GAATCTGTTCTTTTGTTCCTTTTAAAAATCAGTCTGCAATTGGTGATTAACTGGAATACTTTTGATGCCAGGTTCTCACATCTT~GGCCATCAGGCA |

***BRI1***

|  |  | ....|....|....|....|....|....|....|....|....|....|....|....|....|....|....|....|....|....|....|....| |
| --- | --- | --- |
| ppa000566m | 3501 | CGAGAGCGTTGAGATTGAGCTTTTACAGCATTTAAAGGTAGCTTGTGCTTGTTTGGAGGATAGGGCATGGCGGCGTCCCACAATGATTCAAGTCATGGCA |
| PRUAV002156 |  |  |
| PavBRI1_‘Regina’ |  | ~~~~~~~~~~~~~~~~~~~~~~~~~~~~~~~~~~~~~~~~~~~~~~~~~~~~~~~~~~~~~~~~~~~~~~~~~~~~~~~~~~~~~~~~~~~~~~~~~~~~ |
| PavBRI1_‘Lapins’ |  | ~~~~~~~~~~~~~~~~~~~~~~~~~~~~~~~~~~~~~~~~~~~~~~~~~~~~~~~~~~~~~~~~~~~~~~~~~~~~~~~~~~~~~~~~~~~~~~~~~~~~ |
| PavBRI1_‘Garnet’ |  | ~~~~~~~~~~~~~~~~~~~~~~~~~~~~~~~~~~~~~~~~~~~~~~~~~~~~~~~~~~~~~~~~~~~~~~~~~~~~~~~~~~~~~~~~~~~~~~~~~~~~ |
|  |  |  |
|  |  | ....|....|....|....|....|....|....|....|....|....|....|....|....|....|....|....|....|....|....|....| |
| ppa000566m |  | ATGTTCAAGGAAATCCAAACAGGGTCCGGGATTGACTCTCAATCAACCATAGCCACTGACGACGGAGGTTTTGGTGCAGTTGAAATGGTAGAGATGAGCA |
| PRUAV002156 |  |  |
| PavBRI1_‘Regina’ |  | ~~~~~~~~~~~~~~~~~~~~~gggtctGGGATTGACTCTC~ATC~ACCATAGCCACTGACGACGGAGGTTTTGGTGCGGTTGAAATGGTAGAAATGAGCA |
| PavBRI1_‘Lapins’ |  | ~~~~~~~~~~~~~~~~~~~~cgctctggGCATTGACTCTC~ATCAACCATAGCCACTGACGACGGAGGTTTTGGTGCGGTTGAAATGGTAGAAATGAGCA |
| PavBRI1_‘Garnet’ |  | ~~~~~~~~~~~~~~~~~~~~ggcactGGGCATTGACTCTC~ATCAACCATAGCCACTGACGACGGAGGTTTTGGTGCGGTTGAAATGGTAGAAATGAGCA |
|  |  |  |
|  |  | ....|....|....|....|....|....|....|....|....|....|....|....|....|....|....|....|....|....|....|....| |
| ppa000566m |  | TAAAAGAAGTCCCTGAAAGCAAGCAGTAGCCCAAAAAGCCAAGCCAATTTTCAAATGAATTCTTTGGAAGAAAGAAGAAACAAGGTGGAGATGATACTTT |
| PRUAV002156 |  |  |
| PavBRI1_‘Regina’ |  | TAAAAGAAGTCCCTGAAAGCAAGCAGTAGCCCAAAAAGCCATGCCAATTTTCAAGAGAATTCTTTGGAAGAAAGAAGAAACAAGGTGGAGATGAAACTTT |
| PavBRI1_‘Lapins’ |  | TAAAAGAAGTCCCTGAAAGCAAGCAGTAGCCCAAAAAGCCATGCCAATTTTCAAGAGAATTCTTTGGAAGAAAGAAGAAACAAGGTGGAGATGAAACTTT |
| PavBRI1_‘Garnet’ |  | TAAAAGAAGTCCCTGAAAGCAAGCAGTAGCCCAAAAAGCCATGCCAATTTTCAAGAGAATTCTTTGGAAGAAAGAAGAAACAAGGTGGAGATGATACTTT |
|  |  |  |
|  |  | ....|....|....|....|....|....|....|....|....|....|....|....|....|....|....|....|....|....|....|....| |
| ppa000566m |  | CAGCTCCCCCAATTTTTTTCCCTCTTCTTTACAGCTTCAAATGCTGTGAAGCTGCCTGTTTTGCTGTACCATTTAATGTATGTAATCTTGTTATTTATAC |
| PRUAV002156 |  |  |
| PavBRI1_‘Regina’ |  | CAGCTCCCCCAATTTT |
| PavBRI1_‘Lapins’ |  | CAGCTCCCCCAATTTT |
| PavBRI1_‘Garnet’ |  | CAGCTCCCCCAATTAT |

***CAC1***

|  |  | ....|....|....|....|....|....|....|....|....|....|....|....|....|....|....|....|....|....|....|....| |
| --- | --- | --- |
| ppa009593m | 278 | AAAGCATGCTCTCCCCCCATGGCTCGCCTTGGTTCTACTGCCCAAACTTCGCAGCCGCAGCAGCACCTGCTTTCCTTTCACAACTATCTCAATCGCTGTC |
| PRUAV000620 |  | aaagcatgctctgcccccatggctcgccttggctctactgcccaaacttcgcacccgcagcagcacctgctttcctttcacaactgtctcaatcgctgtc |
| PavCAC_‘Regina’ |  | ~~~~~~~~~~~~~~~~~~~~~~~~~~~~~~~~~~~~~~~~~~~~~~~~~~~~~~~~~~~~~~~ccatgcgttTCCTTTC~CAACTGTCTCAATCGCTGTC |
| PavCAC_‘Lapins’ |  | ~~~~~~~~~~~~~~~~~~~~~~~~~~~~~~~~~~~~~~~~~~~~~~~~~~~~~~~~~~~~~~~ccctgCCATT~CTTTC~CA~CTGTCTCAATCGCTGTC |
| PavCAC_‘Garnet’ |  | ~~~~~~~~~~~~~~~~~~~~~~~~~~~~~~~~~~~~~~~~~~~~~~~~~~~~~~~cggccctggggtgttcacTCTGAC~CA~CTGTCTCAATCGCTGTC |
|  |  |  |
|  |  | ....|....|....|....|....|....|....|....|....|....|....|....|....|....|....|....|....|....|....|....| |
| ppa009593m |  | CCATTCCCTCTCTGCAGATTTCTGGGCTTCAGGTTTGGTTAATTCGACATACCTTCTACTTTACTCCCATTTGTGAATGTAAAATTTTTCACTTTACGAG |
| PRUAV000620 |  | ccattccctctctgcagatttctgggcttcag~~~~~~~~~~~~~~~~~~~~~~~~~~~~~~~~~~~~~~~~~~~~~~~~~~~~~~~~~~~~~~~~~~~~ |
| PavCAC_‘Regina’ |  | CCATTCCCTCTCTGCAGATTTCTGGGCTTCAGGTTTGGTTAATTCGACAGACCACCTACTTTACTCCACTTTGTGAATGTAAATTTTTTCATTTTACGAG |
| PavCAC_‘Lapins’ |  | CCATTCCCTCTCTGCAGATTTCTGGGCTTCAGGTTTGGTTAATTCGACAGACCACCTACTTTACTCCACTTTGTGAATGTAAATTTTTTCATTTTACGAG |
| PavCAC_‘Garnet’ |  | CCATTCCCTCTCTGCAGATTTCTGGGCTTCAGGTTTGGTTAATTCGACAGACCACCTACTTTACTCCACTTTGTGAATGTAAATTTTTTCATTTTACGAG |
|  |  |  |
|  |  | ....|....|....|....|....|....|....|....|....|....|....|....|....|....|....|....|....|....|....|....| |
| ppa009593m |  | TCTTCCTTGTTAAATTTTGCTTCTTTTCTCTTACCCTTGTTACTTGCCGTATACTGTCCTGTTCAGTCGTTAATTATGGATTTCTTTTATGTTTGAAATG |
| PRUAV000620 |  | ~~~~~~~~~~~~~~~~~~~~~~~~~~~~~~~~~~~~~~~~~~~~~~~~~~~~~~~~~~~~~~~~~~~~~~~~~~~~~~~~~~~~~~~~~~~~~~~~~~~~ |
| PavCAC_‘Regina’ |  | TCTTCCTTGTTAAATTTTGCTTCTTTTCTCTTACCCTTGTTACTTGCCATATCCTGTCCTGTTCAGCCGTTAATTATGGATTTCTCTTATGTTCGAAATG |
| PavCAC_‘Lapins’ |  | TCTTCCTTGTTAAATTTTGCTTCTTTTCTCTTACCCTTGTTACTTGCCATATCCTGTCCTGTTCAGCCGTTAATTATGGATTTCTCTTATGTTCGAAATG |
| PavCAC_‘Garnet’ |  | TCTTCCTTGTTAAATTTTGCTTCTTTTCTCTTACCCTTGTTACTTGCCATATCCTGTCCTGTTCAGCCGTTAATTATGGATTTCTCTTATGTTCGAAATG |
|  |  |  |
|  |  | ....|....|....|....|....|....|....|....|....|....|....|....|....|....|....|....|....|....|....|....| |
| ppa009593m |  | GGTTTCATATGTATATAATTATGTATGCATCATGTGTAACACATATAATATATAAGAAGCTGACAGTTGGTGGATATGCATTGAAATTGATGTAGTTGAA |
| PRUAV000620 |  | ~~~~~~~~~~~~~~~~~~~~~~~~~~~~~~~~~~~~~~~~~~~~~~~~~~~~~~~~~~~~~~~~~~~~~~~~~~~~~~~~~~~~~~~~~~~~~~~~~~~~ |
| PavCAC_‘Regina’ |  | GGTTTCATATGTATATGGTTATATATGCATCATGTGTAACACATATAATATATAAGAAGCTGACAGTTGGTGGGTATGCATTGAAATTGATGTAGTTGAA |
| PavCAC_‘Lapins’ |  | GGTTTCATATGTATATGGTTATATATGCATCATGTGTAACACATATAATATATAAGAAGCTGACAGTTGGTGGGTATGCATTGAAATTGATGTAGTTGAA |
| PavCAC_‘Garnet’ |  | GGTTTCATATGTATATGGTTATATATGCATCATGTGTAACACATATAATATATAAGAAGCTGACAGTTGGTGGGTATGCATTGAAATTGATGTAGTTGAA |
|  |  |  |
|  |  | ....|....|....|....|....|....|....|....|....|....|....|....|....|....|....|....|....|....|....|....| |
| ppa009593m |  | GAATTGATAGTTACATCACATGTAGAATTTTGATTGAGCCGTAACCCAGAAAGGATTCTATTCAAAATGATGTCTTTCATGTACACCTGCAAGCTACGAC |
| PRUAV000620 |  | ~~~~~~~~~~~~~~~~~~~~~~~~~~~~~~~~~~~~~~~~~~~~~~~~~~~~~~~~~~~~~~~~~~~~~~~~~~~~~~~~~~~~~~~~~~~~~~~~~~~~ |
| PavCAC_‘Regina’ |  | GAATTGATAGCTGCATCACATGTAGGATTTTGATTGAGTTGTAACCCAGAAAGGATTCTAGTCAAAATGATGTCTTTCATGTACACCTGCAAGTTACGAC |
| PavCAC_‘Lapins’ |  | GAATTGATAGCTGCATCACATGTAGGATTTTGATTGAGTTGTAACCCAGAAAGGATTCTAGTCAAAATGATGTCTTTCATGTACACCTGCAAGTTACGAC |
| PavCAC_‘Garnet’ |  | GAATTGATAGCTGCATCACATGTAGGATTTTGATTGAGTTGTAACCCAGAAAGGATTCTAGTCAAAATGATGTCTTTCATGTACACCTGCAAGTTACGAC |
|  |  |  |
|  |  | ....|....|....|....|....|....|....|....|....|....|....|....|....|....|....|....|....|....|....|....| |
| ppa009593m |  | CGATTAATGATCACCATAGTCATATACTGTACTGTCATTTATCTATAGCTTTATGTTGCAGCGACAGTTCTATGTACATCGAAATGTTGCTGCTATTCTT |
| PRUAV000620 |  | ~~~~~~~~~~~~~~~~~~~~~~~~~~~~~~~~~~~~~~~~~~~~~~~~~~~~~~~~~~~~~~~~~~~~~~~~~~~~~~~~~~~~~~~~~~~~~~~~~~~~ |
| PavCAC_‘Regina’ |  | CGATTAATGATCACCATAGTCATATACTGTACTGTCATTTATCTATAGCTTTTTGTTGCAGCGACAGTTCTATGTACAGTGAAATGTTGCTGCTATTCCT |
| PavCAC_‘Lapins’ |  | CGATTAATGATCACCATAGTCATATACTGTACTGTCATTTATCTATAGCTTTTTGTTGCAGCGACAGTTCTATGTACAGTGAAATGTTGCTGCTATTCCT |
| PavCAC_‘Garnet’ |  | CGATTAATGATCACCATAGTCATATACTGTACTGTCATTTATCTATAGCTTTTTGTTGCAGCGACAGTTCTATGTACAGTGAAATGTTGCTGCTATTCCT |
|  |  |  |
|  |  | ....|....|....|....|....|....|....|....|....|....|....|....|....|....|....|....|....|....|....|....| |
| ppa009593m |  | TTTTCTTTTTTTCTGATCATATGGTTGACTTGTGTAGAGCCCCATCAGGAAGCAATCTGCTGCCTGGAAGGTGCAGGCACAGCTTAGTGAGGTAACCTGT |
| PRUAV000620 |  | ~~~~~~~~~~~~~~~~~~~~~~~~~~~~~~~~~~~~~agccccatcaggaagcaatctgctgcctggaaggtgcaggcacagcttagtgag~~~~~~~~~ |
| PavCAC_‘Regina’ |  | TTTTCCTTTTTTCTGATCATATGGTTGACTTGTGTAGAGCCCCATCAGGAAGCAATCTGCTGCCTGGAAGGTGCAGGCACAGCTTAGTGAGGTAACCTGT |
| PavCAC_‘Lapins’ |  | TTTTCCTTTTTTCTGATCATATGGTTGACTTGTGTAGAGCCCCATCAGGAAGCAATCTGCTGCCTGGAAGGTGCAGGCACAGCTTAGTGAGGTAACCTGT |
| PavCAC_‘Garnet’ |  | TTTTCCTTTTTTCTGATCATATGGTTGACTTGTGTAGAGCCCCATCAGGAAGCAATCTGCTGCCTGGAAGGTGCAGGCACAGCTTAGTGAGGTAACCTGT |
|  |  |  |
|  |  | ....|....|....|....|....|....|....|....|....|....|....|....|....|....|....|....|....|....|....|....| |
| ppa009593m |  | TTGCAATAAGTGTAGTACATGAATTTGATTGAAGAACATTGTCATCAATGCATAATAAATTTCTTTCTGAATCCAAGGTAACTGAGAAATCGTCGAATTC |
| PRUAV000620 |  | ~~~~~~~~~~~~~~~~~~~~~~~~~~~~~~~~~~~~~~~~~~~~~~~~~~~~~~~~~~~~~~~~~~~~~~~~~~~~~gtaactgagaaatcgtcgaattc |
| PavCAC_‘Regina’ |  | TTGCAATAAGTGTAGTACATGAATTTGATTGGAGAACATTGTCATCAATGCATAATAAATTTCTTTCTAAATC~AAGGTAACTGAGAAATCGTCGAATTC |
| PavCAC_‘Lapins’ |  | TTGCAATAAGTGTAGTACATGAATTTGATTGGAGAACATTGTCATCAATGCATAATAAATTTCTTTCTAAATC~AAGGTAACTGAGAAATCGTCGAATTC |
| PavCAC_‘Garnet’ |  | TTGCAATAAGTGTAGTACATGAATTTGATTGGAGAACATTGTCATCAATGCATAATAAATTTCTTTCTAAATC~AAGGTAACTGAGAAATCGTCGAATTC |
|  |  |  |
|  |  | ....|....|....|....|....|....|....|....|....|....|....|....|....|....|....|....|....|....|....|....| |
| ppa009593m |  | TGCACCCATAAATAACACCAAGTCTGAAGATGGATCACTGGAAGGGAAAGATGAGTCTACTGAAAAGCGTTCCAGCAATATTCCAGATGCATCATCAATA |
| PRUAV000620 |  | tgcacccataaataacaccaagtctgaagatggatcactggaagggaaagatgagtctactgaaaagcgttccagcaatattccagatgcatcatcaata |
| PavCAC_‘Regina’ |  | TGCACCCATAAATAACACCAAGTCTGAAGATGGATCACTGGAAGGGAAAGATGAGTCTACTGAAAAGCGTTCCAGCAATATTCCAGATGCATCATCAATA |
| PavCAC_‘Lapins’ |  | TGCACCCATAAATAACACCAAGTCTGAAGATGGATCACTGGAAGGGAAAGATGAGTCTACTGAAAAGCGTTCCAGCAATATTCCAGATGCATCATCAATA |
| PavCAC_‘Garnet’ |  | TGCACCCATAAATAACACCAAGTCTGAAGATGGATCACTGGAAGGGAAAGATGAGTCTACTGAAAAGCGTTCCAGCAATATTCCAGATGCATCATCAATA |
|  |  |  |
|  |  | ....|....|....|....|....|....|....|....|....|....|....|....|....|....|....|....|....|....|....|....| |
| ppa009593m |  | TCAGCATTCATGGCTCAAGTATCAGACCTGGTTAAGTAAGTTGCAGCGGCTTTCTTTCGTAATTTATATGAGCAACTATGTGTCAGACGTACATGTTTTT |
| PRUAV000620 |  | tcagcattcatggctcaagtatcagacctggttaa~~~~~~~~~~~~~~~~~~~~~~~~~~~~~~~~~~~~~~~~~~~~~~~~~~~~~~~~~~~~~~~~~ |
| PavCAC_‘Regina’ |  | TCAGCATTCATGGCTCAAGTATCAGACCTGGTTAAGTAAGTTGCAGCGGTTTTCTTTCGTAATTTATATGAGCAACTGTGTGTCAGACGTACATTTTTTT |
| PavCAC_‘Lapins’ |  | TCAGCATTCATGGCTCAAGTATCAGACCTGGTTAAGTAAGTTGCAGCGGTTTTCTTTCGTAATTTATATGAGCAACTGTGTGTCAGACGTACATTTTTTT |
| PavCAC_‘Garnet’ |  | TCAGCATTCATGGCTCAAGTATCAGACCTGGTTAAGTAAGTTGCAGCGGTTTTCTTTCGTAATTTATATGAGCAACTGTGTGTCAGACGTACATTTTTTT |
|  |  |  |
|  |  | ....|....|....|....|....|....|....|....|....|....|....|....|....|....|....|....|....|....|....|....| |
| ppa009593m |  | TA~~~ATTAAAATGAGGTCATTTTCCTGAGCAGGTTAGTGGATTCAAGAGATATTGTGGAGCTCCAAATGAAGCAACTAGATTTAGAGCTCGTGATAAGA |
| PRUAV000620 |  | ~~~~~~~~~~~~~~~~~~~~~~~~~~~~~~~~~gttagtggattcaagagatattgtggagctccaaatgaagcaactagatttagagctagtgataaga |
| PavCAC_‘Regina’ |  | TATTAATTAAAATGAGGTCATTTTCATGAGCAGGTTAGTGGATTCAAgagatattgtggagctccaaatgaagca~ctagatttagagctagtgata~ga |
| PavCAC_‘Lapins’ |  | TATTAATTAAAATGAGGTCATTTTCATGAGCAGGTTAGTGGATTCAAGAGATattgtg~agctccaaatgaagcaactagatttagagctagtgataaga |
| PavCAC_‘Garnet’ |  | TATTAATTAAAATGAGGTCATTTTCATGAGCAGGTTAGTGGATTCAAGAGAtattgtggagctccaaatgaagcaactagatttagagctagtgaataga |
|  |  |  |
|  |  | ....|....|....|....|....|....|....|....|....|....|....|....|....|....|....|....|....|....|....|....| |
| ppa009593m |  | AAGAAA~GAAGCCTTGGA~GAAGCCAGCACC~ACAAGCTCCTTTTGCTCCACCACCAGCACATTATCCTTATGCCATGCTACCACCTCAACAAGCAGCAG |
| PRUAV000620 |  | aagaaa~gaagccttgga~gaagccagcacc~acaagctcctatggctccactaccagcacattatccttatgccatgctaccacctcaacaagcggcag |
| PavCAC_‘Regina’ |  | aagaaa~gaagccttgga~gaagccagcac~~acaagctc~tatg~ctc~actac~agcacattatccttatgc~atgctac~ac~tcaacaagccgcag |
| PavCAC_‘Lapins’ |  | aagaaaagaagcct~~ga~gaagccagcacccacaagctcctatggctccactac~agcacattatccttatgc~atgctaccacctca~ca~gcggcag |
| PavCAC_‘Garnet’ |  | aagaaa~gaagccttggaagaagtcagcacc~aca~gctcctatggctccactaccagcacattatccttatgc~atgctaccacctca~caagc~gcag |
|  |  |  |
|  |  | ....|....|....|....|....|....|....|....|....|....|....|....|....|....|....|....|....|....|....|....| |
| ppa009593m |  | CCCCAGCAACTG~CTCCTGCTCCAGCAAGCCATCCAGCAG~CAGCAGCACCTGCATTACCCGCCCCTGCAAAGGCTAGCACATCTTCTCACCCTCCACTG |
| PRUAV000620 |  | ccccagcaacag~ctcctgctccagcaagccctccagcag~cagcagcacctgcattacccgcccctgcaaaggctagcacatcttctcaccctccactg |
| PavCAC_‘Regina’ |  | ctccagcaacaggctc~tgct~cagccagcc~tccagcaggcagcagcacctgcat~acctggctctgcaa~~gctagca~atcttc~~a~cgt~ca~tg |
| PavCAC_‘Lapins’ |  | c~ccagcaacag~ctc~tgct~cagccagccctccagcag~cagcagcaactgcat~acccgtccttgca~~ggctagccaatcttct~a~cctccaatg |
| PavCAC_‘Garnet’ |  | gcccagca~cag~ctc~tgcttcagcaagtcctc~agcag~cagcagca~ctgcat~ac~~gtca~tgca~~~gctagca~atcttc~~acccttcc~tg |
|  |  |  |
|  |  | ....|....|....|....|....|....|....|....|....|....|....|....|....|....|....|....|....|....|....|....| |
| ppa009593m |  | AAATGCCCCATGGCTGGAACCTTTTACCGTTGTCCTGCACCTGGTGAACCACCTTTTGTTAAGGCAAGTGTCTGTGTTACTTATCTTTTAAAGCTTGAAT |
| PRUAV000620 |  | aaatgtcccatggctggaaccttttaccgtagtcctgcacctggtgaaccatcttttgttaaggtag~~~~~~~~~~~~~~~~~~~~~~~~~~~~~~~~~ |
| PavCAC_‘Regina’ |  | ~~agttcc~atg~ct~gaaccttt~accgta |
| PavCAC_‘Lapins’ |  | aatgtcc~gatggcgtgaacctttaac~g~a |
| PavCAC_‘Garnet’ |  | aaggtcc~~attcctggaaccgtt~~c |
|  |  |  |
|  |  | ....|....|....|....|....|....|....|....|....|....|....|....|....|....|....|....|....|....|....|....| |
| ppa009593m |  | TCTGAGAGAGATTGCAGAAGACCATTCACAATTTTCCTCATCATACACATCATTACTTATATTAGGAGTTCTGAATGTTCAGGTAGGGGATAAAGTTCAG |
| PRUAV000620 |  | ~~~~~~~~~~~~~~~~~~~~~~~~~~~~~~~~~~~~~~~~~~~~~~~~~~~~~~~~~~~~~~~~~~~~~~~~~~~~~~~~~~gggataaagttcagaaag |
| PavCAC_‘Regina’ |  |  |
| PavCAC_‘Lapins’ |  |  |
| PavCAC_‘Garnet’ |  |  |
|  |  |  |
|  |  | ....|....|....|....|....|....|....|....|....|....|....|....|....|....|....|....|....|....|....|....| |
| ppa009593m |  | AAAGGTCAAGTCATTTGCATCATTGAAGCCATGAAGTTGATGAATGAAATTGAGGTATGTTTTTGCATTGAAATGATTTTAGCTTTTCTTTCATTTGAAA |
| PRUAV000620 |  | gtcaagtcatttgcatcattgaagccatgaagttgatgaatgaaattgag~~~~~~~~~~~~~~~~~~~~~~~~~~~~~~~~~~~~~~~~~~~~~~~~~~ |
| PavCAC_‘Regina’ |  |  |
| PavCAC_‘Lapins’ |  |  |
| PavCAC_‘Garnet’ |  |  |
|  |  |  |
|  |  | ....|....|....|....|....|....|....|....|....|....|....|....|....|....|....|....|....|....|....|....| |
| ppa009593m |  | TGTATGTTGCTGTACTCTCAATTTATGTGTCCCTCTCTCTCTTTAGGCTGATCAATCTGGGACAGTAACTGAGATACTGGCCGAAGATGCAAAGCCAGTG |
| PRUAV000620 |  | ~~~~~~~~~~~~~~~~~~~~~~~~~~~~~~~~~~~~~~~~~~gctgatcaatctgggacagtaactgagatactggcagaagatgcaaaaccagtgagtg |
| PavCAC_‘Regina’ |  |  |
| PavCAC_‘Lapins’ |  |  |
| PavCAC_‘Garnet’ |  |  |
|  |  |  |
|  |  | ....|....|....|....|....|....|....|....|....|....|....|....|....|....|....|....|....|....|....|....| |
| ppa009593m |  | AGTGTAGACACGGTAAGTAAACTCTCCCTCTTTCACGCATACATACACAGAGACACAAACTCACTACTCTCTCGTGATCTATATCTAAGTGATGTGGCCT |
| PRUAV000620 |  | tagacacg~~~~~~~~~~~~~~~~~~~~~~~~~~~~~~~~~~~~~~~~~~~~~~~~~~~~~~~~~~~~~~~~~~~~~~~~~~~~~~~~~~~~~~~~~~~~ |
| PavCAC_‘Regina’ |  |  |
| PavCAC_‘Lapins’ |  |  |
| PavCAC_‘Garnet’ |  |  |
|  |  |  |
|  |  | ....|....|....|....|....|....|....|....|....|....|....|....|....|....|....|....|....|....|....|....| |
| ppa009593m |  | CTTCCATGCAGCCTCTTTTTGTCATAGTACCATGAGCAACTT |
| PRUAV000620 |  | ~~~~~~~cctctttttgtcatcgtaccatgagcaacttggggaagaattttttgaaggattttggtgatagacactctttgaagatgttttattctcgaa |
| PavCAC_‘Regina’ |  |  |
| PavCAC_‘Lapins’ |  |  |
| PavCAC_‘Garnet’ |  |  |

***CBP***

|  |  | ....|....|....|....|....|....|....|....|....|....|....|....|....|....|....|....|....|....|....|....| |
| --- | --- | --- |
| ppa006697m | 80 | ACAGCAGCGCCTGCTCCTCCCCCTACATGACTGCTCCTTCAAGCCCCACTCGATTTGGCAACTATTTCTTCAGCGCACCCACCAGCCCCACCCGGGCCTC |
| PRUAV016855 |  | ACAGCAGCGCCTGCTCCTCCCCTTACATGACTGCTCCTTCAAGCCCCACTCGATTTGGCAACTATTTCTTCAGCGCACCCACCAGCCCCACCCGGGCCTC |
| PavCAL_‘Regina’ |  | ~~~~~~~~~~~~~~~~~~~~~~~~~~~~~~~~~~~~~~~~~~~~~~~cctgtagttgggcactatttcttcagcgcACCCACCAGCCCCACCCGGGCCTC |
| PavCAL_‘Lapins’ |  | ~~~~~~~~~~~~~~~~~~~~~~~~~~~~~~~~~~~~~~~~~~~~~~cctgacgctgtcacttaatgactgctcctcacttacatgactg~~~ctcctcct |
| PavCAL_‘Garnet’ |  | ~~~~~~~~~~~~~~~~~~~~~~~~~~~~~~~~~~~~~~~~~~~~~~~aggttgggcactattatcttcagcgcatcc~~ACCAGCCCCACTCGGGGCCTC |
|  |  |  |
|  |  | ....|....|....|....|....|....|....|....|....|....|....|....|....|....|....|....|....|....|....|....| |
| ppa006697m |  | TTCCTTCTACAGCCACTTCAACGACCTCTCCGGCGAAAACGGTCCAAGACTTTCAGCCTCTTCAATCCCTTTCAAGTGGGAAGAGAAGCCCGGAATTCCC |
| PRUAV016855 |  | TTCCTTCTACAGCCACTTCAACGACTTCTCCGGCGAAAACGGTCCAAGACTTTCAGCCTCTTCGATCCCTTTCAAGTGGGAAGAGAAGCCCGGAATTCCC |
| PavCAL_‘Regina’ |  | TTCCTTCTACAGCCACTTCAACGACTTCTCCGGCGAAAACGGTCCAAGACTTTCAGCCTCTTCGATCCCTTTCAAGTGGGAAGAGAAGCCCGGAATTCCC |
| PavCAL_‘Lapins’ |  | tctatctgaCTG~CACTTCC~CGACTTCTCCGGCGAAAACGGTCCAAGACTTTCAGCCTCTTCGATCCCTTTCAAGTGGGAAGAGAAGCCCGGAATTCCC |
| PavCAL_‘Garnet’ |  | TTCCTTCTACAGCCACTTCAACGACTTCTCCGGCGAAAACGGTCCAAGACTTTCAGCCTCTTCGATCCCTTTCAAGTGGGAAGAGAAGCCCGGAATTCCC |
|  |  |  |
|  |  | ....|....|....|....|....|....|....|....|....|....|....|....|....|....|....|....|....|....|....|....| |
| ppa006697m |  | AAGTCCAGAGCTACCATTAATGGTGATCATAGTCATCATCTTGAGGAGGATTTCGAGTTTGATTTTAGTGGGCAGTTGGAGAAGGCTTCCTTGCCGGCAG |
| PRUAV016855 |  | AAGTCCAGAGCTACCATTAATGGTGATCATAGTCATCATCTTGAGGAGGATTTTGAGTTTGATTTTAGTGGGCAGTTGGAGAAGGCTTCCTTGCCGGCAG |
| PavCAL_‘Regina’ |  | AAGTCCAGAGCTACCATTAATGGTGATCATAGTCATCATCTTGAGGAGGATTTTGAGTTTGATTTTAGTGGGCAGTTGGAGAAGGCTTCCTTGCCGGCAG |
| PavCAL_‘Lapins’ |  | AAGTCCAGAGCTACCATTAATGGTGATCATAGTCATCATCTTGAGGAGGATTTTGAGTTTGATTTTAGTGGACAGTTGGAGAAGGCTTCCTTGCCGGCAG |
| PavCAL_‘Garnet’ |  | AAGTCCAGAGCTACCATTAATGGTGATCATAGTCATCATCTTGAGGAGGATTTTGAGTTTGATTTTAGTGGACAGTTGGAGAAGGCTTCCTTGCCGGCAG |
|  |  |  |
|  |  | ....|....|....|....|....|....|....|....|....|....|....|....|....|....|....|....|....|....|....|....| |
| ppa006697m |  | ACGAGCTCTTCGACGGCGGCAAGATCAGGCCTCTGAAACCCCCTCCTCGCTTACAAGTTGGGAGCAATGGAGCTGATGAGCCAGGTCCCTCCGGTTTTTC |
| PRUAV016855 |  | ACGAGCTCTTCGACGGCGGCAAGATCAGGCCTCTGAAACCCCCTCCTCGCTTACAAGTTGGGAGCAATGGAGCTGATGAGCCAGGTCCCTCCGGTTTT~C |
| PavCAL_‘Regina’ |  | ACGAGCTCTTCGACGGCGGCAAGATCAGGCCTCTGAAACCCCCTCCTCGCTTACAAGTTGGGAGCAATGGAGCTGATGAGCCAGGTCCCTCCGGTTTTTC |
| PavCAL_‘Lapins’ |  | ACGAGCTCTTCGACGGCGGCAAGATCAGGCCTCTGAAACCCCCTCCTCGCTTACAAGTTGGGAGCAATGGAGCTGATGAGCCAGGTCCCTCCGGTTTTTC |
| PavCAL_‘Garnet’ |  | ACGAGCTCTTCGACGGCGGCAAGATCAGGCCTCTGAAACCCCCTCCTCGCTTACAAGTTGGGAGCAATGGAGCTGATGAGCCAGGTCCCTCCGGTTTTTC |
|  |  |  |
|  |  | ....|....|....|....|....|....|....|....|....|....|....|....|....|....|....|....|....|....|....|....| |
| ppa006697m |  | CCCGAGGTCACCGTCTCCAAGAGCGTCGAGACTTTCCCAAGGAAA~GAAATTGGTTCAG~GGAGTACTATCTCCGCGGCATCACCGAAAGGACCATCAGG |
| PRUAV016855 |  | TCCGAGGTCACCGTCTCCAAGAGCGTCGAGACTTTCCCAAGGAAACGAAATTGGTACCAAGGAGTACTATCCCCGAGGCATCACCGAAAG~ACCATCAGG |
| PavCAL_‘Regina’ |  | YCCGAGGTCACCGTCTCCAAGAGCGTCGAGACTTTCCCAAGGAAA~GAAATTGGT~CCAAGGAGTACTATCCCCGAGGCATCACCGAAAG~ACCATCAGG |
| PavCAL_‘Lapins’ |  | CCCGAGGTCACCGTCTCCAAGAGCGTCGAGACTTTCCCAAGGAAA~GAAATTGGT~CCAAGGAGTACTATCCCCGAGGCATCACCGAAAG~ACCATCAGG |
| PavCAL_‘Garnet’ |  | CCCGAGGTCACCGTCTCCAAGAGCGTCGAGACTTTCCCAAGGAAA~GAAATTGGT~CCAAGGAGTACTATCCCCGAGGCATCACCGAAAG~ACCATCAGG |
|  |  |  |
|  |  | ....|....|....|....|....|....|....|....|....|....|....|....|....|....|....|....|....|....|....|....| |
| ppa006697m |  | CTCATGATGATCCTTTCGCTGCCGCGATGAATGAGACCCGAAAGAACGAGTATGAATACGGAAATCAAGAAGAAAGGCGGGGAAGAGAAAGATCTCCTTC |
| PRUAV016855 |  | CTCATGATGATCCTTTCGCTGCCGCTATGAATGAGACCCGTTTGCAGTGAGTGAC |
| PavCAL_‘Regina’ |  | A |
| PavCAL_‘Lapins’ |  | A |
| PavCAL_‘Garnet’ |  | A |

***CBPb***

|  |  | ....|....|....|....|....|....|....|....|....|....|....|....|....|....|....|....|....|....|....|....| |
| --- | --- | --- |
| ppa008039m | 501 | AATGGACAACACCACAAAGAGAAGTGATCAGCACCAAAGAGGAAGAGACAGAACTCCTGCAGCCTTGTCATCCTCTGTTTCGGGCCATAGAGCAACAAGG |
| PRUAV006432 |  | ~~~~~~~~~~~~~ACAAAGAGAAGTGATCAGCACCAAAGAGGAAGAGACAGAACTCCTGCAGCCTTGTCATCCTCTGTTTCGGGCCATAGAGCAACAAGG |
| PavCALb_‘Regina’ |  | ~~~~~~~~~~~~~~~~~~~~~~~~~~~~~~~~~~~~~~~~~~~~~~~~~~~~~~~~~~~~~~~~~~~~~~~~~~~~~~~~~~~~~~gtAGCTGAGCACAT |
| PavCALb_‘Lapins’ |  | ---------------------------------------------------------------------------------------------------- |
| PavCALb_‘Garnet’ |  | ~~~~~~~~~~~~~~~~~~~~~~~~~~~~~~~~~~~~~~~~~~~~~~~~~~~~~~~~~~~~~~~~~~~~~~~~~~~~~~~ggtggggggggtgcatgaTGT |
|  |  |  |
|  |  | ....|....|....|....|....|....|....|....|....|....|....|....|....|....|....|....|....|....|....|....| |
| ppa008039m |  | TCCCTATCTCCTTTGAGGGTTTCGGAGTATCAATGGGAAGAAGAAGAAAAACAACAGCAACAGCAGCAACAACAAAACAACAGACAGTTAGCTCCAAAAG |
| PRUAV006432 |  | TCCCTATCTCCTTTGAGGGTTTCGGAGTATCAATGGGAAGAAGAAGAAAAACAACAGCAACAGCAGCAACAACAAAACAACAGACAGTTAGCTCCGAAAG |
| PavCALb_‘Regina’ |  | GTCCTATCTCCTTTGAGGGTTTCGGAGTATCAATGGGAAGAAGAAGAAAAACAACAGCAACAGCAGCAACAACAAAACAACAGACAGTTAGCTCCGAAAG |
| PavCALb_‘Lapins’ |  | ---------------------------------------------------------------------------------------------------- |
| PavCALb_‘Garnet’ |  | CTGTATTTGCCTTTGAGGGTTTCGGAGTATCAATGGGAAGAAGAAGAAAAACAACAGCAACAGCAGCAACAACAAAACAACAGACAGTTAGCTCCGAAAG |
|  |  |  |
|  |  | ....|....|....|....|....|....|....|....|....|....|....|....|....|....|....|....|....|....|....|....| |
| ppa008039m |  | CCATGTTTTCTCCCTCTGCTGCTGCTTCAGCTTCAAAGGCTTCAAAGAAATGGAAACTGAGAGACTTTCTGCTGTTTCGAAGCGCATCAGAAGGAAGAGC |
| PRUAV006432 |  | CCATGTTTTCTCCCTCTGCTGCTGCTTCAGCTTCAAAGGCTTCAAAGAAATGGAAACTGAAAGACTTTCTGCTGTTTCGAAGCGCATCAGAAGGAAGAGC |
| PavCALb_‘Regina’ |  | CCATGTTTTCTCCCTCTGCTGCTGCTTCAGCTTCAAAGGCTTCAAAGAAATGGAAACTGAAAGAYTTTCTGCTGTTTCGAAGCGCATCAGAAGGAAGAGC |
| PavCALb_‘Lapins’ |  | --------------TCTGCTGCTGCTTCAGCTTCAAAGGCTTCAAAGAAATGGAAACTGAAAGACTTTCTGCTGTTTCGAAGCGCATCAGAAGGAAGAGC |
| PavCALb_‘Garnet’ |  | CCATGTTTTCTCCCTCTGCTGCTGCTTCAGCTTCAAAGGCTTCAAAGAAATGGAAACTGAAAGACTTTCTGCTGTTTCGAAGCGCATCAGAAGGAAGAGC |
|  |  |  |
|  |  | ....|....|....|....|....|....|....|....|....|....|....|....|....|....|....|....|....|....|....|....| |
| ppa008039m |  | AACAGATAAAGATCCATTCCGAAAGTACTCAAATCTGTTCAAGAAAAACGAAGATGTCAAGAACTCCAGCTTCAGGTCCATAGACAGCCCGGCGGCCACG |
| PRUAV006432 |  | AACAGATAAAGATCCATTCCGAAAGTACTCAAATCTGTTCAAGAAAAACGAAGATGTCAAGAACTCCAGCTTCAGGTCCATAGACAGCCCGGCGGCCACG |
| PavCALb_‘Regina’ |  | AACAGATAAAGATCCATTCCGAAAGTACTCAAATCTGTTCAAGAAAAACGAAGATGTCAAGAACTCCAGCTTCAGGTCCATAGACAGCCCGGCGGCCACG |
| PavCALb_‘Lapins’ |  | AACAGATAAAGATCC------------------------------------------------------------------------------------- |
| PavCALb_‘Garnet’ |  | AACAGATAAAGATCCATTCCGAAAGTACTCAAATCTGTTCAAGAAAAACGAAGATGTCAAGAACTCCAGCTTCAGGTCCATAGACAGCCCGGCGGCCACG |
|  |  |  |
|  |  | ....|....|....|....|....|....|....|....|....|....|....|....|....|....|....|....|....|....|....|....| |
| ppa008039m |  | AGCTCAAGAAGAAGAGGGCCAGTTTCAGCTCATGAGTTGCATTACACTATGAACAAAGCAGTGTCTAATGATATGAAGAAGAAGACCTTCTTGCCTTACA |
| PRUAV006432 |  | AGCACAAGAAGAAGAGGGCCAGTTTCAGCTCATGAGTTGCATTACACTATGAACAAAGCAGTGTCTAATGATATGAAGAAGAAGACCTTCTTGCCTTACA |
| PavCALb_‘Regina’ |  | AGCACAAGAAGAAGAGGGCCAGTTTCAGCTCATGAGTTGCATTACACTATGAACAAAGCAGTGTCTAATGATATGAAGAAGAAGACCTTCTTGCCTTACA |
| PavCALb_‘Lapins’ |  | ---------------------------------------------------------------------------------------------------- |
| PavCALb_‘Garnet’ |  | AGCACAAGAAGAAGAGGGCCAGTTTCAGCTCATGAGTTGCATTACACTATGAACAAAGCAGTGTCTAATGATATGAAGAAGAAGACCTTCTTGCCTTACA |
|  |  |  |
|  |  | ....|....|....|....|....|....|....|....|....|....|....|....|....|....|....|....|....|....|....|....| |
| ppa008039m |  | AGCAAGGCATTTTGGGTCGATTGGCCTTCAATCCTGCTGTCAGTGCACTTGCCAATGGCTTTGGGTCTCTTTCACGGTCCTGAATCTGCCATAGATTCTT |
| PRUAV006432 |  | AGCAGGGCATTTTGGGTCGATTGGCCTTCAATCCTGCTGTCAGTGCACTTGCCAATGGCTTTGGGTCTCTTTCACGGTCCTGAATCTGCCATAGAATTTG |
| PavCALb_‘Regina’ |  | AGCAGGGCATTTTGGGTCGATTGGCCTTCAATCCTGCTGTCAGTGCACTTGCCAATGGCTTTGGGTCTCTTTCACGGTCCTGAATCTGCCATAGAATTTG |
| PavCALb_‘Lapins’ |  | ---------------------------------------------------------------------------------------------------- |
| PavCALb_‘Garnet’ |  | AGCAGGGCATTTTGGGTCGATTGGCCTTCAATCCTGCTGTCAGTGCACTTGCCAATGGCTTTGGGTCTCTTTCACGGTCCTGAATCTGCCATAGAATTTG |
|  |  |  |
|  |  | ....|....|....|....|....|....|....|....|....|....|....|....|....|....|....|....|....|....|....|....| |
| ppa008039m |  | TATTAATTTTTGAGAAAATTTGCTGTTTTTCATCAATTTTTTTCTGCTGTAAAGTTGCCTATTTATTTGTTTTTAGCAGTGTAGAATACATACGTAAGAG |
| PRUAV006432 |  | ATTAATTTTTGAGAAAATCTGCTGTTTTTCATCAAATTTTTTCTGCTGTAAAGTTGCCTATTTATTTGTTTTTAGCTAGTGTAGAATACATACGTAAGAG |
| PavCALb_‘Regina’ |  | ATTAATTTTTGAGAAAATCTGCTGTTTTTCATCAAATTTTTTCTGCTGTAAAGTTGCCTATTTATTTGTTTTTAGCAGTGTAGAATACATACGTAAGAGG |
| PavCALb_‘Lapins’ |  | ---------------------------------------------------------------------------------------------------- |
| PavCALb_‘Garnet’ |  | ATTAATTTTTGAGAAAATCTGCTGTTTTTCATCAAATTTTTTCTGCTGTAAAGTTGCCTATTTATTTGTTTTTAGCAGTGTAGAATACATACGTAAGAGG |
|  |  |  |
|  |  | ....|....|....|....|....|....|....|....|....|....|....|....|....|....|....|....|....|....|....|....| |
| ppa008039m |  | GATTGTGGTTGATGGTTTGCAGTTAAAAACACAAAAATCAAGCATGATTATTGCCCCCATTACTGCATACACATTCTTTCTTTCTTTCTTTCTTTGTTTT |
| PRUAV006432 |  | GATTGTGGTTGATGGTTTGCAGTTAAAAACACAAAAATCAGGCATGATTATTGCCCCCATTACTGCATACACATTTCTTTCTTTCTTTCTTTGTTTTATA |
| PavCALb_‘Regina’ |  | ATTGTGGTTGTGGTTTTTGCAGTa |
| PavCALb_‘Lapins’ |  | ---------------------------------------------------------------------------------------------------- |
| PavCALb_‘Garnet’ |  | ATTGTGGTTGATGGTTTGCAGT |

***CBPc***

|  |  | ....|....|....|....|....|....|....|....|....|....|....|....|....|....|....|....|....|....|....|....| |
| --- | --- | --- |
| ppa001109m | 2005 | CAAAATGTTGCAACCAGTAACGCTGTCTTGTCACAGGAACTCACTGCAATGGTCGCTGGGAATCAAATGGAAGAGACAGAGCAAGCTGATGACTCCAAAT |
| PRUAV000501 |  | CAAAATGTTGCAACCAGTGACGCTGTCTTGTCACAGGAACTCACTGCAATGGTTGCTGGGAATCAAATGGAAGAGACAGAGCAAGCTGATGACTCCAAAT |
| PavCALc_‘Regina’ |  | ~~~~~~~~~~~~~~~~~~~~~~~~~~~~~~~~~~~~~~~~~~~~~~~~~~~~~~~~~~~~~~~~~~~~~~~~~~~~~~~~~~ttccaaacttaaactccA |
| PavCALc_‘Lapins’ |  | ~~~~~~~~~~~~~~~~~~~~~~~~~~~~~~~~~~~~~~~~~~~~~~~~~~~~~~~~~~~~~~~~~~~~~~~~~~~~~~~~~cccgcaaactatactccaa |
| PavCALc_‘Garnet’ |  | ~~~~~~~~~~~~~~~~~~~~~~~~~~~~~~~~~~~~~~~~~~~~~~~~~~~~~~~~~~~~~~~~~~~~~~~~~~~~~~~~~~~~tcaatcttaaCTGAAT |
|  |  |  |
|  |  | ....|....|....|....|....|....|....|....|....|....|....|....|....|....|....|....|....|....|....|....| |
| ppa001109m |  | CATCAGAACAAATCCAGCTTTCTGATGAAGATGCTTTCAAGATTGAAGATCATGAAAACTGCAAGAAGACTGAACCCTTCCAGCTTAATGACAGTGCTGA |
| PRUAV000501 |  | CATCAGAACAAATCCAGCTTTCTGATGAAGATGCTTTCAAGATTGAAGATCATGAAAACTGCAAGAAGACTGAACCCTTCCAGCTTAATGATAGTGCTGA |
| PavCALc_‘Regina’ |  | ATCTCAGAACAATCCAGCTTTCTGATGAAGATGCTTTCAAGATTGAAGATCATGAAAACTGCAAGAAGACTGAACCCTTCCAGCTTAATGATAGTGCTGA |
| PavCALc_‘Lapins’ |  | TCATCAGAACAATCCAGCTTTCTGATGAAGATACTTTCAAGATTGAAGATCATGAAAACTGCAAGAAGACTGAACCCTTCCAGCTTAATGATAGTGCTGA |
| PavCALc_‘Garnet’ |  | CATCAGAACAAATCCAGCTTTCTGATGAAGATGCTTTCAAGATTGAAGATCATGAAAACTGCAAGAAGACTGAACCCTTCCAGCTTAATGATAGTGCTGA |
|  |  |  |
|  |  | ....|....|....|....|....|....|....|....|....|....|....|....|....|....|....|....|....|....|....|....| |
| ppa001109m |  | AGTTGGCAACCTGTCTGGCGGGAAGTACAAGAAGCCAAAAATTTCAACTTCCATTGAATCTAAGGATCAGGGTGATTTAAGGCTGAACAACAGATCAGGT |
| PRUAV000501 |  | AGTTGGCAACCAGTCTGGAGGGAAGTACAAGAAGCCAAAAATTTCAACTTCCATTGAATCTGAGGATCAGGGTGATTTAAGGCTGAACAACAGATCAGGT |
| PavCALc_‘Regina’ |  | AGTTGGCAACCAGTCTGGAGGGAAGTACAAGAAGCCAAAAATTTCAACTTCCATTGAATCTGAGGATCAGGGTGATTTAAGGCTGAACAACAGATCAGGT |
| PavCALc_‘Lapins’ |  | AGTTGGCAACCAGTCTGGAGGGAAGTACAAGAAGCCAAAAATTTCAACTTCCATTGAATCTGAGGATCAGGGTGATTTAAGGCTGAACAACAGATCAGGT |
| PavCALc_‘Garnet’ |  | AGTTGGCAACCAGTCTGGAGGGAAGTACAAGAAGCCAAAAATTTCAACTTCCATTGAATCTGAGGATCAGGGTGATTTAAGGCTGAACAACAGATCAGGT |
|  |  |  |
|  |  | ....|....|....|....|....|....|....|....|....|....|....|....|....|....|....|....|....|....|....|....| |
| ppa001109m |  | CTCTCAGAAAACAGCACAGGAGAATCACATAATATGGAAATGGAAAACAATTCAGAGCCAGATGCAACAGAAACTTTCATGGCCAATAATAGCATTAGCC |
| PRUAV000501 |  | CCCTCAGAAAACAGCACAGGAGAACCTCACGATATGGAAGTGGAAAACAATTCAGAGCCAGATGCAACAGAAACTTTCATGGCCAATAATGGTATTAGCC |
| PavCALc_‘Regina’ |  | CCCTCAGAAAACAGCACAGGAGAACCTCACGATATGGAAGTGGAAAACAATTCAGAGCCAGATGCAACAGAAACTTTCATGGCCAATAATGGTATTAGCC |
| PavCALc_‘Lapins’ |  | CCCTCAGAAAACAGCACAGGAGAACCTCACAATATAGAAGTGGAAAACAATTCAGAGCCAGATGCAACAGAAACTTTCATGGCCAATAATGGTATTAGCC |
| PavCALc_‘Garnet’ |  | CCCTCAGAAAACAGCACAGGAGAACCTCACGATATGGAAGTGGAAAACAATTCAGAGCCAGATGCAACAGAAACTTTCATGGCCAATAATGGTATTAGCC |
|  |  |  |
|  |  | ....|....|....|....|....|....|....|....|....|....|....|....|....|....|....|....|....|....|....|....| |
| ppa001109m |  | CTGGGCTGAAGAGAAAATTTTCCCATGGAGAAAGCAATTCCAAGCAAGAACTTCCCGACGCCTGCAACTACCGAAGAGGAAGCAAATTCAAGAGACTTAG |
| PRUAV000501 |  | CTGGGCTGAAGAGAAAATTTTCCCATGGAGAAAGCAATTCCAACCAAGAACTTCCTGACACCTGCAACTACCGAAGAGGAAGCAAATTCAAGAGACTTAG |
| PavCALc_‘Regina’ |  | CTGGGCTGAAGAGAAAATTTTCCCATGGAGAAAGCAATTCCAACCAAGAACTTCCYGACACCTGCAACTACCGAAGAGGAAGCAAATTCAAGAGACTTAG |
| PavCALc_‘Lapins’ |  | CTGGGCTGAAGAGAAAATTTTCCCATGGAGAAAGCAATTCCAACCAAGAACTTCCCGACACCTGCAACTACCGAAGAGGAAGCAAATTCAAGAGACTTAG |
| PavCALc_‘Garnet’ |  | CTGGGCTGAAGAGAAAATTTTCCCATGGAGAAAGCAATTCCAACCAAGAACTTCCTGACACCTGCAACTACCGAAGAGGAAGCAAATTCAAGAGACTTAG |
|  |  |  |
|  |  | ....|....|....|....|....|....|....|....|....|....|....|....|....|....|....|....|....|....|....|....| |
| ppa001109m |  | CGTGGATGAGGAGGAACAAAGGAAGTACAATCCAAGAGAACCAAATTATCTGCCTGTGGTTCCTGATCCTGAAGCAGAAAAGGTTGACCTCAGGCATCAG |
| PRUAV000501 |  | CGTGGAGGAGGA~~~ACAAAGGAAGTACAATCCAAGAGAACCAAATTATCTGCCTGTGGTTCCTGATCCTGAAGCAGAAAAGGTTGACCTCAGGCATCAG |
| PavCALc_‘Regina’ |  | CGTGGAGGAGGA~~~ACAAAGGAAGTACAATCCAAGAGAACCAAATTATCTGCCTGTGGTTCCTGATCCTGAAGCAGAAAAGGTTGACCTCAGGCATCAG |
| PavCALc_‘Lapins’ |  | CGTGGAGGAGGA~~~ACAAAGGAAGTACAATCCAAGAGAACCAAATTATCTGCCTGTGGTTCCTGATCCTGAAGCAGAAAAGGTTGACCTCAGGCATCAG |
| PavCALc_‘Garnet’ |  | CGTGGAGGAGGA~~~ACAAAGGAAGTACAATCCAAGAGAACCAAATTATCTGCCTGTGGTTCCTGATCCTGAAGCAGAAAAGGTTGACCTCAGGCATCAG |
|  |  |  |
|  |  | ....|....|....|....|....|....|....|....|....|....|....|....|....|....|....|....|....|....|....|....| |
| ppa001109m |  | ATGATGGACGAAAAGAAAAATGCAGAGGAATGGATGCTTGACTTTGCACTCCAACAGGCCGTAACAAAACTTGCTCCAGCGAGGAAGAAGAAAGTGGCAT |
| PRUAV000501 |  | ATGATGGACGAAAAGAAAAATGCAGAGGAGTGGATGCTTGACTTTGCACTCCAACAGGCTGTAACAAAACTTGCTCCAGCGAGGAAGAAGAAAGTGGCAT |
| PavCALc_‘Regina’ |  | ATGATGGACGAAAAGAAAAATGCAGAGGAGTGGATGCTTGACTTTGCACTCCAACAGGCTGTAACAAAACTTGCTCCAGCGAGGAAGAAGAAAGTGGCAT |
| PavCALc_‘Lapins’ |  | ATGATGGACGAAAAGAAAAATGCAGAGGAGTGGATGCTTGACTTTGCACTCCAACAGGCTGTAACAAAACTTGCTCCAGCGAGGAAGAAGAAAGTGGCAT |
| PavCALc_‘Garnet’ |  | ATGATGGACGAAAAGAAAAATGCAGAGGAGTGGATGCTTGACTTTGCACTCCAACAGGCTGTAACAAAACTTGCTCCAGCGAGGAAGAAGAAAGTGGCAT |
|  |  |  |
|  |  | ....|....|....|....|....|....|....|....|....|....|....|....|....|....|....|....|....|....|....|....| |
| ppa001109m |  | TGCTTGTTGAAGCTTTCGAAGCAGTCATGCCAGTGCCCAAATGTGAAACAAGTCGCAGGCATACTTCAGCAGCCTTCAGTCAGGCAAGGCCTATGCAAGC |
| PRUAV000501 |  | TGCTTGTTGAAGCTTTCGAAGCGGTCATGCCAGTGCCCAAATGTGAAACAAGTCGCAGGCATACTTCAGCAGCCTTCAATCAGGCAAGGCCTATGCAAGC |
| PavCALc_‘Regina’ |  | TGCTTGTTGAAGCTTTCGAAGCGGTCATGCCAGTGCCCAAATGTGAAACAAGTCGCAGGCATACTTCAGCAGCCTTCAATCAGGCAAGGCCTATGCAAGC |
| PavCALc_‘Lapins’ |  | TGCTTGTTGAAGCTTTCGAAGCGGTCATGCCAGTGCCCAAATGTGAAACAAGTCGCAGGCATACTTCAGCAGCCTTCAGTCAGGCAAGGCCTGTGCAAGC |
| PavCALc_‘Garnet’ |  | TGCTTGTTGAAGCTTTCGAAGCGGTCATGCCAGTGCCCAAATGTGAAACAAGTCGCAGGCATACTTCAGCAGCCTTCAATCAGGCAAGGCCTATGCAAGC |
|  |  |  |
|  |  | ....|....|....|....|....|....|....|....|....|....|....|....|....|....|....|....|....|....|....|....| |
| ppa001109m |  | TTGTAGCTGATGGCAGTTAAGTTACTGAGGTTAATATATAATCACAAACTCCAATACCTTCTGATATTTGATTCTTATAGCTATTCATGACTTCATATTG |
| PRUAV000501 |  | TTGTAGCTGATGGCAGTTA~~~~~CTGAGGTTAAAAGACTGCAATATATGCATAATAAGTCACTGAATTAGGAGGTATCTATACAGCCGGCAACACAAGA |
| PavCALc_‘Regina’ |  | TTGTAGCTGATGGCAGTTA~~~~~CTGAGGTTAATATATAATCACAAACTCCAAGACCTTCTGATATTTGATTCTTATAGCTATTCATGACTTCATATTG |
| PavCALc_‘Lapins’ |  | TTGTAGCTGATGGCAGTTA~~~~~CTGAGGTTAATATATAATCACAAACTCCAAGACCTTCTGATATTTGATTCTTATAGCTATTCATGACTTCATATTG |
| PavCALc_‘Garnet’ |  | TTGTAGCTGATGGCAGTTA~~~~~CTGAGGTTAATATATAATCACAAACTCCAAGACCTTCTGATATTTGATTCTTATAGCTATTCATGACTTCATATTG |
|  |  |  |
|  |  | ....|....|....|....|....|....|....|....|....|....|....|....|....|....|....|....|....|....|....|....| |
| ppa001109m |  | TTGGTTTCTAATAAGAAAGGGAATTAATTATCATATTGTTATGCAGGTGAAAAGACTGCAATATATGCATAATAAGTCACTGAATTAGGAGGTATCGATA |
| PRUAV000501 |  | GAAACTACGGGACCAGCAGAACAACTGACCATTTCTCGGCTCCAGTCTGGATTTCTGGAGAGGAAGGAAGCAGCTTCTTGCAGTGATGATTTGAATGGGG |
| PavCALc_‘Regina’ |  | TTGGTTTCTAATAAGAAAGGGAATTAATTCTCATATTGTTATGCAGTTGAAAAGACTGCAATATATACATAATAAGTCACTGAATTAGGAGGCATCTATG |
| PavCALc_‘Lapins’ |  | TTGGTTTCTAATAAGAAAGGGAATTAATTCTCATATTGTTATGCAGGTGAAAAGACTGCAATATATACATAATAAGTCACTGAATTAGGAGGCATCTATG |
| PavCALc_‘Garnet’ |  | TTGGTTTCTAAAAAGAAAGGGAATTAATTCTCATATTGTTATGCAGGTTAAAAGACTGCAATATATGCATAATAAGTCACTGAATTAGGAGGTATCTATA |
|  |  |  |
|  |  | ....|....|....|....|....|....|....|....|....|....|....|....|....|....|....|....|....|....|....|....| |
| ppa001109m |  | CAGCCAGCCGGACAAGAGAAACTACGGGACCAGCAGAACAACTGACCTTTTCTCGGCTCCAGTCTGGTTTTCTGGAGAGAAAGGAAGCAGCTTCATGCAG |
| PRUAV000501 |  | TTAAACTGTGGACACAGTAATGTTGTTCTCCTAAGCTCAGTGGATGTACTACTAATGTACCTTCGTTAGCTAATATTTGTAATGACTAGTTTTAATACGT |
| PavCALc_‘Regina’ |  | CAGCCAGTCAGGACGAGAGACTATacgacacccagaacaactgagaacttcttgccgctactctggatatctgggagaaaaaaagaagaaaaa |
| PavCALc_‘Lapins’ |  | CAGCCAGTCGGACGAGAAACTACGCGACCAGCAGAACAACTGACCATTTCTTGTCTCCAGTCTGGATTTTCTGGAGAGGAAAGGAAGCAA |
| PavCALc_‘Garnet’ |  | CAGCCGGCAACACAAGAGAAACTACGGGACCAGCAGAACAACTGACCATTTCTCGGCTCCAGTCTGGATTTCTGGAGAGGGAAAggaagcaaa |

***CBPd***

|  |  | *....|....|....|....|....|....|....|....|....|....|....|....|....|....|....|....|....|....|....|....|* |  |
| --- | --- | --- | --- |
| ppa012091m |  | ~~~~~~~~~~~~~~~~~~~~~~~~~~~~~~~~~~~~~~~~~~~~~~~~~~~~~~~~~~~~~~~~~~~~~~~~~~~~~~~~~~~~~~~~~~~~~~~~~~~~ |  |
| PRUAV002776 | 251 | TTGCACCCTACTCTTCAACAGACGGTGCAAGGCCCCTCCCTCTTCTCTCTCACCTTATCTTATCTTTTACTGTGATTGCCGACGAAAGAAAATCGTAGCT |  |
| PavCALd_‘Regina’ |  | ~~~~~~~~~~~~~~~~~~~~~~~~~~~~~~~~~~~~~~~~~~~tcCCCTT~~~~~~ATCTTATCTTTTACTGTGATTGCCGACGAAAGAAAATCGTAGCT |  |
| PavCALd_‘Lapins’ |  | ~~~~~~~~~~~~~~~~~~~~~~~~~~~~~~~~~aggccccccttcctcttaaAAATATCTTATCTTTTACTGTGATTGCCGACGAAAGAAAATCGTAGCT |  |
| PavCALd_‘Garnet’ |  | ~~~~~~~~~~~~~~~~~~~~~~~~~~~~~~~~~~~~~~~~~~~~~~~~~~~~~~~~~~~~taCCTTT~~GT~TGATTGCCGACGAAAGAAAATCGTAGCT |  |
|  |  |  |  |
|  |  | ....|....|....|....|....|....|....|....|....|....|....|....|....|....|....|....|....|....|....|....| |  |
| ppa012091m |  | ~~~~~~~~~~~~~~~~~~~~~~~~~~~~~~~~~~~~~~~~~~~~~~~~~~~~~~~~~~~~~~~~~~~~~~~~~~~~~~~~~~~~~~~~~~~~~~~~~~~~ |  |
| PRUAV002776 |  | TCGCTGCTACAGAGTCTCTTGGGAAACCACTTTTGAGTCTGTGTTTTGATTTTTATTGCTCTTTATTCCGTCTGTCTCTTTCTGATTGAAACTTTTATCC |  |
| PavCALd_‘Regina’ |  | TCGCTGCTACAGAGTCTCTTGGGAAACCACTTTTGAGTCTGTGTTTTGATTTTTATTGCTCTTTATTCCGTCTGTCTCTTTCTGATTGAAACTTTTATCC |  |
| PavCALd_‘Lapins’ |  | TCGCTGCTACAGAGTCTCTTGGGAAACCACTTTTGAGTCTGTGTTTTGATTTTTATTGCTCTTTATTCCGTCTGTCTCTTTCTGATTGAAACTTTTATCC |  |
| PavCALd_‘Garnet’ |  | TCGCTGCTACAGAGTCTCTTGGGAAACCACTTTTGAGTCTGTGTTTTGATTTTTATTGCTCTTTATTCCGTCTGTCTCTTTCTGATTGAAACTTTTATCC |  |
|  |  |  |  |
|  |  | ....|....|....|....|....|....|....|....|....|....|....|....|....|....|....|....|....|....|....|....| |  |
| ppa012091m | 1 | ~~~~~~~~~~~~~~~~~~~~~~~~~~~~~~~~~~~~~~~~TGTTAAACTTTCTTCTCTCT~~CTCTTTTTCTTCCCCATTTCTGATTTCAATTCTCAGAC |  |
| PRUAV002776 |  | ACCATTTCACATGCTTTTGTTTCTCTTTTTTTCATCACGCTGTTAAACTCTCTTCTCTCTTTCTCTTTTTCTTCCTCATTTCTGATTTCAATTCTCAGAC |  |
| PavCALd_‘Regina’ |  | ACCATTTCACATGCTTTTGTTTCTCTTTTTTTCATCACGCTGTTAAACTCTCTTCTCTCTTTCTCTTTTTCTTCCTCATTTCTGATTTCAATTCTCAGAC |  |
| PavCALd_‘Lapins’ |  | ACCATTTCACATGCTTTTGTTTCTCTTTTTTTCATCACGCTGTTAAACTCTCTTCTCTCTTTCTCTTTTTCTTCCTCATTTCTGATTTCAATTCTCAGAC |  |
| PavCALd_‘Garnet’ |  | ACCATTTCACATGCTTTTGTTTCTCTTTTTTTCATCACGCTGTTAAACTCTCTTCTCTCTTTCTCTTTTTCTTCCTCATTTCTGATTTCAATTCTCAGAC |  |
|  |  |  |  |
|  |  | ....|....|....|....|....|....|....|....|....|....|....|....|....|....|....|....|....|....|....|....| |  |
| ppa012091m |  | TTTGTTTGATCTCAATCTCACTTGGGTATTTCTTATTTTGCTATTTTCTTAAATCT~AAGTCTTTTCTTATTTATTTTTGTTTTTACAATTCTGGGTTAC |  |
| PRUAV002776 |  | TCTGTTTGATCTCAATCTCACTTGGGTATTTCTTATTTTGCTATTTTCTTAAATCTCAAGTCTTTTCTTATTTATTTTTGTTTTTACAATACTGGGTTAC |  |
| PavCALd_‘Regina’ |  | TCTGTTTGATCTCAATCTCACTTGGGTATTTCTTATTTTGCTATTTTCTTAAATCTCAAGTCTTTTCTTATTTATTTTTGTTTTTACAATACTGGGTTAC |  |
| PavCALd_‘Lapins’ |  | TCTGTTTGATCTCAATCTCACTTGGGTATTTCTTATTTTGCTATTTTCTTAAATCTCAAGTCTTTTCTTATTTATTTTTGTTTTTACAATACTGGGTTAC |  |
| PavCALd_‘Garnet’ |  | TCTGTTTGATCTCAATCTCACTTGGGTATTTCTTATTTTGCTATTTTCTTAAATCTCAAGTCTTTTCTTATTTATTTTTGTTTTTACAATACTGGGTTAC |  |
|  |  |  |  |
|  |  | ....|....|....|....|....|....|....|....|....|....|....|....|....|....|....|....|....|....|....|....| |  |
| ppa012091m |  | TTTGATTTCAGCTTATCTTTCATTTTTCATTTCTGGGCAATCTGAGATCTCAGTTTTTGTCACCTGGGTTGTGCTGATTTCGATCCAATTTGCTTACTTT |  |
| PRUAV002776 |  | TTTGATTTCAACTTATCTTTCATTTTTCATTTCTGGGCAATCTGAGATCTCAGTTTTTGTCAC~TGGGTTGTGCTGATTTCGATCCAATTTGCTTACTTT |  |
| PavCALd_‘Regina’ |  | TTTGATTTCAACTTATCTTTCATTTTTCATTTCTGGGCAATCTGAGATCTCAGTTTTTGTCAC~TGGGTTGTGCTGATTTCGATCCAATTTGCTTACTTT |  |
| PavCALd_‘Lapins’ |  | TTTGATTTCAACTTATCTTTCATTTTTCATTTCTGGGCAATCTGAGATCTCAGTTTTTGTCAC~TGGGTTGTGCTGATTTCGATCCAATTTGCTTACTTT |  |
| PavCALd_‘Garnet’ |  | TTTGATTTCAACTTATCTTTCATTTTTCATTTCTGGGCAATCTGAGATCTCAGTTTTTGTCAC~TGGGTTGTGCTGATTTCGATCCAATTTGCTTACTTT |  |
|  |  |  |  |
|  |  | ....|....|....|....|....|....|....|....|....|....|....|....|....|....|....|....|....|....|....|....| |  |
| ppa012091m |  | CCCTCGAATTTCAATTCGAAGGCAATGTCACAAGGCTATGCGATCGAGCTCTACTTCGATCCAGCCCTCGAAAACCAGGTCTTGAAGGCTTGGAATGTGC |  |
| PRUAV002776 |  | CCCTCGAATTTCAATTCGAAGGCAATGTCACAAGGCTATGCGATCGAGCTCTACTTCGATCCAGCCCTCGAAAACCAGGTCTTGAAGGCTTGGAATGTGC |  |
| PavCALd_‘Regina’ |  | CCCTCGAATTTCAATTCGAAGGCAATGTCACAAGGCTATGCGATCGAGCTCTACTTCGATCCAGCCCTCGAAAACCAGGTCTTGAAGGCTTGGAATGTGC |  |
| PavCALd_‘Lapins’ |  | CCCTCGAATTTCAATTCGAAGGCAATGTCACAAGGCTATGCGATCGAGCTCTACTTCGATCCAGCCCTCGAAAACCAGGTCTTGAAGGCTTGGAATGTGC |  |
| PavCALd_‘Garnet’ |  | CCCTCGAATTTCAATTCGAAGGCAATGTCACAAGGCTATGCGATCGAGCTCTACTTCGATCCAGCCCTCGAAAACCAGGTCTTGAAGGCTTGGAATGTGC |  |
|  |  |  |  |
|  |  | ....|....|....|....|....|....|....|....|....|....|....|....|....|....|....|....|....|....|....|....| |  |
| ppa012091m |  | TCGCTCGCCGTCAGATTAGTACCCAACTCATTGAAATCGAATCACGCCCCCACATCACCCTCTTCTCCAGCCCCTTCATTGAACCAGCGAAGCTCGAAAA |  |
| PRUAV002776 |  | TCGCTCGCCGTCAGATTAGTACCCAACTCATTGAAATCGAATCACGCCCCCACATCACCCTCTTCTCCAGCCCTTTCATTGAACCCGCGAAGCTCGAAAA |  |
| PavCALd_‘Regina’ |  | TCGCTCGCCGTCAGATTAGTACCCAACTCATTGAAATCGAATCACGCCCCCACATCACCCTCTTCTCCAGCCCTTTCATTGAACCCGCGAAGCTCGAAAA |  |
| PavCALd_‘Lapins’ |  | TCGCTCGCCGTCAGATTAGTACCCAACTCATTGAAATCGAATCACGCCCCCACATCACCCTCTTCTCCAGCCCTTTCATTGAACCCGCGAAGCTCGAAAA |  |
| PavCALd_‘Garnet’ |  | TCGCTCGCCGTCAGATTAGTACCCAACTCATTGAAATCGAATCACGCCCCCACATCACCCTCTTCTCCAGCCCTTTCATTGAACCCGCGAAGCTCGAAAA |  |
|  |  |  |  |
|  |  | ....|....|....|....|....|....|....|....|....|....|....|....|....|....|....|....|....|....|....|....| |  |
| ppa012091m |  | CGTAATCAAAGCCTTCGCTTCGAAGCAAGAACCTTTGGCGTTGTCCTTCTCCTCAATTGGGAGCCTTCCACATGACAACAATGTCCTGTTTCTGTCACCA |  |
| PRUAV002776 |  | CGTAATCAAAGCCTTCGCTTCGAAGCAAGAACCTTTGGCGTTGTCTTTCTCCTCAATTGGGAGCCTTCCACATGACAACAATGTCCTGTTTCTGTCACCA |  |
| PavCALd_‘Regina’ |  | CGTAATCAAAGCCTTCGCTTCGAAGCAAGAACCTTTGGCGTTTTTCTTTCTCCa |  |
| PavCALd_‘Lapins’ |  | CGTAATCAAAGCCTTCGCTTCGAAGCAAGAACCTTTGGCGTTGTCCTTTCTCC |  |
| PavCALd_‘Garnet’ |  | CGTAATCAAAGCCTTCGCTTCGAAGCAAGAACCTTTGGCGTTTCTCTTTCTCCa |  |

***CBPe***

|  |  | ....|....|....|....|....|....|....|....|....|....|....|....|....|....|....|....|....|....|....|....| |  |
| --- | --- | --- | --- |
| ppa004675m | 501 | GTTGGTCTTGTGGGTGAGATTTCTTTTACAGATAATTCAAGCTGGACGAGGAGCCGTAGGTTCAGGCTAGGAGCAAGAGTTTTAGATAAATTTGATGGAA |  |
| PRUAV024877 |  | ~~~~~~~~~~~~~~~~~~~~~~~~~~~~~~~~~~~~~~~~~~~~~~~AAGGAGCCGTAGGTTCAGGCTAGGAGCAAGAGTTTTAGATAAATTTGATGGAA |  |
| PavCALe_‘Regina’ |  | ~~~~~~~~~~~~~~~~~~~~~~~~~~~~~~~~~~~~~~~~~~~~~~~~~~~~~~~~~~~~~~~~~~~~~~~~~~~~~~~~~~~~~cggaatttgatgga~ |  |
| PavCALe_‘Lapins’ |  | ~~~~~~~~~~~~~~~~~~~~~~~~~~~~~~~~~~~~~~~~~~~~~~~~~~~~~~~~~~~~~~~~~~~~~~~~~~~~~~~~~~gggaaattttggatggtt |  |
| PavCALe_‘Garnet’ |  | ~~~~~~~~~~~~~~~~~~~~~~~~~~~~~~~~~~~~~~~~~~~~~~~~~~~~~~~~~~~~~~~~~~~~~~~~~~~~~~~~~~~~cgaaatttgcaaggtt |  |
|  |  |  |  |
|  |  | ....|....|....|....|....|....|....|....|....|....|....|....|....|....|....|....|....|....|....|....| |  |
| ppa004675m |  | CTAGAATAAAAGAAGCAAAGACAGAATCATTTATTGTCAGGGATCACCGTGGAGAATGTGAGAATAATTCATTTTTTGCTCACTCGATACTTCTATTTGA |  |
| PRUAV024877 |  | CTAGAATAAAAGAAGCAAAGACAGAATCATTTATTGTCAGGGATCACCGTGGAGAAT~~~~~~~~~~~~~~~~~~~~~~~~~~~~~~~~~~~~~~~~~~~ |  |
| PavCALe_‘Regina’ |  | CTAGA~TAAA~GA~GCAAAGACAGAATCATTTATTGTCAGGGATCACCGTGGAGAATGTGAGAATCATTCATTTTTTGCTCACTCGATACTTCTATTTGA |  |
| PavCALe_‘Lapins’ |  | c~AGA~TAAAAGA~GCAA~GACAGAATCATTTATTGTCAGGGATCACCGTGGAGAATGTGAGAATCATTCATTTTTTGCTCACTCGATACTTCTATTTGA |  |
| PavCALe_‘Garnet’ |  | cTAGAATAAAAGA~GCAAAGACAGAATCATTTATTGTCAGGGATCACCGTGGAGAATGTGAGAATCATTCATTTTTTGCTCACTCGATACTTCTATTTGA |  |
|  |  |  |  |
|  |  | ....|....|....|....|....|....|....|....|....|....|....|....|....|....|....|....|....|....|....|....| |  |
| ppa004675m |  | TGTAAATAACTCATTACATTTTCATGCATGGCTCATGGCCTTCAAATTTAGGATCAGCTGACCCAAGTAAGCATACATTATTATATAACCTGAGCTGCTT |  |
| PRUAV024877 |  | ~~~~~~~~~~~~~~~~~~~~~~~~~~~~~~~~~~~~~~~~~~~~~~~~~~~~~~~~~~~~~~~~~~~~~~~~~~~~~~~~~~~~~~~~~~~~~~~~~~~~ |  |
| PavCALe_‘Regina’ |  | TGTAAATAACTCGTTACATTTTCATGCATGGCTCATGGCCTTCAAATTTAGGATTAGCTAACCCAAGTAAACATACATTATTATATAACCTGAGCTACTT |  |
| PavCALe_‘Lapins’ |  | TGTAAATAACTCGTTACATTTTCATGCATGGCTCATGGCCTTCAAATTTAGGATTAGCTAACCCAAGTAAACATACATTATTATATAACCTGAGCTACTT |  |
| PavCALe_‘Garnet’ |  | TGTAAATAACTCGTTACATTTTCATGCATGGCTCATGGCCTTCAAATTTAGGATTAGCTAACCCAAGTAAACATACATTATTATATAACCTGAGCTACTT |  |
|  |  |  |  |
|  |  | ....|....|....|....|....|....|....|....|....|....|....|....|....|....|....|....|....|....|....|....| |  |
| ppa004675m |  | TTGGTTCTGCAGTGTACAAGAAACACCACCCACCATCTCTGCTTGATGAGGTGTGGAGACTAGAAAAGATTGGCAAGGACGGAGCTTTCCATAAGCGTTT |  |
| PRUAV024877 |  | ~~~~~~~~~~~~TGTACAAGAAACACCACCCACCATCTCTTCTTGATGAAGTGTGGAGACTAGAAAAGATTGGCAAGGACGGAGCTTTCCATAAGCGTTT |  |
| PavCALe_‘Regina’ |  | TTGGTTCTGCAGTGTACAAGAAACACCACCCACCATCTCTTCTTGATGAAGTGTGGAGACTAGAAAAGATTGGCAAGGACGGAGCTTTCCATAAGCGTTT |  |
| PavCALe_‘Lapins’ |  | TTGGTTCTGCAGTGTACAAGAAACACCACCCACCATCTCTTCTTGATGAAGTGTGGAGACTAGAAAAGATTGGCAAGGACGGAGCTTTCCATAAGCGTTT |  |
| PavCALe_‘Garnet’ |  | TTGGTTCTGCAGTGTACAAGAAACACCACCCACCATCTCTTCTTGATGAAGTGTGGAGACTAGAAAAGATTGGCAAGGACGGAGCTTTCCATAAGCGTTT |  |
|  |  |  |  |
|  |  | ....|....|....|....|....|....|....|....|....|....|....|....|....|....|....|....|....|....|....|....| |  |
| ppa004675m |  | GAGTCGGGAAAACATCAACACTGTGAAGGATTTTCTCACCCTACTCTTCATAGACTCTTCAAGGCTTCGGAATGTAATTATTGACTTTTCTTATCTCGGT |  |
| PRUAV024877 |  | GAGTCGGGAAAACATCAACACTGTGAAGGATTTTCTCACCCTACTCTTCATAGACTCTTCAAGGCTTCGGAAT~~~~~~~~~~~~~~~~~~~~~~~~~~~ |  |
| PavCALe_‘Regina’ |  | GAGTCGGGAAAACATCAACACTGTGAAGGATTTTCTCACCCTACTCTTCATAGACTCTTCAAGGCTTCGGAATGTAATTATTGACTTTTCTTATCTCGGT |  |
| PavCALe_‘Lapins’ |  | GAGTCGGGAAAACATCAACACTGTGAAGGATTTTCTCACCCTACTCTTCATAGACTCTTCAAGGCTTCGGAATGTAATTATTGACTTTTCTTATCTCGGT |  |
| PavCALe_‘Garnet’ |  | GAGTCGGGAAAACATCAACACTGTGAAGGATTTTCTCACCCTACTCTTCATAGACTCTTCAAGGCTTCGGAATGTAATTATTGACTTTTCTTATCTCGGT |  |
|  |  |  |  |
|  |  | ....|....|....|....|....|....|....|....|....|....|....|....|....|....|....|....|....|....|....|....| |  |
| ppa004675m |  | TAAATTTATTTTTCAGTGCTTTATAATTCACACTGATAAGTTTGAAGTTAGTGTCTTTTGCATTTAAATGATGCGTTATGTGTCTCAATCTAGTGACCAT |  |
| PRUAV024877 |  | ~~~~~~~~~~~~~~~~~~~~~~~~~~~~~~~~~~~~~~~~~~~~~~~~~~~~~~~~~~~~~~~~~~~~~~~~~~~~~~~~~~~~~~~~~~~~~~~~~~~~ |  |
| PavCALe_‘Regina’ |  | TAAATTTATTTTTCAATGCTTTATAATTCACACTGATAAGTTTGAAGTTAGTGTCTTTTGCATTTAAACGATGCGTTATGTGTCTCAATCTAGTGACCAT |  |
| PavCALe_‘Lapins’ |  | TAAATTTATTTTTCAATGCTTTATAATTCACACTGATAAGTTTGAAGTTAGTGTCTTTTGCATTTAAACGATGCGTTATGTGTCTCAATCTAGTGACCAT |  |
| PavCALe_‘Garnet’ |  | TAAATTTATTTTTCAATGCTTTATAATTCACACTGATAAGTTTGAAGTTAGTGTCTTTTGCATTTAAACGATGCGTTATGTGTCTCAATCTAGTGACCAT |  |
|  |  |  |  |
|  |  | ....|....|....|....|....|....|....|....|....|....|....|....|....|....|....|....|....|....|....|....| |  |
| ppa004675m |  | AAGAGTTTATTTATGTTAAAATTTATTTTATTATCTGGAAATGCAGATCCTTGGCACGGGTATGTCTGCTAAGATGTGGGAAGTCACAGTCGAGCATGCG |  |
| PRUAV024877 |  | ~~~~~~~~~~~~~~~~~~~~~~~~~~~~~~~~~~~~~~~~~~~~~~ATCCTTGGCACAGGTATGTCTGCTAAGATGTGGGAAGTCACAGTCGAGCATGCG |  |
| PavCALe_‘Regina’ |  | AAGAGTTT~~~TAT~~~~~~AT~~ATTTTATTATCTGGAAATGCAGATCCTTGGCACAGGTATGTCTGCTAAGATGTGGGAAGTCACAGTCGAGCATGCG |  |
| PavCALe_‘Lapins’ |  | AAGAGTTT~~~TAT~~~~~~AT~~ATTTTATTATCTGGAAATGCAGATCCTTGGCACAGGTATGTCTGCTAAGATGTGGGAAGTCACAGTCGAGCATGCG |  |
| PavCALe_‘Garnet’ |  | AAGAGTTT~~~TAT~~~~~~AT~~ATTTTATTATCTGGAAATGCAGATCCTTGGCACAGGTATGTCTGCTAAGATGTGGGAAGTCACAGTCGAGCATGCG |  |
|  |  |  |  |
|  |  | ....|....|....|....|....|....|....|....|....|....|....|....|....|....|....|....|....|....|....|....| |  |
| ppa004675m |  | CAGACATGTATACTTGATAAGAGGATGTACTTGTACTGCCCTCCCAGTTCACAGCAGAGAACCGGTGTGGTCTTCAACATTGTGGGG~CAAGTGATGGGA |  |
| PRUAV024877 |  | CAGACATGTATACTTGATAAGAGGATGTACTTGTACTGCCCTCCCAGTTCACAGCAGAGAACCGGTGTGGTCTTCAACATTGTGGGGGCAAGTGATGTGA |  |
| PavCALe_‘Regina’ |  | CAGACATGTATACTTGATAAGAGGATGTACTTGTACTGCCCTCCCAGTTCA |  |
| PavCALe_‘Lapins’ |  | CAGACATGTATACTTGATAAGAGGATGTACTTGTACTGCCCTCCCAGTTCA |  |
| PavCALe_‘Garnet’ |  | CAGACATGTATACTTGATAAGAGGATGTACTTGTACTGCCCTCCCAGTTCA |  |

***CBPf***

|  |  | ....|....|....|....|....|....|....|....|....|....|....|....|....|....|....|....|....|....|....|....| |  |
| --- | --- | --- | --- |
| ppa000516m | 3351 | TGTTGCACAAAAGCAAAAGTTTACAATTCGGGAAATATCTCCAGAATGGGGTTATGCCACTGAGGCCACAAAGGTATGTTTCGCATCATACTATGTCTGG |  |
| PRUAV004837 |  | TGTTGCACAAAAGCAAAAGTTTACAATTCGGGAAATATCTCCAGAATGGGGTTATGCCACTGAGGCTACTAAGGT~~~~~~~~~~~~~~~~~~~~~~~~~ |  |
| PavCALf_‘Regina’ |  | ~~~~~~~~~~~~~~~~~~~~~~~~~~~~~~~~~~~~~~~~~~~~~~~~~~~~~~~~~~~~~~~~~~~~~~~~~~~~~~~~~~~~~~~~~~~~~~~~~~~~ |  |
| PavCALf_‘Lapins’ |  | ~~~~~~~~~~~~~~~~~~~~~~~~~~~~~~~~~~~~~~~~~~~~~~~~~~~~~~~~~~~~~~~~~~~~~~~~~~~~~~~~~~~~~~~~~~~~~~~~~~~~ |  |
| PavCALf_‘Garnet’ |  | ~~~~~~~~~~~~~~~~~~~~~~~~~~~~~~~~~~~~~~~~~~~~~~~~~~~~~~~~~~~~~~~~~~~~~~~~~~~~~~~~~~~~~~~~~~~~~~~~~~~~ |  |
|  |  |  |  |
|  |  | ....|....|....|....|....|....|....|....|....|....|....|....|....|....|....|....|....|....|....|....| |  |
| ppa000516m |  | TAATGCTCCTTAGAATAATCATTCGGTTAATATGGAAGTAGTACTCCCATGATGTTCCCCGTGCTTGTTTTATAATTTTCTGGAGCATTCCTGAATCAAT |  |
| PRUAV004837 |  | ~~~~~~~~~~~~~~~~~~~~~~~~~~~~~~~~~~~~~~~~~~~~~~~~~~~~~~~~~~~~~~~~~~~~~~~~~~~~~~~~~~~~~~~~~~~~~~~~~~~~ |  |
| PavCALf_‘Regina’ |  | ~~~~~ctggaaataat~ccattccgtct~a~at~g~agtaga~~tcccct~~agttcc~~t~gct~g~tttata~~tt~c~ggagcattcctgataatct |  |
| PavCALf_‘Lapins’ |  | ~~~~~~c~~tag~aat~atcattcgact~a~atgg~agtagat~tc~~~~~tagttc~~~ttcgctgttt~a~ag~tt~c~~gagcattc~tgaatatct |  |
| PavCALf_‘Garnet’ |  | ~~~~gtcggtaggaataatcattcggctta~atg~~agtagtactc~~~~gtacgtcc~~ttgc~tggtttata~ttt~cgggaggcatc~tga~tatct |  |
|  |  |  |  |
|  |  | ....|....|....|....|....|....|....|....|....|....|....|....|....|....|....|....|....|....|....|....| |  |
| ppa000516m |  | CTTCATGTCTCTGCTCCTTAATGGAGTCCTTATCTAACAGTTCATTC~TCTGCTTCACCTTTTTT~CATATGATCATTATACTACAAATCAGTAAGGAAA |  |
| PRUAV004837 |  | ~~~~~~~~~~~~~~~~~~~~~~~~~~~~~~~~~~~~~~~~~~~~~~~~~~~~~~~~~~~~~~~~~~~~~~~~~~~~~~~~~~~~~~~~~~~~~~~~~~~~ |  |
| PavCALf_‘Regina’ |  | ~cagt~~~ctccgctcctt~atg~agtccgtatctaccagtccat~~ctctgcctcaccttttt~ccatatgatcat~atactacaagccagcaaggaaa |  |
| PavCALf_‘Lapins’ |  | ccagt~~tctctgctcgt~~atg~agtcgta~tcta~ccagtcat~~ctctgc~tcaacttttt~~catatgatcat~atactacaa~gcagca~ggaaa |  |
| PavCALf_‘Garnet’ |  | catgttct~tctgctcct~aatg~agtcctattctaacaagtcat~cctctgc~tcaccttttttccatatgatcattatactacaaaccagcaaggaaa |  |
|  |  |  |  |
|  |  | ....|....|....|....|....|....|....|....|....|....|....|....|....|....|....|....|....|....|....|....| |  |
| ppa000516m |  | AGTTGGATAT~GTTAACCTGAACTAAATCACTGAATAAAGCCCT~GTCACT~TCAGATTATCCCTAGTGAATATTTTCCA~CCAGGTGCTTCTTATGCTA |  |
| PRUAV004837 |  | ~~~~~~~~~~~~~~~~~~~~~~~~~~~~~~~~~~~~~~~~~~~~~~~~~~~~~~~~~~~~~~~~~~~~~~~~~~~~~~~~~~~~~~~~~~~~~~~~~~~~ |  |
| PavCALf_‘Regina’ |  | agt~ggatat~gtt~acctgaactaaatgactgaataaagccct~GTCACTATCAGAT~ATCCCTAGCGAATATTTTCCACCCAGGTGCTTCTTATGCTA |  |
| PavCALf_‘Lapins’ |  | agttggatattgtt~acctg~actaaatgactg~ataaagcccttgtcactatcagattatccctagcgaatatttt~CA~CCAGGTGCTTCTTATGCTA |  |
| PavCALf_‘Garnet’ |  | agttggatat~gttAACCTGAACTAAATGACTGAATAAAGCCCTTGTCACTATCAGATTATCCCTAGCGAATATTTTACA~CCAGGTGCTTCTTATGCTA |  |
|  |  |  |  |
|  |  | ....|....|....|....|....|....|....|....|....|....|....|....|....|....|....|....|....|....|....|....| |  |
| ppa000516m |  | CTAACAGTTTGAGTTATTAGTCCAGATCTCGATGACCAGATATCAAAATTACCAATAATCCAGT~~~ACAACAATCTGTCTAATCTAAACTGTTTCAGAA |  |
| PRUAV004837 |  | ~~~~~~~~~~~~~~~~~~~~~~~~~~~~~~~~~~~~~~~~~~~~~~~~~~~~~~~~~~~~~~~~~~~~~~~~~~~~~~~~~~~~~~~~~~~~~~~~~~~~ |  |
| PavCALf_‘Regina’ |  | CTAAAAGTTTGAGTTATTAGTCCAGATCTCGATGACCAGATATCAGAATTACCAATAATCCAGTGCAACAACAATCTGTCTAATCTAAACTGTTTCAGAA |  |
| PavCALf_‘Lapins’ |  | CTAAAAGTTTGAGTTATTAGTCCAGATCTCGATGACCAGATATCAGAATTACCAATAATCCAGTGCAACAACAATCTGTCTAATCTAAACTGTTTCAGAA |  |
| PavCALf_‘Garnet’ |  | CTAAAAGTTTGAGTTATTAGTCCAGATCTCGATGACCAGATATCAGAATTACCAATAATCCAGTGCAACAACAATCTGTCTAATCTAAACTGTTTCAGAA |  |
|  |  |  |  |
|  |  | ....|....|....|....|....|....|....|....|....|....|....|....|....|....|....|....|....|....|....|....| |  |
| ppa000516m |  | ACCAAATACCCTGGTCTTGT~AAGGGTAGCCTGTCTATGCTTTACTTATTATTGGTCCCTCAGAGCAAAATGAACTCTACAAAAATCAACTTGGAATGTT |  |
| PRUAV004837 |  | ~~~~~~~~~~~~~~~~~~~~~~~~~~~~~~~~~~~~~~~~~~~~~~~~~~~~~~~~~~~~~~~~~~~~~~~~~~~~~~~~~~~~~~~~~~~~~~~~~~~~ |  |
| PavCALf_‘Regina’ |  | ACCAAATACCCTGGTCTTGTAA~GGGTAGCCTGTCTATGCTTTACTTTTTATTGGTCCCTCAGACCAAAATGAACTCTACAAAAATCAACTTGGAATGTT |  |
| PavCALf_‘Lapins’ |  | ACCAAATACCCTGGTCTTGTAAAGGGTAGCCTGTCTATGCTTTACTTTTTATTGGTCCCTCAGACCAAAATGAACTCTACAAAAATCAACTTGGAATGTT |  |
| PavCALf_‘Garnet’ |  | ACCAAATACCCTGGTCTTGTAAAGGGTAGCCTGTCTATGCTTTACTTTTTATTGGTCCCTCAGACCAAAATGAACTCTACAAAAATCAACTTGGAATGTT |  |
|  |  |  |  |
|  |  | ....|....|....|....|....|....|....|....|....|....|....|....|....|....|....|....|....|....|....|....| |  |
| ppa000516m |  | GATCTTACATTGTCTGTAAGAAAATAATGAGTTAATACTGCAATTTTGATTTAAGCTGCAATCAACGTATGATCTGATCAGTATAAAAAACAATAATTAA |  |
| PRUAV004837 |  | ~~~~~~~~~~~~~~~~~~~~~~~~~~~~~~~~~~~~~~~~~~~~~~~~~~~~~~~~~~~~~~~~~~~~~~~~~~~~~~~~~~~~~~~~~~~~~~~~~~~~ |  |
| PavCALf_‘Regina’ |  | GATCTTACATTGTCTGTAAGAAAATAATGAGTTAATACTGTAATTTTGATTTAAGCTGCAATCAACGTATGATCTGATCAGTATAAAAAACAATAATTAA |  |
| PavCALf_‘Lapins’ |  | GATCTTACATTGTCTGTAAGAAAATAATGAGTTAATACTGTAATTTTGATTTAAGCTGCAATCAACGTATGATCTGATCAGTATAAAAAACAATAATTAA |  |
| PavCALf_‘Garnet’ |  | GATCTTACATTGTCTGTAAGAAAATAATGAGTTAATACTGTAATTTTGATTTAAGCTGCAATCAACGTATGATCTGATCAGTATAAAAAACAATAATTAA |  |
|  |  |  |  |
|  |  | ....|....|....|....|....|....|....|....|....|....|....|....|....|....|....|....|....|....|....|....| |  |
| ppa000516m |  | AACCAGTCATGTTCATGGGTACTCTC~~GTGTGCATTTTAGGCCAAGCAGTCTTTTTCTTTTTTAATGTGATATTTACTTCTTCTTTTTAAAAAAAAAAA |  |
| PRUAV004837 |  | ~~~~~~~~~~~~~~~~~~~~~~~~~~~~~~~~~~~~~~~~~~~~~~~~~~~~~~~~~~~~~~~~~~~~~~~~~~~~~~~~~~~~~~~~~~~~~~~~~~~~ |  |
| PavCALf_‘Regina’ |  | AACCAGTCATGTTCATGGGTACTGTCTTATGTGCATTTTAGGCCAAGCAGTCTTTTTCTTTTTTAATGTGATATTTACTTTTTTTTTTTAAA~~~~~~~T |  |
| PavCALf_‘Lapins’ |  | AACCAGTCATGTTCATGGGTACTGTTTTATGTGCATTTTAGGCCAAGCAGTCTTTTTCTTTTTTAATGTGATATTTACTTTTTTTTTTTAAA~~~~~~~T |  |
| PavCALf_‘Garnet’ |  | AACCAGTCATGTTCATGGGTACTGTCTTATGTGCATTTTAGGCCAAGCAGTCTTTTTCTTTTTTAATGTGATATTTACTTTTTTTTTTTAAA~~~~~~~T |  |
|  |  |  |  |
|  |  | ....|....|....|....|....|....|....|....|....|....|....|....|....|....|....|....|....|....|....|....| |  |
| ppa000516m |  | TAAAAAATTTGGCCAAGTGACAATGCAGAGAACTGTTGAGTATCCACCTTATAACAAAGTCCTAATACGATAGATTCTCCCTTGGATTTGTTTTTCCCCA |  |
| PRUAV004837 |  | ~~~~~~~~~~~~~~~~~~~~~~~~~~~~~~~~~~~~~~~~~~~~~~~~~~~~~~~~~~~~~~~~~~~~~~~~~~~~~~~~~~~~~~~~~~~~~~~~~~~~ |  |
| PavCALf_‘Regina’ |  | TTAAAAATTTGGACATGTGACAATGCAGAGAACTGTTGAGTGTCCACCTTATAACAAAGTCCTAATACGATAGATTCTCCCTTGGATTTGTTCTTCCCAA |  |
| PavCALf_‘Lapins’ |  | TTAAAAATTTGGACATGTGACAATGCAGAGAACTGTTGAGTGTCCACCTTATAACAAAGTCCTAATACGATAGATTCTCCCTTGGATTTGTTCTTCCC~A |  |
| PavCALf_‘Garnet’ |  | TTAAAAATTTGGACATGTGACAATGCAGAGAACTGTTGAGTGTCCACCTTATAACAAAGTCCTAATACGATAGATTCTCCCTTGGATTTGTTCTTCCC~A |  |
|  |  |  |  |
|  |  | ....|....|....|....|....|....|....|....|....|....|....|....|....|....|....|....|....|....|....|....| |  |
| ppa000516m |  | AAAAAAAAAAA~~TTTCTTTGTGGAGCTTTTGTTTTTTGTAGTTTAAGATTAAACTTAAAGCCCACGTTGCTCGATGCAGGTCATCATTGTTGGTTCTTT |  |
| PRUAV004837 |  | ~~~~~~~~~~~~~~~~~~~~~~~~~~~~~~~~~~~~~~~~~~~~~~~~~~~~~~~~~~~~~~~~~~~~~~~~~~~~~~~~~~CATCATTGTTGGATCTTT |  |
| PavCALf_‘Regina’ |  | AAAAA~GAAATTGTTTCTTTGTGGAGCTTTTGTTTTTGGTAGTTTAAGATTAAACTTAAAGCCCACATTGCTCGATGCAGGTCATCATTGTTGGATCTTT |  |
| PavCALf_‘Lapins’ |  | AAAAAAGAAATTGTTTCTTTGTGGAGCTTTTGTTTTTGGTAGTTTAAGATTAAACTTAAAGCCCACATTGCTCGATGCAGGTCATCATTGTTGGATCTTT |  |
| PavCALf_‘Garnet’ |  | AAAAAAGAAATTGTTTCTTTGTGGAGCTTTTGTTTTTGGTAGTTTAAGATTAAACTTAAAGCCCACATTGCTCGATGCAGGTCATCATTGTTGGATCTTT |  |
|  |  |  |  |
|  |  | ....|....|....|....|....|....|....|....|....|....|....|....|....|....|....|....|....|....|....|....| |  |
| ppa000516m |  | TCTGTGCGATCCATCAGATTCTGCATGGAGTTGTATGTTTGGTGACATTGAAGTCCCTGCTCAGATCATTCAGGACGGTGTACTCTGTTGTGAAGCTCCT |  |
| PRUAV004837 |  | TCTGTGTGATCCATCAGAATCTGCATGGAGTTGTATGTTTGGTGACGTTGAAGTCCCTGCTCAGATCATTCAGGACGGTGTACTCCGTTGTGAAGCTCCT |  |
| PavCALf_‘Regina’ |  | TCTGTGTGATCCATCAGAATCTGCATGGAGTTGTATGTTTGGTGACGTTGAAGTCCCTGCTCAGATCATTCAGGACGGTGTACTCCGTTGTGAAGCTCCT |  |
| PavCALf_‘Lapins’ |  | TCTGTGTGATCCATCAGAATCTGCATGGAGTTGTATGTTTGGTGACGTTGAAGTCCCTGCTCAGATCATTCAGGATGGTGTACTCCGTTGTGAAGCTCCT |  |
| PavCALf_‘Garnet’ |  | TCTGTGTGATCCATCAGAATCTGCATGGAGTTGTATGTTTGGTGACGTTGAAGTCCCTGCTCAGATCATTCAGGACGGTGTACTCCGTTGTGAAGCTCCT |  |
|  |  |  |  |
|  |  | ....|....|....|....|....|....|....|....|....|....|....|....|....|....|....|....|....|....|....|....| |  |
| ppa000516m |  | CCTCACCTTTTTGGGAAGGTGACTATCTGCATTACTTCAAGCAACCGGGTGTCCTGCAGTGAAGTCAGAGAGTTTGAGTATCGAGTTAAGGGCAGCAGTG |  |
| PRUAV004837 |  | CCTCATCTTTTTGGGAAAGTGACTATCTGCATTACTTCAAGCAACCGGGTGTCCTGCAGTGAAGTCAGGGAGTTTGAGTATCGAGTTAAGAGCAGTAGTG |  |
| PavCALf_‘Regina’ |  | CCTCATCTTTTTGGGAAAGTGACTATCTGCATTACTTCAAGCAACCGGGTGTCCTGCAGTGAAGTCAGGGAGTTTGAGTATCGAGTTAAGAGCAGTAGTG |  |
| PavCALf_‘Lapins’ |  | CCTCATCTTTTTGGGAAAGTGACTATCTGCATTACTTCAAGCAACCGGGTGTCCTGCAGTGAAGTCAGGGAGTTTGAGTATCGAGTTAAGAGCAGTAGTG |  |
| PavCALf_‘Garnet’ |  | CCTCATCTTTTTGGGAAAGTGACTATCTGCATTACTTCAAGCAACCGGGTGTCCTGCAGTGAAGTCAGGGAGTTTGAGTATCGAGTTAAGAGCAGTAGTG |  |
|  |  |  |  |
|  |  | ....|....|....|....|....|....|....|....|....|....|....|....|....|....|....|....|....|....|....|....| |  |
| ppa000516m |  | GTACTAATAACTCGCCTCCAACAGAAACTACCAAGAGTGCTGAAGAGCTGTTATTACTTGTCAGGTTCGTTCAAATGCTCATGTCTGATTCATCAATGCA |  |
| PRUAV004837 |  | GTACTAATCACTCGCCTCCACCAGAAACTACCAAGAGTGCTGAAGAGCTGTTATTACTTGTCAGATTCGTTCAAACGCTCTTGTCTGATTCATCAATGCA |  |
| PavCALf_‘Regina’ |  | GTACTAATCACTCGCCTCCACCAGAAACTACCAAGAGTGCTGAAGAGCTGTTATTACTTGTCAGATTCGTTCAAACGCTCTTGTCTGATTCATCAATGCa |  |
| PavCALf_‘Lapins’ |  | GTACTAATCACTCGCCTCCACCAGAAACTACCAAGAGTGCTGAAGAGCTGTTATTACTTGTCAGATTCGTTCAAACGCTCTTGTCTGATTCATCAATGCA |  |
| PavCALf_‘Garnet’ |  | GTACTAATCACTCGCCTCCACCAGAAACTACCAAGAGTGCTGAAGAGCTGTTATTACTTGTCAGATTCGTTCAAACGCTCTTGTCTGATTCATCAATGCA |  |
|  |  |  |  |
|  |  | ....|....|....|....|....|....|....|....|....|....|....|....|....|....|....|....|....|....|....|....| |  |
| ppa000516m |  | GAACAGAGATAGTGTTGAACCTGAGACCCTGAGGAGATTGAAAGCTGACGATGATTCATGGGATAGTATCATTGAGGCTTTATTACTCGGCAGTGGAAGT |  |
| PRUAV004837 |  | GAACAGAGATGGTGTTGAACCTGAGACCCTGAGGAGATTGAAAGCCGACGATGATTCATGGGGTAGTATCATTGAGGCTTTATTACTCGGCAGTGGAAGT |  |
| PavCALf_‘Regina’ |  | Gaacaggaag |  |
| PavCALf_‘Lapins’ |  | GAACAGAGATGGGTAATCA |  |
| PavCALf_‘Garnet’ |  | Ggaacaggag |  |

***CLF***

|  |  | ....|....|....|....|....|....|....|....|....|....|....|....|....|....|....|....|....|....|....|....| |  |
| --- | --- | --- | --- |
| ppa001213m | 2601 | AAAAGAAGTTGGCTTCTCTGATCCAGTGCTGGAGTCATTAGCACAGTGTTTCTCTAGAAGTCCTTCTGAAGTCAAGGTATGTCAGTTTCTTTTTTCTATA |  |
| PRUAV072866 |  | ~~~~~~~~~~~~~~~~~~~~~~~~~~~~~~~~~~~~~~~~~~~~~~~~~~~~~~~~~~~~~~~~~~~~~~~~~aag~~~~~~~~~~~~~~~~~~~~~~~~ |  |
| PRUAV034373 |  | ~~~~~~~~~~~~~~~~~~~~~~~~~~~~~~~~~~~~~~~~~~~~~~~~~~~~~~~~~~~~~~~~~~~~~~~~~~~~~~~~~~~~~~~~~~~~~~~~~~~~ |  |
| PavCLF_‘Regina’ |  | ~~~~~~~~~~~~~~~~~~~~~~~~~~~~~~~~~~~~~~~~~~~~~~~~~~~~~~~~~~~~~~~~~~~~~~~~~~~~~~~~~~~~~~~~~~~~~~~~~~~~ |  |
| PavCLF_‘Lapins’ |  | ~~~~~~~~~~~~~~~~~~~~~~~~~~~~~~~~~~~~~~~~~~~~~~~~~~~~~~~~~~~~~~~~~~~~~~~~~~~~~~~~~~~~~~~~~~~~~~~~~~~~ |  |
| PavCLF_‘Garnet’ |  | ~~~~~~~~~~~~~~~~~~~~~~~~~~~~~~~~~~~~~~~~~~~~~~~~~~~~~~~~~~~~~~~~~~~~~~~~~~~~~~~~~~~~~~~~~~~~~~~~~~~~ |  |
|  |  |  |  |
|  |  | ....|....|....|....|....|....|....|....|....|....|....|....|....|....|....|....|....|....|....|....| |  |
| ppa001213m |  | TTACTGGACTACTTTGCCCACTCTGCCATGCTTCTGTCCAATTTATAACACGATAAAAACCAGTTAGTGTAAGCTTTGGTCCAGCCGTTTCCCATTTGCT |  |
| PRUAV072866 |  | ~~~~~~~~~~~~~~~~~~~~~~~~~~~~~~~~~~~~~~~~~~~~~~~~~~~~~~~~~~~~~~~~~~~~~~~~~~~~~~~~~~~~~~~~~~~~~~~~~~~~ |  |
| PRUAV034373 |  | ~~~~~~~~~~~~~~~~~~~~~~~~~~~~~~~~~~~~~~~~~~~~~~~~~~~~~~~~~~~~~~~~~~~~~~~~~~~~~~~~~~~~~~~~~~~~~~~~~~~~ |  |
| PavCLF_‘Regina’ |  | ~~~~~~~~~~~~~~~~~~~~~~~~~~~~~~~~~~~~~~~~~~~~~~~~~~~~~~~~~~~~~~~~~~~~~~~~~~~~~~~~~~~~~~~~~~~~~~~~~~~~ |  |
| PavCLF_‘Lapins’ |  | ~~~~~~~~~~~~~~~~~~~~~~~~~~~~~~~~~~~~~~~~~~~~~~~~~~~~~~~~~~~~~~~~~~~~~~~~~~~~~~~~~~~~~~~~~~~~~~~~~~~~ |  |
| PavCLF_‘Garnet’ |  | ~~~~~~~~~~~~~~~~~~~~~~~~~~~~~~~~~~~~~~~~~~~~~~~~~~~~~~~~~~~~~~~~~~~~~~~~~~~~~~~~~~~~~~~~~~~~~~~~~~~~ |  |
|  |  |  |  |
|  |  | ....|....|....|....|....|....|....|....|....|....|....|....|....|....|....|....|....|....|....|....| |  |
| ppa001213m |  | TTGTGCTGGCTTTACAGAGATATGA~AACACCATCAAACGTCTTATACAGGCAAGATATGATACTCTCGTAAAGGAAGAGGAGGCTGTAGGGGG~TTGTA |  |
| PRUAV072866 |  | ~~~~~~~~~~~~~~~~~agatatganaacaccatcaaacgtcttatacaggcaagatatgatactctcgtaaaggaagaggaggctgtgggggg~ttgta |  |
| PRUAV034373 |  | ~~~~~~~~~~~~~~~~~~~~~~~~~~~~~~~~~~~~~~~~~~~~~~~~~~~~~~~~~~~~~~~~~~~~~~~~~~~~~~~~~~~~~~~~~~~~~~~~~~~~ |  |
| PavCLF_‘Regina’ |  | ~~~~~~~~~~~~~~~~~~~~~~~~~~~~~~~~~~~~~~~~~~~~~~~~~~~~~~~~~~~~~~~~~~~~~~~~~~~~~~~~~~~~~~~~~~~~~~~~~~~~ |  |
| PavCLF_‘Lapins’ |  | ~~~~~~~~~~~~~~~~~~~~~~~~~~~~~~~~~~~~~~~~~~~~~~~~~~~~~~~~~~~~~~~~~~~~~~~~~~~~~~~~~~~~~~~~~~~~~~~~~~~~ |  |
| PavCLF_‘Garnet’ |  | ~~~~~~~~~~~~~~~~~~~~~~~~~~~~~~~~~~~~~~~~~~~~~~~~~~~~~~~~~~~~~~~~~~~~~~~~~~~~~~~~~~~~~~~~~~~~~~~~~~~~ |  |
|  |  |  |  |
|  |  | ....|....|....|....|....|....|....|....|....|....|....|....|....|....|....|....|....|....|....|....| |  |
| ppa001213m |  | ACTATGGATAATGAAGACATTTCACAAAATGGGAATTATTTTCTTGATAAA~GATCTAGATGCAGCTCTGGATTCATTTGACAACCTATTTTGTCGTCGA |  |
| PRUAV072866 |  | actatggataatgaagacatttcacaaaatgggaattattttcttgataaa~gatctagatgctgctctggattcatttgacaacctattttgtcgtcga |  |
| PRUAV034373 |  | ~~~~~~~~~~~~~~~~~~~~~~~~~~~~~~~~~~~~~~~~~~~~~~~~~~~~~~~~~~~~~~~~~~~~~~~~~~~~~~~~~~~~~~~~~~~~~~~~~~~~ |  |
| PavCLF_‘Regina’ |  | ~~~~~~~~~~~~~~~~~~~~~~~~~~~~~~~~~~~~~~~~~~~~~~~~~~~~~~~~~~~~~~~~~~~~~~~~~~~~~~~~~~~~~~~~~~~cgtgaagtt |  |
| PavCLF_‘Lapins’ |  | ~~~~~~~~~~~~~~~~~~~~~~~~~~~~~~~~~~~~~~~~~~~~~~~~~~~~~~~~~~~~~~~~~~~~~~~~~~~~~~~~~~~~~~~~~~~~~~~~~~~c |  |
| PavCLF_‘Garnet’ |  | ~~~~~~~~~~~~~~~~~~~~~~~~~~~~~~~~~~~~~~~~~~~~~~~~~~~~~~~~~~~~~~~~~~~~~~~~~~~~~~~~~~~~~~~~~~~~~~~~~~~A |  |
|  |  |  |  |
|  |  | ....|....|....|....|....|....|....|....|....|....|....|....|....|....|....|....|....|....|....|....| |  |
| ppa001213m |  | TGCCTTGTAAGTATTCCTTTTGTAAATTCACTTGCATGTTTCTTTTTTAGTGATATTTTTTCTTAGTAGTTTCTCTGCTGTTTCTTTTTGGCAGGTGTTT |  |
| PRUAV072866 |  | tgcctt~~~~~~~~~~~~~~~~~~~~~~~~~~~~~~~~~~~~~~~~~~~~~~~~~~~~~~~~~~~~~~~~~~~~~~~~~~~~~~~~~~~~~~~~gtgttt |  |
| PRUAV034373 |  | ~~~~~~~~~~~~~~~~~~~~~~~~~~~~~~~~~~~~~~~~~~~~~~~~~~~~~~~~~~~~~~~~~~~~~~~~~~~~~~~~~~~~~~~~~~~~~~~~~~~~ |  |
| PavCLF_‘Regina’ |  | gtgctgctgtggatt~ctttgtgctgctctggattcatttgtgctgctctggattcatttgcgctgctctggatctGTTGGTTCTTTTTGGGAGGTGTTT |  |
| PavCLF_‘Lapins’ |  | tg~cctTGTAGTATTCCTTTTGTAA~TTCACTTGCATGTTTCTTTTTTTGTGATATCTTTTCTTAGTAGTTTCTCTGCTGTTTCTTTTTGGCAGGTGTTT |  |
| PavCLF_‘Garnet’ |  | GGCCCTTGTAGTATTCCTTTTGTAAATTCACTTGCATGTTTCTTTTTTTGTGATATCTTTTCTTAGTAGTTTCTCTGCTGTTTCTTTTTGGCAGGTGTTT |  |
|  |  |  |  |
|  |  | ....|....|....|....|....|....|....|....|....|....|....|....|....|....|....|....|....|....|....|....| |  |
| ppa001213m |  | GATTGCAGATTACACGGATGTTCACAGGATCTTGTCTTTCCTGTAAGTAATTGTTTAATAGCTGTCTGAATATGGGACCAGATTTTTATACCCTGCACTG |  |
| PRUAV072866 |  | gattgcagattacatggatgttcacaggatcttgtctttcct~~~~~~~~~~~~~~~~~~~~~~~~~~~~~~~~~~~~~~~~~~~~~~~~~~~~~~~~~~ |  |
| PRUAV034373 |  | ~~~~~~~~~~~~~~~~~~~~~~~~~~~~~~~~~~~~~~~~~~~~~~~~~~~~~~~~~~~~~~~~~~~~~~~~~~~~~~~~~~~~~~~~~~~~~~~~~~~~ |  |
| PavCLF_‘Regina’ |  | GATTGCAGATTACATGGATGTTCACAGGATCTTGTCTTTCCTGTAAGTAATTGTTTAATAGCTGTCTGAATGTGGCACCAGACTTTTATACCCTGCGCTG |  |
| PavCLF_‘Lapins’ |  | GATTGCAGATTACATGGATGTTCACAGGATCTTGTCTTTCCTGTAAGTAATTGTTTAATAGCTGTCTGAATGTGGCACCAGACTTTTATACCCTGCGCTG |  |
| PavCLF_‘Garnet’ |  | GATTGCAGATTACATGGATGTTCACAGGATCTTGTCTTTCCTGTAAGTAATTGTTTAATAGCTGTCTGAATGTGGCACCAGACTTTTATACCCTGCGCTG |  |
|  |  |  |  |
|  |  | ....|....|....|....|....|....|....|....|....|....|....|....|....|....|....|....|....|....|....|....| |  |
| ppa001213m |  | ATAAAGAAAAATATTGTCAGGCTGAGAAACAACCTCCTTGGAGCTCTCCGGATGCCGAAAATGCATCATGTGGTCCAAATTGCTATCGATCAGTACGTGT |  |
| PRUAV072866 |  | ~~~~~~~~~~~~~~~~~~~~gctgagaaacaacctccttggagctctccagataccgaaaatgcatcatgtggtccaaattgctatcgatca~~~~~~~~ |  |
| PRUAV034373 |  | ~~~~~~~~~~~~~~~~~~~~~~~~~~~~~~~~~~~~~~~~~~~~~~~~~~~~~~~~~~~~~~~~~~~~~~~~~~~~~~~~~~~~~~~~~~~~~~~~~~~~ |  |
| PavCLF_‘Regina’ |  | ATAAAGAAAAATATTGTCAGGCTGAGAAACAACCTCCTTGGAGCTCTCCAGATRCCGAAAATGCATCATGTGGTCCAAATTGCTATCGATCAGTACGTGT |  |
| PavCLF_‘Lapins’ |  | ATAAAGAAAAATATTGTCAGGCTGAGAAACAACCTCCTTGGAGCTCTCCAGATRCCGAAAATGCATCATGTGGTCCAAATTGCTATCGATCAGTACGTGT |  |
| PavCLF_‘Garnet’ |  | ATAAAGAAAAATATTGTCAGGCTGAGAAACAACCTCCTTGGAGCTCTCCAGATGCCGAAAATGCATCATGTGGTCCAAATTGCTATCGATCAGTACGTGT |  |
|  |  |  |  |
|  |  | ....|....|....|....|....|....|....|....|....|....|....|....|....|....|....|....|....|....|....|....| |  |
| ppa001213m |  | GTTGTGTAGCTGGGAATGTTGTAAAGAATTGACAAACAAATGATTTATGTGGTTTCAAACTGTCTCATTTCAGGTACTGAAATCTGAAAGAATTGCTAGA |  |
| PRUAV072866 |  | ~~~~~~~~~~~~~~~~~~~~~~~~~~~~~~~~~~~~~~~~~~~~~~~~~~~~~~~~~~~~~~~~~~~~~~~~~gtactgaaatctgaaagaattgctaga |  |
| PRUAV034373 |  | ~~~~~~~~~~~~~~~~~~~~~~~~~~~~~~~~~~~~~~~~~~~~~~~~~~~~~~~~~~~~~~~~~~~~~~~~~~~~~~~~~~~~~~~~~~~~~~~~~~~~ |  |
| PavCLF_‘Regina’ |  | GTTGTTTAGCTGGGAATGTTGTAAAGAATTGGCAAACAAATGATTTATGTGGTTTCAAACTGTCTCATTTCAGGTACTGAAATCTGAAAGAATTGCTAGA |  |
| PavCLF_‘Lapins’ |  | GTTGTTTAGCTGGGAATGTTGTAAAGAATTGGCAAACAAATGATTTATGTGGTTTCAAACTGTCTCATTTCAGGTACTGAAATCTGAAAGAATTGCTAGA |  |
| PavCLF_‘Garnet’ |  | GTTGTTTAGCTGGGAATGTTGTAAAGAATTGGCAAACAAATGATTTATGTGGTTTCAAACTGTCTCTTTTC~~GTGCAGagatctcagaaaaatgtgata |  |
|  |  |  |  |
|  |  | ....|....|....|....|....|....|....|....|....|....|....|....|....|....|....|....|....|....|....|....| |  |
| ppa001213m |  | GTGAGCTCTGGTGATGTTGAAGAAAAAAATGTCACATCATTAGATGGTGCTAGTGCTCAAACGTCAACGAGAAAGAAGTCTTCTGTTATATCTGCTAGAA |  |
| PRUAV072866 |  | gtgagctctgttgatgtt~~agaaa~~~~~g~~~~~~ |  |
| PRUAV034373 |  | ~~~~~~~~~~~~~~~~~~~~~~~~~~~~~~~~~~~~~~~~~~~~~~~~~~~~~~~~~~~~~~~~~~~~~~~~~~~~~~~~~~~~~~~~~~~~~~~~~~~~ |  |
| PavCLF_‘Regina’ |  | GTGAGCTCTGTTGATGTTGAAGAAAAAAATGTCACATCATTAGATGGTGCTAGTGCTCAAATGTCAACGAGAAAGAAGTCTTCTGTTATATCTGCTAGAA |  |
| PavCLF_‘Lapins’ |  | GTGAGCTCTGTTGATGTTGAAGAAAAAAATGTCACATCATTAGATGGTGCTAGTGCTCAAATGTCAACGAGAAAGAAGTCTTCTGTTATATCTGCTAGAA |  |
| PavCLF_‘Garnet’ |  | gagagatctgtggagatgtaaaaaaaaaatgtgtcatctctatatgtggcgctagcgctcatgtgtccaagaaaaagatctcctgtgatatctgcgctag |  |
|  |  |  |  |
|  |  | ....|....|....|....|....|....|....|....|....|....|....|....|....|....|....|....|....|....|....|....| |  |
| ppa001213m |  | AGAAGGTAAAGTCCGGCCAAAGTGAAAGTGCTTCATCTAATGCAAAAGCCATCTCAGAAAGCAGTGATTCGGAGAATGGACCCAGGCAGGATGCTACTCA |  |
| PRUAV072866 |  |  |  |
| PRUAV034373 |  | ~~~~~~~~~~~~~~~~~~~~~~~~~~~~~~~~~~~~~~~~~~~~~~~~~~~~~~~~~~~~~~~~~~~~~~~~~~~~~~~~~~~~~~~~~~~~~~~~~~~~ |  |
| PavCLF_‘Regina’ |  | AGAAGGTAAAGTCCGGCCAAAGTGAAAGTGCTTCATCTAATGCAAAAGCCATCTCAGAAAGCAGTGATTCGGAGAATGGACCCAGGCAGGATGCTACCCA |  |
| PavCLF_‘Lapins’ |  | AGAAGGTAAAGTCCGGCCAAAGTGAAAGTGCTTCATCTAATGCAAAAGCCATCTCAGAAAGCAGTGATTCGGAGAATGGACCCAGGCAGGATGCTACCCA |  |
| PavCLF_‘Garnet’ |  | agaaagtgtagtctcggcccaatgagagtgcgtctcatcaagcgcaagccctctctcagagcactgtgtctcagagtgggcacccgcgcgagatgctaca |  |
|  |  |  |  |
|  |  | ....|....|....|....|....|....|....|....|....|....|....|....|....|....|....|....|....|....|....|....| |  |
| ppa001213m |  | TCATCAAACACCCTCAAAGACTAAGCTAGCAGGAAAAAGTGGAATTGGCAAGAGGAACAGCAAGAGAGTTGCAGAGCGTGTTCTAGTTTGCATGCAGAAG |  |
| PRUAV072866 |  |  |  |
| PRUAV034373 |  | ~~~~~~~~~~~~~~~~~~~~~~~~~~~~~~~~~~~~~~~~~~~~~~~~~~~~~~~~~~~~~~~~~~~~~~~~~~~~~~~~~~~~~~~~~~~~~~~~~~~~ |  |
| PavCLF_‘Regina’ |  | TCATCAAACACCCTCAAAGACTAAGCTAGCAGGAAAAAGTGGAATTGGCAAGAGGAACAGCAAGAGAGTTGCAGAGCGTGTTCTAGTTTGCATGCAGAAG |  |
| PavCLF_‘Lapins’ |  | TCATCAAACACCCTCAAAGACTAAGCTAGCAGGAAAAAGTGGAATTGGCAAGAGGAACAGCAAGAGAGTTGCAGAGCGTGTTCTAGTTTGCATGCAGAAG |  |
| PavCLF_‘Garnet’ |  | tcatcatcacacactctcagacaaactctcacgagaaagtgtggttgtgacgagaagcacaacgagagagtgcagagcgtgctctattgcgtgtgcagga |  |
|  |  |  |  |
|  |  | ....|....|....|....|....|....|....|....|....|....|....|....|....|....|....|....|....|....|....|....| |  |
| ppa001213m |  | AGGCAGAAGAAAATGGTGGTTTCAGATTCTGATTCCATTGTTAATGCTGGTCTTTGTCCAAGTGATATGAAGCTTAGATCAAATTCATGCAAAGATAATG |  |
| PRUAV072866 |  |  |  |
| PRUAV034373 |  | ~~~~~~~~~~~~~~~~~~~~~~~~~~~~~~~~~~~~~~~~~~~~~~~~~~~~~~~~~~~~~~~~~~~~~~~~~~~~~~~~~~~~~~atgcaaagataatg |  |
| PavCLF_‘Regina’ |  | AGGCAGAAGAAAATGGTGGTTTCTGATTCTGATTCCATTGTTAATGCTGGTCTTTGTCCAAGTGATATGAAGCTTAGATCAAATTCATGCAAAGATAATG |  |
| PavCLF_‘Lapins’ |  | AGGCAGAAGAAAATGGTGGTTTCTGATTCTGATTCCATTGTTAATGCTGGTCTTTGTCCAAGTGATATGAAGCTTAGATCAAATTCATGCAAAGATAATG |  |
| PavCLF_‘Garnet’ |  | gacgcagaagatgtggtggtctcagactcagacactgtgatgtgctgtctgtgtccgtgagatgagcgcagatctcatactcgcgcagatatgagacact |  |
|  |  |  |  |
|  |  | ....|....|....|....|....|....|....|....|....|....|....|....|....|....|....|....|....|....|....|....| |  |
| ppa001213m |  | AAGACACTAGTTCGTCTTCACAAAAAAATCTGAAATCTTCTACCAGTGGAGGGTTTAGGAGGATGGAATCACCTACCAAGGGCAGACACAAAGTAGTTCA |  |
| PRUAV072866 |  |  |  |
| PRUAV034373 |  | aagacactagttcttcttcacaaaagaatctgaaatcttctaccagtggagggtttaggaggaaggaatcacctactaagggcagacacaaagtagttca |  |
| PavCLF_‘Regina’ |  | AAGACACTAGTTCTTCTTCACAAAAGAATCTGAAATCTTCTACCAGTGGAGGGTTAAGGGAGGAAGGAG |  |
| PavCLF_‘Lapins’ |  | AGGACActagttcttcttcacaaaagaatctgaaatcttctaccagtggaggtagtaagagagaagaggga |  |
| PavCLF_‘Garnet’ |  | cgatctccttctcacagagactctgatctctatacagtagagtatgggagaagaaaaaggg |  |

***COL2***

|  |  | ....|....|....|....|....|....|....|....|....|....|....|....|....|....|....|....|....|....|....|....| |  |
| --- | --- | --- | --- |
| ppa026514m | 1 | ATGAAAGCTAGGGTTTGCGAGCTCTGCGACCAAGAAGCTTCGCTCTACTGCCCCTCCGATTCCGCCTTCCTCTGCTCCCGCTGCGACGCTAGGGTCCACC |  |
| PRUAV017210 |  | atgaaagccagggtttgcgagctctgcgaccaagaagcttcgctctactgcccctccgattccgccttcctctgctcccgctgcgacgctaaggtccact |  |
| PavCOL2_‘Regina’ |  | ~~~~~~~~~~~~~~~~~~~~~~~~~~~~~~~~~~~~~~~~~~~~~~~~~~~~~~~~~~~~~~~~~~~~~~~~~~~~~~~~~~~~~~~~~~~~~~~~~~~~ |  |
| PavCOL2_‘Lapins’ |  | ~~~~~~~~~~~~~~~~~~~~~~~~~~~~~~~~~~~~~~~~~~~~~~~~~~~~~~~~~~~~~~~~~~~~~~~~~~~~~~~~~~~~~~~~~~~~~~~~~~~~ |  |
| PavCOL2_‘Garnet’ |  | ~~~~~~~~~~~~~~~~~~~~~~~~~~~~~~~~~~~~~~~~~~~~~~~~~~~~~~~~~~~~~~~~~~~~~~~~~~~~~~~~~~~~~~~~~~~~~~~~~~~~ |  |
|  |  |  |  |
|  |  | ....|....|....|....|....|....|....|....|....|....|....|....|....|....|....|....|....|....|....|....| |  |
| ppa007007m |  | AGGCCAACTTCCTCGTCGCTCGCCACATCCGCCAATATATCTGCTACAATTGCAAGGGTCTTACCGGAAGTCGGAATATCCGGTCATTCTGCTCGTCATG |  |
| PRUAV007294 |  | cggccaacttcctcgtcgctcgccacatccgccaatctatctgctacaattgtaaaggtcttgccggaagtcggaatctacagtcactctgctcgtcatg |  |
| PavCOL2_‘Regina’ |  | ~~~~~~~~~~~~~~~~~~~~~~~~~~~~~~~~~~~~~~~~~~~~~~~~~~CGGGAGGGTGGGAGCTG~AGTCGTAGTCTACAGTCACTCTGCTCGTCATG |  |
| PavCOL2_‘Lapins’ |  | ~~~~~~~~~~~~~~~~~~~~~~~~~~~~~~~~~~~~~~~~~~~~~~~~~~GGGGGGGGGTGTAGCTGTAGTCGTGGTCTACAGTCACTCTGCTCGTCATG |  |
| PavCOL2_‘Garnet’ |  | ~~~~~~~~~~~~~~~~~~~~~~~~~~~~~~~~~~~~~~~~~~~~~~~~CGCGGAGGGAGCATCGCTG~AGTCGTAGTCTCCAGTCACTCTGCTCGTCATG |  |
|  |  |  |  |
|  |  | ....|....|....|....|....|....|....|....|....|....|....|....|....|....|....|....|....|....|....|....| |  |
| ppa007007m |  | CTCGCCGGATAATTTTTCTGGTCATGGTAACGGCGACGGGGACACCCAGTCCTCTTCCTCGGCTTGTTCCGCCTGCGTCTCTAGCACTGATTCCTTTGGC |  |
| PRUAV007294 |  | ctcgccggataatttttctggtcatggcaacggcgacggggacacccagtcctcatcctcggcctgttccgcctgcgtctccagcactgattcctttggc |  |
| PavCOL2_‘Regina’ |  | CTCGCCGGATAATTTTTCTGGTCATGGCAACGGCGACGGGGACACCCAGTCCTCATCCTCGGCCTGTTCCGCCTGCGTCTCCAGCACTGATTCCTTTGGC |  |
| PavCOL2_‘Lapins’ |  | CTCGTCGGATAATTTTTCTGGTCATGGCAACGGCGACGGGGACACCCAGTCCTCATCCTCGGCYTGTTCCGCCTGCGTCTCCAGCACTGATTCCTTTGGC |  |
| PavCOL2_‘Garnet’ |  | CTCGTCGGATAATTTTTCTGGTCATGGCAACGGCGACGGGGACACCCAGTCCTCATCCTCGGCYTGTTCCGCCTGCGTCTCCAGCACTGATTCCTTTGGC |  |
|  |  |  |  |
|  |  | ....|....|....|....|....|....|....|....|....|....|....|....|....|....|....|....|....|....|....|....| |  |
| ppa007007m |  | GGAACTGCGGCGACGAAGGCTGGGTTTGATAACTTGAAATCTGAGAGCTCCGTAACTCAGGTTTCCGGCAAGTTGTCGAATATTCCGGCGAGATTCTCCG |  |
| PRUAV007294 |  | ggaactgcggcgacgaaggctgggttcgataaccggaaatctgagagctccgtaactcaggtttccggcaagttgtcgaatattccggcgagattctccg |  |
| PavCOL2_‘Regina’ |  | GGAACTGCGGCGACGAAGGCTGGGTTCGATAACCGGAAATCTGAGAGCTCCGTAACTCAGGTTTCCGGCAAGTTGTCGAATATTCCGGCGAGATTCTCCG |  |
| PavCOL2_‘Lapins’ |  | GGAACTGCGGCGACGAAGGCTGGGTTCGATAACCGGAAATCTGAGAGCTCCGTAACTCAGGTTTCCGGCAAGTTGTCGAATATTCCGGCGAGATTCTCCG |  |
| PavCOL2_‘Garnet’ |  | GGAACTGCGGCGACGAAGGCTGGGTTCGATAACCGGAAATCTGAGAGCTCCGTAACTCAGGTTTCCGGCAAGTTGTCGAATATTCCGGCGAGATTCTCCG |  |
|  |  |  |  |
|  |  | ....|....|....|....|....|....|....|....|....|....|....|....|....|....|....|....|....|....|....|....| |  |
| ppa007007m |  | GCGCAAAGAGGAAGTGCGTACAGAGGGCGCAAGCGCGAACTTCGACGAGCGCGGATGCAAAAGCGAAAGGTAGTTTCATAAATTGGTGCAGTCAGCTGGG |  |
| PRUAV007294 |  | gcgcaaagaggaagtacgtacagagggcgcgagcgcgaacttcgacgagcgtggatgcaaaagcgaagggtagtttcgtaaattggtgcagtaagctggg |  |
| PavCOL2_‘Regina’ |  | GCGCAAAGAGGAAGTACGTACAGAGGGCGCGAGCGCGAACTTCGACGAGCGTGGATGCAAAAGCGAAGGGTAGAA |  |
| PavCOL2_‘Lapins’ |  | GCGCAAAGAGGAAGTACGTACAGAGGGCGCGAGCGCGAACTTCGACGAGCGTGGATGCAAAAGCGAAGGGTAGAA |  |
| PavCOL2_‘Garnet’ |  | GCGCAAAGAGGAAGTGCGTACAGAGGGCGCGAGCGCGAACTTCGACGAGCGTGGATGCAAAAGCGAAGGGTAGAA |  |

***COP1***

|  |  | ....|....|....|....|....|....|....|....|....|....|....|....|....|....|....|....|....|....|....|....| |  |
| --- | --- | --- | --- |
| ppa002554m | 4851 | TTGGTGCACAAGGCAGGAAACTAGTGTTCTTGACATTGACATGAAAGCAAACATTTGTTGTGTTAAGTATAATCCTGGATCCAGCAACTGCATTGCGGTA |  |
| PRUAV007932 |  | ttggtgcacaaggcaggaaactagtgttcttgacattgacatgaaagcgaacatttgttgtgttaagtataatcctggatcaagcaactgcattgcg~~~ |  |
| PavCOP1_‘Regina’ |  | ~~~~~~~~~~~~~~~~~~~~~~~~~~~~~~~~~~~~~~~~~~~~~~~~~~~~~~~~~~~~~~~gacgggatccgGGAGATCA~GCA~CTGCATTGCGGTA |  |
| PavCOP1_‘Lapins’ |  | ~~~~~~~~~~~~~~~~~~~~~~~~~~~~~~~~~~~~~~~~~~~~~~~~~~~~~~~~~~~~~~~~~~ccaaatcctgAGATCAAGCAACTGCATTGCGGTA |  |
| PavCOP1_‘Garnet’ |  | ~~~~~~~~~~~~~~~~~~~~~~~~~~~~~~~~~~~~~~~~~~~~~~~~~~~~~~~~~~~~~~~~tagtgttATCCTGGATCA~GCA~CTGCATTGCGGTA |  |
|  |  |  |  |
|  |  | ....|....|....|....|....|....|....|....|....|....|....|....|....|....|....|....|....|....|....|....| |  |
| ppa002554m |  | TGCTTCTTTCATTACCAATATTGGAGAAAAATTATGGCACCAGCGTTTTCTGCTTTTGTGTGTGGTCGTGTTTAATACTAGATAATTTGAGTGCAACAAG |  |
| PRUAV007932 |  | ~~~~~~~~~~~~~~~~~~~~~~~~~~~~~~~~~~~~~~~~~~~~~~~~~~~~~~~~~~~~~~~~~~~~~~~~~~~~~~~~~~~~~~~~~~~~~~~~~~~~ |  |
| PavCOP1_‘Regina’ |  | TGCTCCTTTCATTACCAATATTGGAGAAAAATTATGGCACCAGAGTTTTCTGCTTTTGTGTGTTGTCATGTTTAATACCAGATAATTTGAGTGCAACAAG |  |
| PavCOP1_‘Lapins’ |  | TGCTCCTTTCATTACCAATATTGGAGAAAAATTATGGCACCAGAGTTTTCTGCTTTTGTGTGTTGTCATGTTTAATACCAGATAATTTGAGTGCAACAAG |  |
| PavCOP1_‘Garnet’ |  | TGCTCCTTTCATTACCAATATTGGAGAAAAATTATGGCACCAGAGTTTTCTGCTTTTGTGTGTTGTCATGTTTAATACCAGATAATTTGAGTGCAACAAG |  |
|  |  |  |  |
|  |  | ....|....|....|....|....|....|....|....|....|....|....|....|....|....|....|....|....|....|....|....| |  |
| ppa002554m |  | TGCTTTACTCGGTTAATACGCAGTAATATACTGTGCCTGAGAAATCATTCTTATAGTCTATGCACTGGACCAACATGCCTTCAGCAGGCATGTATATGTG |  |
| PRUAV007932 |  | ~~~~~~~~~~~~~~~~~~~~~~~~~~~~~~~~~~~~~~~~~~~~~~~~~~~~~~~~~~~~~~~~~~~~~~~~~~~~~~~~~~~~~~~~~~~~~~~~~~~~ |  |
| PavCOP1_‘Regina’ |  | TGCTTTAATCGGTTAATACACAGTAATATACTGTGCCTGAGAAATCATTCTTATGGTCTATGCACTGGACCAACATGCCTTCAGCAGGCATGTATATGTG |  |
| PavCOP1_‘Lapins’ |  | TGCTTTAATCGGTTAATACACAGTAATATACTGTGCCTGAGAAATCATTCTTATGGTCTATGCACTGGACCAACATGCCTTCAGCAGGCATGTATATGTG |  |
| PavCOP1_‘Garnet’ |  | TGCTTTAATCGGTTAATACACAGTAATATACTGTGCCTGAGAAATCATTCTTATGGTCTATGCACTGGACCAACATGCCTTCAGCAGGCATGTATATGTG |  |
|  |  |  |  |
|  |  | ....|....|....|....|....|....|....|....|....|....|....|....|....|....|....|....|....|....|....|....| |  |
| ppa002554m |  | TTAATGTGTGCATGCACTTATCAATTAAGTTTTGCCTTGTCATCCTGTTATGGGTGGGGTATGTGGTGTATAAAATA~TCATATCCAGGATATGTCCTGT |  |
| PRUAV007932 |  | ~~~~~~~~~~~~~~~~~~~~~~~~~~~~~~~~~~~~~~~~~~~~~~~~~~~~~~~~~~~~~~~~~~~~~~~~~~~~~~~~~~~~~~~~~~~~~~~~~~~~ |  |
| PavCOP1_‘Regina’ |  | TTAATGTGTGCATGCGCTTATCAATTAAGTTTTGCTTTGACATCCTGTTATGGGTGGGGTATGTGGTGTATAAAATAATCATATCCAGGATATGTCCTGT |  |
| PavCOP1_‘Lapins’ |  | TTAATGTGTGCATGCGCTTATCAATTAAGTTTTGCTTTGACATCCTGTTATGGGTGGGGTATGTGGTGTATAAAATAATCATATCCAGGATATGTCCTGT |  |
| PavCOP1_‘Garnet’ |  | TTAATGTGTGCATGCGCTTATCAATTAAGTTTTGCTTTGACATCCTGTTATGGGTGGGGTATGTGGTGTATAAAATAATCATATCCAGGATATGTCCTGT |  |
|  |  |  |  |
|  |  | ....|....|....|....|....|....|....|....|....|....|....|....|....|....|....|....|....|....|....|....| |  |
| ppa002554m |  | TAATATTTCTTGGGAACTTAGCACAGTTCAGAGTTACAGTACTGTGTTAATGATAATTTTGTATTTTTCAAATATCAAAGAGGGGCTCCTCTTTGCATCA |  |
| PRUAV007932 |  | ~~~~~~~~~~~~~~~~~~~~~~~~~~~~~~~~~~~~~~~~~~~~~~~~~~~~~~~~~~~~~~~~~~~~~~~~~~~~~~~~~~~~~~~~~~~~~~~~~~~~ |  |
| PavCOP1_‘Regina’ |  | TAATAATTCTTAGGAACTTAGCACAGTTCAGAGTTACAGTACTTTGTTAATGATAATTTTGTATTTTTCAAATATCAAAGAGGGGCTCCTCTTTGCATCA |  |
| PavCOP1_‘Lapins’ |  | TAATAATTCTTAGGAACTTAGCACAGTTCAGAGTTACAGTACTTTGTTAATGATAATTTTGTATTTTTCAAATATCAAAGAGGGGCTCCTCTTTGCATCA |  |
| PavCOP1_‘Garnet’ |  | TAATAATTCTTAGGAACTTAGCACAGTTCAGAGTTACAGTACTTTGTTAATGATAATTTTGTATTTTTCAAATATCAAAGAGGGGCTCCTCTTTGCATCA |  |
|  |  |  |  |
|  |  | ....|....|....|....|....|....|....|....|....|....|....|....|....|....|....|....|....|....|....|....| |  |
| ppa002554m |  | ATGTCAGGATGCCAATTAGTGAAAATCAGGAGGTATACCTGCCGTATGTTATCCAAACTTGTCCTAAGCATAGAAGAATATTATTTTAAAGTTTCAGTGC |  |
| PRUAV007932 |  | ~~~~~~~~~~~~~~~~~~~~~~~~~~~~~~~~~~~~~~~~~~~~~~~~~~~~~~~~~~~~~~~~~~~~~~~~~~~~~~~~~~~~~~~~~~~~~~~~~~~~ |  |
| PavCOP1_‘Regina’ |  | ATGTCAGGATGCCGATTAGTGAAAATCAGGAGGTATACCTGCCGTATGTTATCCAAACTTGTCCTGAGCATAGAAGAATATTATTTTAAAGTTTCAGTGC |  |
| PavCOP1_‘Lapins’ |  | ATGTCAGGATGCCGATTAGTGAAAATCAGGAGGTATACCTGCCGTATGTTATCCAAACTTGTCCTGAGCATAGAAGAATATTATTTTAAAGTTTCAGTGC |  |
| PavCOP1_‘Garnet’ |  | ATGTCAGGATGCCGATTAGTGAAAATCAGGAGGTATACCTGCCGTATGTTATCCAAACTTGTCCTGAGCATAGAAGAATATTATTTTAAAGTTTCAGTGC |  |
|  |  |  |  |
|  |  | ....|....|....|....|....|....|....|....|....|....|....|....|....|....|....|....|....|....|....|....| |  |
| ppa002554m |  | TAATTTTGTCTTATGATGTTTCCGTCGAATAAAGCCTCAACTCTTTCTCCTTCTGCCTTTTACAGGTTGGTTCAGCAGACCATCACATCCACTATTATGA |  |
| PRUAV007932 |  | ~~~~~~~~~~~~~~~~~~~~~~~~~~~~~~~~~~~~~~~~~~~~~~~~~~~~~~~~~~~~~~~~~gttggttcagcagaccatcacatccactattatga |  |
| PavCOP1_‘Regina’ |  | TAATTTTCTATTATGATGTTTCCGTCAAATAAAGCCTCAACTCTTTCTCCTTCTGCCTTTTACAGGTTGGTTCAGCAGACCATCACATCCACTATTATGA |  |
| PavCOP1_‘Lapins’ |  | TAATTTTCTATTATGATGTTTCCGTCAAATAAAGCCTCAACTCTTTCTCCTTCTGCCTTTTACAGGTTGGTTCAGCAGACCATCACATCCACTATTATGA |  |
| PavCOP1_‘Garnet’ |  | TAATTTTCTATTATGATGTTTCCGTCAAATAAAGCCTCAACTCTTTCTCCTTCTGCCTTTTACAGGTTGGTTCAGCAGACCATCACATCCACTATTATGA |  |
|  |  |  |  |
|  |  | ....|....|....|....|....|....|....|....|....|....|....|....|....|....|....|....|....|....|....|....| |  |
| ppa002554m |  | TTTAAGAAATCCGAGCCAGCCACTCCATGTGTTCACCGGGCACAAGAAAGCTGTTTCTTATGTGAAATTC~TTGTC~~AAACTATGAGCTTGCCTCTGCA |  |
| PRUAV007932 |  | tttaagaaatccaagccaaccactccatgtgttcaccgggcacaagaaagctgtttcttatgtaaaaatcctagtcnnaaactatgagcttgcctctgca |  |
| PavCOP1_‘Regina’ |  | TTTAAGAAATCCAAGCCAACCACTCCATGTGTTCACCGGGCACAAGAAAGCTGTTTCTTATGTGAAATTC~TTGTC~~AAACTATGAGCTTGCCCTCTGC |  |
| PavCOP1_‘Lapins’ |  | TTTAAGAAATCCAAGCCAACCACTCCATGTGTTCACCGGGCACAAGAAAGCTGTTTCTTATGTGAAATTC~TTGTC~~AAACTATGAGCTGGCCCTCTGC |  |
| PavCOP1_‘Garnet’ |  | TTTAAGAAATCCAAGCCAACCACTCCATGTGTTCACCGGGCACAAGAAAGCTGTTTCTTATGTGAAATTC~TTGTC~~AAACTATGAG~TGCCCCTTTCT |  |
|  |  |  |  |
|  |  | ....|....|....|....|....|....|....|....|....|....|....|....|....|....|....|....|....|....|....|....| |  |
| ppa002554m |  | TCCACTGATAGCACATTGCGGTTATGGAATGTGAAGGAAAATATTCCAGTAAGTGATGAAACAAAGTCTTTTTTAGTACACTTTAATAGTTAACAAGTTA |  |
| PRUAV007932 |  | tccactgatagcacactgcggttatggaatgtgaaggaaa~tnttcca~~~~~~~~~~~~~~~~~~~~~~~~~~~~~~~~~~~~~~~~~~~~~~~~~~~~ |  |
| PavCOP1_‘Regina’ |  | AA |  |
| PavCOP1_‘Lapins’ |  | AA |  |
| PavCOP1_‘Garnet’ |  | Gccaa |  |

***CRP***

|  |  | ....|....|....|....|....|....|....|....|....|....|....|....|....|....|....|....|....|....|....|....| |  |
| --- | --- | --- | --- |
| ppa000036m | 4351 | GTTCGTTGGCTGATCATGTTCCTCACGGGTATAGGAAAAGACCCCTATTTGAAGTTCTTACAAGGAATAATGTTCCATTGCTGAGAGCAACTTGGTTTAT |  |
| PRUAV078949 |  | ~~~~~~~~~~~~~~~~~~~~~~~~~~~~~~~~~~~~~~~~~~~~~~~~~~~~~~~~~~~~~~~~~~~~~~~~~~~~~~~~~~~~~~~~~~~~~~~~~~~~ |  |
| PavCRP_‘Regina’ |  | ~~~~~~~~~~~~~~~~~~~~~gcggag~~~aT~~~~~~~~~~~CCTATTTGA~GTTCTTACAAGGAATAATGTTCCATTGCTGAGAGCAACTTGGTTTAT |  |
| PavCRP_‘Lapins’ |  | ~~~~~~~~~~~~~~~~~~~~~ccGGAA~~~AT~~~~~~~~~~~CCTATTTGA~GTTCTTACAAGGAATAATGTTCCATTGCTGAGAGCAACTTGGTTTAT |  |
| PavCRP_‘Garnet’ |  | ~~~~~~~~~~~~~~~~~ggggGGAAGA~~~AT~~~~~~~~~~~CCTATTTGA~GTTCTTACAAGGAATAATGTTCCATTGCTGAGAGCAACTTGGTTTAT |  |
|  |  |  |  |
|  |  | ....|....|....|....|....|....|....|....|....|....|....|....|....|....|....|....|....|....|....|....| |  |
| ppa000036m |  | TAAAGTAACTTATCTTAATCAGGTATATATGCTCGCACATTCATAACAGAACTGAGTTCTGATCTTAACCTGTTTGTCAATACTTTTCATGTTGGAAGTT |  |
| PRUAV078949 |  | ~~~~~~~~~~~~~~~~~~~~~~~~~~~~~~~~~~~~~~~~~~~~~~~~~~~~~~~~~~~~~~~~~~~~~~~~~~~~~~~~~~~~~~~~~~~~~~~~~~~~ |  |
| PavCRP_‘Regina’ |  | TAAAGTAACTTATCTTAATCAGGTATATATGCTTGCACATTCACAACAGAACTGAGTTCTGACCTTAACCTGTTTGTCAATACTTTTCATGTTGGAAGTT |  |
| PavCRP_‘Lapins’ |  | TAAAGTAACTTATCTTAATCAGGTATATATGCTTGCACATTCACAACAGAACTGAGTTCTGACCTTAACCTGTTTGTCAATACTTTTCATGTTGGAAGTT |  |
| PavCRP_‘Garnet’ |  | TAAAGTAACTTATCTTAATCAGGTATATATGCTTGCACATTCACAACAGAACTGAGTTCTGACCTTAACCTGTTTGTCAATACTTTTCATGTTGGAAGTT |  |
|  |  |  |  |
|  |  | ....|....|....|....|....|....|....|....|....|....|....|....|....|....|....|....|....|....|....|....| |  |
| ppa000036m |  | TCCAGTAGCATGTTATTGCTGCATTTTTTGCAGGTCAGGCCAGGTTCTGCCATTATTTCTTCTGGGGCACCTGATAAAGCTCAGTTGTCCCGCACAGAGC |  |
| PRUAV078949 |  | ~~~~~~~~~~~~~~~~~~~~~~~~~~~~~~~~~~~~~~~~~~~~~~~~~~~~~~~~~~~~~~~~~~~~~~~~~~~~~~~~~~~~~~~~~~~~~~~~~~~~ |  |
| PavCRP_‘Regina’ |  | TCCAGTAGCATGTTATTGCTGCATTTTTTGCAGGTCAAGCCAGGTTCTGCCATTATTTCTTCTGGGGCACCTGATAAAGCTCAGTTGTCCCGCACAGAGC |  |
| PavCRP_‘Lapins’ |  | TCCAGTAGCATGTTATTGCTGCATTTTTTGCAGGTCAAGCCAGGTTCTGCCATTATTTCTTCTGGGGCACCTGATAAAGCTCAGTTGTCCCGCACAGAGC |  |
| PavCRP_‘Garnet’ |  | TCCAGTAGCATGTTATTGCTGCATTTTTTGCAGGTCAAGCCAGGTTCTGCCATTATTTCTTCTGGGGCACCTGATAAAGCTCAGTTGTCCCGCACAGAGC |  |
|  |  |  |  |
|  |  | ....|....|....|....|....|....|....|....|....|....|....|....|....|....|....|....|....|....|....|....| |  |
| ppa000036m |  | TCTGGACAAAAGATGTTATCGATTACCTGCAGTACCTTCTCGATGAGTTATTCTCGAGAAATAATTCTCATTCTACTTCACACAACAGAGATCGTTCGCC |  |
| PRUAV078949 |  | ~~~~~~~~~~~~~~~~~~~~~~~~~~~~~~~~~~~~~~~~~~~~~~~~~~~~~~CGAGAAATAATTCTCATTCTACTTCACACAACAGAGATCGTTCGCC |  |
| PavCRP_‘Regina’ |  | TCTGGACAAAAGATGTTATCGATTACCTGCAGTACCTTCTCGATGAGTTATTCTCGAGAAATAATTCTCATTCTACTTCACACAACAGAGATCGTTCGCC |  |
| PavCRP_‘Lapins’ |  | TCTGGACAAAAGATGTTATCGATTACCTGCAGTACCTTCTCGATGAGTTATTCTCGAGAAATAATTCTCATTCTACTTCACACAACAGAGATCGTTCGCC |  |
| PavCRP_‘Garnet’ |  | TCTGGACAAAAGATGTTATCGATTACCTGCAGTACCTTCTCGATGAGTTATTCTCGAGAAATAATTCTCATTCTACTTCACACAACAGAGATCGTTCGCC |  |
|  |  |  |  |
|  |  | ....|....|....|....|....|....|....|....|....|....|....|....|....|....|....|....|....|....|....|....| |  |
| ppa000036m |  | ACAAACGCTTTATGCTGGGTCAGTTCCACAAAGGAGTGATCCAGCATCAGCAGTCCCTGACGGTGAGGAGCCTTCCCTACATTTCAAGTGGTGGTATGTG |  |
| PRUAV078949 |  | ACAAACGGTTTATGCTGGGTCAGTTCCACAAAGGAGTGATCCAGCATCAGCAGTCCTTGACGGTGAGGAGCCTTCCCTACATTTCAAGTGGTGGTATGTG |  |
| PavCRP_‘Regina’ |  | ACAAACGGTTTATGCTGGGTCAGTTCCACAAAGGAGTGATCCAGCATCAGCAGTCCTTGACGGTGAGGAGCCTTCCCTACATTTCAAGTGGTGG~ATGGG |  |
| PavCRP_‘Lapins’ |  | ACAAACGGTTTATGCTGGGTCAGTTCCACAAAGGAGTGATCCAGCATCAGCAGTCCTTGACGGTGAGGAGCCTTCCCTACATTTCAAGTGGTGGT~TGGT |  |
| PavCRP_‘Garnet’ |  | ACAAACGGTTTATGCTGGGTCAGTTCCACAAAGGAGTGATCCAGCATCAGCAGTCCTTGACGGTGAGGAGCCTTCCCTACATTTCAAGTGGTG~TA~GGG |  |
|  |  |  |  |
|  |  | ....|....|....|....|....|....|....|....|....|....|....|....|....|....|....|....|....|....|....|....| |  |
| ppa000036m |  | GTGCGGCTCTTGCAATGGCATCATGCCGAGGGGCTGCTTCTTCCTACTCT |  |
| PRUAV078949 |  | GTGCGGCTCTTGCAATGGCATCATGCCGAGGGGCTGCTTCTTCCTACTCT |  |
| PavCRP_‘Regina’ |  | TGGTGCA |  |
| PavCRP_‘Lapins’ |  | TGGTGCA |  |
| PavCRP_‘Garnet’ |  | GGGGTGCA |  |

***DAM5***

|  |  | ....|....|....|....|....|....|....|....|....|....|....|....|....|....|....|....|....|....|....|....| |  |
| --- | --- | --- | --- |
| ppa010822m | 6451 | TTTATCATTTTAGCTGGAGAATGAAAACCACATCAAACTGAGTAAGGAACTCGAGGAGAAGAGCCGCCAGCTGAGGTACAAAATTCAT~~GATC~TTGTA |  |
| PRUAV009602 |  | ~~~~~~~~~~~~~CTGGAGCATGAAAACCACATCAAACTGAGTAAGGAACTCGAGGAGAAGAGCCGCCAGCTGAG~~~~~~~~~~~~~~~~~~~~~~~~~ |  |
| PavDAM5_‘Regina’ |  | ~~~~~~~~~~~~~~~~~~~~~~~~~~~~~~~~~~~~~~~~~~~~~~~~~GGACGTATATCGTCAGCATGTATGCACT~CAG~~TTCAGTAGAACATTGTA |  |
| PavDAM5_‘Lapins’ |  | ~~~~~~~~~~~~~~~~~~~~~~~~~~~~~~~~~~~~~~~~~~~~~~~~~~~~GGGGAAAGACGCAGCACTAGGTACT~CAA~~TTCA~TAGAAC~TTGTA |  |
| PavDAM5_‘Garnet’ |  | ~~~~~~~~~~~~~~~~~~~~~~~~~~~~~~~~~~~~~~~~~~~~~~~~~~~~~~GAGGATCGTCAGCTGTAGGTAG~~~AAA~TTCA~T~GAAC~TTGTA |  |
|  |  |  |  |
|  |  | ....|....|....|....|....|....|....|....|....|....|....|....|....|....|....|....|....|....|....|....| |  |
| ppa010822m |  | AGATATATGTGGCTGTTTAGCTGGTATTGTTGTTTGATGTATCTGCTGAAATTGGACGGGCTTGCAACAGGCAGATGAAAGGTGAGGATCTTGAAGGGCT |  |
| PRUAV009602 |  | ~~~~~~~~~~~~~~~~~~~~~~~~~~~~~~~~~~~~~~~~~~~~~~~~~~~~~~~~~~~~~~~~~~~~~~GCAGATGAAAGGTGATGATCTTGAAGGGCT |  |
| PavDAM5_‘Regina’ |  | AGATATTTGTGGCTGTTTAGTTGGTATTATTGTTTGATGTATCTGCTGAAATTGGACTGGCTTGCAACAGGCAGATGAAAGGTGATGATCTTGAAGGGCT |  |
| PavDAM5_‘Lapins’ |  | AGATATTTGTGGCTGTTTAGTTGGTATTATTGTTTGATGTATCTGCTGAAATTGGACTGGCTTGCAACAGGCAGATGAAAGGTGATGATCTTGAAGGGCT |  |
| PavDAM5_‘Garnet’ |  | AGATATTTGTGGCTGTTTAGTTGGTATTATTGTTTGATGTATCTGCTGAAATTGGACTGGCTTGCAACAGGCAGATGAAAGGTGATGATCTTGAAGGGCT |  |
|  |  |  |  |
|  |  | ....|....|....|....|....|....|....|....|....|....|....|....|....|....|....|....|....|....|....|....| |  |
| ppa010822m |  | GAATCTGGATGAGTTGTTGAAGTTGGAACAACTGGTGGAAGCAAGCCTTGGCCGTGTCATGGAAACTAAGGTCTGCCCTAGCTATTTTATTTTCTAGTAA |  |
| PRUAV009602 |  | GAATCTGGATGAGTTGCTGAAGTTGGAACAACTGGTGGAAGCAAGCCTTGGCCGTGTCATGGAAACTAAG~~~~~~~~~~~~~~~~~~~~~~~~~~~~~~ |  |
| PavDAM5_‘Regina’ |  | GAATCTGGATGAGTTGCTGAAGTTGGAACAACTGGTGGAAGCAAGCCTTGGCCGTGTCATGGAAACTAAGGTCTGCCCTAGCTAATTTATTTTCTAGTAA |  |
| PavDAM5_‘Lapins’ |  | GAATCTGGATGAGTTGCTGAAGTTGGAACAACTGGTGGAAGCAAGCCTTGGCCGTGTCATGGAAACTAAGGTCTGCCCTAGCTAATTTATTTTCTAGTAA |  |
| PavDAM5_‘Garnet’ |  | GAATCTGGATGAGTTGCTGAAGTTGGAACAACTGGTGGAAGCAAGCCTTGGCCGTGTCATGGAAACTAAGGTCTGCCCTAGCTAATTTATTTTCTAGTAA |  |
|  |  |  |  |
|  |  | ....|....|....|....|....|....|....|....|....|....|....|....|....|....|....|....|....|....|....|....| |  |
| ppa010822m |  | CCA~~GTGC~~~~~~~~~~~~~~~~~~~~~~~~~~~~~~~~~~~~~~~~~~~~~~~~~~~~~~~~~~~~~~~~~~~~~~~~~~~~~~~~~~~~~~~~~~~ |  |
| PRUAV009602 |  | ~~~~~~~~~~~~~~~~~~~~~~~~~~~~~~~~~~~~~~~~~~~~~~~~~~~~~~~~~~~~~~~~~~~~~~~~~~~~~~~~~~~~~~~~~~~~~~~~~~~~ |  |
| PavDAM5_‘Regina’ |  | GCATGGTGCGAAGGCTCTTTGTATTGTGGTTTAATTGTCGAAATCGTTTATTATTTAAATCTCAAATTAGGGATCCTTGTTGCATAAAGTTAGTTAAATT |  |
| PavDAM5_‘Lapins’ |  | GCATGGTGCGAAGGCTCTTTGTATTGTGGTTTAATTGTCGAAATCGTTTATTATTTAAATCTCAAATTAGGGATCCTTGTTGCATAAAGTTAGTTAAATT |  |
| PavDAM5_‘Garnet’ |  | GCATGGTGCGAAGGCTCTTTGTATTGTGGTTTAATTGTCGAAATCGTTTATTATTTAAATCTCAAATTAGGGATCCTTGTTGCATAAAGTTAGTTAAATT |  |
|  |  |  |  |
|  |  | ....|....|....|....|....|....|....|....|....|....|....|....|....|....|....|....|....|....|....|....| |  |
| ppa010822m |  | ~~~~~~~~~~~~~~~~~CCTAGCTTCTTCTTTTTCTTTTTCTTTTTTTTTCTTTTCTGGATGATTCTTGACTGAAGACATAAGCAATGAACCGTTAGAAT |  |
| PRUAV009602 |  | ~~~~~~~~~~~~~~~~~~~~~~~~~~~~~~~~~~~~~~~~~~~~~~~~~~~~~~~~~~~~~~~~~~~~~~~~~~~~~~~~~~~~~~~~~~~~~~~~~~~~ |  |
| PavDAM5_‘Regina’ |  | GTTTAAACGAAATGGTCAATTGGTCTCCGATAATTTTTATTTTTTATTTGTTTTTCTGGATGATTCTTGAATGAAAACATAAGCAATGAACCGGTCCAAT |  |
| PavDAM5_‘Lapins’ |  | GTTTAAACGAAATGGTCAATTGGTCTCCGATAATTTTTATTTTTTATTTGTTTTTCTGGATGATTCTTGAATGAAAACATAAGCAATGAACCGGTCCAAT |  |
| PavDAM5_‘Garnet’ |  | GTTTAAACGAAATGGTCAATTGGTCTCCGATAATTTTTATTTTTTATTTGTTTTTCTGGATGATTCTTGAATGAAAACATAAGCAATGAACCGGTCCAAT |  |
|  |  |  |  |
|  |  | ....|....|....|....|....|....|....|....|....|....|....|....|....|....|....|....|....|....|....|....| |  |
| ppa010822m |  | AATATAAAGTAGGTCTTTGTTTGAAGAAGTATCGTCTCAGTAAATAGGGTATATTTTTATTGCTGTATTTTGTGAAAAGCATAGGTGTATTC~~~~~TGA |  |
| PRUAV009602 |  | ~~~~~~~~~~~~~~~~~~~~~~~~~~~~~~~~~~~~~~~~~~~~~~~~~~~~~~~~~~~~~~~~~~~~~~~~~~~~~~~~~~~~~~~~~~~~~~~~~~~~ |  |
| PavDAM5_‘Regina’ |  | AATATAAAGTAGGTCTTTGTTTGAAGAAGTGTCGTTTGTGTAAATAGGGTATATTTTTATTGCTGTATTTTGTGAAAAGCATAGTTGTATTCTGGGATGA |  |
| PavDAM5_‘Lapins’ |  | AATATAAAGTAGGTCTTTGTTTGAAGAAGTGTCGTTTGTGTAAATAGGGTATATTTTTATTGCTGTATTTTGTGAAAAGCATAGTTGTATTCTGGGATGA |  |
| PavDAM5_‘Garnet’ |  | AATATAAAGTAGGTCTTTGTTTGAAGAAGTGTCGTTTGTGTAAATAGGGTATATTTTTATTGCTGTATTTTGTGAAAAGCATAGTTGTATTCTGGGTTGA |  |
|  |  |  |  |
|  |  | ....|....|....|....|....|....|....|....|....|....|....|....|....|....|....|....|....|....|....|....| |  |
| ppa010822m |  | G~~~~~~~~~~~~~~~~~~~~~~~~~~~~~~~~~~~~~~~~~~~~~~~~~~~~~~~~~~~~~~~~~~~~~~~~~~~~~~~~~~~~~~~~~~~~~~~~~~~ |  |
| PRUAV009602 |  | ~~~~~~~~~~~~~~~~~~~~~~~~~~~~~~~~~~~~~~~~~~~~~~~~~~~~~~~~~~~~~~~~~~~~~~~~~~~~~~~~~~~~~~~~~~~~~~~~~~~~ |  |
| PavDAM5_‘Regina’ |  | GGTCTTAAATATCGGGATAATATCGATATATTGACGATAACACTATGGTTAGTTGTTAAAATATCGGACCCCTAAAAATTAGATATTATCGGGATAATAT |  |
| PavDAM5_‘Lapins’ |  | GGTCTTAAATATCGGGATAATATCGATATATTGACGATAACACTATGGTTAGTTGTTAAAATATCGGACCCCTAAAAATTAGATATTATCGGGATAATAT |  |
| PavDAM5_‘Garnet’ |  | GGTCTTAAATATCGGGATAATATCGATATATTGACGATAACACTATGGTTAGTTGTTAAAATATCGGACCCCTAAAAATTAGATATTATCGGGATAATAT |  |
|  |  |  |  |
|  |  | ....|....|....|....|....|....|....|....|....|....|....|....|....|....|....|....|....|....|....|....| |  |
| ppa010822m |  | ~~~~~~~~~~~~~~~~~~~~~~~~~~~TTGTCACTAAGCTTTATTTTCTGAAAACTAACATCTAAATGC~~~TGTTCCTTTCTTTTCCAGGAAGAGCTGA |  |
| PRUAV009602 |  | ~~~~~~~~~~~~~~~~~~~~~~~~~~~~~~~~~~~~~~~~~~~~~~~~~~~~~~~~~~~~~~~~~~~~~~~~~~~~~~~~~~~~~~~~~~GAAGAGCTGA |  |
| PavDAM5_‘Regina’ |  | CGGGGATATTATCGATATTTAATACCTTTGTCACTGAGCTTTATTTTCTGAAAACTAACATCTAAATGCAACTGTTTCATTCTTTTCCAGGAAGAGCTGA |  |
| PavDAM5_‘Lapins’ |  | CGGGGATATTATCGATATTTAATACCTTTGTCACTGAGCTTTATTTTCTGAAAACTAACATCTAAATGCAACTGTTTCATTCTTTTCCAGGAAGAGCTGA |  |
| PavDAM5_‘Garnet’ |  | CGGGGATATTATCGATATTTAATACCTTTGTCACTGAGCTTTATTTTCTGAAAACTAACATCTAAATGCAACTGTTTCATTCTTTTCCAGGAAGAGCTGA |  |
|  |  |  |  |
|  |  | ....|....|....|....|....|....|....|....|....|....|....|....|....|....|....|....|....|....|....|....| |  |
| ppa010822m |  | TTAAGAGTGAGATTATGGCACTTGAAAGAAAGGTTAGATGATTGATACGTACAGATCTGTAAATGGAAACAAACGTAAAATAATCACGACTTAAAAACAT |  |
| PRUAV009602 |  | TTAAGAGTGAGATTATGGCACTTGAAAGAAAG~~~~~~~~~~~~~~~~~~~~~~~~~~~~~~~~~~~~~~~~~~~~~~~~~~~~~~~~~~~~~~~~~~~~ |  |
| PavDAM5_‘Regina’ |  | TTAAGAGTGAGATTATGGCACTTGAAAGAAAGGTTAGATGATTGATACGTACAGATCTGTAAATGGAAACAAACGTAAAATAATCACGACTTAAAAAC~~ |  |
| PavDAM5_‘Lapins’ |  | TTAAGAGTGAGATTATGGCACTTGAAAGAAAGGTTAGATGATTGATACGTACAGATCTGTAAATGGAAACAAACGTAAAATAATCACGACTTAAAAAC~~ |  |
| PavDAM5_‘Garnet’ |  | TTAAGAGTGAGATTATGGCACTTGAAAGAAAGGTTAGATGATTGATACGTACAGATCTGTAAATGGAAACAAACGTAAAATAATCACGACTTAAAAAC~~ |  |
|  |  |  |  |
|  |  | ....|....|....|....|....|....|....|....|....|....|....|....|....|....|....|....|....|....|....|....| |  |
| ppa010822m |  | ATATAAATTAACATCAATAATCATAACCAAGTTTGGCTTTGGTTCTCTCTTTCATTCACTGACTATAATTTCAGGGAACTGAGCTAGTTGAAGCTAACAA |  |
| PRUAV009602 |  | ~~~~~~~~~~~~~~~~~~~~~~~~~~~~~~~~~~~~~~~~~~~~~~~~~~~~~~~~~~~~~~~~~~~~~~~~~~GGAGCTGAGCTAGTTGAAGCCAACAA |  |
| PavDAM5_‘Regina’ |  | ~~~~~~~~~~~~~~~~~~~~~~~~~~~CGAGTTTGGCTTTGGTTCTCTCTTTCATGCACTGACTATAATTTCAGGGAGCTGAGCTAGTTGAAGCCAACAA |  |
| PavDAM5_‘Lapins’ |  | ~~~~~~~~~~~~~~~~~~~~~~~~~~~CGAGTTTGGCTTTGGTTCTCTCTTTCATGCACTGACTATAATTTCAGG~AGCTGAGCTAGTTGAAGTCCACA~ |  |
| PavDAM5_‘Garnet’ |  | ~~~~~~~~~~~~~~~~~~~~~~~~~~~CGAGTTTGGCTTTGGTTCTCTCTTTCATGCACTGACTATAATTTCAGG~AGCTGAGCTAGTTGAAGCC~ACA~ |  |
|  |  |  |  |
|  |  | ....|....|....|....|....|....|....|....|....|....|....|....|....|....|....|....|....|....|....|....| |  |
| ppa010822m |  | CCAGC~~~~TA~A~~GGCAGACGGTAAGCAACTACCACAATCATGTACATACTTCTTCTTACTTTTTCTTCTCCTCTTTTTGTTTTTTGAGAGGAAGAGT |  |
| PRUAV009602 |  | CCAGC~~~~TA~A~~GGCAGACG~~~~~~~~~~~~~~~~~~~~~~~~~~~~~~~~~~~~~~~~~~~~~~~~~~~~~~~~~~~~~~~~~~~~~~~~~~~~~ |  |
| PavDAM5_‘Regina’ |  | C~AGCCC~~TATAG~GGCAGGA |  |
| PavDAM5_‘Lapins’ |  | C~AGCCCCATA~AG~GGCAGA |  |
| PavDAM5_‘Garnet’ |  | C~~GCCCTCTATAGGGGC~GAAA |  |

***DAM6***

|  |  | ....|....|....|....|....|....|....|....|....|....|....|....|....|....|....|....|....|....|....|....| |  |
| --- | --- | --- | --- |
| ppa010714m | 6101 | GTATTTTTTGCATAGCAGATGGCGATGTTATCCGGAGGAAATACTGGACCTGCGTTTGTGGAGCCGGAGACGTTGATTACTAATGTTGG~~AGGTGGAGG |  |
| PRUAV027249 |  | GTATTTTTCGCATAGCAGATGGCGATGTTATCCGGAGGAAATACTGGACCTGCGCTTGTGGAGCCGGAGACGTTGAATACTAATATTGG~~AGGTGGAGG |  |
| PavDAM6_‘Regina’ |  | ~~~~~~~~~~~~~~~~~~~~~~~~~~~~~~~~~~~~~~~~~~~~~~~~~~~~~~~~~~~~~~~~~~~~ggggatgat~~cta~taTTGG~~AGGTGGAGG |  |
| PavDAM6_‘Lapins’ |  | ~~~~~~~~~~~~~~~~~~~~~~~~~~~~~~~~~~~~~~~~~~~~~~~~~~~~~~~~~~~~~~~~~~~gggggatgat~~cta~tattgggaaggtggAGG |  |
| PavDAM6_‘Garnet’ |  | ~~~~~~~~~~~~~~~~~~~~~~~~~~~~~~~~~~~~~~~~~~~~~~~~~~~~~~~~~~~~~~~~~~~gggggatga~~~cta~tattgg~~aggtgGAGG |  |
|  |  |  |  |
|  |  | ....|....|....|....|....|....|....|....|....|....|....|....|....|....|....|....|....|....|....|....| |  |
| ppa010714m |  | AGAAGAAGACGGCATGTCATCTGAATCTGCCATAATTGCCACCTCCACCAGCTGCAACAGTGCTCACAGTCTCTCTCTTGAAGATGACTGCTCCGATGTC |  |
| PRUAV027249 |  | AGAAGA~~~CGGCATGTCATCTGAATCTGCCACAATGGCCACCTCTACCAGCTGCAATAGTGCTCTCAGTCTCTCTCTTGAAGATGACTGCTCCGATGTC |  |
| PavDAM6_‘Regina’ |  | AGAA~~~GACGGCATGTCATCTGAATCTGCCACAATTGCCACCTCCACCACCTGCAACAA |  |
| PavDAM6_‘Lapins’ |  | AGAA~~~GACGGCATGTCATCTGAATCTGCCACAATTGCCACCTCCACCACCTGCAACAA |  |
| PavDAM6_‘Garnet’ |  | AGA~~~~GACGGCATGTCATCTGAATCTGCCACAATTGCCACCTCCACCACCTGCAACAA |  |

***DDF1***

|  |  | ....|....|....|....|....|....|....|....|....|....|....|....|....|....|....|....|....|....|....|....| |  |
| --- | --- | --- | --- |
| ppa021197m | 1 | ATGAACAGGTTCTTCTCTCATTTTTCTGACTCCGTGGACCAGCCCGAGTCAAGTTCGTTGTCCGACGCCACAGTCACGACTCTAAGGGCTTCTTGGTCCG |  |
| PRUAV084930 |  | ~~~~~~~~~~~~~~~~~~~~~~~~~~~~~~~~~~~~~~~~~~~~~~~~~~~~~~~~~~~~~~~~~~~~~~~~~~~~~~~~~~~~~~~~~~~~~~~~~~~~ |  |
| PavDDF1_‘Regina’ |  | ~~~~~~~~~~~~~~~~~~~~~~~~~~~~~~~~~~~~~~~~~~~~~~~~~~~~~~~~~~~~~~~~~~~~~~~~~~~~~~~~~~~~~~ggc~~~~gggt~cg |  |
| PavDDF1_‘Lapins’ |  | ~~~~~~~~~~~~~~~~~~~~~~~~~~~~~~~~~~~~~~~~~~~~~~~~~~~~~~~~~~~~~~~~~~~~~~~~~~~~~~~~~~~~~~gg~~~~~~gg~~cG |  |
| PavDDF1_‘Garnet’ |  | ~~~~~~~~~~~~~~~~~~~~~~~~~~~~~~~~~~~~~~~~~~~~~~~~~~~~~~~~~~~~~~~~~~~~~~~~~~~~~~~~~~~~~~~~~~~~~~~~t~~~ |  |
|  |  |  |  |
|  |  | ....|....|....|....|....|....|....|....|....|....|....|....|....|....|....|....|....|....|....|....| |  |
| ppa021197m |  | ACGAGGACGTCATATTG~GCGTCGAGCCGACCAAAGAAGCGAGCTGGGAGGAGGGTTTTCAAGGAGACCAGGCACCCTGTTTATAGGGGCGTGAGGAGGA |  |
| PRUAV084930 |  | ~~~~~~~~~~~~~~~~~~~~~~~~~~~~~~~~~~~~~~~~~~~~~~~~AGGAAGGTTTTCAAGGAGACGAGGCACCCGGTTTACAGGGGCGTGAGGAGAA |  |
| PavDDF1_‘Regina’ |  | A~GATGTCGTCATATTG~GCGTCGAGCAGACCAAAGAAGCGAGCTGGGAGGAGGGTTTTCAAGGAGACCAGGCACCCTGTTTATAGGGRCGTGAGGAGGA |  |
| PavDDF1_‘Lapins’ |  | ~~GA~GTCGTCATATTG~GCGTCGAGCAGACCAAAGAAGCGAGCTGGGAGGAGGGTTTTCAAGGAGACCAGGCACCCTGTTTATAGGGGCGTGAGGAGGA |  |
| PavDDF1_‘Garnet’ |  | agga~~~cgtcatattgcgcgtcgAGCAGACCAAAGAAGCGAGCTGGGAGGAGGGTTTTCAAGGAGACCAGGCACCCTGTTTATAGGGGCGTGAGGAGGA |  |
|  |  |  |  |
|  |  | ....|....|....|....|....|....|....|....|....|....|....|....|....|....|....|....|....|....|....|....| |  |
| ppa021197m |  | GGAACAATGACAAGTGGGTGTGTGAAATGAGAGAGCCCAAGAAGACGAAGTCCAGGATATGGCTCGGGACTTATCCGACGGCGGAGATGGCTGCTCGTGC |  |
| PRUAV084930 |  | GGAACAACAACAAGTGGGTGTGTGAGTTGAGAGAGCCCAACAAGAAGAAGTCAAGGGTTTGGCTCGGGACGTATCCAACGGCTGAGATGGCTGCTCGTGC |  |
| PavDDF1_‘Regina’ |  | GGAACAATGACAAGTGGGTGTGTGAAATGAGAGAGCCCAAGAAGACGAAGTCCAGGATATGGCTCGGGAGTTATCCGACGGCGGAGATGGCTGCTCGTGC |  |
| PavDDF1_‘Lapins’ |  | GGAACAATGACAAGTGGGTGTGTGAAATGAGAGAGCCCAAGAAGACGAAGTCCAGGATATGGCTCGGGAGTTATCCGACGGCGGAGATGGCTGCTCGTGC |  |
| PavDDF1_‘Garnet’ |  | GGAACAATGACAAGTGGGTGTGTGAAATGAGAGAGCCCAAGAAGACGAAGTCCAGGATATGGCTCGGGAGTTATCCGACGGCGGAGATGGCTGCTCGTGC |  |
|  |  |  |  |
|  |  | ....|....|....|....|....|....|....|....|....|....|....|....|....|....|....|....|....|....|....|....| |  |
| ppa021197m |  | CCATGACGTGGCGGCATTGGCGTTTAGAGGGAAGCTTGCCTGCCTCAACTTCCCTGACTCCGCTTGGAGGCTGCCCGTGCCGGCTTCCATGGATGCAATG |  |
| PRUAV084930 |  | CCATGACGTGGCGGCATTGGCGTTCAGAGGGAAGCTTGCCTGCATAAACTTTGCTGACTCCGCATGG~CGCTGCCCGTGCCGGCTTCCATGGATACCATG |  |
| PavDDF1_‘Regina’ |  | CCATGACGTGGCGGAATTGGCGTTTAGAGGGAAGCTTGCCTGCCTCAACTTCGCTGACTCCGCGTGGAGGCTGCCCGTGCCTGCTTCCATGGATGCAATG |  |
| PavDDF1_‘Lapins’ |  | CCATGACGTGGCGGAATTGGCGTTTAGAGGGAAGCTTGCCTGCCTCAACTTCGCTGACTCCGCGTGGAGGCTGCCCGTGCCTGCTTCCATGGATGCAATG |  |
| PavDDF1_‘Garnet’ |  | CCATGACGTGGCGGAATTGGCGTTTAGAGGGAAGCTTGCCTGCCTCAACTTCGCTGACTCCGCGTGGAGGCTGCCCGTGCCTGCTTCCATGGATGCAATG |  |
|  |  |  |  |
|  |  | ....|....|....|....|....|....|....|....|....|....|....|....|....|....|....|....|....|....|....|....| |  |
| ppa021197m |  | GATATTCGGAGAGCAGCCGCCGAGGCAGCTGAGGGGTTTAGGCCGGTGGAGTTTGGTGGAGTGTCCAGCAGCAGCAGTGATGAGAAGGAGAGAATGGTGG |  |
| PRUAV084930 |  | GATATCCGAAGGGCAGCTGCTGAGGCGGCGAAGG |  |
| PavDDF1_‘Regina’ |  | GATATTCGGAGAGCGGCCGCGGAGGCAGCTGAGGGGTTTAGGCCGGTGGAGTTTGGTGGAGTGTCCAGCAGCAGCAGTGATGAGAAGGAGAGAATGATGG |  |
| PavDDF1_‘Lapins’ |  | GATATTCGGAGAGCGGCCGCGGAGGCAGCTGAGGGGTTTAGGCCGGTGGAGTTTGGTGGAGTGTCCAGCAGCAGCAGTGATGAGAAGGAGAGAATGATGG |  |
| PavDDF1_‘Garnet’ |  | GATATTCGGAGAGCGGCCGCGGAGGCAGCTGAGGGGTTTAGGCCGGTGGAGTTTGGTGGAGTGTCCAGCAGCAGCAGTGATGAGAAGGAGAGAATGATGG |  |
|  |  |  |  |
|  |  | ....|....|....|....|....|....|....|....|....|....|....|....|....|....|....|....|....|....|....|....| |  |
| ppa021197m |  | TGCAGGTGGAAGAGAAGAAGAAGAAGAAGGATAGTGTGAAAATGGAAAAAAGTAGAAGCTTGAGCTTGTCCTATTGGGATGAGGAAGAAGTGTTTGACAT |  |
| PRUAV084930 |  |  |  |
| PavDDF1_‘Regina’ |  | TGCAGGAGGAAGAGAAGAAGAAGAAG~~~GTTAGTGTGAAAATGGAAAAAAGCAGAAGCTTGACCTCGTCCTATTGGGATGAGGAGGAAGTGTTTGACAT |  |
| PavDDF1_‘Lapins’ |  | TGCAGGAGGAAGAGAAGAAGAAGAAG~~~GTTAGTGTGAAAATGGAAAAAAGCAGAAGCTTGACCTCGTCCTATTGGGATGAGGAGGAAGTGTTTGACAT |  |
| PavDDF1_‘Garnet’ |  | TGCAGGAGGAAGAGAAGAAGAAGAAG~~~GTTAGTGTGAAAATGGAAAAAAGCAGAAGCTTGACCTCGTCCTATTGGGATGAGGAGGAAGTGTTTGACAT |  |
|  |  |  |  |
|  |  | ....|....|....|....|....|....|....|....|....|....|....|....|....|....|....|....|....|....|....|....| |  |
| ppa021197m |  | GCCAAGGTTGCTTGATGACATGGCTCAAGGCCTTCTTCTTACTCCACCTCAATG~CTTAGGTGGCGACAT~~~TTGGGATGACATGGGAACCGATGCTGA |  |
| PRUAV084930 |  |  |  |
| PavDDF1_‘Regina’ |  | GCCAAGGTTGCTTGATGACATGGCTCAAGGCCTGCTTCTTTCTCCACCTCA~TGGCTTAATGGGTTGCGatgactgggaagacatggcagcatgacgcaa |  |
| PavDDF1_‘Lapins’ |  | GCCAAGGTTGCTTGATGACATGGCTCAAGGCCTGCTTCTTTCTCCACCTCAATG~CTTAGGTGGCTACATGAATTGGGATGACATGGGAACCGATG~TGA |  |
| PavDDF1_‘Garnet’ |  | GCCAAGGTTGCTTGATGACATGGCTCAAGGCCTGCTTCTTTCTCCACCTCAATG~CTTAGGTGGCTACATGAATTGGGATGACATGGGAACCGATGCTGA |  |
|  |  |  |  |
|  |  | ....|....|....|....|....|....|....|....|....|....|....|....|....|....|....|....|....|....|....|....| |  |
| ppa021197m |  | TGTCAAATTGTGGAGTTTCTCCAATTAA |  |
| PRUAV084930 |  |  |  |
| PavDDF1_‘Regina’ |  | aatgtcaaaa |  |
| PavDDF1_‘Lapins’ |  | AAATGGTCAAAA |  |
| PavDDF1_‘Garnet’ |  | TGTCAAAA |  |

***DDL***

|  |  | ....|....|....|....|....|....|....|....|....|....|....|....|....|....|....|....|....|....|....|....| |  |
| --- | --- | --- | --- |
| ppa005178m | 80 | AGGAGCCCATCTAGGAAAAGTCCATCCAGAATAGAAAGATCACCTACTCGACATAGGAGGTCCCACAGGGGTAGTTCTCCACCAAGAGAGAAACATTCAG |  |
| PRUAV004162 |  | aggagcccatctaggaaaagtccatccagaatagaaagatcacctactcgacataggaggtcccacaggggtagttctccaccaagagagaaacattcgg |  |
| PavDDL_‘Regina’ |  | ~~~~~~~~~~~~~~~~~~~~~~~~~~~~~~~~~~~~~~~~~~~~~~~~~~~~~~~~~~~~~~~~~~~~~~~~~~~~~~~~~~~~~~~~~~~aa~~~~~gg |  |
| PavDDL_‘Lapins’ |  | ~~~~~~~~~~~~~~~~~~~~~~~~~~~~~~~~~~~~~~~~~~~~~~~~~~~~~~~~~~~~~~~~~~~~~~~~~~~~~~~~~~~~~~~~~~~aa~~~~~GG |  |
| PavDDL_‘Garnet’ |  | ~~~~~~~~~~~~~~~~~~~~~~~~~~~~~~~~~~~~~~~~~~~~~~~~~~~~~~~~~~~~~~~~~~~~~~~~~~~~~~~~~~~~~~~~~~~aa~~~~~~G |  |
|  |  |  |  |
|  |  | ....|....|....|....|....|....|....|....|....|....|....|....|....|....|....|....|....|....|....|....| |  |
| ppa005178m |  | GTCACCCTAAGTCTCCAAAGCATGCAAGGTCCCCCTCTCCTCCTGTTCGCTCTCCTTCTCCTCGGACAAAACGGTTAAGAAGAGCTCAAGCTGGCAGAGA |  |
| PRUAV004162 |  | gtcaccctaagtctccaaagcatgcaaggtccccttctcctcctgctcgctctccttctcctcggacaaaacggttaagaagagctcaagctggcagaga |  |
| PavDDL_‘Regina’ |  | TT~A~CCTA~GTCTCCAA~GCATGCAAGGTCCCCTTCTCCTCCTGCTCGCTCTCCTTCTCCTCGGACAAAACGGTTAAGAAGAGCTCAAGCTGGCAGAGA |  |
| PavDDL_‘Lapins’ |  | GT~A~CCTAAGTCTCCAA~GCATGCAAGGTCCCCTTCTCCTCCTGCTCGCTCTCCTTCTCCTCGGACAAAACGGTTAAGAAGAGCTCAAGCTGGCAGAGA |  |
| PavDDL_‘Garnet’ |  | GT~~CCCTAAGTCTCCAA~GCATGCAAGGTCCCCTTCTCCTCCTGCTCGCTCTCCTTCTCCTCGGACAAAACGGTTAAGAAGAGCTCAAGCTGGCAGAGA |  |
|  |  |  |  |
|  |  | ....|....|....|....|....|....|....|....|....|....|....|....|....|....|....|....|....|....|....|....| |  |
| ppa005178m |  | GGCTGTTAAAGAACCTGAAAGAAGTAACGGGAGGGGAACTGATAGGGGTTTACAGAAGGAAGGGGTTTCAGAGAGAGATGTCGGGAGTGATAGGAAAGAG |  |
| PRUAV004162 |  | ggctgtgaaagaacctgaaagaagtaatgggaggggaactgataggggtttacagaatgaaggggtttcagagagagatgtcgggagtgataggaaagag |  |
| PavDDL_‘Regina’ |  | GGCTGTGAAAGAAYCTGAAAGAAGTAATGGGAGGGGAACTGATAGGGGTTTACAGAATGAAGGGGTTTCAGAGAGAGATGTCGGGAGTGATAGGAAAGAG |  |
| PavDDL_‘Lapins’ |  | GGCTGTGAAAGAAYCTGAAAGAAGTAATGGGAGGGGAACTGATAGGGGTTTACAGAATGAAGGGGTTTCAGAGAGAGATGTCGGGAGTGATAGGAAAGAG |  |
| PavDDL_‘Garnet’ |  | GGCTGTGAAAGAAYCTGAAAGAAGTAATGGGAGGGGAACTGATAGGGGTTTACAGAATGAAGGGGTTTCAGAGAGAGATGTCGGGAGTGATAGGAAAGAG |  |
|  |  |  |  |
|  |  | ....|....|....|....|....|....|....|....|....|....|....|....|....|....|....|....|....|....|....|....| |  |
| ppa005178m |  | AAACGGTCGGGAAGAGACGATGTTGATGGTAAATCATCAAGACCAAGACATGGTACTTCTCCATCAGATCGACAGCGAAGGAGTAGGCATATATCTCCCT |  |
| PRUAV004162 |  | aaacggtcgggaagagacaatgttgacggtaaatcatcaagaccaagacatggtacttctccatcagatcgacagcgaaggagtaggcatatatctccct |  |
| PavDDL_‘Regina’ |  | AAACGGTCGGGAAGAGACAATGTTGACGGTAAATCATCAAGACCAAGACATGGTACTTCTCCATCAGATCGACAGCGAAGGAGTAGGCATATATCTCCCT |  |
| PavDDL_‘Lapins’ |  | AAACGGTCGGGAAGAGACAATGTTGACGGTAAATCATCAAGACCAAGACATGGTACTTCTCCATCAGATCGACAGCGAAGGAGTAGGCATATATCTCCCT |  |
| PavDDL_‘Garnet’ |  | AAACGGTCGGGAAGAGACAATGTTGACGGTAAATCATCAAGACCAAGACATGGTACTTCTCCATCAGATCGACAGCGAAGGAGTAGGCATATATCTCCCT |  |
|  |  |  |  |
|  |  | ....|....|....|....|....|....|....|....|....|....|....|....|....|....|....|....|....|....|....|....| |  |
| ppa005178m |  | CGCCTCAACCTGCTGGTGTTACCAGAGACGTGGAGAAAGTAATTGAGAATGACAGTGAAAGGAATCATGGCAGATGGAGTGATAGAAGAATGCAAAGGGA |  |
| PRUAV004162 |  | cacctcaacctgctggtgttaccagagacgtggagaaagtaattgagaatgacagtgaaaggaatcatggcagagggagtgatagaagaatgcaaaggga |  |
| PavDDL_‘Regina’ |  | CACCTCAACCTGCTGGTGTTACCAGAGACGTGGAGAAAGTAATTGAGAATGACAGTGAAAGGAATCATGGCAGAGGGAGTGATAGAAGAATGCAAAGGAA |  |
| PavDDL_‘Lapins’ |  | CACCTCAACCTGCTGGTGTTACCAGAGACGTGGAGAAAGTAATTGAGAATGACAGTGAAAGGAATCATGGCAGAGGGAGTGATAGAAGAATGCAAAGGAA |  |
| PavDDL_‘Garnet’ |  | CACCTCAACCTGCTGGTGTTACCAGAGACGTGGAGAAAGTAATTGAGAATGACAGTGAAAGGAATCATGGCAGAGGGAGTGATAGAAGAATGCAAAGGAA |  |
|  |  |  |  |
|  |  | ....|....|....|....|....|....|....|....|....|....|....|....|....|....|....|....|....|....|....|....| |  |
| ppa005178m |  | AAAGGGTTCAGATAGGGAAACCGATAGTGAAAGAGTGGAGAGAAGGTCAGGAAAAGACAGTACTGATCATAGGTCTTCAAGAACAAGACATGGGCGATCT |  |
| PRUAV004162 |  | aaagggttcagatagggaaactgatagtgaaagagtggagagaaggtcaggaaaagacagtactgatcataggtcttcaagaacaaaacatgggcaatct |  |
| PavDDL_‘Regina’ |  | AAAAGGGTTA |  |
| PavDDL_‘Lapins’ |  | AAAAGGGTAA |  |
| PavDDL_‘Garnet’ |  | AAAAAGGGTTA |  |

***ELF3***

|  |  | ....|....|....|....|....|....|....|....|....|....|....|....|....|....|....|....|....|....|....|....| |  |
| --- | --- | --- | --- |
| ppa002858m | 127 | ATCCCTTCTCAGAGGTTCAACCCTGTGGTGATGCCTCTTAATCCAAATAACACTAGCAGTGTGGTTCCCTCAGCTTTCTCGAGCCAGGTAAACTCATAAT |  |
| PRUAV005120 |  | ATCCCATCTCAGAGGTTCAACCCTGCGGTGATGCCTCTTAATCCAAATAGCACCAGCAGTGTGGTTCCCACAGCTTTCTCGAGCCAG~~~~~~~~~~~~~ |  |
| PavELF3_‘Regina’ |  | ~~~~~~~~~~~~~~~~~~~~~~~~~~~~~~~~~~~~~~~~~~~~~~~~~~~~~gtGATG~G~GGTT~CCACAGCTTTCTCGAGCCAGGTAAATTCATAAT |  |
| PavELF3_‘Lapins’ |  | ~~~~~~~~~~~~~~~~~~~~~~~~~~~~~~~~~~~~~~~~~~~~~~~~~~~~~gcgacgtgagtgcggcgatgcctcttatgagaacaggcccttgacat |  |
| PavELF3_‘Garnet’ |  | ~~~~~~~~~~~~~~~~~~~~~~~~~~~~~~~~~~~~~~~~~~~~~~~~~~aacaGGCGGTGTGGT~~~CACAGCTTTCTCGAGCCAGGTAAATTCATAAT |  |
|  |  |  |  |
|  |  | ....|....|....|....|....|....|....|....|....|....|....|....|....|....|....|....|....|....|....|....| |  |
| ppa002858m |  | TTGTGCCTAGCTTTCTCGT~TTAAGGTTTACTGAG~GATTAGTTTTGAAGTGTTATCCTTTTAAGCTTTAGTTAGGTTATGGATTTCACTAGGTTTGTTT |  |
| PRUAV005120 |  | ~~~~~~~~~~~~~~~~~~~~~~~~~~~~~~~~~~~~~~~~~~~~~~~~~~~~~~~~~~~~~~~~~~~~~~~~~~~~~~~~~~~~~~~~~~~~~~~~~~~~ |  |
| PavELF3_‘Regina’ |  | TTGTGCTTAACTTTCTCGTT~TAAGGTTTACTGAG~GATTAGTTTTGAAGTGTTATCCTTTTGAACTTTAGTTAGGTTATGGATTTCACTAGGTTTGTTT |  |
| PavELF3_‘Lapins’ |  | tggggcgaaactttctcGTTATAAAGTTTACTGAGAGATTAGTTTTGAAGTGTTATCCTTTTGAACTTTAGTTAGGTTATGGATTTCACTATGTTTGTTT |  |
| PavELF3_‘Garnet’ |  | TTGTGCTTAACTTTCTCGTT~TAAGGTTTACTGAG~GATTAGTTTTGAAGTGTTATCCTTTTGAACTTTAGTTAGGTTATGGATTTCACTAGGTTTGTTT |  |
|  |  |  |  |
|  |  | ....|....|....|....|....|....|....|....|....|....|....|....|....|....|....|....|....|....|....|....| |  |
| ppa002858m |  | TCTGTTTTTGTAGTAGCTAACTACAAATTCTGTTTTATGCTATATCCAGTAGTTTTGCATGCTACTATGTACATGTGTTGTGTTTTATTGCCAATATAGT |  |
| PRUAV005120 |  | ~~~~~~~~~~~~~~~~~~~~~~~~~~~~~~~~~~~~~~~~~~~~~~~~~~~~~~~~~~~~~~~~~~~~~~~~~~~~~~~~~~~~~~~~~~~~~~~~~~~~ |  |
| PavELF3_‘Regina’ |  | TCTATTTTTGTAGTAGCTAACTACAAATTTTGTTTTATGCTATATCCAGTAGTTTTGCATGCTACTATGTACATGTGTTGTGTTTT~TTGCCAATATAGT |  |
| PavELF3_‘Lapins’ |  | TCTATTTTTGTAGTAGCTAACTACAAATTTTGTTTTATGCTATATCCAGTAGTTTTGCATGCTACTATGTACATGTGTTGTGTTTT~TTGCCAATATAGT |  |
| PavELF3_‘Garnet’ |  | TCTATTTTTGTAGTAGCTAACTACAAATTTTGTTTTATGCTATATCCAGTAGTTTTGCATGCTACTATGTACATGTGTTGTGTTTT~TTGCCAATATAGT |  |
|  |  |  |  |
|  |  | ....|....|....|....|....|....|....|....|....|....|....|....|....|....|....|....|....|....|....|....| |  |
| ppa002858m |  | TCAGTCCAGAAAAGAAAATGCCAATATAACCAATATTGCTATATCTGCACTTTAAGAAGGGAAAATCAATAGTGTTTTCAGAAATAGTATTGGAAGCTTC |  |
| PRUAV005120 |  | ~~~~~~~~~~~~~~~~~~~~~~~~~~~~~~~~~~~~~~~~~~~~~~~~~~~~~~~~~~~~~~~~~~~~~~~~~~~~~~~~~~~~~~~~~~~~~~~~~~~~ |  |
| PavELF3_‘Regina’ |  | TCAGTCCAGAAAAGAAAATGCCAATATTACCAATATTGGCATATCTGCACTTTAAGAGGGGAAAATCAATAGTGTATTCAGAAATAGTATTGGAAGCTTC |  |
| PavELF3_‘Lapins’ |  | TCAGTCCAGAAAAGAAAATGCCAATATTACCAATATTGGCATATCTGCACTTTAAGAGGGGAAAATCAATAGTGTATTCAGAAATAGTATTGGAAGCTTC |  |
| PavELF3_‘Garnet’ |  | TCAGTCCAGAAAAGAAAATGCCAATATTACCAATATTGGCATATCTGCACTTTAAGAGGGGAAAATCAATAGTGTATTCAGAAATAGTATTGGAAGCTTC |  |
|  |  |  |  |
|  |  | ....|....|....|....|....|....|....|....|....|....|....|....|....|....|....|....|....|....|....|....| |  |
| ppa002858m |  | TATAGGGAAAATTGGTTATTTCATATCTGATTTTTCTGGAGCAATTTATGTTTTCAGGGAAGTCGTTCTGAAGGAAATTTGCCTGTTCCGCTTCATGTTC |  |
| PRUAV005120 |  | ~~~~~~~~~~~~~~~~~~~~~~~~~~~~~~~~~~~~~~~~~~~~~~~~~~~~~~~~GGGA~GTCGTTCTGAAGGAAATTTGCCTGTTCCGCTTCATGTTC |  |
| PavELF3_‘Regina’ |  | TATAGGGAAAATTGGTTATTGCATATCTGATTTTTCTGGAGCAATTTATGTTTTCAGGGGAGTCGTTCTGAAGGAAATTTGCCTGTTCCGCTTCATGTTC |  |
| PavELF3_‘Lapins’ |  | TATAGGGAAAATTGGTTATTGCATATCTGATTTTTCTGGAGCAATTTATGTTTTCAGGGGAGTCGTTCTGAAGGAAATTTGCCTGTTCCGCTTCATGTTC |  |
| PavELF3_‘Garnet’ |  | TATAGGGAAAATTGGTTATTGCATATCTGATTTTTCTGGAGCAATTTATGTTTTCAGGGGAGTCGTTCTGAAGGAAATTTGCCTGTTCCGCTTCATGTTC |  |
|  |  |  |  |
| 65 |  | ....|....|....|....|....|....|....|....|....|....|....|....|....|....|....|....|....|....|....|....| |  |
| ppa002858m |  | ATCCATCTACACCTAGTCGTCAGGCTGAGATGTTTCATGCTCGTCAATCTGATGGAGCAAATGAGAACACGCCCTTGACACAACCCGACCAAAGAAAGAA |  |
| PRUAV005120 |  | ATCCATCTACACCTACTCATCAGGCTGAGATGTTTCATGCTCGTCAATCTGATGGAGCAAATGAGAACACGCCCTTGACACAACCCGACCAAAGAAAGAA |  |
| PavELF3_‘Regina’ |  | ATCCATCTACACCTACTCATCAGGCTGAGATGTTTCATGCTCGTCAATCTGATGGAGCAAATGAGAACCCCCCTTGACAAT |  |
| PavELF3_‘Lapins’ |  | ATCCATCTACACCTACTCATCAGGCTGAGATGTTTCATGCTCGTCAATCTGATGGAGCAAATGAGAACACGCCCTTGAc |  |
| PavELF3_‘Garnet’ |  | ATCCATCTACACCTACTCATCAGGCTGAGATGTTTCATGCTCGTCAATCTGATGGAGCAAATGAGAACACCCCCTTGACA |  |

***ELF8***

|  |  | ....|....|....|....|....|....|....|....|....|....|....|....|....|....|....|....|....|....|....|....| |  |
| --- | --- | --- | --- |
| ppa015419m | 8201 | ATGTTCAAGATCCTCTTGCAGCAGCTGGGCTTGAAGATTCTGATGCCGAGGATGAGGTGGTAAATTTTATACATTCTCATGTCTGAGTTCTAACCTGCTG |  |
| PRUAV003418 |  | atgttcaagatcctcttgcagcagctgggcttgaagattctgatgccgaggatgagg~~~~~~~~~~~~~~~~~~~~~~~~~~~~~~~~~~~~~~~~~~~ |  |
| PavELF8_‘Regina’ |  | ~~~~~~~~~~~~~~~~~~~~~~~~~~~~~~~~~~~~~~~~~~~~~~~~~~~~~~~~~~~~~ggggatttttaatTCTCATGTCTGAGTTCTAACCTGCTG |  |
| PavELF8_‘Lapins’ |  | ~~~~~~~~~~~~~~~~~~~~~~~~~~~~~~~~~~~~~~~~~~~~~~~~~~~~~~~~~~~~~~~~~~TAATT~~~TCTCATGTCTGAGTTCTAACCTGCTG |  |
| PavELF8_‘Garnet’ |  | ~~~~~~~~~~~~~~~~~~~~~~~~~~~~~~~~~~~~~~~~~~~~~~~~~~~~~~~~~~~~~~~~~~~~~TTCG~TCTCATGTCTGAGTTCTAACCTGCTG |  |
|  |  |  |  |
|  |  | ....|....|....|....|....|....|....|....|....|....|....|....|....|....|....|....|....|....|....|....| |  |
| ppa015419m |  | AAAATTTCTTGCTGAAATGCATTGAGAGACTAAATGTTTCACTTCCTGCTGGCTGTAACATATTCATCTTATGTTCTAGGCTGCACCTTCAACAACCACA |  |
| PRUAV003418 |  | ~~~~~~~~~~~~~~~~~~~~~~~~~~~~~~~~~~~~~~~~~~~~~~~~~~~~~~~~~~~~~~~~~~~~~~~~~~~~~~~~ctgcaccttcaacaaccaca |  |
| PavELF8_‘Regina’ |  | AAAATTTCTTGCTGAAATGCATTGAGAGACTAAATGTTTCACTTCCTGCTGGCTGTAACATATTCATCTAATGTTCTAGGCTGCACCTTCAA |  |
| PavELF8_‘Lapins’ |  | aAAATTTCTTGCTGAAATGCATTGAGAGACTAMATGTTTCACTTCCTGCTGGCTGTAACATATTCATCTAATGTTCTAGGCTGCACCTTCAACAACCACA |  |
| PavELF8_‘Garnet’ |  | AAAATTTCTTGCTGAAATGCATTGAGAGACTACATGTTTCACTTCCTGCTGGCTGTAACATATTCATCTAATGTTCTAGGCTGCACCTTCAACAACCACA |  |
|  |  |  |  |
|  |  | ....|....|....|....|....|....|....|....|....|....|....|....|....|....|....|....|....|....|....|....| |  |
| ppa015419m |  | GTTCGACGAAGGCGGGCATGGTCAGAATCTGATGATGATGAGCAACAAGAGAGGCAGCCGGGGTCGAGTCCTGTAAGAGAAAATTCTGCAGAGTTACGGA |  |
| PRUAV003418 |  | gttcgacgaaggcgggcatggtcagaatctgatgatgatgagcaacaagagaggcagccgggatcgagtcctgtaagagaaaattctgcagagttacgga |  |
| PavELF8_‘Regina’ |  |  |  |
| PavELF8_‘Lapins’ |  | GTTCGACGAAGGCGGGCATGGTCAGAATCTGATGATGATGAGCAACAAGAGAGGCAGCCGGGATCGAGTCCTGTAAGAGAAAATTCTGCAGAGTTACGGA |  |
| PavELF8_‘Garnet’ |  | GTTCGACGAAGGCGGGCATGGTCAGAATCTGATGATGATGAGCAACAAGAGAGGCAGCCGGGATCGAGTCCTGTAAGAGAAAATTCTGCAGAGTTACAGA |  |
|  |  |  |  |
|  |  | ....|....|....|....|....|....|....|....|....|....|....|....|....|....|....|....|....|....|....|....| |  |
| ppa015419m |  | GTGATGGAGAAGGCAGAGAAGGCGGTGATAAAGTGAATGGAGAGGCCGCTCTTGATGATGATGATGATTAA |  |
| PRUAV003418 |  | gtgatggagaaggcagagaaggcggtgataaagggaatggagaggccgctcttgatgatgatga~~~ttaagatccatcatcttgagctctacggaatca |  |
| PavELF8_‘Regina’ |  |  |  |
| PavELF8_‘Lapins’ |  | GTGATGGAGAAGGCAGAGAAGGCGGTGATAAAGGGATGGGAGAGGCCC |  |
| PavELF8_‘Garnet’ |  | GTGATGGAGAAGGCAGAGAAGGCGGTGATAAAGGGAATGGAGAGGCCCA |  |

***EMF2***

|  |  | ....|....|....|....|....|....|....|....|....|....|....|....|....|....|....|....|....|....|....|....| |  |
| --- | --- | --- | --- |
| ppa002685m | 1901 | TTTTACTTGGCAGCAAATGCTGGAGGATATTGTCTGTGGAGCCAGATACCATTGGAATTACTGTATATCTCTTGGGAAAATTCTCCTAA~TTTTGTTTTG |  |
| PRUAV015728 |  | ~~~~~~~~~~~~~CAAATGCTGGAGGATATTGCCTGTGGAGCCAGATACCATTGGAATTACTGTATATCTCTTGGGAAA~TTCTCCTAANNTTTGGTCTG |  |
| PavEMF2_‘Regina’ |  | ~~~~~~~~~~~~~~~~~~~~~~~~~~~~~~~~~~~~~~~~~~~~~~~~~~~~~~~~~~~~~~NNNNNNNNNNNNNNNNNNNNNNNNNNN~~TANNNN~~G |  |
| PavEMF2_‘Lapins’ |  |  |  |
| PavEMF2_‘Garnet’ |  | ~~~~~~~~~~~~~~~~~~~~~~~~~~~~~~~~~~~~~~~~~~~~~~~~~~~~~~~~~~~~~~CCCTCCCATCATCGAACCTGCGCCTA~GTTATGGAATG |  |
|  |  |  |  |
|  |  | ....|....|....|....|....|....|....|....|....|....|....|....|....|....|....|....|....|....|....|....| |  |
| ppa002685m |  | GGACAGAGAGCTGAGATGATCTCAACTATTGACATGCACTCTTGCTTCTTGAAGGTATGGCGTCATCAGATGACTCTTGAATAACTATATGTCTTCTTCC |  |
| PRUAV015728 |  | GGACAGAGAGCTGAGATGATCTCCACTATTGACATGCACTCTTGCTTCTTGAAG~~~~~~~~~~~~~~~~~~~~~~~~~~~~~~~~~~~~~~~~~~~~~~ |  |
| PavEMF2_‘Regina’ |  | GGACAGAGAGCTGAGATGATCTCCACTATTGACATGCACTCTTGCTTCTTGAAGGTATGGCATCATCAGATGACTCTTGATTAACTATATGTCTTCTTTC |  |
| PavEMF2_‘Lapins’ |  |  |  |
| PavEMF2_‘Garnet’ |  | GGACAGAGAGCTGAGATGATCTCCACTATTGACATGCACTCTTGCTTCTTGAAGGTATGGCATCATCAGATGACTCTTGATTAACTATATGTCTTCTTTC |  |
|  |  |  |  |
|  |  | ....|....|....|....|....|....|....|....|....|....|....|....|....|....|....|....|....|....|....|....| |  |
| ppa002685m |  | TTGACAAAGAGGAACTGGTTAACAGTGCAGCTAAACCGGACTTGTAGTTGTCAAAAAATAAAAATAAAAATCATTGTTCTCTATTTGAATCTCTTATTCA |  |
| PRUAV015728 |  | ~~~~~~~~~~~~~~~~~~~~~~~~~~~~~~~~~~~~~~~~~~~~~~~~~~~~~~~~~~~~~~~~~~~~~~~~~~~~~~~~~~~~~~~~~~~~~~~~~~~~ |  |
| PavEMF2_‘Regina’ |  | TTGACAAAGAGGAACTGGTTAACAGTGCAGCTAAACCGGACTTGTAGTTGTAAAAAAA~~~~~~~~~AAATCATTGTTCTCTATTTGAATCTCGTATTCA |  |
| PavEMF2_‘Lapins’ |  |  |  |
| PavEMF2_‘Garnet’ |  | TTGACAAAGAGGAACTGGTTAACAGTGCAGCTAAACCGGACTTGTAGTTGTAAAAAAA~~~~~~~~~AAATCATTGTTCTCTATTTGAATCTCGTATTCA |  |
|  |  |  |  |
|  |  | ....|....|....|....|....|....|....|....|....|....|....|....|....|....|....|....|....|....|....|....| |  |
| ppa002685m |  | TGATTGGACCAAAATAATAGACCTTCTTTCTTCCACTCTCAGTTTGATTTACATGTTCCAATTTCCCTCTTGCAGTTGAGCTGTTTAAATGAGGATAAGT |  |
| PRUAV015728 |  | ~~~~~~~~~~~~~~~~~~~~~~~~~~~~~~~~~~~~~~~~~~~~~~~~~~~~~~~~~~~~~~~~~~~~~~~~~~~TTGAGCTGTTTAAATGAGGACAAGT |  |
| PavEMF2_‘Regina’ |  | TGATTGGACCAAAATAATAGACCTTCTGTCTTCCACTCTCAGTTTGATTTACATGTTCCAATTTCCCTCTTGCAGTTGAGCTGTTTAAATGAGGACAAGT |  |
| PavEMF2_‘Lapins’ |  |  |  |
| PavEMF2_‘Garnet’ |  | TGATTGGACCAAAATAATAGACCTTCTGTCTTCCACTCTCAGTTTGATTTACATGTTCCAATTTCCCTCTTGCAGTTGAGCTGTTTAAATGAGGACAAGT |  |
|  |  |  |  |
|  |  | ....|....|....|....|....|....|....|....|....|....|....|....|....|....|....|....|....|....|....|....| |  |
| ppa002685m |  | GCCTTATGATTGCAACTCCCTATAATCCAGAAACTGTTGTATGTTATCTTCTCAGTGTTTTCTCCTTTAATTCAATAATGTTGATCTCTCTCTCTCTATA |  |
| PRUAV015728 |  | GCCTTATGATTGCAACTCCCTATAATCCAGAAACTGTT~~~~~~~~~~~~~~~~~~~~~~~~~~~~~~~~~~~~~~~~~~~~~~~~~~~~~~~~~~~~~~ |  |
| PavEMF2_‘Regina’ |  | GCCTTATGATTGCAACTCCCTATAATCCAGAAACTGTTGTATGTTATCTTCTCAGTGTTTTCTCCTTTAATTCAATAATTCTGATCKCTCTCTCTCTCTA |  |
| PavEMF2_‘Lapins’ |  |  |  |
| PavEMF2_‘Garnet’ |  | GCCTTATGATTGCAACTCCCTATAATCCAGAAACTGTTGTATGTTATCTTCTCAGTGTTTTCTCCTTTAATTCAATAATTCTGATCTCTCTCTCTCTCTA |  |
|  |  |  |  |
|  |  | ....|....|....|....|....|....|....|....|....|....|....|....|....|....|....|....|....|....|....|....| |  |
| ppa002685m |  | TACACACGCGCGCGCGCGCACACACACACACACACACTATCCAACATTTCTCTTCTAGGCAACTGTTGCTGATGAATATCTGTTACTTTTGGTTGTTTGG |  |
| PRUAV015728 |  | ~~~~~~~~~~~~~~~~~~~~~~~~~~~~~~~~~~~~~~~~~~~~~~~~~~~~~~~~~~~~~~~~~~~~~~~~~~~~~~~~~~~~~~~~~~~~~~~~~~~~ |  |
| PavEMF2_‘Regina’ |  | ~~~~~~~~~~~~~~~~~~~~~~~~CACACACACACTCTATCCAACATTTCTCTTCTAGGCAACTGTTGCTGATGAATATCTGTTACTTTTGGTTTTTTGG |  |
| PavEMF2_‘Lapins’ |  |  |  |
| PavEMF2_‘Garnet’ |  | ~~~~~~~~~~~~~~~~~~~~~~~~CACACACACACTCTATCCAACATTTCTCTTCTAGGCAACTGTTGCTGATGAATATCTGTTACTTTTGGTTGTTTGG |  |
|  |  |  |  |
|  |  | ....|....|....|....|....|....|....|....|....|....|....|....|....|....|....|....|....|....|....|....| |  |
| ppa002685m |  | GCTTTCAAAGATCCTTTCCCAGCAACTCCAGGTCACCATTTCTGCAGAGGAGTTTGGTGCTAGGGAAAAATCTCTTTATAATACATACACACGTAGTGAC |  |
| PRUAV015728 |  | ~~~~~~~~~~ATCCTTTCCCAGCAACTACAGGTCACCATTTCTGCAGAGGAGTTTGGTGCTAGGGAAAAATCTCTTTATAATACATACATATGTAGTGAC |  |
| PavEMF2_‘Regina’ |  | GCTTTCAAAGATCCTTTCCCAGCAACTACAGGTCACCATTTCTGCANAGGAGTTTGGNGCTAGGGAAAAATCTCTTTATAATACATACATATGTAGTGAC |  |
| PavEMF2_‘Lapins’ |  |  |  |
| PavEMF2_‘Garnet’ |  | GCTTTCAAAGATCCTTTCCCAGCAACTACAGGTCACCATTTCTGCAGAGGAGTTTGGTGCTAGGGAAAAATCTCTTTATAATACATACATATGTAGTGAC |  |
|  |  |  |  |
|  |  | ....|....|....|....|....|....|....|....|....|....|....|....|....|....|....|....|....|....|....|....| |  |
| ppa002685m |  | ATTCCTTCTACATTGTTATCCCATATCATTCGGTAATCATCATCTCTCATAAAGTAATTATATCTGTTCACACCATTCTTTTTGTTGATTCTCATTCTCT |  |
| PRUAV015728 |  | ATTCCTTCTACATCGTTACCTCATATCATTCGGT~~~~~~~~~~~~~~~~~~~~~~~~~~~~~~~~~~~~~~~~~~~~~~~~~~~~~~~~~~~~~~~~~~ |  |
| PavEMF2_‘Regina’ |  | ATTCCTTCTACATCGTTACCTCATATCATTCGGTAATCATCATCTCTCATAAAGTAATTATGTCTGTTCGCACCATTCTGTTTATTGATTCTCATTCTCT |  |
| PavEMF2_‘Lapins’ |  |  |  |
| PavEMF2_‘Garnet’ |  | ATTCCTTCTACATCGTTACCTCATATCATTCGGTAATCATCATCTCTCATAAAGTAATTATGTCTGTTCGCACCATTCTGTTTATTGATTCTCATTCTCT |  |
|  |  |  |  |
|  |  | ....|....|....|....|....|....|....|....|....|....|....|....|....|....|....|....|....|....|....|....| |  |
| ppa002685m |  | GCTTCCCTTTAATTATCGTT~GTTAGATATTTGTTTGATAATTAACTAAATGCTGTTTCAGCATGGTGGCTCTACGCATTGAGTCTTTTGTAGCATAACT |  |
| PRUAV015728 |  | ~~~~~~~~~~~~~~~~~~~~~~~~~~~~~~~~~~~~~~~~~~~~~~~~~~~~~~~~~~~~~~~~~~~~~~~~~~~~~~~~~~~~~~~~~~~~~~~~~~~~ |  |
| PavEMF2_‘Regina’ |  | GCTTCCCTTTAATTATTGNNNGT~ANATATTTGTTTGATCATTAACTAAATGCTGTTTCAGCATGGTGGCTCTACGCATT~~~~~~~~~~~~~~~~~~~~ |  |
| PavEMF2_‘Lapins’ |  |  |  |
| PavEMF2_‘Garnet’ |  | GCTTCCCTTTAATTATTGTTTGTTAGATATTTGTTTGATCATTAACTAAATGCTGTTTCAACATGGTGGCTCTACGCATT~~~~~~~~~~~~~~~~~~~~ |  |
|  |  |  |  |
|  |  | ....|....|....|....|....|....|....|....|....|....|....|....|....|....|....|....|....|....|....|....| |  |
| ppa002685m |  | ATTTGATTTCTTTGCATCACTCAGAAAGTCAAAAGCATTAATATTCAAGGAAACATGCCAATTATGAGGCCAAATTTTGTGTCCTGTTTTTTTTCAATGT |  |
| PRUAV015728 |  | ~~~~~~~~~~~~~~~~~~~~~~~~~~~~~~~~~~~~~~~~~~~~~~~~~~~~~~~~~~~~~~~~~~~~~~~~~~~~~~~~~~~~~~~~~~~~~~~~~~~~ |  |
| PavEMF2_‘Regina’ |  | ~~~~~~~~~~~~~~~~~~~~~~~~~~~~~~~~~~~~~~~~~~~~~~~~~~~~~~~~~~~~~~TATGAGGCCAAATTTTGTGTACTGTTTTTTC~CNNGN~ |  |
| PavEMF2_‘Lapins’ |  |  |  |
| PavEMF2_‘Garnet’ |  | ~~~~~~~~~~~~~~~~~~~~~~~~~~~~~~~~~~~~~~~~~~~~~~~~~~~~~~~~~~~~~~TATGAGGCCAAATTTTGTGTACTGTTTTTTT~CATGGT |  |
|  |  |  |  |
|  |  | ....|....|....|....|....|....|....|....|....|....|....|....|....|....|....|....|....|....|....|....| |  |
| ppa002685m |  | TTATTCCACATTGGTTTCTTCCAGATAGTACCCTCACAGAACAACATATTT~GTTT~GACTAATCTATATGTATT~GCCTTTTCAAATATGTAACTTATA |  |
| PRUAV015728 |  | ~~~~~~~~~~~~~~~~~~~~~~~~~~~~~~~~~~~~~~~~~~~~~~~~~~~~~~~~~~~~~~~~~~~~~~~~~~~~~~~~~~~~~~~~~~~~~~~~~~~~ |  |
| PavEMF2_‘Regina’ |  | TTATTCCACANTAGTTTCTTCCAGATAGTACCCTCACAGTACA~CATATTTTGNTTTGACTAATCTAN~TGNATTTCCCTTTTNNAATATGNAGCTTANN |  |
| PavEMF2_‘Lapins’ |  |  |  |
| PavEMF2_‘Garnet’ |  | TTATTCCACATTAGTTTCTTCCAGATAGTACCCTCACAGTACAACATATT~TGTTT~GACTAATCTAAATGTATTTTCCTTTTCAAATATGGAGCTTATA |  |
|  |  |  |  |
|  |  | ....|....|....|....|....|....|....|....|....|....|....|....|....|....|....|....|....|....|....|....| |  |
| ppa002685m |  | CTTAGTGTTTGCATCTCAAC~TCGCATTTT~ATCAGTTTCCGG~TTCAGTTTAAATTG~AAGTGGTTAATCAATGG~TCTCTTGGTA~TTTGTCCATAA~ |  |
| PRUAV015728 |  | ~~~~~~~~~~~~~~~~~~~~~~~~~~~~~~~~~~~~~~~~~~~~~~~~~~~~~~~~~~~~~~~~~~~~~~~~~~~~~~~~~~~~~~~~~~~~~~~~~~~~ |  |
| PavEMF2_‘Regina’ |  | CTTANNGTT~GNNNN~CA~~CTNNCATTTTTANCNNTTTCCGG~TNCA~NTNAAATNN~A~G~GGNTNANNNANGGNNNNNTNGNTN~TTNGTCCANAA~ |  |
| PavEMF2_‘Lapins’ |  |  |  |
| PavEMF2_‘Garnet’ |  | CTTAGTGTTTGCATCTCAACCTCGCATTTT~ATCAGTTTCCGGGTTCA~TTTAAATTGGAAGTGGTTAATCAATGGGTCTCTTG~TAATTTGTCCATAAA |  |
|  |  |  |  |
|  |  | ....|....|....|....|....|....|....|....|....|....|....|....|....|....|....|....|....|....|....|....| |  |
| ppa002685m |  | GAATAGATTCCATGAACATAA~TTAGGGTATACTCCCAAATAACTT~C~TTATAATTCATTTT~CTTATATTCAT~GGAGAGGACAAAAT~CTGTGAGAT |  |
| PRUAV015728 |  | ~~~~~~~~~~~~~~~~~~~~~~~~~~~~~~~~~~~~~~~~~~~~~~~~~~~~~~~~~~~~~~~~~~~~~~~~~~~~~~~~~~~~~~~~~~~~~~~~~~~~ |  |
| PavEMF2_‘Regina’ |  | NAANNNNNTNCNNNNANNTAN~NNNGGGNNN~CTCCNNNN~~ACT~~CNTNNAAATNNNTTNN~~TTAN~~TCNN~GGA~NGNACAAA~T~CNN~GNNN~ |  |
| PavEMF2_‘Lapins’ |  |  |  |
| PavEMF2_‘Garnet’ |  | GAATAAAACTCAAGAACATAAATTAGGGTATACTCCCAAATAACTTTCCTTATAATTCATTTTTCCTAA~~TCATTG~AGAGGACAAAATTCTGTGAGAT |  |
|  |  |  |  |
|  |  | ....|....|....|....|....|....|....|....|....|....|....|....|....|....|....|....|....|....|....|....| |  |
| ppa002685m |  | C~TTAAGCTGATT~GTTTATGA~~TGCTTTGGCTTTT~AAGTCAGGATGTAGATTTATTTTGTGTTTCAGCTCTTGA~CCGTATGCT~TAAAAACTGCTT |  |
| PRUAV015728 |  | ~~~~~~~~~~~~~~~~~~~~~~~~~~~~~~~~~~~~~~~~~~~~~~~~~~~~~~~~~~~~~~~~~~~~~~~~~~~~~~~~~~~~~~~~~~~~~~~~~~~~ |  |
| PavEMF2_‘Regina’ |  | G~~~AANNNNNNNNNATNGNNNNN |  |
| PavEMF2_‘Lapins’ |  |  |  |
| PavEMF2_‘Garnet’ |  | CCTTAAGCTGATTTGTTTTAGAATTGCTT~GGCTTTTTTAGTCAGATTGTAAATTTATTTTGGTGTTCAGCCCTTGAACCATA~GCTCTAAAACCTGCTT |  |
|  |  |  |  |
|  |  | ....|....|....|....|....|....|....|....|....|....|....|....|....|....|....|....|....|....|....|....| |  |
| ppa002685m |  | GTTACTTGTCTTGTCCAGGTTAAGGGCAGGAAATGTGATTTTTAATTATAGATACTACAACAATACATTGCAAAGGACAGAAGGTATTAATGATGTTTTA |  |
| PRUAV015728 |  | ~~~~~~~~~~~~~~~~~~~~TAAGGGCAGGAAATGTGATTTTTAATTATAGATACTACAGCAATACATTGCAAAGGACAGAAG~~~~~~~~~~~~~~~~~ |  |
| PavEMF2_‘Regina’ |  |  |  |
| PavEMF2_‘Lapins’ |  |  |  |
| PavEMF2_‘Garnet’ |  | GT~ACT~GCTATG~~CAGGTTAAT~GCAAGAAAAAATGGTATCTTTAT |  |

***ESD4***

|  |  | ....|....|....|....|....|....|....|....|....|....|....|....|....|....|....|....|....|....|....|....| |  |
| --- | --- | --- | --- |
| ppa017866m | 1151 | CATGAGAACTCAAACATAGAGATCACAGGTGAAATGCTGCAGTGCCTTAGACCACGTGCATGGTTGAATGACGAGGTATTTTCTAGGTTACATTTGTACT |  |
| PRUAV016402 |  | CATGAGAACTCAAACATAGAGATCACAGGTGAAATGCTGCAGTGCCTTAGACCACGTGCATGGTTGAATGACGAG~~~~~~~~~~~~~~~~~~~~~~~~~ |  |
| PavESD4_‘Regina’ |  | ~~~~~~~~~~~~~~~~~~~~~~~~~~~~~~~~~~~~~~~~~~~~~~~~~~~~~~~~~~~~~GGTT~~~AGA~GAGGTATTTTCTAGGTTACATTTGTACT |  |
| PavESD4_‘Lapins’ |  | ~~~~~~~~~~~~~~~~~~~~~~~~~~~~~~~~~~~~~~~~~~~~~~~~~~~~~~~~~~~~~~acT~CCAGACGAGGTATTTTCTAGGTTACATTTGTACT |  |
| PavESD4_‘Garnet’ |  | ~~~~~~~~~~~~~~~~~~~~~~~~~~~~~~~~~~~~~~~~~~~~~~~~~~~~~~~~~~~~~~~~T~~~~GA~GAGGTATTTTCTAGGTTACATTTGTACT |  |
|  |  |  |  |
|  |  | ....|....|....|....|....|....|....|....|....|....|....|....|....|....|....|....|....|....|....|....| |  |
| ppa017866m |  | TTTTTGTTAAATTGCAATAACAAAAAAAACAATTCATTGCATTTTGTTTTGTATGGTTCATGTGGGGTTGTCCAGGCTTGCCTAGCTGGTAATGTGATTG |  |
| PRUAV016402 |  | ~~~~~~~~~~~~~~~~~~~~~~~~~~~~~~~~~~~~~~~~~~~~~~~~~~~~~~~~~~~~~~~~~~~~~~~~~~~~~~~~~~~~~~~~~~~~~~~~~~~~ |  |
| PavESD4_‘Regina’ |  | TTTTTGTTAAATTGCAATAACAAAAAAAAGTTTTCATTACATTTTGTTTTTTATGGTTCATGGGGGCTTTTCTGGGCTTGCCTAGTTGGTAATGTAATTG |  |
| PavESD4_‘Lapins’ |  | TTTTTGTTAAATTGCAATAACAAAAAAAAGTTTTCATTACATTTTGTTTTTTATGGTTCATGGGGGCTTTTCTGGGCTTGCCTAGTTGGTAATGTAATTG |  |
| PavESD4_‘Garnet’ |  | TTTTTGTTAAATTGCAATAACAAAAAAAAGTTTTCATTACATTTTGTTTTTTATGGTTCATGGGGGCTTTTCTGGGCTTGCCTAGTTGGTAATGTAATTG |  |
|  |  |  |  |
|  |  | ....|....|....|....|....|....|....|....|....|....|....|....|....|....|....|....|....|....|....|....| |  |
| ppa017866m |  | ATACATATGTATTCTTTTCCG~TCCAGGTCATAAATGTCTACTTTGAGTTGCTGAAAGAGAGGGAAAAGAGAGAGCCACAGAATTTCTTAAAATGTCATT |  |
| PRUAV016402 |  | ~~~~~~~~~~~~~~~~~~~~~~~~~~~GTCATAAATGTCTACTTTGAGTTGCTGAAAGAGAGGGAAAAGAGAGAGCCACAGAAGTTCTTAAAATGTCATT |  |
| PavESD4_‘Regina’ |  | ATACATTTGCACTCTTTTCCATTCCAGGTCATAAATGTCTACTTTGAGTTGCTGAAAGAGAGGGAAAAGAGAGAGCCACAGAAGTTCTTAAAATGTCATT |  |
| PavESD4_‘Lapins’ |  | ATACATTTGCACTCTTTTCCATTCCAGGTCATAAATGTCTACTTTGAGTTGCTGAAAGAGAGGGAAAAGAGAGAGCCACAGAAGTTCTTAAAATGTCATT |  |
| PavESD4_‘Garnet’ |  | ATACATTTGCACTCTTTTCCATTCCAGGTCATAAATGTCTACTTTGAGTTGCTGAAAGAGAGGGAAAAGAGAGAGCCACAGAAGTTCTTAAAATGTCATT |  |
|  |  |  |  |
|  |  | ....|....|....|....|....|....|....|....|....|....|....|....|....|....|....|....|....|....|....|....| |  |
| ppa017866m |  | TCTTCAACACATTTTTCTACAAAAAGGTTTGTAATTTGCTTTCTTTCTTGTTCATTCTTTTACACTTTGTTCCGGGCAGTGGTTGTATATGCATAATCTG |  |
| PRUAV016402 |  | TCTTCAACACATTTTTCTACAAAAAG~~~~~~~~~~~~~~~~~~~~~~~~~~~~~~~~~~~~~~~~~~~~~~~~~~~~~~~~~~~~~~~~~~~~~~~~~~ |  |
| PavESD4_‘Regina’ |  | TCTTCAACACATTTTTCTACAAAAAGGTTTGTAATTTGCTTTCTTTCTTGTTCTTTCTTTTACACTTTGTTCCTGGCGGTGGTTGTATATGCATAATCTC |  |
| PavESD4_‘Lapins’ |  | TCTTCAACACATTTTTCTACAAAAAGGTTTGTAATTTGCTTTCTTTCTTGTTCTTTCTTTTACACTTTGTTCCTGGCGGTGGTTGTATATGCATAATCTC |  |
| PavESD4_‘Garnet’ |  | TCTTCAACACATTTTTCTACAAAAAGGTTTGTAATTTGCTTTCTTTCTTGTTCTTTCTTTTACACTTTGTTCCTGGCGGTGGTTGTATATGCATAATCTC |  |
|  |  |  |  |
|  |  | ....|....|....|....|....|....|....|....|....|....|....|....|....|....|....|....|....|....|....|....| |  |
| ppa017866m |  | TTTAGTTTTCCTTTTTTTTTTTTTTACTGTCAGAATAATATTTATTGATTTCATTAATTTGTTGTCTAGATAAGTGCACTGGTATGAATACATGAGTTCT |  |
| PRUAV016402 |  | ~~~~~~~~~~~~~~~~~~~~~~~~~~~~~~~~~~~~~~~~~~~~~~~~~~~~~~~~~~~~~~~~~~~~~~~~~~~~~~~~~~~~~~~~~~~~~~~~~~~~ |  |
| PavESD4_‘Regina’ |  | TTCAGTTTTCCCTTTTTTTTTT~~~ACTGTCAAAAAAATTTTTATTGATTTCATTAATTTGTTGTCTAAATAAGT~~ACTGGTATGAATACATGAGTTCT |  |
| PavESD4_‘Lapins’ |  | TTCAGTTTTCCCTTTTTTTTTT~~~ACTGTCAAAAAAATTTTTATTGATTTCATTAATTTGTTGTCTAAATAAGT~~ACTGGTATGAATACATGAGTTCT |  |
| PavESD4_‘Garnet’ |  | TTCAGTTTTCCCTTTTTTTTTT~~~ACTGTCAAAAAAATTTTTATTGATTTCATTAATTTGTTGTCTAAATAAGT~~ACTGGTATGAATACATGAGTTCT |  |
|  |  |  |  |
|  |  | ....|....|....|....|....|....|....|....|....|....|....|....|....|....|....|....|....|....|....|....| |  |
| ppa017866m |  | TGTCACTGC~~~~~~~~~~~~~~~~~~~~~~~~~~~~~~~~~~~~~~~~~~~~~~~~~~~~~~~~~~AAAAACTATAAAGCTCTAATGCATGTTCATTGT |  |
| PRUAV016402 |  | ~~~~~~~~~~~~~~~~~~~~~~~~~~~~~~~~~~~~~~~~~~~~~~~~~~~~~~~~~~~~~~~~~~~~~~~~~~~~~~~~~~~~~~~~~~~~~~~~~~~~ |  |
| PavESD4_‘Regina’ |  | TGTCACTGCTTGATTTAGGGAACCACTTCTTAGGACTATTTCTGTTTTTATGAAGTAAAAAAAATGCAAAAATTATAAAGCTCTAATGCATGTTCATTTT |  |
| PavESD4_‘Lapins’ |  | TGTCACTGCTTGATTTAGGGAACCACTTCTTAGGACTATTTCTGTTTTTATGAAGTAAAAAAAATGCAAAAATTATAAAGCTCTAATGCATGTTCATTTT |  |
| PavESD4_‘Garnet’ |  | TGTCACTGCTTGATTTAGGGAACCACTTCTTAGGACTATTTCTGTTTTTATGAAGTAAAAAAAATGCAAAAATTATAAAGCTCTAATGCATGTTCATTTT |  |
|  |  |  |  |
|  |  | ....|....|....|....|....|....|....|....|....|....|....|....|....|....|....|....|....|....|....|....| |  |
| ppa017866m |  | CAAATAGTCTCATACTTCTATTTCATCATTTAATTATTAGGACTGTTTGTTGAGTTGGATTGGTCAAAAGCTTTGACCAATTTGGCCTGAATTTTT~ATT |  |
| PRUAV016402 |  | ~~~~~~~~~~~~~~~~~~~~~~~~~~~~~~~~~~~~~~~~~~~~~~~~~~~~~~~~~~~~~~~~~~~~~~~~~~~~~~~~~~~~~~~~~~~~~~~~~~~~ |  |
| PavESD4_‘Regina’ |  | CAAATAGTCTCATACTTCTATTTCATCATTTAATTATTAGGACTGTTTATTGAGTTGGATTGGTCAAAAGCTTTGATCAATTTGGCCTGAATTTTTTATT |  |
| PavESD4_‘Lapins’ |  | CAAATAGTCTCATACTTCTATTTCATCATTTAATTATTAGGACTGTTTATTGAGTTGGATTGGTCAAAAGCTTTGATCAATTTGGCCTGAATTTTTTATT |  |
| PavESD4_‘Garnet’ |  | CAAATAGTCTCATACTTCTATTTCATCATTTAATTATTAGGACTGTTTATTGAGTTGGATTGGTCAAAAGCTTTGATCAATTTGGCCTGAATTTTTTATT |  |
|  |  |  |  |
|  |  | ....|....|....|....|....|....|....|....|....|....|....|....|....|....|....|....|....|....|....|....| |  |
| ppa017866m |  | AGGATGAGTCTCATTTGAGTTTTGGCTGAGTTTTTAGACCTGATTTGAAGTTCAGGATGAATCAGCATGATATAACGCCAACCCAAATTGTACCCATTTG |  |
| PRUAV016402 |  | ~~~~~~~~~~~~~~~~~~~~~~~~~~~~~~~~~~~~~~~~~~~~~~~~~~~~~~~~~~~~~~~~~~~~~~~~~~~~~~~~~~~~~~~~~~~~~~~~~~~~ |  |
| PavESD4_‘Regina’ |  | AGGATGAGTCTCATTTGAGTTTTGGCTGAGTTTTTAGACCTGATTTGAAGTTCAGGATGAATCAGCATGATATAACGCCAACCCAAATTGTACCCATTTG |  |
| PavESD4_‘Lapins’ |  | AGGATGAGTCTCATTTGAGTTTTGGCTGAGTTTTTAAACCTGATTTGAAGTTCAGGATGAATCAGCATGATATAACGCCAACCCAAATTGTACCCATTTG |  |
| PavESD4_‘Garnet’ |  | AGGATGAGTCTCATTTGAGTTTTGGCTGAGTTTTTAGACCTGATTTGAAGTTCAGGATGAATCAGCATGATATAACGCCAACCCAAATTGTACCCATTTG |  |
|  |  |  |  |
|  |  | ....|....|....|....|....|....|....|....|....|....|....|....|....|....|....|....|....|....|....|....| |  |
| ppa017866m |  | TTTCTTTCTCATTTGTATACATATATTACATTTCATTAGGCCCGAAGATTGGGCTGGGCTGAGAGCTCAGCCATGTGCTTATTGGGCTAACCTCAGGCAA |  |
| PRUAV016402 |  | ~~~~~~~~~~~~~~~~~~~~~~~~~~~~~~~~~~~~~~~~~~~~~~~~~~~~~~~~~~~~~~~~~~~~~~~~~~~~~~~~~~~~~~~~~~~~~~~~~~~~ |  |
| PavESD4_‘Regina’ |  | TTTCTTTCTCATTTGTATACATATATTACATTTCATTAGGCCCGAAGATTGGGCTGGGCTGAGAGCTCAGCCATGTGCTTATTGGGCTAACCTCAGGCAA |  |
| PavESD4_‘Lapins’ |  | TTTCTTTCTCATTTGTATACATATATTACATTTCATTAGGCCCGAAGATTGGGCTGGGCTGAGAGCTCAGCCATGTGCTTATTGGGCTAACCTCAGGCAA |  |
| PavESD4_‘Garnet’ |  | TTTCTTTCTCATTTGTATACATATATTACATTTCATTAGGCCCGAAGATTGGGCTGGGCTGAGAGCTCAGCCATGTGCTTATTGGGCTAACCTCAGGCAA |  |
|  |  |  |  |
|  |  | ....|....|....|....|....|....|....|....|....|....|....|....|....|....|....|....|....|....|....|....| |  |
| ppa017866m |  | GGTCTGTCAGCTCAGCCCGTGCAATTCACACTCCTAGTAATTATTTGCCTAGATTGGGTAACCATCTTTGCTGTTGGATTTAGGATATTGATTTCTATGT |  |
| PRUAV016402 |  | ~~~~~~~~~~~~~~~~~~~~~~~~~~~~~~~~~~~~~~~~~~~~~~~~~~~~~~~~~~~~~~~~~~~~~~~~~~~~~~~~~~~~~~~~~~~~~~~~~~~~ |  |
| PavESD4_‘Regina’ |  | GGTCTGTCAGCTCAGCCCGTGCAATTCACACTCCTAGTAATTATTTGCCTAGATTGGGTAACCATCTTTGCTGTTGGATTTAGGATATTGATTTCTATAT |  |
| PavESD4_‘Lapins’ |  | GGTCTGTCAGCTCAGCCCGTGCAATTCACACTCCTAGTAATTATTTGCCTAGATTGGGTAACCATCTTTGCTGTTGGATTTAGGATATTGATTTCTATAT |  |
| PavESD4_‘Garnet’ |  | GGTCTGTCAGCTCAGCCCGTGCAATTCACACTCCTAGTAATTATTTGCCTAGATTGGGTAACCATCTTTGCTGTTGGATTTAGGATATTGATTTCTATAT |  |
|  |  |  |  |
|  |  | ....|....|....|....|....|....|....|....|....|....|....|....|....|....|....|....|....|....|....|....| |  |
| ppa017866m |  | TAGCTACTCTTATTTTACTGAAATTGTATGTGCAGTTGATAGGTGGGAAAAGTAACTATGATTATAAATCCGTCAGAAGGTGGACTACCCAAAAGAAGCT |  |
| PRUAV016402 |  | ~~~~~~~~~~~~~~~~~~~~~~~~~~~~~~~~~~~TTGATAGGTGGGAAAAGTAACTATGATTATAAATCCGTCAGAAGGTGGACTACCCAAAAGAAGCT |  |
| PavESD4_‘Regina’ |  | TAGCTACTCTTATTTTACTGAAATTGTATGTGCAGTTGATAGGTGGGAAAAGTAACTATGATTATAA~TCCGTCAgtgggagcacacacaccacaatc |  |
| PavESD4_‘Lapins’ |  | TAGCTACTCTTATTTTACTGAAATTGTATGTGCAGTTGATAGGTGGGAAAAGTAACTATGATTATAA~TCCGTCGAGGGGGaaaaacacacacatcagca |  |
| PavESD4_‘Garnet’ |  | TAGCTACTCTTATTTTACTGAAATTGTATGTGCAGTTGATAggtg~~aaaagta~ctatgat~ataa~tc~gtcaggggggagacacaccaaaattcggg |  |
|  |  |  |  |
|  |  | ....|....|....|....|....|....|....|....|....|....|....|....|....|....|....|....|....|....|....|....| |  |
| ppa017866m |  | GGGATACAGCCTCATCGATTGTGACAAAGTAACTTCATTCAAACCTTCTTCATTGCACATATACACAGTTTAAGTGAAATTACATTTATTTCATAAGTTG |  |
| PRUAV016402 |  | GGGATACAGCCTCATCGAATGTGACAAA~~~~~~~~~~~~~~~~~~~~~~~~~~~~~~~~~~~~~~~~~~~~~~~~~~~~~~~~~~~~~~~~~~~~~~~~ |  |
| PavESD4_‘Regina’ |  |  |  |
| PavESD4_‘Lapins’ |  | atatcctagcgtcat |  |
| PavESD4_‘Garnet’ |  | ccattttttcggggggggtgtat |  |

***FAR1***

|  |  | *....|....|....|....|....|....|....|....|....|....|....|....|....|....|....|....|....|....|....|....|* |
| --- | --- | --- |
| ppa001996m | 1901 | CAATACATGGTATTTGCGGTGTTGATGGAGAGAATCAATGCAGCACAGCATCTGGTGACAAGTTGTTTGGTCCCAAAGTGAGTAATGCCAACAAGACTCC |
| PRUAV021842 |  | ~~~~~~~~~~~~~~~~~~~~~~~~~~~~~~~~~~~~~~~~~~~~~~~~~~~~~~~~~~~~~~~~~~~~~~~~~~~~~~~~~~~AATGCCAACAAGACTCC |
| PavFAR1_‘Regina’ |  | ~~~~~~~~~~~~~~~~~~~~~~~~~~~~~~~~~~~~~~~~~~~~~~~~~~~~~~~~~~~~~~~~~~~~~~~~~~~~~~~~~~~~~~~~~~~~~~~~~~~~ |
| PavFAR1_‘Lapins’ |  | ~~~~~~~~~~~~~~~~~~~~~~~~~~~~~~~~~~~~~~~~~~~~~~~~~~~~~~~~~~~~~~~~~~~~~~~~~~~~~~~~~~~~~~~~~~~~~~~~~~~~ |
| PavFAR1_‘Garnet’ |  | ~~~~~~~~~~~~~~~~~~~~~~~~~~~~~~~~~~~~~~~~~~~~~~~~~~~~~~~~~~~~~~~~~~~~~~~~~~~~~~~~~~~~~~~~~~~~~~~~~~~~ |
|  |  |  |
|  |  | ....|....|....|....|....|....|....|....|....|....|....|....|....|....|....|....|....|....|....|....| |
| ppa001996m |  | TAGAAGAGCTGGGTCCGGGAAGGAGGTGGCTAGG~AATGAGAACACTGCAAGCAAGAAAGGAAAGGTACCTGATGGTGACGGTTTAAATATGGTCAATTT |
| PRUAV021842 |  | TAGAAGAGCTGGGTCCGGGAAGGAGGTGGCTAGGGAATGAGAACACTGCAAGCAAGAAAGGAAAGGTACCTCA~GCTGGAAGCTACGAGTGTTGGAACAC |
| PavFAR1_‘Regina’ |  | ~~~~~~~~~~~~~~ccggta~ggaagtgGCTAGG~~ATGAGAACACTGCAAGCAAGAAAGGAAAGGTACCTGATGGTGACAGTTTATATATGGTCAATTT |
| PavFAR1_‘Lapins’ |  | ~~~~~~~~~~~~~~tcggcagag~~GTGGCTAGG~~ATGAGAACACTGCAAGCAAGAAAGGAAAGGTACCTGATGGTGACAGTTTATATATGGTCAATTT |
| PavFAR1_‘Garnet’ |  | ~~~~~~~~~~~~~~agacggtagaagtggCTAGG~~ATGAGAACACTGCAAGCAAGAAAGGAAAGGTACCTGATGGTGACAGTTTATATATGGTCAATTT |
|  |  |  |
|  |  | ....|....|....|....|....|....|....|....|....|....|....|....|....|....|....|....|....|....|....|....| |
| ppa001996m |  | TTGTTTATT~ATTTTTTGGTTTTCATTTGAAATATAGTTTTGAATCTGATACGTGAATGAGTCATGTAATGTGAAAGAAAACTACTGATGGCTCAGGTTT |
| PRUAV021842 |  | AAGACGGCTTTCACCAAAT~~~~~~~~~~~~~~~~~~~~~~~~~~~~~~~~~~~~~~~~~~~~~~~~~~~~~~~~~~~~~~~~~~~~~~~~~~~~~~~~~ |
| PavFAR1_‘Regina’ |  | TTGTTTATTTTTATTTTTATTTTCATTTGAAATATAGTTTTGAATCTGATACATGAATGAGTCATGTAATTTGAAAGAAAACTACTGATGGTTTAGGTTT |
| PavFAR1_‘Lapins’ |  | TTGTTTATTTTTATTTTTATTTTCATTTGAAATATAGTTTTGAATCTGATACATGAATGAGTCATGTAATTTGAAAGAAAACTACTGATGGTTTAGGTTT |
| PavFAR1_‘Garnet’ |  | TTGTTTATTTTTATTTTTATTTTCATTTGAAATATAGTTTTGAATCTGATACATGAATGAGTCATGTAATTTGAAAGAAAACTACTGATGGTTTAGGTTT |
|  |  |  |
|  |  | ....|....|....|....|....|....|....|....|....|....|....|....|....|....|....|....|....|....|....|....| |
| ppa001996m |  | GCTTAATTGGCATAACAGGTACCTCACCTGGAAGCTACGAGTGTTGGAACACAAGATGGCTTTCACCAAATGGTATGTCATGCTTTCATCCTAATAGTAG |
| PRUAV021842 |  | ~~~~~~~~~~~~~~~~~~~~~~~~~~~~~~~~~~~~~~~~~~~~~~~~~~~~~~~~~~~~~~~~~~~~~~~~~~~~~~~~~~~~~~~~~~~~~~~~~~~~ |
| PavFAR1_‘Regina’ |  | GCTTAATTGGCATAACAGGTACCTCAGCTGGAAGCTACGAGTGTTGGAACACAAGACGGCTTTCACCAAATGGTATGTCATGCTTTCATCCTAATAGTAG |
| PavFAR1_‘Lapins’ |  | GCTTAATTGGCATAACAGGTACCTCAGCTGGAAGCTACGAGTGTTGGAACACAAGACGGCTTTCACCAAATGGTATGTCATGCTTTCATCCTAATAGTAG |
| PavFAR1_‘Garnet’ |  | GCTTAATTGGCATAACAGGTACCTCAGCTGGAAGCTACGAGTGTTGGAACACAAGACGGCTTTCACCAAATGGTATGTCATGCTTTCATCCTAATAGTAG |
|  |  |  |
|  |  | ....|....|....|....|....|....|....|....|....|....|....|....|....|....|....|....|....|....|....|....| |
| ppa001996m |  | TTTAGTTATGGGTGATTGCTTCCTTTTCTGATTTAATTTGGATGTAGTAAACTGTGGGTAATTTTAATAGAGGTATTCCTAAATATGGGTCCGCTCTGAA |
| PRUAV021842 |  | ~~~~~~~~~~~~~~~~~~~~~~~~~~~~~~~~~~~~~~~~~~~~~~~~~~~~~~~~~~~~~~~~~~~~~~~~~~~~~~~~~~~~~~~~~~~~~~~~~~~~ |
| PavFAR1_‘Regina’ |  | TTTAGTTATGGGTGATTGCTTCCTTTTCTGATTTAATTTGGATGTAGTAAATTGTGGGTAATTTTAATAGAGGTATTCCTAAATATGGGTCCGCTCTGAA |
| PavFAR1_‘Lapins’ |  | TTTAGTTATGGGTGATTGCTTCCTTTTCTGATTTAATTTGGATGTAGTAAATTGTGGGTAATTTTAATAGAGGTATTCCTAAATATGGGTCCGCTCTGAA |
| PavFAR1_‘Garnet’ |  | TTTAGTTATGGGTGATTGCTTCCTTTTCTGATTTAATTTGGATGTAGTAAATTGTGGGTAATTTTAATAGAGGTATTCCTAAATATGGGTCCGCTCTGAA |
|  |  |  |
|  |  | ....|....|....|....|....|....|....|....|....|....|....|....|....|....|....|....|....|....|....|....| |
| ppa001996m |  | AACTGATGATTTTTGAAGATTGTTGTCTTTCCAATCAAGAGACTTTGCTGTGCCAAAAACAAGATAACATTAATACTGGACATGGATTTGATAAGAGGGC |
| PRUAV021842 |  | ~~~~~~~~~~~~~~~~~~~~~~~~~~~~~~~~~~~~~~~~~~~~~~~~~~~~~~~~~~~~~~~~~~~~~~~~~~~~~~~~~~~~~~~~~~~~~~~~~~~~ |
| PavFAR1_‘Regina’ |  | AACTGATGATTTTTGAAGATYGTGGTCTTTCCAATCAAGAGATTTCACTGTGCCAAAAACAAGATAACATTAATACTGGACATGGATTTGATAAGGGGCC |
| PavFAR1_‘Lapins’ |  | AACTGATGATTTTTGAAGATTGTGGTCTTTCCAATCAAGAGATTTCACTGTGCCAAAAACAAGATAACATTAATACTGGACATGGATTTGATAAGGGGCC |
| PavFAR1_‘Garnet’ |  | AACTGATGATTTTTGAAGATYGTGGTCTTTCCAATCAAGAGATTTCACTGTGCCAAAAACAAGATAACATTAATACTGGACATGGATTTGATAAGGGGCC |
|  |  |  |
|  |  | ....|....|....|....|....|....|....|....|....|....|....|....|....|....|....|....|....|....|....|....| |
| ppa001996m |  | ATGGTCAAATTATGAGCATAATTTAAATCACGGATCTAATACTGCTAGCATAAGTTAGAAGTGAGGATGGCAGAATTTCACTGACCATCATGGATTCCTG |
| PRUAV021842 |  | ~~~~~~~~~~~~~~~~~~~~~~~~~~~~~~~~~~~~~~~~~~~~~~~~~~~~~~~~~~~~~~~~~~~~~~~~~~~~~~~~~~~~~~~~~~~~~~~~~~~~ |
| PavFAR1_‘Regina’ |  | ATGATCTAATTGTGAGCATAATTTAAATCACGGATCTAATACTGCTTGCATAAGTTAGAAGTGAGGATGGCAGAATTTCACTGACCATCATGGATTCCTG |
| PavFAR1_‘Lapins’ |  | ATGATCTAATTGTGAGCATAATTTAAATCACGGATCTAATACTGCTTGCATAAGTTAGAAGTGAGGATGGCAGAATTTCACTGACCATCATGGATTCCTG |
| PavFAR1_‘Garnet’ |  | ATGATCTAATTGTGAGCATAATTTAAATCACGGATCTAATACTGCTTGCATAAGTTAGAAGTGAGGATGGCAGAATTTCACTGACCATCATGGATTCCTG |
|  |  |  |
|  |  | ....|....|....|....|....|....|....|....|....|....|....|....|....|....|....|....|....|....|....|....| |
| ppa001996m |  | ACATGCTTTGATAAATTGACTTGAGAATACTGGATTCTGTAGCCTTTTGAAGATATACCAACAGGTTGAGGAAAACCTTTTGAACCCAATGGGTTATTAA |
| PRUAV021842 |  | ~~~~~~~~~~~~~~~~~~~~~~~~~~~~~~~~~~~~~~~~~~~~~~~~~~~~~~~~~~~~~~~~~~~~~~~~~~~~~~~~~~~~~~~~~~~~~~~~~~~~ |
| PavFAR1_‘Regina’ |  | ACATGCTTTGATAAATTGATTTGAGAATACTTGATTCTGTAGCCTTTTGTAGATATACCAACAGGTTGAGGAAAACCTTTTGAACCCAATGGGTTATTAA |
| PavFAR1_‘Lapins’ |  | ACATGCTTTGATAAATTGATTTGAGAATACTTGATTCTGTAGCCTTTTGTAGATATACCAACAGGTTGAGGAAAACCTTTTGAACCCAATGGGTTATTAA |
| PavFAR1_‘Garnet’ |  | ACATGCTTTGATAAATTGATTTGAGAATACTTGATTCTGTAGCCTTTTGTAGATATACCAACAGGTTGAGGAAAACCTTTTGAACCCAATGGGTTATTAA |
|  |  |  |
|  |  | ....|....|....|....|....|....|....|....|....|....|....|....|....|....|....|....|....|....|....|....| |
| ppa001996m |  | CTTATTGAACAGTGCTTATGTTTATGCTATGTGAA~TATATGTATCCTTTTCTTAGATGGTGGATGTTTGAGGTGAAGATTTTAAACTTGATGGGTTACT |
| PRUAV021842 |  | ~~~~~~~~~~~~~~~~~~~~~~~~~~~~~~~~~~~~~~~~~~~~~~~~~~~~~~~~~~~~~~~~~~~~~~~~~~~~~~~~~~~~~~~~~~~~~~~~~~~~ |
| PavFAR1_‘Regina’ |  | CTTATTGAACAGTGCTTATGTTTATGCTATGTGAAATATATGTATCCTTTTCTTAGATGGTGGATGTTTGAGGTGAAGATTTTGAACTTGATGGGTTACT |
| PavFAR1_‘Lapins’ |  | CTTATTGAACAGTGCTTATGTTTATGCTATGTGAAATATATGTATCCTTTTCTTAGATGGTGGATGTTTGAGGTGAAGATTTTGAACTTGATGGGTTACT |
| PavFAR1_‘Garnet’ |  | CTTATTGAACAGTGCTTATGTTTATGCTATGTGAAATATATGTATCCTTTTCTTAGATGGTGGATGTTTGAGGTGAAGATTTTGAACTTGATGGGTTACT |
|  |  |  |
|  |  | ....|....|....|....|....|....|....|....|....|....|....|....|....|....|....|....|....|....|....|....| |
| ppa001996m |  | GAACAATGCTTATATGTATGTAAAGTGTAACATTTTTTTTTTCATTTTATATCTAATAGATAGTTTAATCAAAATTGAACGATCATGGACACCGATAATT |
| PRUAV021842 |  | ~~~~~~~~~~~~~~~~~~~~~~~~~~~~~~~~~~~~~~~~~~~~~~~~~~~~~~~~~~~~~~~~~~~~~~~~~~~~~~~~~~~~~~~~~~~~~~~~~~~~ |
| PavFAR1_‘Regina’ |  | GAACAATGCTTATATGCATGTTAAGTGTAACATATTTTTTTTCATTTTATAACTAGTAGATAGTTGAATCAAAATTGAATGATCATGGACACCGATAATT |
| PavFAR1_‘Lapins’ |  | GAACAATGCTTATATGCATGTTAAGTGTAACATATTTTTTTTCATTTTATAACTAGTAGATAGTTGAATCAAAATTGAATGATCATGGACACCGATAATT |
| PavFAR1_‘Garnet’ |  | GAACAATGCTTATATGCATGTTAAGTGTAACATATTTTTTTTCATTTTATAACTAGTAGATAGTTGAATCAAAATTGAATGATCATGGACACCGATAATT |
|  |  |  |
|  |  | ....|....|....|....|....|....|....|....|....|....|....|....|....|....|....|....|....|....|....|....| |
| ppa001996m |  | TTGATTTGAAGATATCATTATACGTGTGTAGCATTTTTCTGTGAGGCAGTTTACTGCAAGTATGGTTTAGTGTGTGAGAGAATTCCCTACATGTGTAACA |
| PRUAV021842 |  | ~~~~~~~~~~~~~~~~~~~~~~~~~~~~~~~~~~~~~~~~~~~~~~~~~~~~~~~~~~~~~~~~~~~~~~~~~~~~~~~~~~~~~~~~~~~~~~~~~~~~ |
| PavFAR1_‘Regina’ |  | TTGATTTGAAGATATCATTATACGTGTGAAGCATTTTTCTGTGAGGCAGTTAACTGCAAGTATGGTTTAGTGT~~GAGAGAATTCCCTACGTGTGTAACA |
| PavFAR1_‘Lapins’ |  | TTGATTTGAAGATATCATTATACGTGTGAAGCATTTTTCTGTGAGGCAGTTAACTGCAAGTATGGTTTAGTGT~~GAGAGAATTCCCTACGTGTGTAACA |
| PavFAR1_‘Garnet’ |  | TTGATTTGAAGATATCATTATACGTGTGAAGCATTTTTCTGTGAGGCAGTTAACTGCAAGTATGGTTTAGTGT~~GAGAGAATTCCCTACGTGTGTAACA |
|  |  |  |
|  |  | ....|....|....|....|....|....|....|....|....|....|....|....|....|....|....|....|....|....|....|....| |
| ppa001996m |  | TTTGTAAGTTGAAAATATCGTATGCTGATCTCTAAGGTGAGATTTTGTATTGAACTTGTAATTTTGGGAAAAAGAAAATTCTGGTGGGCTTGAAGTTGAG |
| PRUAV021842 |  | ~~~~~~~~~~~~~~~~~~~~~~~~~~~~~~~~~~~~~~~~~~~~~~~~~~~~~~~~~~~~~~~~~~~~~~~~~~~~~~~~~~~~~~~~~~~~~~~~~~~~ |
| PavFAR1_‘Regina’ |  | TTTGTAAGTTGAAAATATCAAATGCTGATCGCTAAG~TGAGATTTTGTGTTGAACTTGTAATTTTGGGAAAA~GAAA~~~~~~~~~~~~~~~~~~TTGAG |
| PavFAR1_‘Lapins’ |  | TTTGTAAGTTGAAAATATCAAATGCTGATCGCTAAG~TGAGATTTTGTGTTGAACTTGTAATTTTGGGAAAA~GAAA~~~~~~~~~~~~~~~~~~TTGAG |
| PavFAR1_‘Garnet’ |  | TTTGTAAGTTGAAAATATCAAATGCTGATCGCTAAGGTGAGATTTTGTGTTgaacttgtatttttgg~aaaaagaaaa~~~~~~~~~~~~~~~~~ttgag |
|  |  |  |
|  |  | ....|....|....|....|....|....|....|....|....|....|....|....|....|....|....|....|....|....|....|....| |
| ppa001996m |  | CAAAATAGAGTAATAGATGC~TTGTTACATGTGGCAGCAACTCCCTGAC~ACAAGG~GCGATGCAGTTGCGGAACACGGTGCCATCGACAATGTTTCAAA |
| PRUAV021842 |  | ~~~~~~~~~~~~~~~~~~~~~~~~~~~~~~~~~~~~GCAACTCCCTGAC~ACAAGA~GCGATGCAGTTGCGGAACGCGGTGCCGTCGACAATGTTTCAAA |
| PavFAR1_‘Regina’ |  | CAAA~TAGAGTA~TAGatgcgt~gtacatgtgg~cagc~actccctgaa~acaaggagcgatgcagt~gcg~~acgcg~tgc~gtcgaca~tgtt~caaa |
| PavFAR1_‘Lapins’ |  | CAAA~TAGAGTAATAGATGCGT~GTtacatgtg~cagc~actccctgaccacaagaagcgatgcagt~gcgga~cgcg~tgc~gtcgaca~tgtt~caaa |
| PavFAR1_‘Garnet’ |  | caaaatagagtaatagatgcgttgttacatgtg~cagc~actccctgacc~caaga~gcgatgcagttgcggaacgcg~tgc~gtcgacaatgtttcaa~ |
|  |  |  |
|  |  | ....|....|....|....|....|....|....|....|....|....|....|....|....|....|....|....|....|....|....|....| |
| ppa001996m |  | ATGTTGCATCAGCAGCACAGTTCCATAATGTGGCATCAACACATGTGCATGAGAATCATCTCCCTCAATGA |
| PRUAV021842 |  | ATGTTGCATCAGCAGCACAGTTCCATAATGTGGTATCAACACATGTGCATGAGAATCATCTCCCTCAATGACTCAAACTTTACCTTGTCCGTTAAAAGAG |
| PavFAR1_‘Regina’ |  | ~tgt~gcatcgcccaacacactgtttctcttag |
| PavFAR1_‘Lapins’ |  | ~tgt~gtagtcagagctttattatatata |
| PavFAR1_‘Garnet’ |  | Ttgttgcatccagcagcacagatttcattaagctgt |

***FCA***

|  |  | ....|....|....|....|....|....|....|....|....|....|....|....|....|....|....|....|....|....|....|....| |  |
| --- | --- | --- | --- |
| ppa002369m | 6051 | CTAAATGTTTCTGGCGTCTTTCTTGTTCAGGGTTCCGGCATTCGGAGGTCCAGGTTTTGGTCCTCGATTTCAAACCCCTGGGGCGAGGTAATCCCCCACC |  |
| PRUAV005057 |  | ~~~~~~~~~~~~~~~~~~~~~~~~~~~~~~GGTTCCGGCATTCGGAGGTCCAGGTTTTGGTCCTCGATTTCAAGCCCCTGGGGCGAG~~~~~~~~~~~~~ |  |
| PavFCA_‘Regina’ |  | ~~~~~~~~~~~~~~~~~~~~~~~~~~~~~~~~~~~~~~~~~~~~~~~~~~~~~~~~~~~~~~~~~~~~~~~~~~~~~~cggggcGGGGTA~TCCCC~ACC |  |
| PavFCA_‘Lapins’ |  | ~~~~~~~~~~~~~~~~~~~~~~~~~~~~~~~~~~~~~~~~~~~~~~~~~~~~~~~~~~~~~~~~~~~~~~~~~~~~~~c~gag~GGAGTA~TCCCC~ACC |  |
| PavFCA_‘Garnet’ |  | ~~~~~~~~~~~~~~~~~~~~~~~~~~~~~~~~~~~~~~~~~~~~~~~~~~~~~~~~~~~~~~~~~~~~~~~~~~~~~cggggggGAAGTA~TCCCC~ACC |  |
|  |  |  |  |
|  |  | ....|....|....|....|....|....|....|....|....|....|....|....|....|....|....|....|....|....|....|....| |  |
| ppa002369m |  | ATTCTCCAAATATTTTGGTGAAGCTATTGCGTTTTGTCCTCATTTTATTCGCTGTAGTTATTTACATACTTTGTTGTTAACTTACTCCAAACAGACCAGT |  |
| PRUAV005057 |  | ~~~~~~~~~~~~~~~~~~~~~~~~~~~~~~~~~~~~~~~~~~~~~~~~~~~~~~~~~~~~~~~~~~~~~~~~~~~~~~~~~~~~~~~~~~~~~~ACCAGT |  |
| PavFCA_‘Regina’ |  | ATTCTC~TGATATTTTGGTGAAGCTATTGCGTTGTGTTCTCATTTTATTCGCTGTAGTTATTTACATACTTTGTTGTTAACTTACTCCAAACAGACCAGT |  |
| PavFCA_‘Lapins’ |  | ATTCTC~TGATATTTTGGTGAAGCTATTGCGTTGTGTTCTCATTTTATTCGCTGTAGTTATTTACATACTTTGTTGTTAACTTACTCCAAACAGACCAGT |  |
| PavFCA_‘Garnet’ |  | ATTCTC~TGATATTTTGGTGAAGCTATTGCGTTGTGTTCTCATTTTATTCGCTGTAGTTATTTACATACTTTGTTGTTAACTTACTCCAAACAGACCAGT |  |
|  |  |  |  |
|  |  | ....|....|....|....|....|....|....|....|....|....|....|....|....|....|....|....|....|....|....|....| |  |
| ppa002369m |  | CCCCAGCGTTGGTGATCCTATGAACGACCAAATTCCAACTCATGCTTGGCATCCAATGAGTCCACCAAACTTGGGACCATCCCCCAATGCTGGGATTCGT |  |
| PRUAV005057 |  | CCCTAGCTTTGGTGATCCTATGAACGACCAAATTCCAACTCATGCTTGGCATCCAATGAGTCCACCAAACTTGGGACCATCCCCCAATGCTGGGATTCGT |  |
| PavFCA_‘Regina’ |  | CCCTAGCTTTGGTGATCCTATGAACGACCAAATTCCAACTCATGCTTGGCATCCAATGAGTCCACCAAACTTGGGACCATCCCCCAATGCTGGGATTCGT |  |
| PavFCA_‘Lapins’ |  | CCCTAGCTTTGGTGATCCTATGAACGACCAAATTCCAACTCATGCTTGGCATCCAATGAGTCCACCAAACTTGGGACCATCCCCCAATGCTGGGATTCGT |  |
| PavFCA_‘Garnet’ |  | CCCTAGCTTTGGTGATCCTATGAACGACCAAATTCCAACTCATGCTTGGCATCCAATGAGTCCACCAAACTTGGGACCATCCCCCAATGCTGGGATTCGT |  |
|  |  |  |  |
|  |  | ....|....|....|....|....|....|....|....|....|....|....|....|....|....|....|....|....|....|....|....| |  |
| ppa002369m |  | GGCTTTGGGGGCCATTTTCTTCCTAGGGCCGGAAACATGGCGTTGCCCTTGAATTCGGTAAGAATAATTCAATGATTGTCCTGCAACAATTTGCCATGAG |  |
| PRUAV005057 |  | GGCTTTGGGGGCCATTTTCTTCCTAGGGCTG~~~ACATGGCTTTACCCTTGAATTCG~~~~~~~~~~~~~~~~~~~~~~~~~~~~~~~~~~~~~~~~~~~ |  |
| PavFCA_‘Regina’ |  | GGCTTTGGGGGCCATTTTCTTCCTAGGGCTG~~~ACATGGCTTTACCCTTGAATTCGGTAAGAATAATTCAATGATTGTCCTGCAACATTTTGCCATGAG |  |
| PavFCA_‘Lapins’ |  | GGCTTTGGGGGCCATTTTCTTCCTAGGGCTG~~~ACATGGCTTTACCCTTGAATTCGGTAAGAATAATTCAATGATTGTCCTGCAACATTTTGCCATGAG |  |
| PavFCA_‘Garnet’ |  | GGCTTTGGGGGCCATTTTCTTCCTAGGGCTG~~~ACATGGCTTTACCCTTGAATTCGGTAAGAATAATTCAATGATTGTCCTGCAACATTTTGCCATGAG |  |
|  |  |  |  |
|  |  | ....|....|....|....|....|....|....|....|....|....|....|....|....|....|....|....|....|....|....|....| |  |
| ppa002369m |  | AAACCATTATCTATTGTTACATGTTTCTTCTTCGTTCTCTTAAGACATCCATCAAAGGCGTATAGTATTTGACTTATCTGTAATGATTGGGCTGCAAATT |  |
| PRUAV005057 |  | ~~~~~~~~~~~~~~~~~~~~~~~~~~~~~~~~~~~~~~~~~~~~~~~~~~~~~~~~~~~~~~~~~~~~~~~~~~~~~~~~~~~~~~~~~~~~~~~~~~~~ |  |
| PavFCA_‘Regina’ |  | AAACCATTATCTATTGTTATCT~TTTG~~~~~CGTTCTCTTAAGACATCCATCAAAGGCATATAGCATTTGACTTATCTGTAATGATTGGGCTGCAAATT |  |
| PavFCA_‘Lapins’ |  | AAACCATTATCTATTGTTATCT~TTTG~~~~~CGTTCTCTTAAGACATCCATCAAAGGCATATAGCATTTGACTTATCTGTAATGATTGGGCTGCAAATT |  |
| PavFCA_‘Garnet’ |  | AAACCATTATCTATTGTTATCT~TTTG~~~~~CGTTCTCTTAAGACATCCATCAAAGGCATATAGCATTTGACTTATCTGTAATGATTGGGCTGCAAATT |  |
|  |  |  |  |
|  |  | ....|....|....|....|....|....|....|....|....|....|....|....|....|....|....|....|....|....|....|....| |  |
| ppa002369m |  | AAGTGATTTGTTGGCATTGAAATGGAGTGTTGATTGAATAGTATTACAATGACCTTGTCATGTCATCTTCAGCTGAAGCTATGAAAGGATGGCTGCTGTG |  |
| PRUAV005057 |  | ~~~~~~~~~~~~~~~~~~~~~~~~~~~~~~~~~~~~~~~~~~~~~~~~~~~~~~~~~~~~~~~~~~~~~~~~~~~~~~~~~~~~~~~~~~~~~~~~~~~~ |  |
| PavFCA_‘Regina’ |  | AAGTGATTTGTTGGCATTGAAATGGAGTCTTGATTGAATAGTATTACAATGACCTTGTCATGTCATCTTCAGCTGAAGCGATGAAAGGATGGCTGCTGCA |  |
| PavFCA_‘Lapins’ |  | AAGTGATTTGTTGGCATTGAAATGGAGTCTTGATTGAATAGTATTACAATGACCTTGTCATGTCATCTTCAGCTGAAGCGATGAAAGGATGGCTGCTGCA |  |
| PavFCA_‘Garnet’ |  | AAGTGATTTGTTGGCATTGAAATGGAGTCTTGATTGAATAGTATTACAATGACCTTGTCATGTCATCTTCAGCTGAAGCGATGAAAGGATGGCTGCTGCA |  |
|  |  |  |  |
|  |  | ....|....|....|....|....|....|....|....|....|....|....|....|....|....|....|....|....|....|....|....| |  |
| ppa002369m |  | GCATTGAATTTT~CTATTTTATTTAATTTATAATTTTCATGTTGTATATGATGCTAGTTTCTT~TTATGATTTTAGTTATAAATTTCTGGTTTTATGCTT |  |
| PRUAV005057 |  | ~~~~~~~~~~~~~~~~~~~~~~~~~~~~~~~~~~~~~~~~~~~~~~~~~~~~~~~~~~~~~~~~~~~~~~~~~~~~~~~~~~~~~~~~~~~~~~~~~~~~ |  |
| PavFCA_‘Regina’ |  | GCATTGAATTTTTCCTTTTTATTTAATTTATAATTTTCATGTTGTATATGATGCTAGTTTCTTGTTATGATTTTAGTTATAAATTTGTGGTTTTATGCTT |  |
| PavFCA_‘Lapins’ |  | GCATTGAATTTTTCCTTTTTATTTAATTTATAATTTTCATGTTGTATATGATGCTAGTTTCTTGTTATGATTTTAGTTATAAATTTGTGGTTTTATGCTT |  |
| PavFCA_‘Garnet’ |  | GCATTGAATTTTTCCTTTTTATTTAATTTATAATTTTCATGTTGTATATGATGCTAGTTTCTTGTTATGATTTTAGTTATAAATTTGTGGTTTTATGCTT |  |
|  |  |  |  |
|  |  | ....|....|....|....|....|....|....|....|....|....|....|....|....|....|....|....|....|....|....|....| |  |
| ppa002369m |  | TTAAAAGTCTTATTATTTGCTCCCTAAATATTTAGGGTGGCCATGGTGGCTCTGCAGAGGGTCCTCTTCCTGGGATGCCAGTTTCGTCTTCTTTAACATC |  |
| PRUAV005057 |  | ~~~~~~~~~~~~~~~~~~~~~~~~~~~~~~~~~~~GGTGGCCATGGTGGCTCTGCAGAGGGTCCTCTTCCTGGGATGCCAGTTTCATCTTCTTTAACATC |  |
| PavFCA_‘Regina’ |  | TTAAAAGTCTTATTATTTGCTCCCCAAATATTTAGGGTGGCCATGGTGGCTCTGCAGAGGGTCCTCTTCCTGGGATGCCAGTTTCATCTTCTTTAACATC |  |
| PavFCA_‘Lapins’ |  | TTAAAAGTCTTATTATTTGCTCCCCAAATATTTAGGGTGGCCATGGTGGCTCTGCAGAGGGTCCTCTTCCTGGGATGCCAGTTTCATCTTCTTTAACATC |  |
| PavFCA_‘Garnet’ |  | TTAAAAGTCTTATTATTTGCTCCCCAAATATTTAGGGTGGCCATGGTGGCTCTGCAGAGGGTCCTCTTCCTGGGATGCCAGTTTCATCTTCTTTAACATC |  |
|  |  |  |  |
|  |  | ....|....|....|....|....|....|....|....|....|....|....|....|....|....|....|....|....|....|....|....| |  |
| ppa002369m |  | ACAACAGGTATAAGGTGATCAAATTTTCTTTTAATTTACATCGGACTCAAAAATCAAAACAATGATCTTTAAGTTTTCGTGGTATTTCATAGTGAAAATG |  |
| PRUAV005057 |  | ACAACAG~~~~~~~~~~~~~~~~~~~~~~~~~~~~~~~~~~~~~~~~~~~~~~~~~~~~~~~~~~~~~~~~~~~~~~~~~~~~~~~~~~~~~~~~~~~~~ |  |
| PavFCA_‘Regina’ |  | ACAACAGGTATAAGGTGATCAAATTTTCTTTTAATTTACTTCGGACTCAAAAATCAAAACAATGATCTTTCAGTTTTCGTGGTATTTCATAGTGAAAATG |  |
| PavFCA_‘Lapins’ |  | ACAACAGGTATAAGGTGATCAAATTTTCTTTTAATTTACTTCGGACTCAAAAATCAAAACAATGATCTTTCAGTTTTCGTGGTATTTCATAGTGAAAATG |  |
| PavFCA_‘Garnet’ |  | ACAACAGGTATAAGGTGATCAAATTTTCTTTTAATTTACTTCGGACTCAAAAATCAAAACAATGATCTTTCAGTTTTCGTGGTATTTCATAGTGAAAATG |  |
|  |  |  |  |
|  |  | ....|....|....|....|....|....|....|....|....|....|....|....|....|....|....|....|....|....|....|....| |  |
| ppa002369m |  | ACATATTGAATCTTTTTCAGAGTTTTAACCAGTCTATGCCGCATGTCGGCCAGAAAATATCTCCAGTGCAGAAGCCTATTCAGTCACCTCAGCATTTGCC |  |
| PRUAV005057 |  | ~~~~~~~~~~~~~~~~~~~~AGTTGTAACCAGTCTATGCCACACGTCGGCCAGAAAATATCTCCAGTACAGAAGCCTATTCAGTCACCTCAGCATTTGCC |  |
| PavFCA_‘Regina’ |  | ACATATTGAATCTTTTTCAGAGTTGTAACCAG~CAGGCCCCCCccctaaaa |  |
| PavFCA_‘Lapins’ |  | ACATATTGAATCTTTTTCAGAGTTGTAACCAGTGACCCccccccccaaaaa |  |
| PavFCA_‘Garnet’ |  | ACATATTGAATCTTTTTCAGAGTTGTAACCAG~CAGGCCCCCcccccaaaa |  |

***FIE1***

|  |  | ....|....|....|....|....|....|....|....|....|....|....|....|....|....|....|....|....|....|....|....| |  |
| --- | --- | --- | --- |
| ppa007346m | 1 | AGGGGTTGGTCTGTTGCCATCCTGCGCTGTGGACATGGCGGCGAAGTTCGCTTTAGGGTCGGAGCCAG~TGGCGGGTTCACTCGCACCCTCAAAGAAGAG |  |
| PRUAV011214 |  | AGGAGTTGGTCTGTTGCCATCCTGCGCTGTGGAGATGGCGGCGAAGTTCGCTTTAGGGTCGGAGCCAG~TGGCGGGTTCACTCGCACCCTCAAAGAAGAG |  |
| PavFIE1_‘Regina’ |  | ~~~~~~~~~~~~~~~~~~~~~~~~~~~~~~~~GTCAATGAGTGGTGTT~G~TTT~GGGTGGGAG~CAGATGGCGGGTTCACTCGCACCCTCAAAGAAGAG |  |
| PavFIE1_‘Lapins’ |  | ~~~~~~~~~~~~~~~~~~~~AAGGCCGGAGTGTACACCATAAATCATT~G~~TT~~GATCGGAGCCAG~TGGCGGGTTCACTCGCACCCTCAAAGAAGAG |  |
| PavFIE1_‘Garnet’ |  |  |  |
|  |  |  |  |
|  |  | ....|....|....|....|....|....|....|....|....|....|....|....|....|....|....|....|....|....|....|....| |  |
| ppa007346m |  | AGAGTACAGAGTCACTAACAGGCTCCAAGAGGGCAAGAGGCCCTTATACGCCATCGTTTTCAACTTCATCGACTCTCGCTACTTCAACGTCTTCGCCACA |  |
| PRUAV011214 |  | AGAGTACAGAGTCACTAACAGGCTCCAAGAGGGCAAGAGGCCCTTATACGCCATCGTTTTCAACTTCATCGACTCTCGCTACTTCAACGTCTTCGCCACA |  |
| PavFIE1_‘Regina’ |  | AGAGTACAGAGTCACTAACAGGCTCCAAGAGGGCAAGAGGCCCTTATACGCCATCGTTTTCAACTTCATCGACTCTCGCTACTTCAACGTCTTCGCCACA |  |
| PavFIE1_‘Lapins’ |  | AGAGTACAGAGTCACTAACAGGCTCCAAGAGGGCAAGAGGCCCTTATACGCCATCGTTTTCAACTTCATCGACTCTCGCTACTTCAACGTCTTCGCCACA |  |
| PavFIE1_‘Garnet’ |  |  |  |
|  |  |  |  |
|  |  | ....|....|....|....|....|....|....|....|....|....|....|....|....|....|....|....|....|....|....|....| |  |
| ppa007346m |  | GTCGGTGGCAATCGGGTTTGTTGCTCTTGCCCTCTATAACTCATCCTACAAGGAACTAAAAGCAACAAAAAAGAACTCTTTTTTCGTAAAAGAAATTTAC |  |
| PRUAV011214 |  | GTCGGTGGCAATCGGGT~~~~~~~~~~~~~~~~~~~~~~~~~~~~~~~~~~~~~~~~~~~~~~~~~~~~~~~~~~~~~~~~~~~~~~~~~~~~~~~~~~~ |  |
| PavFIE1_‘Regina’ |  | GTCGGTGGCAATCGGGTTTGTTGCTCTTGCCCTCTATAACTCATCCTACAAGGAACTAAAAGCAACAAAAAAGAACTCTTTTTTTGTAAAAGAAATTTAC |  |
| PavFIE1_‘Lapins’ |  | GTCGGTGGCAATCGGGTTTGTTGCTCTTGCCCTCTATAACTCATCCTACAAGGAACTAAAAGCAACAAAAAAGAACTCTTTTTTTGTAAAAGAAATTTAC |  |
| PavFIE1_‘Garnet’ |  |  |  |
|  |  |  |  |
|  |  | ....|....|....|....|....|....|....|....|....|....|....|....|....|....|....|....|....|....|....|....| |  |
| ppa007346m |  | AGTGGAACTCCAATGCCGGGAT~GTAAGCTGGACTTTCCTTCTTTTTCTCGGGAACCAAACAGAGATTTTATGCTTTTTTGGCTGATTGTTATTATTTTT |  |
| PRUAV011214 |  | ~~~~~~~~~~~~~~~~~~~~~~~~~~~~~~~~~~~~~~~~~~~~~~~~~~~~~~~~~~~~~~~~~~~~~~~~~~~~~~~~~~~~~~~~~~~~~~~~~~~~ |  |
| PavFIE1_‘Regina’ |  | AATGGAACTCCAATGCCGGGATTGTAAGCTGGACTTTCCTTCTTTTTCTCGGGAACCAAACAGAGATTTTATGCTTTTTTGGCTGATTGTTATTATTTTT |  |
| PavFIE1_‘Lapins’ |  | AATGGAACTCCAATGCCGGGATTGTAAGCTGGACTTTCCTTCTTTTTCTCGGGAACCAAACAGAGATTTTATGCTTTTTTGGCTGATTGTTATTATTTTT |  |
| PavFIE1_‘Garnet’ |  |  |  |
|  |  |  |  |
|  |  | ....|....|....|....|....|....|....|....|....|....|....|....|....|....|....|....|....|....|....|....| |  |
| ppa007346m |  | TTA~~~~~~ATTATATTTTGCTTTAATGGGTTTTGTTAGATAGAGTGGATTACTAGAGATTTCGGCTCAATCTGGGTTTTTGGTCTCGGCTTTGCCTAAT |  |
| PRUAV011214 |  | ~~~~~~~~~~~~~~~~~~~~~~~~~~~~~~~~~~~~~~~~~~~~~~~~~~~~~~~~~~~~~~~~~~~~~~~~~~~~~~~~~~~~~~~~~~~~~~~~~~~~ |  |
| PavFIE1_‘Regina’ |  | TATTTATTTATTATATTTTGTTTTAATGGGTTTTGTTAGATAGAGTGGATTACTAGAGATTTCGGCTCTATCTGGGTTTTTGGTCTCGGCYTTGCCTAAT |  |
| PavFIE1_‘Lapins’ |  | TATTTATTTATTATATTTTGTTTTAATGGGTTTTGTTAGATAGAGTGGATTACTAGAGATTTCGGCTCTATCTGGGTTTTTGGTCTCGGCTTTGCCTAAT |  |
| PavFIE1_‘Garnet’ |  |  |  |
|  |  |  |  |
|  |  | ....|....|....|....|....|....|....|....|....|....|....|....|....|....|....|....|....|....|....|....| |  |
| ppa007346m |  | AATTTGTTTACATAGACGAAAGTTAGGGCCTTGGAATACAATGAAATGATTATTAAGGTTAGTGTTTGGGGAAAACAACGCAAAAGGCACCACATTTGAT |  |
| PRUAV011214 |  | ~~~~~~~~~~~~~~~~~~~~~~~~~~~~~~~~~~~~~~~~~~~~~~~~~~~~~~~~~~~~~~~~~~~~~~~~~~~~~~~~~~~~~~~~~~~~~~~~~~~~ |  |
| PavFIE1_‘Regina’ |  | AATTTGTTTACATAGACGAAAATTAGGGCCTTGGAATACAATGAAATGATTATTAAGGTTAGTGTTTGGGGGAAAAAACGCAAAAGGCACCACATTTGAT |  |
| PavFIE1_‘Lapins’ |  | AATTTGTTTACATAGACGAAAATTAGGGCCTTGGAATACAATGAAATGATTATTAAGGTTAGTGTTTGGGGGAAAAAACGCAAAAGGCACCACATTTGAT |  |
| PavFIE1_‘Garnet’ |  |  |  |
|  |  |  |  |
|  |  | ....|....|....|....|....|....|....|....|....|....|....|....|....|....|....|....|....|....|....|....| |  |
| ppa007346m |  | ATTTACAATATTGGATTGCAATGCACCTGTGGTAGGGGTGAATTATAGTTGTTCTGAGCTGGTAATGTTATAGTTCACTGCTTCATTTGCAAATAAACAA |  |
| PRUAV011214 |  | ~~~~~~~~~~~~~~~~~~~~~~~~~~~~~~~~~~~~~~~~~~~~~~~~~~~~~~~~~~~~~~~~~~~~~~~~~~~~~~~~~~~~~~~~~~~~~~~~~~~~ |  |
| PavFIE1_‘Regina’ |  | ATTTACAATATTGGATTGCAATGCAGCTGTGGTAGGGGTGAATTATAGTTGTTCTGAGCTGGTAATGTTATAGTTCACTGCTTCATTTGCAAATAAACAA |  |
| PavFIE1_‘Lapins’ |  | ATTTACAATATTGGATTGCAATGCAGCTGTGGTAGGGGTGAATTATAGTTGTTCTGAGCTGGTAATGTTATAGTTCACTGCTTCATTTGCAAATAAACAA |  |
| PavFIE1_‘Garnet’ |  |  |  |
|  |  |  |  |
|  |  | ....|....|....|....|....|....|....|....|....|....|....|....|....|....|....|....|....|....|....|....| |  |
| ppa007346m |  | CATGAATTAGTTCCCATAAGTGATTATTTGAGTAAAAATCTTATCTGAGAGAGACAAATT~ACAAGGTTGGTTCTCTTTCCTCTATTT~CTTGTACTTGT |  |
| PRUAV011214 |  | ~~~~~~~~~~~~~~~~~~~~~~~~~~~~~~~~~~~~~~~~~~~~~~~~~~~~~~~~~~~~~~~~~~~~~~~~~~~~~~~~~~~~~~~~~~~~~~~~~~~~ |  |
| PavFIE1_‘Regina’ |  | CATGAATTAGTTCCCATAAGTGATTATTTGCGTAAAAATCTTATCTGAGAGAGACAAATT~ACAAGGTTGGTTCTCTTTCCTCTATTT~CTTGTACTTGT |  |
| PavFIE1_‘Lapins’ |  | CATGAATTAGTTCCCATAAGTGATTATTTGCGTAAAAATCTTATCTGAGAGAGACAAATTTACAAGGTTGGTTCTCTTTCCTCTATTTTCTTGTACTTGT |  |
| PavFIE1_‘Garnet’ |  |  |  |
|  |  |  |  |
|  |  | ....|....|....|....|....|....|....|....|....|....|....|....|....|....|....|....|....|....|....|....| |  |
| ppa007346m |  | TGTAAAATGTTGCTTATTTTTATATTATTCTTTT~~GTCAGGTGACTGTATACCAATGTCTAGAAGGGGG~TGTGATCGCCG~TGTTGCAGTC~TTACAT |  |
| PRUAV011214 |  | ~~~~~~~~~~~~~~~~~~~~~~~~~~~~~~~~~~~~~~~~~~~GACTGTATACCAATGTCTAGAAGGGGG~CGTGATCGCTG~TGTTGCAGTC~TTACAT |  |
| PavFIE1_‘Regina’ |  | TGTAAATTGTTGCTTATTTTTATTTT~ATTCTTTT~GTCAGGTGACTGTATACCAATGTCTAGAAGGGGGGCGTGATCGCTGGGGTTGCAGTCCTTACAT |  |
| PavFIE1_‘Lapins’ |  | TGTAAATTGTTGCTTATTTTTTTTTTTATTCTTTTTGTCAGGTGACTGTATACCA~TGTCTA~AAGGGGGGCGTGATCGCTGGTGTTGCAGTC~TTAC~ |  |
| PavFIE1_‘Garnet’ |  |  |  |
|  |  |  |  |
|  |  | ....|....|....|....|....|....|....|....|....|....|....|....|....|....|....|....|....|....|....|....| |  |
| ppa007346m |  | T~GATGAA~GATGTAAGTAAA~~GACAGCC~ATCATTC~TG~CCATTGT~TCAAAT~~GGATTAGTGTTCTCTTGTCAGTCACTTATCATAC~~~~~~~~ |  |
| PRUAV011214 |  | T~GATGAA~GAT~~~~~~~~~~~~~~~~~~~~~~~~~~~~~~~~~~~~~~~~~~~~~~~~~~~~~~~~~~~~~~~~~~~~~~~~~~~~~~~~~~~~~~~~ |  |
| PavFIE1_‘Regina’ |  | TTGATGAAAGATGTAAGGTAAAATACAGCCCATCATTCCTGGCCATTTTGTCAAAAGGGGATTAATTTGTTCCACTTAGTCCGGTCCACTTATACAAAAT |  |
| PavFIE1_‘Lapins’ |  | TTTGATGAA~GAAGTAAG~TAAA~GACAGCC~A |  |
| PavFIE1_‘Garnet’ |  |  |  |
|  |  |  |  |
|  |  | ....|....|....|....|....|....|....|....|....|....|....|....|....|....|....|....|....|....|....|....| |  |
| ppa007346m |  | GGTTT~AGCATTCATTTGCGATTTTTAAAACTTTTCTTTGTCGATATGTGATTTACATGTGCTTTGTAGATCAGAAGGATGAGTCTTTTTACACTGCGAG |  |
| PRUAV011214 |  | ~~~~~~~~~~~~~~~~~~~~~~~~~~~~~~~~~~~~~~~~~~~~~~~~~~~~~~~~~~~~~~~~~~~~~~~~~~AAGGATGAGTCTTTTTACACTGCGAG |  |
| PavFIE1_‘Regina’ |  | GGTTTTAGCATTTCTTTTTGGTCTGAAATTCTTAAAAATTTTTTTTTTTTGCGAGATATGGT |  |
| PavFIE1_‘Lapins’ |  |  |  |
| PavFIE1_‘Garnet’ |  |  |  |
|  |  |  |  |
|  |  | ....|....|....|....|....|....|....|....|....|....|....|....|....|....|....|....|....|....|....|....| |  |
| ppa007346m |  | CTGGGCATGCAACATTGATGGAAACCCATTTCTTGTGGCTGGAGGAATTAATGGTATAATGCGGGTCATTGATTGTGGTAGTGAGAAGATAGACAAGGTA |  |
| PRUAV011214 |  | CTGGGCATGCAATGTTGATGGAAACCCATTTCTTGTGGCTGGAGGAATTAATGGTATAATGCGGGTCATTGATTGTGGTAGTGAGAAGATAGACAAG~~~ |  |
| PavFIE1_‘Regina’ |  |  |  |
| PavFIE1_‘Lapins’ |  |  |  |
| PavFIE1_‘Garnet’ |  |  |  |

***FIO1***

|  |  | *....|....|....|....|....|....|....|....|....|....|....|....|....|....|....|....|....|....|....|....|* |  |
| --- | --- | --- | --- |
| ppa003576m | 1950 | TGAATTTGTTCAAGGCCA~AACATGTCGATGGGGGCTGGCCTGGTCCTTTTTGTCCCCTGCCAAGAAGATTGCATCATCTCATGTGGCTGTGAAGAACAA |  |
| PRUAV020955 |  | tgaatttgttcaaggccanaacttgtcgatgggg~ctggcctggtcccttttgccccctgccaagaagattgcatcatctcatgtggctgtgaagaacaa |  |
| PavFIO1_‘Regina’ |  | ~~~~~~~~~~~~~~~~~~~~~~~~~~~~~~~~~~~~~~~~~~~~~~~~~~~~~~~~~~~~~~~~~~~~~~~~~~~~~~~~~~~~~~g~GGGAGGGAGACA |  |
| PavFIO1_‘Lapins’ |  | ~~~~~~~~~~~~~~~~~~~~~~~~~~~~~~~~~~~~~~~~~~~~~~~~~~~~~~~~~~~~~~~~~~~~~~~~~~~~~~~~~~~~~~gtGGCGGGGAGACA |  |
| PavFIO1_‘Garnet’ |  |  |  |
|  |  |  |  |
|  |  | ....|....|....|....|....|....|....|....|....|....|....|....|....|....|....|....|....|....|....|....| |  |
| ppa003576m |  | CCTCTCTTTCATGCTTGAGGTTTCTTTGTCCTACTTTTCTGGTGTTTCAATTTTTTCCAATATCAGAAATGCATCTACCTGATATTCTGAATGTTGCAAA |  |
| PRUAV020955 |  | cctctctttcatgcttgag~~~~~~~~~~~~~~~~~~~~~~~~~~~~~~~~~~~~~~~~~~~~~~~~~~~~~~~~~~~~~~~~~~~~~~~~~~~~~~~~~ |  |
| PavFIO1_‘Regina’ |  | CCTCTCTTTCATGCTTGAGGTTTCTTTGTCCTACTTTTCTATTGTTTCAATTTTTTCCAATTTCAGAAATGCATCTACCTGATATTCTGAATGTTGCAAA |  |
| PavFIO1_‘Lapins’ |  | CCTCTCTTTCATGCTTGAGGTTTCTTTGTCCTACTTTTCTATTGTTTCAATTTTTTCCAATTTCAGAAATGCATCTACCTGATATTCTGAATGTTGCAAA |  |
| PavFIO1_‘Garnet’ |  |  |  |
|  |  |  |  |
|  |  | ....|....|....|....|....|....|....|....|....|....|....|....|....|....|....|....|....|....|....|....| |  |
| ppa003576m |  | TCTGAAGCTATGAGTTCTTAACATGAGGTTAAGGATTCCCCCAAACCCCATGACCCAACAAACACCCACCCATTTGATTGTTTGAATGACCAACCTGCTT |  |
| PRUAV020955 |  | ~~~~~~~~~~~~~~~~~~~~~~~~~~~~~~~~~~~~~~~~~~~~~~~~~~~~~~~~~~~~~~~~~~~~~~~~~~~~~~~~~~~~~~~~~~~~~~~~~~~~ |  |
| PavFIO1_‘Regina’ |  | TCTGAAGCTATGAGTTCTTAAAATGAGGTTAAGGATTCCCCCAAACCCCATGACCCAACAAACACCCACCCATTTGATTGTTTGAATGACCAACCTGCTT |  |
| PavFIO1_‘Lapins’ |  | TCTGAAGCTATGAGTTCTTAAAATGAGGTTAAGGATTCCCCCAAACCCCATGACCCAACAAACACCCACCCATTTGATTGTTTGAATGACCAACCTGCTT |  |
| PavFIO1_‘Garnet’ |  |  |  |
|  |  |  |  |
|  |  | ....|....|....|....|....|....|....|....|....|....|....|....|....|....|....|....|....|....|....|....| |  |
| ppa003576m |  | TTAGAGAAGAGGAGGAACTAGCTTGGTATGAGGGCTGGTGGCAATTATACCATTATTCTAATGTTAACGGGCGACCTCCAAGATTGATTTTATTGAGCTT |  |
| PRUAV020955 |  | ~~~~~~~~~~~~~~~~~~~~~~~~~~~~~~~~~~~~~~~~~~~~~~~~~~~~~~~~~~~~~~~~~~~~~~~~~~~~~~~~~~~~~~~~~~~~~~~~~~~~ |  |
| PavFIO1_‘Regina’ |  | TTAGAGAAGAGGAGGAACTAGCTTGGTATGAGGGCTGGTAGCAATTATACCATTATTCTAATG~~~~~~~~~~~~~~~~~~GATTGATTTTATTGAGCTT |  |
| PavFIO1_‘Lapins’ |  | TTAGAGAAGAGGAGGAACTAGCTTGGTATGAGGGCTGGTAGCAATTATACCATTATTCTAATG~~~~~~~~~~~~~~~~~~GATTGATTTTATTGAGCTT |  |
| PavFIO1_‘Garnet’ |  |  |  |
|  |  |  |  |
|  |  | ....|....|....|....|....|....|....|....|....|....|....|....|....|....|....|....|....|....|....|....| |  |
| ppa003576m |  | TTTATGAGCATGTCAAATTATTGAAAGATGAATAGTTCTTAGTCCTTCCCTGTGCATGTAACTTGCTGAATAGTTCGTATAGCATTATGGCTTCCTTAAA |  |
| PRUAV020955 |  | ~~~~~~~~~~~~~~~~~~~~~~~~~~~~~~~~~~~~~~~~~~~~~~~~~~~~~~~~~~~~~~~~~~~~~~~~~~~~~~~~~~~~~~~~~~~~~~~~~~~~ |  |
| PavFIO1_‘Regina’ |  | TTTATGAGCATGTCAAATTATTGAAAGATGAATAGTTCTTAGTCATTCCCTGTGCATGTAACTTGCTGAATAGTTCGTATAGCATTATGGCTTCCTTAAA |  |
| PavFIO1_‘Lapins’ |  | TTTATGAGCATGTCAAATTATTGAAAGATGAATAGTTCTTAGTCATTCCCTGTGCATGTAACTTGCTGAATAGTTCGTATAGCATTATGGCTTCCTTAAA |  |
| PavFIO1_‘Garnet’ |  |  |  |
|  |  |  |  |
|  |  | ....|....|....|....|....|....|....|....|....|....|....|....|....|....|....|....|....|....|....|....| |  |
| ppa003576m |  | TTTGAATGCCTAGAGCAGTGAGCTGAGAAGGTTTTATTTTACCTTCCATTTTTTATGTCAAAGTATGCACTAGCAGATATCTTTTGTTTCTGTAATAAGC |  |
| PRUAV020955 |  | ~~~~~~~~~~~~~~~~~~~~~~~~~~~~~~~~~~~~~~~~~~~~~~~~~~~~~~~~~~~~~~~~~~~~~~~~~~~~~~~~~~~~~~~~~~~~~~~~~~~~ |  |
| PavFIO1_‘Regina’ |  | TTTGAATGCCAAGAACAGTGAGCTGAAAAGGTTTTATTTTTCCTTCCATTTTTTATGTCAAAGTGTGCACTAGCAGATATCTTTTGTTTCTGTAATAAGC |  |
| PavFIO1_‘Lapins’ |  | TTTGAATGCCAAGAACAGTGAGCTGAAAAGGTTTTATTTTTCCTTCCATTTTTTATGTCAAAGTGTGCACTAGCAGATATCTTTTGTTTCTGTAATAAGC |  |
| PavFIO1_‘Garnet’ |  |  |  |
|  |  |  |  |
|  |  | ....|....|....|....|....|....|....|....|....|....|....|....|....|....|....|....|....|....|....|....| |  |
| ppa003576m |  | AAATATGTGTTTATGTAGGCATACAATGGGTATCAATGATTATTCAGTTACATCACTTCATATCATAATTCTCATCATCTCTTGCGTTTTGTTATCGTCT |  |
| PRUAV020955 |  | ~~~~~~~~~~~~~~~~~~~~~~~~~~~~~~~~~~~~~~~~~~~~~~~~~~~~~~~~~~~~~~~~~~~~~~~~~~~~~~~~~~~~~~~~~~~~~~~~~~~~ |  |
| PavFIO1_‘Regina’ |  | AAATATGTGTTTATGTGGGCATACAATAGGTATCAATGATTATCCAGTTACATCGCTYCACGTCATAATTCTCATCATCTCTCACATTTTGTTATTGTCT |  |
| PavFIO1_‘Lapins’ |  | AAATATGTGTTTATGTGGGCATACAATAGGTATCAATGATTATCCAGTTACATCGCTYCACGTCATAATTCTCATCATCTCTCACATTTTGTTATTGTCT |  |
| PavFIO1_‘Garnet’ |  |  |  |
|  |  |  |  |
|  |  | ....|....|....|....|....|....|....|....|....|....|....|....|....|....|....|....|....|....|....|....| |  |
| ppa003576m |  | TGTTCCTACTAATGTGATGTCTAATCCCACACCTTGTCTACTTTGTAGGGCCTCGACCGAAAATTCAGTGCCATAAATGTATTGCAATCAGTCGAATCCT |  |
| PRUAV020955 |  | ~~~~~~~~~~~~~~~~~~~~~~~~~~~~~~~~~~~~~~~~~~~~~~~~ggcctcgaccgaaaattcagtgccataaatgtattgcaatcagtcgaatcct |  |
| PavFIO1_‘Regina’ |  | TGTTCCTACTAATGTGATGTCTAATTCCACACCTTGTCTACTTTGTAGGGCCTCGACCGAAAATTCAGTGCCATAAATGTATTGCAATCAGTCGAATCCT |  |
| PavFIO1_‘Lapins’ |  | TGTTCCTACTAATGTGATGTCTAATTCCACACCTTGTCTACTTTGTAGGGCCTCGACCGAAAATTCAGTGCCATAAATGTATTGCAATCAGTCGAATCCT |  |
| PavFIO1_‘Garnet’ |  |  |  |
|  |  |  |  |
|  |  | ....|....|....|....|....|....|....|....|....|....|....|....|....|....|....|....|....|....|....|....| |  |
| ppa003576m |  | TTTTCTGCAGTAGTGGTGCATTGTGTAACCTGAAC~ACATCCTCATTTACAGTTGATGTATGCTCATCTGTTAATTTGTTATTCTGTTTATTTATCAAAT |  |
| PRUAV020955 |  | ttttcngcagtagtggtgcattgtgtaacctgaaccacatcctcatttacagttgat~~~~~~~~~~~~~~~~~~~~~~~~~~~~~~~~~~~~~~~~~~~ |  |
| PavFIO1_‘Regina’ |  | TTTTCTGCAGTAGTGGTGCTTTTTAAAAACcgtaaaa |  |
| PavFIO1_‘Lapins’ |  | TTTTCTGCAGTAGTGGTGCTTGTAAAACACccctgaaa |  |
| PavFIO1_‘Garnet’ |  |  |  |

***FKF1***

|  |  | ....|....|....|....|....|....|....|....|....|....|....|....|....|....|....|....|....|....|....|....| |  |
| --- | --- | --- | --- |
| ppa002863m | 1680 | ACGACTCTTGAGGCTGTTTGTTGGAGGAAATTTACAGTTGGAGGTGCCGTGGAGCCTTCACGATGCAATTTCAGTGCTTGTGCTGTAGGAAATCGACTTG |  |
| PRUAV022253 |  | ~~~~~~~~~~~~~~~~~~~~~~~~A~~AAATTTACAGTTGGAGGTGCCGTGGAGCCTTCACGATGCAATTTCAGTGCTTGTGCTGTAGGAAATCGACTTG |  |
| PavFKF1_‘Regina’ |  | ~~~~~~~~~~~~~~~~~~~~~~~~~~~~~~~~gggagttggaGG~GCAGTGGAG~CTTCACGATGCAATTTCAGTGCTTGTGCTGTAGGAAATCGACTTG |  |
| PavFKF1_‘Lapins’ |  | ~~~~~~~~~~~~~~~~~~~~~~~~~~~~~~~~~~agtggggagtgTCTGTGCAGCCTTCACGATGCAATTTCAGTGCTTGTGCTGTAGGAAATCGACTTG |  |
| PavFKF1_‘Garnet’ |  | ~~~~~~~~~~~~~~~~~~~~~~~~~~~~agttggggtaatgcTGTGCCG~~~~~~CTTCACGATGCAATTTCAGTGCTTGTGCTGTAGGAAATCGACTTG |  |
|  |  |  |  |
|  |  | ....|....|....|....|....|....|....|....|....|....|....|....|....|....|....|....|....|....|....|....| |  |
| ppa002863m |  | TACTGTTTGGAGGGGAAGGAGTTGATATGCAGCCAATGGATGACACATTTGTTCTCAATCTTGATGCTGCTGATCCAGAGTGGCGTCGAGTAAGTGTGAA |  |
| PRUAV022253 |  | TACTGTTTGGAGGGGAAGGAGTTGATATGCAGCCAATGGATGACACGTTTGTTCTCAATCTTGATGCTGCCGATCCAGAGTGGCGTCGAGTAAGTGTGAA |  |
| PavFKF1_‘Regina’ |  | TACTGTTTGGAGGGGAAGGAGTTGATATGCAGCCAATGGATGACACGTTTGTTCTCAATCTTGATGCTGCCGATCCAGAGTGGCGTCGAGTAAGTGTGAA |  |
| PavFKF1_‘Lapins’ |  | TACTGTTTGGAGGGGAAGGAGTTGATATGCAGCCAATGGATGACACGTTTGTTCTCAATCTTGATGCTGCCGATCCAGAGTGGCGTCGAGTAAGTGTGAA |  |
| PavFKF1_‘Garnet’ |  | TACTGTTTGGAGGGGAAGGAGTTGATATGCAGCCAATGGATGACACGTTTGTTCTCAATCTTGATGCTGCCGATCCAGAGTGGCGTCGAGTAAGTGTGAA |  |
|  |  |  |  |
|  |  | ....|....|....|....|....|....|....|....|....|....|....|....|....|....|....|....|....|....|....|....| |  |
| ppa002863m |  | ATCATCACCACCAGGGCGTTGGGGCCACACTCTTTCGTGCTTGAATGGTTCTTGGTTGGTAGTTTTTGGAGGCTGTGGGCGGGAAGGATTGCTCAATGAT |  |
| PRUAV022253 |  | ATCATCACCACCAGGGCGTTGGGGCCACACTCTTTCGTGCTTGAATGGTTCTTGGTTGATAGTTTTTGGAGGCTGTGGGCGGGAAGGATTGCTCAATGAT |  |
| PavFKF1_‘Regina’ |  | ATCATCACCACCAGGGCGTTGGGGCCACACTCTTTCGTGCTTGAATGGTTCTTGGTTGATAGTTTTTGGAGGCTGTGGGCGGGAAGGATTGCTCAATGAT |  |
| PavFKF1_‘Lapins’ |  | ATCATCACCACCAGGGCGTTGGGGCCACACTCTTTCGTGCTTGAATGGTTCTTGGTTGATAGTTTTTGGAGGCTGTGGGCGGGAAGGATTGCTCAATGAT |  |
| PavFKF1_‘Garnet’ |  | ATCATCACCACCAGGGCGTTGGGGCCACACTCTTTCGTGCTTGAATGGTTCTTGGTTGATAGTTTTTGGAGGCTGTGGGCGGGAAGGATTGCTCAATGAT |  |
|  |  |  |  |
|  |  | ....|....|....|....|....|....|....|....|....|....|....|....|....|....|....|....|....|....|....|....| |  |
| ppa002863m |  | GTTTTCATTCTTGACTTGGATGCCAAGCAGCCAACATGGAAAGAAGTTTTTGGTGGAACTCCCCCGCTTCCTAGATCCTGGCATAGCTCTTGTACAGTTG |  |
| PRUAV022253 |  | GTTTTCATTCTTGACTTGGATGCCAAGCAGCCAACATGGAAAGAAATTTTTGGTGGAACTCCCCCTCTTCCTAGATCCTGGCATAGCTCTTGCACAGTAG |  |
| PavFKF1_‘Regina’ |  | GTTTTCATTCTTGACTTGGATGCCAAGCAGCCAACATGGAAAGAAATTTTTGGTGGAACTCCCCCTCTTCCTAGATCCTGGCATAGCTCTTGCACAGTAG |  |
| PavFKF1_‘Lapins’ |  | GTTTTCATTCTTGACTTGGATGCCAAGCAGCCAACATGGAAAGAAATTTTTGGTGGAACTCCCCCTCTTCCTAGATCCTGGCATAGCTCTTGCACAGTAG |  |
| PavFKF1_‘Garnet’ |  | GTTTTCATTCTTGACTTGGATGCCAAGCAGCCAACATGGAAAGAAATTTTTGGTGGAACTCCCCCTCTTCCTAGATCCTGGCATAGCTCTTGCACAGTAG |  |
|  |  |  |  |
|  |  | ....|....|....|....|....|....|....|....|....|....|....|....|....|....|....|....|....|....|....|....| |  |
| ppa002863m |  | AAGGGTCTAAATTAGTTGTGTCAGGTGGATGCACAGATGCTGGGGTACTTCTTAGTGACACATACTTATTGGATCTCACTACAGACCACCCAACATGGAA |  |
| PRUAV022253 |  | AAGGTTCTAAATTAGTTGTGTCAGGTGGATGCACAGATGCTGGGGTACTTCTTAGTGACACATACTTATTGGATCTCACTACAGACCACCCAACATGGAA |  |
| PavFKF1_‘Regina’ |  | AAGGTTCTAAATTAGTTGTGTCAGGTGGATGCACAGATGCTGGGGTACTTCTTAGTGACACATACTTATTGGATCTCACTACAGACCACCCAACATGGAA |  |
| PavFKF1_‘Lapins’ |  | AAGGTTCTAAATTAGTTGTGTCAGGTGGATGCACAGATGCTGGGGTACTTCTTAGTGACACATACTTATTGGATCTCACTACAGACCACCCAACATGGAA |  |
| PavFKF1_‘Garnet’ |  | AAGGTTCTAAATTAGTTGTGTCAGGTGGATGCACAGATGCTGGGGTACTTCTTAGTGACACATACTTATTGGATCTCACTACAGACCACCCAACATGGAA |  |
|  |  |  |  |
|  |  | ....|....|....|....|....|....|....|....|....|....|....|....|....|....|....|....|....|....|....|....| |  |
| ppa002863m |  | AGAGATTCCAACTTCATGGGCTCCTCCCTCTAGGTTGGGGCATTCACTTTCAGTTTATGGTCGATCAAAGATTCTCATGTTTGGTGGACTTGCCAACAGT |  |
| PRUAV022253 |  | AGAGATTCCAACTTCATGGGCTCCTCCCTCTAGGTTGGGGCATTCACTTTCAGTTTATGGTCGATCAAAGATTCTCATGTTCGGTGGACTTGCCAACAGT |  |
| PavFKF1_‘Regina’ |  | AGAGATTCCAACTTCATGGGCTCCTCCCTCTAGGTTGGGGCATTCACTTTCAGTTTATGGTCGATCAAAGATTCTCATGTTCGGTGGACTTGCCAACAGT |  |
| PavFKF1_‘Lapins’ |  | AGAGATTCCAACTTCATGGGCTCCTCCCTCTAGGTTGGGGCATTCACTTTCAGTTTATGGTCGATCAAAGATTCTCATGTTCGGTGGACTTGCCAACAGT |  |
| PavFKF1_‘Garnet’ |  | AGAGATTCCAACTTCATGGGCTCCTCCCTCTAGGTTGGGGCATTCACTTTCAGTTTATGGTCGATCAAAGATTCTCATGTTCGGTGGACTTGCCAACAGT |  |
|  |  |  |  |
|  |  | ....|....|....|....|....|....|....|....|....|....|....|....|....|....|....|....|....|....|....|....| |  |
| ppa002863m |  | GGGCACTTGAGGTTACGATCAGGTGAGACTTACACTATTGATTTGGAAGATGAAAACCCTCAGTGGAGGCAACTGGAGTGTAACGCGTTCACCAGCATAG |  |
| PRUAV022253 |  | GGGCACTTGAGGTTACGATCAGGTGAGACTTACACTATTGATTTGGAAGATGAAAACCCTCAGTGGAGGCAACTGGAGTGTAATGCGTTCACCAGCATAG |  |
| PavFKF1_‘Regina’ |  | GGGCCTTTTTGAGGTTA |  |
| PavFKF1_‘Lapins’ |  | GGGCCTTT~~GAGGTTA |  |
| PavFKF1_‘Garnet’ |  | GGGCCTTTT~GAGGTTA |  |

***FL2***

|  |  | ....|....|....|....|....|....|....|....|....|....|....|....|....|....|....|....|....|....|....|....| |  |
| --- | --- | --- | --- |
| ppa006372m | 1 | ACAGTGCCAAAGACTAAAAACAAAGCTAGAGAAGAAGCTGTGGTCTTCATCTGTGTGTGGTTTTGAAACTCTGGGAAAATATGGATCCAGACGCCTTCTC |  |
| PRUAV036733 |  | ~~~~~~~~~~~~~~~~~~~~~~~~~~~~~~~aagaagctgtggtcttcatctgtgtgcggttttcacactctgggaaaatatggatccagacgccttctc |  |
| PavFL2_‘Regina’ |  | ~~~~~~~~~~~~~~~~~~~~~~~~~~~~~~~~~~~~~~~~~~~~~~~~~~~~~~~~~~~~~~~~~~~~~~~~~~~~~~~~~~~~~~~~~~~~~~~~~~~~ |  |
| PavFL2_‘Lapins’ |  |  |  |
| PavFL2_‘Garnet’ |  | ~~~~~~~~~~~~~~~~~~~~~~~~~~~~~~~~~~~~~~~~~~~~~~~~~~~~~~~~~~~~~~~~~~~~~~~~~~~~~~~~~~~~~~~~~~~~~~~~~~~~ |  |
|  |  |  |  |
|  |  | ....|....|....|....|....|....|....|....|....|....|....|....|....|....|....|....|....|....|....|....| |  |
| ppa006372m |  | AGCGAGCCTCTTCAAGTGGGACCTACGAGGCATGGTTGTTCCGCCGAGCCGGGCTCAGCTAGAAGCCGCCGTGACGCCTCAAGCTGCCGCTGCAGCTGCG |  |
| PRUAV036733 |  | agcgaacctcttcaagtgggacctacgaggcatggttgttccgcccacccgggctcagctagaagcctccgtgacgcctcaagctgccgctgcagctgcg |  |
| PavFL2_‘Regina’ |  | ~~~~~~~~~~~~~~~~~~~~~~~~~~~~~~~~~~~~~~~~~~~~~~~GCGGGGCT~~GCTAG~AGCCTCCGTGACGCCTCAAGCTGCCGCTGCAGCTGCG |  |
| PavFL2_‘Lapins’ |  |  |  |
| PavFL2_‘Garnet’ |  | ~~~~~~~~~~~~~~~~~~~~~~~~~~~~~~~~~~~~~~~~~~~~CGGCCCAGGCCCTTTTTGAAG~CTCCGTGACGCCTCA~GCTGCCGCTGCAGCTGCG |  |
|  |  |  |  |
|  |  | ....|....|....|....|....|....|....|....|....|....|....|....|....|....|....|....|....|....|....|....| |  |
| ppa006372m |  | GCTTACGCTGCTGTGAGGCCGCAGAGAGAG~~CTCGGAGGGCTTGAGGACTTGTTCCAGGCTTATGGGG~TCAGATACTACACGGCAGCGAAGATAGCCG |  |
| PRUAV036733 |  | gcttacgctgccgtgaggccgccgagagagnncttggagggcttgaggacttgttccaggcttatgggggtcagatactacacggcggcgaagatagccg |  |
| PavFL2_‘Regina’ |  | GCTTACGCTGCCGTGAGGCCGCCGAGAGAG~~CTYGGAGGGCTTGAGGACTTGTTCCAGGCTTAT~GGGGTCAGATACTACACGGCGGCGAAGATAGCCG |  |
| PavFL2_‘Lapins’ |  |  |  |
| PavFL2_‘Garnet’ |  | GCTTACGCTGCCGTGAGGCCGCCGAGAGAG~~CTCGGAGGGCTTGAGGACTTGTTCCAGGCTTAT~GGGGTCAGATACTACACGGCGGCGAAGATAGCCG |  |
|  |  |  |  |
|  |  | ....|....|....|....|....|....|....|....|....|....|....|....|....|....|....|....|....|....|....|....| |  |
| ppa006372m |  | AGCTCGGCTTTACTGTGAACACCCTTTTGGATATGAGGGACGGTGAGCTTGACGACATGATGAGTAGCCTCTCTCAGATATTCAGGTGGGATTTGCTTGT |  |
| PRUAV036733 |  | agctcggctttactgtcaacacccttttggatatgagggacggtgagcttgacgacatgatgagtagcctctctcagatattcaggtgggatttgcttgt |  |
| PavFL2_‘Regina’ |  | AGCTCGGCTTTACTGTCAACACCCTTTTGGATATGAGGGACGGTGAGCTTGACGACATGATGAGTAGCCTCTCTCAGATATTCAGGTGGGATTTGCTTGA |  |
| PavFL2_‘Lapins’ |  |  |  |
| PavFL2_‘Garnet’ |  | AGCTCGGCTTTACTGTCAACACCCTTTTGGATATGAGGGACGGTGAGCTTGACGACATGATGAGTAGCCTCTCTCAGATATTCAGGTGGGATTTGCTTGA |  |
|  |  |  |  |
|  |  | ....|....|....|....|....|....|....|....|....|....|....|....|....|....|....|....|....|....|....|....| |  |
| ppa006372m |  | GGGTGAGAGGTACGGTATC~AAAGCCGCCGTCAGAGCAGAGCGTCGTCGCCTCGATGACGAGGACTCGAGGCGGCGCCACACCTTCTCCGGCGACACCAC |  |
| PRUAV036733 |  | gggtgagaggtacggtatcgaaagcggccgtcagagccgagcgtcgccgactcgatgaccaggactcgaggcgccgccccgtc |  |
| PavFL2_‘Regina’ |  | A |  |
| PavFL2_‘Lapins’ |  |  |  |
| PavFL2_‘Garnet’ |  | A |  |

***FLD***

|  |  | ....|....|....|....|....|....|....|....|....|....|....|....|....|....|....|....|....|....|....|....| |  |
| --- | --- | --- | --- |
| ppa001272m | 1 | GGGAACCAAATAGTCCATTTCATTCTTCTCCTTCACCACCACTACCCCAACTAGTACAGACGGCGATGGATCCACCTAACGAATTCCCCGATGATTTTTC |  |
| PRUAV017206 |  | gggaaccaaacagttcatttcattcttctccttcaccaccactaccccaaccagtacagacggcgatggatccacctaacgaattccctgatgatttttc |  |
| PavFLD_‘Regina’ |  | ~~~~~~~~~~~~~~~~~~~~~~~~~~~~~~~~~~~~~~~~~~~~~~~~~~~~~~~~~~~~~~~~~~~~~~~~~~~~~~~~~~~~~~~~~~~~~~~~~~~~ |  |
| PavFLD_‘Lapins’ |  | ~~~~~~~~~~~~~~~~~~~~~~~~~~~~~~~~~~~~~~~~~~~~~~~~~~~~~~~~~~~~~~~~~~~~~~~~~~~~~~~~~~~~~~~~~~~~~~~~~~~~ |  |
| PavFLD_‘Garnet’ |  | ~~~~~~~~~~~~~~~~~~~~~~~~~~~~~~~~~~~~~~~~~~~~~~~~~~~~~~~~~~~~~~~~~~~~~~~~~~~~~~~~~~~~~~~~~~~~~~~~~~~~ |  |
|  |  |  |  |
|  |  | ....|....|....|....|....|....|....|....|....|....|....|....|....|....|....|....|....|....|....|....| |  |
| ppa001272m |  | TTCGTTCCCTCCAATCCCGTTCGCCCTCTTTGTTCCCCCAGAAAACCCTAACCCCAATTCAAACGCAGCGCCAATTCCAAATACAGTCGAAAA~CCCTAG |  |
| PRUAV017206 |  | ttcgttccctccaatcccgttcgccctctttgttcccccagaaaaccctaaccccaattcaaacgcagcgccaattccaaatacagtcgaaaaaccctag |  |
| PavFLD_‘Regina’ |  | ~~~~~~~~~~~~~~~~~~~~~~~~~~~~~~~~~~~~~~~~~~~~~~~~~~~~~~~~~~~~~~~~~~~~~~~~~~~~~~~~~~~~~~~~~~~~~~~~~~~~ |  |
| PavFLD_‘Lapins’ |  | ~~~~~~~~~~~~~~~~~~~~~~~~~~~~~~~~~~~~~~~~~~~~~~~~~~~~~~~~~~~~~~~~~~~~~~~~~~~~~~~~~~~~~~~~~~~~~~~~~~~~ |  |
| PavFLD_‘Garnet’ |  | ~~~~~~~~~~~~~~~~~~~~~~~~~~~~~~~~~~~~~~~~~~~~~~~~~~~~~~~~~~~~~~~~~~~~~~~~~~~~~~~~~~~~~~~~~~~~~~~~~~~~ |  |
|  |  |  |  |
|  |  | ....|....|....|....|....|....|....|....|....|....|....|....|....|....|....|....|....|....|....|....| |  |
| ppa001272m |  | CTCAGCTCACCTTCTTTCCTTTTCGGTCCCCAAGAAACGAAGACGAGGCAGGCCTCATAGGGTTCCGACGTCGTTTCAGTTACCTCCAATTCCCAACGGC |  |
| PRUAV017206 |  | ctcagctcaccttctttccttctcggtccccaagaaacgaagacgaggcaggcctcatagggttccgacgtcgtttcagttacctccaattcccaacggc |  |
| PavFLD_‘Regina’ |  | ~~~gTCT~AC~TTCTTTC~TTCTCGGTCCCCAAGAAACGAAGACGAGGCAGGCCTCATAGGGTTCCGACGTCGTTTCAGTTACCTCCAATTCCCAACGGC |  |
| PavFLD_‘Lapins’ |  | ~~~~ac~~~ctTTCTTTCCTTCTCGGTCCCCAAGAAACGAAGACGAGGCAGGCCTCATAGGGTTCCGACGTCGTTTCAGTTACCTCCAATTCCCAACGGC |  |
| PavFLD_‘Garnet’ |  | ~~CAGCT~AC~TTCTTTCCTTCTCGGTCCCCAAGAAACGAAGACGAGGCAGGCCTCATAGGGTTCCGACGTCGTTTCAGTTACCTCCAATTCCCAACGGC |  |
|  |  |  |  |
|  |  | ....|....|....|....|....|....|....|....|....|....|....|....|....|....|....|....|....|....|....|....| |  |
| ppa001272m |  | GTTTTCAACAGTAATAACAACGGTCTTGCTTCTTTTTCCAGTTCAATTTCAGCTCATTCCTCTAGAAACAATGTAGAAATCCCT~GGTTCTTCGGCAAGA |  |
| PRUAV017206 |  | gttttcaacagtaataacaacggtcttgcttctttttccagttcaatttcagctcattcctctagaaataatgtagaaatccctaggttcttcggcaaga |  |
| PavFLD_‘Regina’ |  | GTTTTCAACAGTAATAACAACGGTCTTGCTTCTTTTTCCAGTTCAATTTCAGCTCATTCCTCTAGAAATAATGTAGAAATCCCT~GGTTCTTCGGCAAGA |  |
| PavFLD_‘Lapins’ |  | GTTTTCAACAGTAATAACAACGGTCTTGCTTCTTTTTCCAGTTCAATTTCAGCTCATTCCTCTAGAAATAATGTAGAAATCCCT~GGTTCTTCGGCAAGA |  |
| PavFLD_‘Garnet’ |  | GTTTTCAACAGTAATAACAACGGTCTTGCTTCTTTTTCCAGTTCAATTTCAGCTCATTCCTCTAGAAATAATGTAGAAATCCCT~GGTTCTTCGGCAAGA |  |
|  |  |  |  |
|  |  | ....|....|....|....|....|....|....|....|....|....|....|....|....|....|....|....|....|....|....|....| |  |
| ppa001272m |  | ACCATGCCTGATATGTCCGACGAGATCATCGTGATCAATAAGGAGTCCACGGCGGAGGCGCTGATCGCGCTGTCGGCTGGTTTTCCTGCTGATTCGCTCA |  |
| PRUAV017206 |  | accatgcctgatatgtccgacgagatcatcgtgatcaataaggagtccacggcggaggcgctgatcgcgctgtcggcgggttttcctgctgattcgctca |  |
| PavFLD_‘Regina’ |  | ACCATGCCTGATATGTCCGACGAGATCATCGTGATCAATAAGGAGTCCACGGCGGAGGCGCTGATCGCGCTGTCGGCGGGTTTTCCTGCTGATTCGCTCA |  |
| PavFLD_‘Lapins’ |  | ACCATGCCTGATATGTCCGACGAGATCATCGTGATCAATAAGGAGTCCACGGCGGAGGCGCTGATCGCGCTGTCGGCGGGTTTTCCTGCTGATTCGCTCA |  |
| PavFLD_‘Garnet’ |  | ACCATGCCTGATATGTCCGACGAGATCATCGTGATCAATAAGGAGTCCACGGCGGAGGCGCTGATCGCGCTGTCGGCGGGTTTTCCTGCTGATTCGCTCA |  |
|  |  |  |  |
|  |  | ....|....|....|....|....|....|....|....|....|....|....|....|....|....|....|....|....|....|....|....| |  |
| ppa001272m |  | CCGAGGAGGAAATTGATTTCGGGGTAATTCGAGTTATTGGGGGCATAGAGCAGGTCAATTACATTCTCATTAGGAACCACATAATTGCTAAATGGCGTGA |  |
| PRUAV017206 |  | ccgaggaggaaatcgatttcggggtaattcgagttattgggggcatagagcaagtcaattacattctcattaggaaccacataattgctaaatggcgtga |  |
| PavFLD_‘Regina’ |  | CCGAGGAGGAAATCGATTTCGGGGTAATTCGAGTTATTGGGGGCATAGAGCAAGTCAATTACATTCTCATTAGGAACCACATAATTGCTAAATGGCGTGA |  |
| PavFLD_‘Lapins’ |  | CCGAGGAGGAAATCGATTTCGGGGTAATTCGAGTTATTGGGGGCATAGAGCAAGTCAATTACATTCTCATTAGGAACCACATAATTGCTAAATGGCGTGA |  |
| PavFLD_‘Garnet’ |  | CCGAGGAGGAAATCGATTTCGGGGTAATTCGAGTTATTGGGGGCATAGAGCAAGTCAATTACATTCTCATTAGGAACCACATAATTGCTAAATGGCGTGA |  |
|  |  |  |  |
|  |  | ....|....|....|....|....|....|....|....|....|....|....|....|....|....|....|....|....|....|....|....| |  |
| ppa001272m |  | AAATGTGTCGAATTGGGTCACAAAAGATATATTTATTGATTCTATACCTAAACATTGTCATTCCCTGTTGGATTCTACTTATAAATATCTGGTTTCGCAT |  |
| PRUAV017206 |  | Aaatgtgtcgaattgggtcacaaaagatatatttattgattctatacctaaacattgtcattccctgttgaattctacttataaatatctggtttcgcat |  |
| PavFLD_‘Regina’ |  | AAATGTGTCGAATTGGTTCACAAAAA |  |
| PavFLD_‘Lapins’ |  | AAATGTGTCGAATTGGGTCACAAAAATGATGAGAATTTGATGGATGATTTCATAGAGAAGCATTTGGGTGGTACTTTTGAAGAAAAAGGAATAAAACCaa |  |
| PavFLD_‘Garnet’ |  | AAATGTGTCGAATTGGGTCACAAAAA |  |
|  |  |  |  |
|  |  | ....|....|....|....|....|....|....|....|....|....|....|....|....|....|....|....|....|....|....|....| |  |
| ppa001272m |  | GGTTATATTAACTTTGGGGTTGCTCCGGCTATCAAAGAGAAGATTCCGGCTGAACCCAGTAAACCAC~ATGTGATT~GTGATTGGTGCTGGGCTTGCTGG |  |
| PRUAV017206 |  | ggttatattaactttggg~ttnctccggctatcaaagagaagattccggctgaacccagtaaaccacnatgtgatttgtgattggtgctgggcttgctgg |  |
| PavFLD_‘Regina’ |  |  |  |
| PavFLD_‘Lapins’ |  | ggaggatttggaaaaaaaaaccaaatttgaggccttgatacctttgcccctggaatccccctttgataaccttgaaagaaagaaaa |  |
| PavFLD_‘Garnet’ |  |  |  |

***FRI***

|  |  | ....|....|....|....|....|....|....|....|....|....|....|....|....|....|....|....|....|....|....|....| |
| --- | --- | --- |
| ppa004224m | 1701 | TGTGCAGATATGATTGAAGAATTAATCAGCAGGGGACAACAGCTTGATGCGGTACATTTCACTTATGAAGTTGGACTTGTGCACAAGTTTCCTCCTGTTC |
| PRUAV000207 |  | ~~~~~~~ATATGATTGAAGAATTAATCAGCAGGGGACAACAGCTTGATGCGGTACATTTCACTTATGAAGTTGGACTTGTGCACAAGTTTCCTCCTGTTC |
| PavFRI_‘Regina’ |  | ~~~~~~~~~~~~~~~~~~~~~~~~~~~~~~~~~~~~~~~~~~~~~~~~~~~~~~~~~~~~~~~caggGGGCTTGA~TTGTGCAC~AGTTTCCTCCTGTTC |
| PavFRI_‘Lapins’ |  | ~~~~~~~~~~~~~~~~~~~~~~~~~~~~~~~~~~~~~~~~~~~~~~~~~~~~~~~~~~~~~~gctggaggtTGGACTTGTGCACAAGTTTCCTCCTGTTC |
| PavFRI_‘Garnet’ |  | ~~~~~~~~~~~~~~~~~~~~~~~~~~~~~~~~~~~~~~~~~~~~~~~~~~~~~~~~~~~~~~agtagaactt~ga~tTGTGCAC~AGTTTCCTCCTGTTC |
|  |  |  |
|  |  | ....|....|....|....|....|....|....|....|....|....|....|....|....|....|....|....|....|....|....|....| |
| ppa004224m |  | CTCTGCTGAAAGCTTTTCTGAAAGATGCTAAGAAAGCTGCAGCTTCTATTATGGAAGATCCCAATAATGCTGGTCGAGCTGCGGTATGATTTTAATCGTT |
| PRUAV000207 |  | CTCTGCTGAGAGCTTTTCTGAAAGATGCTAAGAAAGCTGCAGCTTCTATTATGGAAGATCCCAATAATGCTGGTCGAGCTGCG~~~~~~~~~~~~~~~~~ |
| PavFRI_‘Regina’ |  | CTCTGCTGAGAGCTTTTCTGAAAGATGCTAAGAAAGCTGCAGCTTCTATTATGGAAGATCCCAATAATGCTGGTCGAGCTGCGGTATGATTTTAATCATT |
| PavFRI_‘Lapins’ |  | CTCTGCTGAGAGCTTTTCTGAAAGATGCTAAGAAAGCTGCAGCTTCTATTATGGAAGATCCCAATAATGCTGGTCGAGCTGCGGTATGATTTTAATCATT |
| PavFRI_‘Garnet’ |  | CTCTGCTGAGAGCTTTTCTGAAAGATGCTAAGAAAGCTGCAGCTTCTATTATGGAAGATCCCAATAATGCTGGTCGAGCTGCGGTATGATTTTAATCATT |
|  |  |  |
|  |  | ....|....|....|....|....|....|....|....|....|....|....|....|....|....|....|....|....|....|....|....| |
| ppa004224m |  | GCACCCTATTGGTGTTGCTTGATCCTGCATGTTGTGACTTTCAGTCGTGAATACGCTTCTCAAAGTGGTGTCTGGTTCTTTCTTTACAACATTTCAAATT |
| PRUAV000207 |  | ~~~~~~~~~~~~~~~~~~~~~~~~~~~~~~~~~~~~~~~~~~~~~~~~~~~~~~~~~~~~~~~~~~~~~~~~~~~~~~~~~~~~~~~~~~~~~~~~~~~~ |
| PavFRI_‘Regina’ |  | GCACCGTATTGGTGTTGCATGATCCTGCATGTTGTGACTTTCAGTCGTGAATATGCTTTTCAAAGCGTTGTCTGGTTCTTTCTTTACAACATTTCAAATT |
| PavFRI_‘Lapins’ |  | GCACCGTATTGGTGTTGCATGATCCTGCATGTTGTGACTTTCAGTCGTGAATATGCTTTTCAAAGYGTTGTCTGGTTCTTTCTTTACAACATTTCAAATT |
| PavFRI_‘Garnet’ |  | GCACCGTATTGGTGTTGCATGATCCTGCATGTTGTGACTTTCAGTCGTGAATATGCTTTTCAAAGCGTTGTCTGGTTCTTTCTTTACAACATTTCAAATT |
|  |  |  |
|  |  | ....|....|....|....|....|....|....|....|....|....|....|....|....|....|....|....|....|....|....|....| |
| ppa004224m |  | CCAGTTATGTGTCTGTGGGTGGGGGAGAGAGGGGGTTGAATCCCCTTCCTACCTTCTCATTCCCACAGATCCCTGTTTTTTT~TCCCTTCTCAAATGATT |
| PRUAV000207 |  | ~~~~~~~~~~~~~~~~~~~~~~~~~~~~~~~~~~~~~~~~~~~~~~~~~~~~~~~~~~~~~~~~~~~~~~~~~~~~~~~~~~~~~~~~~~~~~~~~~~~~ |
| PavFRI_‘Regina’ |  | CCAGTTATGTGTCTGTGGGTGGGGCAGAGAGGGGGTTGAATTCCCTTCCCGCCTTCTCATTCCCACAAATCCCTGTTTTTTTCTCCCTTCTCAAATGATT |
| PavFRI_‘Lapins’ |  | CCAGTTATGTGTCTGTGGGTGGGGCAGAGAGGGGGTTGAATTCCCTTCCCGCCTTCTCATTCCCACAAATCCCTGTTTTTTTCTCCCTTCTCAAATGATT |
| PavFRI_‘Garnet’ |  | CCAGTTATGTGTCTGTGGGTGGGGCAGAGAGGGGGTTGAATTCCCTTCCCGCCTTCTCATTCCCACAAATCCCTGTTTTTTTCTCCCTTCTCAAATGATT |
|  |  |  |
|  |  | ....|....|....|....|....|....|....|....|....|....|....|....|....|....|....|....|....|....|....|....| |
| ppa004224m |  | GGGAGGGGAGAGATAGAGGGAGAGATGGAGGGAGA~~GGGGAAAGAAAAGGGAATGTGAAAGGAACTTTTCCCTTGTTTGTGTTTTAAGGAAGGTAGAAA |
| PRUAV000207 |  | ~~~~~~~~~~~~~~~~~~~~~~~~~~~~~~~~~~~~~~~~~~~~~~~~~~~~~~~~~~~~~~~~~~~~~~~~~~~~~~~~~~~~~~~~~~~~~~~~~~~~ |
| PavFRI_‘Regina’ |  | GGGAGGGGAGA~~TAGAGGGAGAGATGGAGGGAGAGAGGGGAAAGAAAAGGGAATGTGAAAGGAACTTTTCCCTTGTTTGTGTTTTAAGGAAGGTAGAAA |
| PavFRI_‘Lapins’ |  | GGGAGGGGAGA~~TAGAGGGAGAGATGGAGGGAGAGAGGGGAAAGAAAAGGGAATGTGAAAGGAACTTTTCCCTTGTTTGTGTTTTAAGGAAGGTAGAAA |
| PavFRI_‘Garnet’ |  | GGGAGGGGAGA~~TAGAGGGAGAGATGGAGGGAGAGAGGGGAAAGAAAAGGGAATGTGAAAGGAACTTTTCCCTTGTTTGTGTTTTAAGGAAGGTAGAAA |
|  |  |  |
|  |  | ....|....|....|....|....|....|....|....|....|....|....|....|....|....|....|....|....|....|....|....| |
| ppa004224m |  | GGGGGATTCCCCCACCCCCACAAAAAGCTCTCTCCTCTAATGGAGGAGAGGATGGTAGCCATCTACAAAAAATACATAATATCAACATCTTACCCTATAT |
| PRUAV000207 |  | ~~~~~~~~~~~~~~~~~~~~~~~~~~~~~~~~~~~~~~~~~~~~~~~~~~~~~~~~~~~~~~~~~~~~~~~~~~~~~~~~~~~~~~~~~~~~~~~~~~~~ |
| PavFRI_‘Regina’ |  | GGGGGATTCCCCCACCCCCACAAAAAGCTTTCTCCTCTAATGGAG~~~AGGATGGTAGCCATCTACAAAAAATACATAATATCAACATCTTACCCTATAT |
| PavFRI_‘Lapins’ |  | GGGGGATTCCCCCACCCCCACAAAAAGCTTTCTCCTCTAATGGAG~~~AGGATGGTAGCCATCTACAAAAAATACTTAATATCAACATCTTACCCTATAT |
| PavFRI_‘Garnet’ |  | GGGGGATTCCCCCACCCCCACAAAAAGCTTTCTCCTCTAATGGAG~~~AGGATGGTAGCCATCTACAAAAAATACATAATATCAACATCTTACCCTATAT |
|  |  |  |
|  |  | ....|....|....|....|....|....|....|....|....|....|....|....|....|....|....|....|....|....|....|....| |
| ppa004224m |  | TAATTATTACATGTGAGTGAAAAGTCAATGCCTAAATTTCCACTGTCCGTTTCAAACATGACGAAATTTATTCCATGCAGTTCTTCCTATTTCATTTCAT |
| PRUAV000207 |  | ~~~~~~~~~~~~~~~~~~~~~~~~~~~~~~~~~~~~~~~~~~~~~~~~~~~~~~~~~~~~~~~~~~~~~~~~~~~~~~~~~~~~~~~~~~~~~~~~~~~~ |
| PavFRI_‘Regina’ |  | TAATTATTACATGTGAGTGAAAAGTCAATGTCTAAATTTCCACTCTCCGTTTCAAACATGACGAAATTTCCTCCATGCAATTCTTCCTATTTCATTTCAT |
| PavFRI_‘Lapins’ |  | TAATTATTACATGTGAGTGAAAAGTCAATGTCTAAATTTCCACTCTCCGTTTCAAACATGACGAAATTTCCTCCATGCAATTCTTCCTATTTCATTTCAT |
| PavFRI_‘Garnet’ |  | TAATTATTACATGTGAGTGAAAAGTCAATGTCTAAATTTCCACTCTCCGTTTCAAACATGACGAAATTTCCTCCATGCAATTCTTCCTATTTCATTTCAT |
|  |  |  |
|  |  | ....|....|....|....|....|....|....|....|....|....|....|....|....|....|....|....|....|....|....|....| |
| ppa004224m |  | ATTCATGCTTGCTCTCATAGTGTTTATCCCTGGTTTTTATCTTATTGTTGGAACAAGTATATATACTGAAAAGTTTTGGGACAGCAAACTCCTGCTATTG |
| PRUAV000207 |  | ~~~~~~~~~~~~~~~~~~~~~~~~~~~~~~~~~~~~~~~~~~~~~~~~~~~~~~~~~~~~~~~~~~~~~~~~~~~~~~~~~~~~~~~~~~~~~~~~~~~~ |
| PavFRI_‘Regina’ |  | ATTCATGCTTGCTCTCAGAGTTTTTATCCCCGGTTTTTATCTTATTGTTGGAAAAAGTATATATACTGAAAAGTTTTGGGACAACAAACTCCTGCTATTG |
| PavFRI_‘Lapins’ |  | ATTCATGCTTGCTCTCAGAGTTTTTATCCCCGGTTTTTATCTTATTGTTGGAAAAAGTATATATACTGAAAAGTTTTGGGACAACAAACTCCTGCTATTG |
| PavFRI_‘Garnet’ |  | ATTCATGCTTGCTCTCAGAGTTTTTATCCCCGGTTTTTATCTTATTGTTGGAAAAAGTATATATACTGAAAAGTTTTGGGACAACAAACTCCTGCTATTG |
|  |  |  |
|  |  | ....|....|....|....|....|....|....|....|....|....|....|....|....|....|....|....|....|....|....|....| |
| ppa004224m |  | GAGTGGAATTTGATTTCCACTTTATGTGTCAATTAACTGGTGAAGCAGTAGGAATAATCGGGTATAATACATCTTGTTATGGAGGTCTTGATGATGAGGC |
| PRUAV000207 |  | ~~~~~~~~~~~~~~~~~~~~~~~~~~~~~~~~~~~~~~~~~~~~~~~~~~~~~~~~~~~~~~~~~~~~~~~~~~~~~~~~~~~~~~~~~~~~~~~~~~~~ |
| PavFRI_‘Regina’ |  | GAGTGGAATTTGATTTCCACCTTATGTGTCAATTAACTGGTGAAGCAGTAGGAATAATCAGGCATAATACATCTTGTTATGGATGTCTTGATGATGAGGC |
| PavFRI_‘Lapins’ |  | GAGTGGAATTTGATTTCCACCTTATGTGTCAATTAACTGGTGAAGCAGTAGGAATAATCAGGCATAATACATCTTGTTATGGATGTCTTGATGATGAGGC |
| PavFRI_‘Garnet’ |  | GAGTGGAATTTGATTTCCACCTTATGTGTCAATTAACTGGTGAAGCAGTAGGAATAATCAGGCATAATACATCTTGTTATGGATGTCTTGATGATGAGGC |
|  |  |  |
|  |  | ....|....|....|....|....|....|....|....|....|....|....|....|....|....|....|....|....|....|....|....| |
| ppa004224m |  | ATTCTGCAGTCATTCACATCCTTGTGTCTCATTTCTGATTTCTTCGGAAAAATGTCCTTAGAAGTGCATGAACTTCAGTCTCCTTTCCTCATATGCAGAA |
| PRUAV000207 |  | ~~~~~~~~~~~~~~~~~~~~~~~~~~~~~~~~~~~~~~~~~~~~~~~~~~~~~~~~~~~~~~~~~~~~~~~~~~~~~~~~~~~~~~~~~~~~~~~~~~~~ |
| PavFRI_‘Regina’ |  | ATTCTGCAGTCATTCACATCCTTGTGTCTCATTTGTGATTTCTTTGGAAAAATGTCCTTAGAAGTGCATGAACTTCAGTCTCCTTTCCTCATATGCAGAA |
| PavFRI_‘Lapins’ |  | ATTCTGCAGTCATTCACATCCTTGTGTCTCATTTCTGATTTCTTTGGAAAAATGTCCTTAGAAGTGCATGAACTTCAGTCTCCTTTCCTCATATGCAGAA |
| PavFRI_‘Garnet’ |  | ATTCTGCAGTCATTCACATCCTTGTGTCTCATTTSTGATTTCTTTGGAAAAATGTCCTTAGAAGTGCATGAACTTCAGTCTCCTTTCCTCATATGCAGAA |
|  |  |  |
|  |  | ....|....|....|....|....|....|....|....|....|....|....|....|....|....|....|....|....|....|....|....| |
| ppa004224m |  | TAATCAAACCTGTAGAAACCTGCGTTTTCATTTGATCACTAATAGTTTCTTTATTACTTTCTGTGCAGAACCTAGCTGGACGCAAAGAGCAGTCAGCACT |
| PRUAV000207 |  | ~~~~~~~~~~~~~~~~~~~~~~~~~~~~~~~~~~~~~~~~~~~~~~~~~~~~~~~~~~~~~~~~~~~~AACCTAGCTGGACGCAAAGAGCAGTCAGCACT |
| PavFRI_‘Regina’ |  | TAATCAAACCTGTAGAAACCTGCGTTTTCATTTGATCACTAATATTTTCTTTATTACTTTCTGTGCAGAACCTAGCTGGACGCAAAGAGCAGTCAGCACT |
| PavFRI_‘Lapins’ |  | TAATCAAACCTGTAGAAACCTGCGTTTTCATTTGATCACTAATATTTTCTTTATTACTTTCTGTGCAGAACCTAGCTGGACGCAA~GAGCAGTCAGCACT |
| PavFRI_‘Garnet’ |  | TAATCAAACCTGTAGAAACCTGCGTTTTCATTTGATCACTAATATTTTCTTTATTACTTTCTGTGCAGAACCTAGCTGGACGCAA~GAGCAGTCAGCACT |
|  |  |  |
|  |  | ....|....|....|....|....|....|....|....|....|....|....|....|....|....|....|....|....|....|....|....| |
| ppa004224m |  | CCGAGCTGTCGTCAAGTGTATTGAAGATTATAAACTTGAGGCAGAATTTCCTCCAGAAAACCTCAAGAAGCGCCTTGAGCAGCTAGAGAAGGTGAAACCA |
| PRUAV000207 |  | CCGAGCTGTCATCAAGTGTATTGAAGATTACAAACTTGAGGCAGAATTTCCTCCAGAAAACCTCAAGAAGCGCCTTGAGCAGCTAGAGAAGGTGAAACCA |
| PavFRI_‘Regina’ |  | CCGAGCTGTCATCA~GTGTATTGAAGATTACAa~cttgaggcaga~tttccttcagatatcttca~gaagcgtcttgagcagctagaga~g~tgaaac~a |
| PavFRI_‘Lapins’ |  | CCGAGCTGTCATCAAGTGTATTGAAGATtacaaacttgaggcaga~tttc~~tcagaaa~ct~ca~gaagcgccttgagcagctagaga~ggtgaaac~a |
| PavFRI_‘Garnet’ |  | CCGAGCTGTCATCAAGTGTATTGA~GAttacaaacttgag~caga~tttc~~tcagaaaact~ca~ga~gcgcct~gagcagctagaga~g~tgaa~c~a |
|  |  |  |
|  |  | ....|....|....|....|....|....|....|....|....|....|....|....|....|....|....|....|....|....|....|....| |
| ppa004224m |  | GAGAAGAAAAGGCCAGCTGCAGTCCCTGCCAACAAACGAACACGAGCCAACAATGGAGGTCCCATGCCTCCAGCCAAGGCTGGACGCTTGACGAATGCAT |
| PRUAV000207 |  | GAGAAGAAAAGGCCAGCTGCAGTCCCTGCCAACAAACGAACACGAGCCAACAATGGAGGTCCCATGCCTCCAGCCAAGGCTGGACGCTTGACGAATGCAT |
| PavFRI_‘Regina’ |  | gagaagaaaagtc~agctgcagtcc~tgc~~ac~aacga~c~cgagc~~acaatgtggtggagggtctcctatgatgg |
| PavFRI_‘Lapins’ |  | gagaagaaaaggccagctgcagtcc~tgc~~acc~acga~cacgagccaccagtggtggaggaggtctacagatgaa |
| PavFRI_‘Garnet’ |  | gagaagaaa~gtc~agctgcagtcc~tgc~~acaaacgaacacgagc~~acagaagtgaggaggagagctcatatgacct |

***FRS6***

|  |  | ....|....|....|....|....|....|....|....|....|....|....|....|....|....|....|....|....|....|....|....| |  |
| --- | --- | --- | --- |
| ppa002486m | 777 | TTTCTGTGATATAGTTGCCATTGACGCTACATGCTTGGAAAACAAGTTTGAAGTCCCGCTGGTGTCATTTATTGGAGTAAATCATCACGGACAATCTGTG |  |
| PRUAV011091 |  | ~~~~~~~~~~ATAGTTGCCATTGACACTACATGCTTGGAAAACAAGTTTGAAGTCCCGCTGGTGTCATTTACTGGAGTAAATCATCATGGACAATCTGTG |  |
| PavFRS6_‘Regina’ |  | ~~~~~~~~~~~~~~~~~~~~~~~~~~~~~~~~~~~~~~~~~~~~~~~~~~~~~~~~~~~~~~~~~~~~~~~~~~~~~~~~~~~~~~~~~~~~~~~~~~~~ |  |
| PavFRS6_‘Lapins’ |  | ~~~~~~~~~~~~~~~~~~~~~~~~~~~~~~~~~~~~~~~~~~~~~~~~~~~~~~~~~~~~~~~~~~~~~~~~~~~~~~~~~~~~~~~~~~~~~~~~~~~~ |  |
| PavFRS6_‘Garnet’ |  | ~~~~~~~~~~~~~~~~~~~~~~~~~~~~~~~~~~~~~~~~~~~~~~~~~~~~~~~~~~~~~~~~~~~~~~~~~~~~~~~~~~~~~~~~~~~~~~~~~~~~ |  |
|  |  |  |  |
|  |  | ....|....|....|....|....|....|....|....|....|....|....|....|....|....|....|....|....|....|....|....| |  |
| ppa002486m |  | CTGCTAGGCTGTGGTTTACTTGCAAGTGAGACAGTTGAATCCTATACATGGTTGTTTAGAGCTTGGCTCACATGCATACTGGGACGCCCTCCTCAAGCGA |  |
| PRUAV011091 |  | CTGCTAGGCTGTGGTTTACTTGCAAGTGAGACAGTTGAATCCTATACATGGTTGTTTAGAGCTTGGCTTACATGCATATTGGGACGCCCTCCTCAAGCGA |  |
| PavFRS6_‘Regina’ |  | ~~~~~~~~~~TTCTTTTTCTGTTTGAGTTGCCTCTCTCCTCTCCTTTCTGGTTGTTTAGAGCTTGGCTTACATGCATATTGGGACGCCCTCCTCAAGCGA |  |
| PavFRS6_‘Lapins’ |  | ~~~~~~~~~~TTTCCGCATTTCTTGTAATGGAGGTTGA~TCCTATACATGGTTGTTTAGAGCTTGGCTTACATGCATATTGGGACGCCCTCCTCAAGCGA |  |
| PavFRS6_‘Garnet’ |  | ~~~~~~~~~~CCCGTAGGCAAGATTACTGCACATGTGA~TCCTATACATGGTTGTTTAGAGCTTGGCTTACATGCATATTGGGACGCCCTCCTCAAGCGA |  |
|  |  |  |  |
|  |  | ....|....|....|....|....|....|....|....|....|....|....|....|....|....|....|....|....|....|....|....| |  |
| ppa002486m |  | TCATTACTAGTCAATGCAGAACATTGCAAACTGCCATTTCTGATGTTTTCCCGAGAGCTTCTCATTGTCTTTGCTTATCACATATAATGCAAAAATTTCC |  |
| PRUAV011091 |  | TCATTACTAGTCAATGCAGAACATTGCAAACTGCCATTTCTGATGTTTTCCCTAGAGCTTCTCATTGTCTTTGCTTATCACATATAATGCAAAAGATTCC |  |
| PavFRS6_‘Regina’ |  | TCATTACTAGTCAATGCAGAACATTGCAAACTGCCATTTCTGATGTTTTCCCTAGAGCTTCTCATTGTCTTTGCTTATCACATATAATGCAAAAGATTCC |  |
| PavFRS6_‘Lapins’ |  | TCATTACTAGTCAATGCAGAACATTGCAAACTGCCATTTCTGATGTTTTCCCTAGAGCTTCTCATTGTCTTTGCTTATCACATATAATGCACAAGATTCC |  |
| PavFRS6_‘Garnet’ |  | TCATTACTAGTCAATGCAGAACATTGCAAACTGCCATTTCTGATGTTTTCCCTAGAGCTTCTCATTGTCTTTGCTTATCACATATAATGCACAAGATTCC |  |
|  |  |  |  |
|  |  | ....|....|....|....|....|....|....|....|....|....|....|....|....|....|....|....|....|....|....|....| |  |
| ppa002486m |  | AG~AAAATTTGGGAGG~ATTGTTCGAGTATGAAGCAATTAAAGAATCTTTTAGTAGAGCAGTTTACTACTCTCTGAGGGTGGAGGAATTTGAAGCAGCTT |  |
| PRUAV011091 |  | AGTAAAATTTGGGAGGTATTGTTTGAGTATGAAGCAATTAAAGAATCTTTTAGTAGAGCAGTTTACTACTCTCTGAGGGTGGAGGAGTTTGAAGCAGCTT |  |
| PavFRS6_‘Regina’ |  | AG~AAAATTTGGGAGG~ATTGTTTGAGTATGAAGCAATTAAAGAATCTTTTAGTAGAGCAGTTTACTACTCTCTGAGGGTGGAGGAGTTTGAAGCAGCTT |  |
| PavFRS6_‘Lapins’ |  | AG~AAAATTTGGGAGG~ATTGTTCGAGTATGAAGCAATTAAAGAATCTTTTAGTAGAGCAGTTTACTACTCTCTGRGGGTGGAGGAGTTTGAAGCAGCTT |  |
| PavFRS6_‘Garnet’ |  | AG~AAAATTTGGGAGG~ATTGTTTGAGTATGAAGCAATTAAAGAATCTTTTAGTAGAGCAGTTTACTACTCTCTGRGGGTGGAGGAGTTTGAAGCAGCTT |  |
|  |  |  |  |
|  |  | ....|....|....|....|....|....|....|....|....|....|....|....|....|....|....|....|....|....|....|....| |  |
| ppa002486m |  | GGGAGGATATGGTCCAGCGCCATGGAATTAGAGATCATAAATGGCTTCAAGCATTATTCGACGATCGGAAGCGGTGGGTTCCAGTATATTTGAAGGATAT |  |
| PRUAV011091 |  | GGGAGGATATGGTCCAGCGCCATGGAATTAGAGATCATAAATGGCTTCAAGCATTATTTGAAGATCGGAAGCGGTGGGTTCCAGTATATTTGAAGGATAT |  |
| PavFRS6_‘Regina’ |  | GGGAGGATATGGTCCAGCGCCATGGAATTAGAGATCATAAATGGCTTCAAGCATTATTTGAAGATCGGAAGCGGTGGGTTCCAGTATATTTGAAGGATAT |  |
| PavFRS6_‘Lapins’ |  | GGGAGGATATGGTCCAGCGCCATGGAATTAGAGATCATAAATGGCTTCAAGCATTATTTGAAGATCGGAAGCGGTGGGTTCCAGTATATTTGAAGGATAT |  |
| PavFRS6_‘Garnet’ |  | GGGAGGATATGGTCCAGCGCCATGGAATTAGAGATCATAAATGGCTTCAAGCATTATTTGAAGATCGGAAGCGGTGGGTTCCAGTATATTTGAAGGATAT |  |
|  |  |  |  |
|  |  | ....|....|....|....|....|....|....|....|....|....|....|....|....|....|....|....|....|....|....|....| |  |
| ppa002486m |  | ATTTTTGGCAGGGATGTCCCCTGTGCAACCAAGTGAGGTTGTCTCTTCATACTTCAAAGAATTTCTTCATAAAGATACTCCTTTAAAGGAATTTTTAGAT |  |
| PRUAV011091 |  | ATTTTTGGCAGGGATGTCCCCTGTGCAACCAAGTGAGGTTGTCTCTTCGTACTTCAAAGAATTTCTTCATAAAGATACTCCTTTAAAGGAATTTTTAGAT |  |
| PavFRS6_‘Regina’ |  | ATTTTTGGCAGGGATGTCCCCTGTGCAACCAAGTGAGGTTGTCTCTTCGTACTTCAAAGAATTTCTTCATAAAGATACTCCTTTAAAGGAATTTTTAGAT |  |
| PavFRS6_‘Lapins’ |  | ATTTTTGGCAGGGATGTCCCCTGTGCAACCAAGTGAGGTTGTCTCTTCGTACTTCAAAGAATTTCTTCATAAAGATACTCCTTTAAAGGAATTTTTAGAT |  |
| PavFRS6_‘Garnet’ |  | ATTTTTGGCAGGGATGTCCCCTGTGCAACCAAGTGAGGTTGTCTCTTCGTACTTCAAAGAATTTCTTCATAAAGATACTCCTTTAAAGGAATTTTTAGAT |  |
|  |  |  |  |
|  |  | ....|....|....|....|....|....|....|....|....|....|....|....|....|....|....|....|....|....|....|....| |  |
| ppa002486m |  | AAGTATGATCAAGCTCTGCAAACGCATCATCGGCTGGAAGCCTTGGCAGATTTGGATTCAAGAAATTCGAGTTATATGTTGAAGTCAGGATGCTATTTTG |  |
| PRUAV011091 |  | AAGTATGATCAAGCTCTGCAAACGCATCATCGGCTGGAAGCCTTGGCAGATTTGGATTCAAGAAATTCGAGTTATATGTTGAAGTCAAGATGCTATTTTG |  |
| PavFRS6_‘Regina’ |  | AAGTATGATCAAGCTCTGCAAACGCATCATCGGCTGGAAGCCTTGGCAGATTTGGATTCAAGAAATTCGAGTTATATGTTGAAGTCAAGATGCTATTTTG |  |
| PavFRS6_‘Lapins’ |  | AAGTATGATCAAGCTCTGCAAACGCATCATCGGCTGGAAGCCTTGGCAGATTTGGATTCAAGAAATTCGAGTTATATGTTGAAGTCAAGATGCTATTTTG |  |
| PavFRS6_‘Garnet’ |  | AAGTATGATCAAGCTCTGCAAACGCATCATCGGCTGGAAGCCTTGGCAGATTTGGATTCAAGAAATTCGAGTTATATGTTGAAGTCAAGATGCTATTTTG |  |
|  |  |  |  |
|  |  | ....|....|....|....|....|....|....|....|....|....|....|....|....|....|....|....|....|....|....|....| |  |
| ppa002486m |  | AGTTGCAACTCTCCAAAGTGTACACTAATGACATTCTTAGGAAGTTTGAATCGGAGGTAGAAGGAATGTACTCTTGTTTCAGCACTAGTCAGTTAAATCC |  |
| PRUAV011091 |  | AGTTGCAACTCGCCAAAGTGTACACTAATGACATTCTTAGGAAGTTTGAATCGGAGGTAGAAGGAATGTACTCTTGTTTCAGCACTAGTCAGTTAAATCC |  |
| PavFRS6_‘Regina’ |  | AGTTGCAACTCGCCAAAGTGTACACTAATGACATTCTTAGGAAGTTTGAATCGGAGGTAGAAGGAATGTACTCTTGTTTCAGCACTAGTCAGTTAAATCC |  |
| PavFRS6_‘Lapins’ |  | AGTTGCAACTCGCCAAAGTGTACACTAATGACATTCTTAGGAAGTTTGAATCGGAGGTAGAAGGAATGTACTCTTGTTTCAGCACTAGTCAGTTAAATCC |  |
| PavFRS6_‘Garnet’ |  | AGTTGCAACTCGCCAAAGTGTACACTAATGACATTCTTAGGAAGTTTGAATCGGAGGTAGAAGGAATGTACTCTTGTTTCAGCACTAGTCAGTTAAATCC |  |
|  |  |  |  |
|  |  | ....|....|....|....|....|....|....|....|....|....|....|....|....|....|....|....|....|....|....|....| |  |
| ppa002486m |  | TGATGGGCCAGTCATAA~CACACATAGTGAAGGAACAAACTGAAGTTGATGGAAATAGGAGAGAGGTGAGAGACTATGAGGTTTT~GTATAATCCATCTG |  |
| PRUAV011091 |  | TGATGGGCCAGTCATAA~CATACATAGTGAAGGAACAAACTGAAGTTGATGGAAATAGGAAAGAGGTGAGAGACTATGAGGTTTT~GTATAATCCATCTG |  |
| PavFRS6_‘Regina’ |  | TGATGGGCCAGTCATAAACATACATAGTGTAGGAACAAACTGAAGTTGATGGAATAGGAAAAGAGGTGAGAGACTATGAGGTTTTTGTATTATTCCATCT |  |
| PavFRS6_‘Lapins’ |  | TGATGGGCCAGTCATAA~CATACATAGTGAAGGAACAAACTGAAGTTGATGGAAATAGGAAAGAGGTGAGAGACTATGAGGTTTT~GTATAATCCATCTG |  |
| PavFRS6_‘Garnet’ |  | TGATGGGCCAGTCATAA~CATACATAGTGAAGGAACAAACTGAAGTTGATGGAAATAGGAAAGAGGTGAGAGACTATGAGGTTTT~GTATAAATCCATCT |  |
|  |  |  |  |
|  |  | ....|....|....|....|....|....|....|....|....|....|....|....|....|....|....|....|....|....|....|....| |  |
| ppa002486m |  | AGATGGAGGTCCTTTGCATCTGTGGCATGTTCAACTTGAGGGGATATTTGTGCAGGCACGCACTATCAGTCCTTAACCAAAATGGCGTGGAGGAGATCCC |  |
| PRUAV011091 |  | AGATGGAGTCCTTTGCATCTGTGGCATGTTCAACTTGAGGGGATATTTGTGCAGGCACACACTTTTCAGTCCTTAACCAAAATGGCGTGGAGGAGATCCC |  |
| PavFRS6_‘Regina’ |  | GAGATGGAGGTCCTTTGCATCTGTGGGCATGTTCAACCTTGAGGGGGATATTTGTGCAGGCACACACTTTCATTCTTTAACCATATTGCCTTGGAAGAGA |  |
| PavFRS6_‘Lapins’ |  | AGATGGAGGTCCTTTGCATCTGTGGCATGTTCAACTTGAGGGGATATTTGTGCAGGCACACACTTTCAGTCCTTAACCAAAATGGCGTGGAGGAGATCCC |  |
| PavFRS6_‘Garnet’ |  | GAGATGGAGGTCCTTTGCATCTGTGGCATGTTCAACTTGAGGGGATATTTGTGCAGGCACACACTTTCAGTCCTTTACCCAAAATGGCGTGGAGGAGATC |  |
|  |  |  |  |
|  |  | ....|....|....|....|....|....|....|....|....|....|....|....|....|....|....|....|....|....|....|....| |  |
| ppa002486m |  | GGCTCAATACGTCCTTTCGCGATGGAGAAAGGATATTGAACGCAATTACATTTTTGATCATAGCTGCAGTGGCATTGACATTAATAACCCTGTTCACAGG |  |
| PRUAV011091 |  | GGCTCAATACGTCCTTTCGCGATGGAGAAAGGATATTAAACGCAATTACATTTTTGATCATAGCTGCAGTGGCATTGACATTAATAACCCAGTTCACAGG |  |
| PavFRS6_‘Regina’ |  | TCCCGGCTCAATACTTCCTTTTCGCGATGGAAGAAAGGATATTAAACGCAATTCATTTTTTGATCATG |  |
| PavFRS6_‘Lapins’ |  | GGCTCAATACGTCCTTTCGCGATGGAGAAAGGATATTAAACGCAATTACATTTTTGATCATAGCTGCAGTGGCATTGACATTAATAACCCAGTTCACAGG |  |
| PavFRS6_‘Garnet’ |  | CCGGGCTCAATACGTCCTTTCGCGATGGAGAAAGGATATTAAACGCAATTACATTTTTGATCATAGCTGCAGTGGCATTGACATTAATAACCCAGTTCAC |  |
|  |  |  |  |
|  |  | ....|....|....|....|....|....|....|....|....|....|....|....|....|....|....|....|....|....|....|....| |  |
| ppa002486m |  | TATGACCATTTGTATAAATGCATTGTGCAAGTTGTGGAAGAAGGGAGGAAATCACAAGACCGATACAAGGTTGCATTTGGGGCTTTGGATGAGATATTGA |  |
| PRUAV011091 |  | TATGACCATTTGTATAAATGCATGGTGCAAGTTGTGGAAGAAGGGAGGAAATCACAAGACCGATACAAGGTTGCATTCGGGGCTTTGGATGAGATCTTGA |  |
| PavFRS6_‘Regina’ |  |  |  |
| PavFRS6_‘Lapins’ |  | TATGACCATTTGTATAAATGCATGGTGCAAGTGTGGAAAAACGGGAAA |  |
| PavFRS6_‘Garnet’ |  | AGGTATGACCATTTTGTATAAATGCATGGTGCAAGTTGTGAGAAGAAGGGAA |  |

***FRS8***

|  |  | ....|....|....|....|....|....|....|....|....|....|....|....|....|....|....|....|....|....|....|....| |  |
| --- | --- | --- | --- |
| ppa001906m | 1401 | ATGGACAAAGCGTAACAGCAAGGAGAAGCGTGGTGCAGTGCTCTGTTGCAATTGTGAGGGTTTTAAAACAATTAAAGATGCAAACAGCCGCAAGAAAGAA |  |
| PRUAV037910 |  | ~~~~~~~~~~~~~~~~~~~~~~~~~~~~~~~~~~~~~~~~~~~~~~~~~~~~~~~~~~~~~~~~~~~~~~~~~~~~~~~~CAAACAGCCGCAAGAAAGAA |  |
| PavFRS8_‘Regina’ |  | ~~~~~~~~~~~~~~~~~~~~~~~~~~~~~~~~~~~~~~~~~~~~~~~~~~~~~~~~~~~~~~~~~~~~~~~~~~~~~~~~~~~~~~~~~~~~~~~~~~~~ |  |
| PavFRS8_‘Lapins’ |  | ~~~~~~~~~~~~~~~~~~~~~~~~~~~~~~~~~~~~~~~~~~~~~~~~~~~~~~~~~~~~~~~~~~~~~~~~~~~~~~~~~~~~~~~~~~~~~~~~~~~~ |  |
| PavFRS8_‘Garnet’ |  | ~~~~~~~~~~~~~~~~~~~~~~~~~~~~~~~~~~~~~~~~~~~~~~~~~~~~~~~~~~~~~~~~~~~~~~~~~~~~~~~~~~~~~~~~~~~~~~~~~~~~ |  |
|  |  |  |  |
|  |  | ....|....|....|....|....|....|....|....|....|....|....|....|....|....|....|....|....|....|....|....| |  |
| ppa001906m |  | ACAAGAACAGGCTGTCTAGCAATGATAAGGTTGAGATTAGTAGAATCTAACAGATGGAGAGTGGACGAAGTCAAGCTTGAACACAACCATTTATTCGATC |  |
| PRUAV037910 |  | ACAAGAACAGGCTGTCTAGCAATGATAAGGTTGAGATTAGTAGAATCTAACAGATGGAGAGTGGATGAAGTCAAGCTTGAACACAACCATTTATTCGATC |  |
| PavFRS8_‘Regina’ |  | ~~~~~~GCCCGATCCGCATGCATATGTATACGCAGATTAGTAGAATCTAACAGATGGAGAGTGGATGAAGTCAAGCTTGAACACAACCATTTATTCGATC |  |
| PavFRS8_‘Lapins’ |  | ~~~~~~~~~~~~~~CGGACGCATGCATAGGTTGAGATTAGTAGAATCTAACAGATGGAGAGTGGATGAAGTCAAGCTTGAACACAACCATTTATTYGATC |  |
| PavFRS8_‘Garnet’ |  | ~~~~~~~~~~~~~~CGGACGCATGCATAGGTTGAGATTAGTAGAATCTAACAGATGGAGAGTGGATGAAGTCAAGCTTGAACACAACCATTTATTYGATC |  |
|  |  |  |  |
|  |  | ....|....|....|....|....|....|....|....|....|....|....|....|....|....|....|....|....|....|....|....| |  |
| ppa001906m |  | CTGAAAGAGCTCAGAACTCCAAGTCGCACAAGAGGATGGACTCAGGAGCTAAAAGAAAGATGGAGCCAACTGTTGATGTTGAAGTACGCACAATCAAGTT |  |
| PRUAV037910 |  | CTGAAAGAGCTCAGAACTCCAAGTCGCACAAGAGGATGGACTCAGGAGCTAAAAGAA~GATGGAGCCAACTGTTGATGTTGAAGTACGCACAATCAAGTT |  |
| PavFRS8_‘Regina’ |  | CTGAAAGAGCTCAGAACTCCAAGTCGCACAAGAGGATGGACTCAGGAGCTAAAAGAAAGATGGAGCCAACTGTTGATGTTGAAGTACGCACAATCAAGTT |  |
| PavFRS8_‘Lapins’ |  | CTGAAAGAGCTCAGAACTCCAAGTCGCACAAGAGGATGGACTCAGGAGCTAAAAGAAAGATGGAGCCAACTGTTGATGTTGAAGTACGCACAATCAAGTT |  |
| PavFRS8_‘Garnet’ |  | CTGAAAGAGCTCAGAACTCCAAGTCGCACAAGAGGATGGACTCAGGAGCTAAAAGAAAGATGGAGCCAACTGTTGATGTTGAAGTACGCACAATCAAGTT |  |
|  |  |  |  |
|  |  | ....|....|....|....|....|....|....|....|....|....|....|....|....|....|....|....|....|....|....|....| |  |
| ppa001906m |  | ATATCGGACACCTGTCGTAGATGCAGTTGGTTATGGAAGCTCAAACTCAAATGAGGGAGAAACTAACAACCATGTTGATCGGTCCAAGCGATTGAAACTC |  |
| PRUAV037910 |  | ATATCGGACACCTGTTGTAGATGCAGTTGGTTATGGAAGCTCAAACTCAAACGAGGGAGAAACTAACAACCATGTTGATCGTTCCAAGCGATTGAAACTC |  |
| PavFRS8_‘Regina’ |  | ATATCGGACACCTGTTGTAGATGCAGTTGGTTATGGAAGCTCAAACTCAAACGAGGGAGAAACTAACAACCATGTTGATCGTTCCAAGCGATTGAAACTC |  |
| PavFRS8_‘Lapins’ |  | ATATCGGACACCTGTTGTAGATGCAGTTGGTTATGGAAGCTCAAACTCAAACGAGGGAGAAACTAACAACCATGTTGATCGGTCCAAGCGATTGAAACTC |  |
| PavFRS8_‘Garnet’ |  | ATATCGGACACCTGTTGTAGATGCAGTTGGTTATGGAAGCTCAAACTCAAACGAGGGAGAAACTAACAACCATGTTGATCGTTCCAAGCGATTGAAACTC |  |
|  |  |  |  |
|  |  | ....|....|....|....|....|....|....|....|....|....|....|....|....|....|....|....|....|....|....|....| |  |
| ppa001906m |  | AAAAATGGAGATGCAAGAGCAATTTATAAGTATTTTTGCCGGGTTCAGCTAACCGATCCTAATTTTTTCTATGTGATCGATCTCAATGATGAAGGGTATT |  |
| PRUAV037910 |  | AAAAATGGAGATGCAAGAGCAATTTATAAGTATTTT~GCCGGGTTCAGCTAACCGATCCTAATTTTT~C~ATGTGATCGATCTCAATGATGAAGGT |  |
| PavFRS8_‘Regina’ |  | AAAAATGGAGATGCAAGAGCAATTTATAAGTATTTT~GCCGGGTTCAGCA |  |
| PavFRS8_‘Lapins’ |  | AAAAATGGAGATGCAAGAGCAATTTATAAGTATTTT~GCCGGGTTCAGCA |  |
| PavFRS8_‘Garnet’ |  | AAAAATGGAGATGCAAGAGCAATTTATAAGTATTTT~GCCGGGTTCAGCA |  |

***FT***

|  |  | ....|....|....|....|....|....|....|....|....|....|....|....|....|....|....|....|....|....|....|....| |  |
| --- | --- | --- | --- |
| ppa012320m | 401 | GATCCTGATGCACCCAGCCCAAGTGACCCCAACCTTAAGGAATATTTGCATTGGTGTGTATTATTTGTAGCTTCTCTCTCCCAAGGAATAAAGACATCTC |  |
| PRUAV011973 |  | GATCCTGATGCACCTAGCCCAAGTGACCCCAACCTAAAGGAATATTTGCATTGGC~~~~~~~~~~~~~~~~~~~~~~~~~~~~~~~~~~~~~~~~~~~~~ |  |
| PavFT_‘Regina’ |  | ~~~~~~~~~~~~~~~~~~~~~~~~~~~~~~~ACCTAAAGGAATATTTGCATTGGTGTGTATTATTTGTCGCTTCTCTCTCCCAAGGAATAAAGACATCTC |  |
| PavFT_‘Lapins’ |  | ~~~~~~~~~~~~~~~~~~~~~~~~GACCCCA~CCTAAAGGAATATTTGCATTGGTGTGTATTATTTGTCGCTTCTCTCTCCCAAGGAATAAAGACATCTC |  |
| PavFT_‘Garnet’ |  | ~~~~~~~~~~~~~~~~~~~~~~~~AACCC~TACCTAAAGGAATATTTGCATTGGTGTGTATTATTTGTCGCTTCTCTCTCCCAAGGAATAAAGACATCTC |  |
|  |  |  |  |
|  |  | ....|....|....|....|....|....|....|....|....|....|....|....|....|....|....|....|....|....|....|....| |  |
| ppa012320m |  | TCTCTCTCTCTCTCC~~~~~~CTCCCTCTTTTCCGTAGAAATTTGTCCTATCCCAGCTATATAGATTGACTATATCTTCCCCCTTTGGCTTAAGCAATTT |  |
| PRUAV011973 |  | ~~~~~~~~~~~~~~~~~~~~~~~~~~~~~~~~~~~~~~~~~~~~~~~~~~~~~~~~~~~~~~~~~~~~~~~~~~~~~~~~~~~~~~~~~~~~~~~~~~~~ |  |
| PavFT_‘Regina’ |  | TCTCTCTCTCTCTCTTTCTTTCTCCCTCTTTTCCATAGAAATTTGTCATATTCCCTCCCTATAGATTGACTATATCTTCCCCTTTTGGCTTAAGCAATTT |  |
| PavFT_‘Lapins’ |  | TCTCTCTCTCTCTCTTTCTTTCTCCCTCTTTTCCATAGAAATTTGTCATATTCCCTCCCTATAGATTGACTATATCTTCCCCTTTTGGCTTAAGCAATTT |  |
| PavFT_‘Garnet’ |  | TCTCTCTCTCTCTCTTTCTTTCTCCCTCTTTTCCATAGAAATTTGTCATATTCCCTCCCTATAGATTGACTATATCTTCCCCTTTTGGCTTAAGCAATTT |  |
|  |  |  |  |
|  |  | ....|....|....|....|....|....|....|....|....|....|....|....|....|....|....|....|....|....|....|....| |  |
| ppa012320m |  | GCCATCAAAGTTGAGGTTTCGAGTTCAAATCTTCATCCCTTTTTTGACCAAATATGTATTGGGGAAAGAGTATATATAGACGAAGAAGGATTTGCTTAGG |  |
| PRUAV011973 |  | ~~~~~~~~~~~~~~~~~~~~~~~~~~~~~~~~~~~~~~~~~~~~~~~~~~~~~~~~~~~~~~~~~~~~~~~~~~~~~~~~~~~~~~~~~~~~~~~~~~~~ |  |
| PavFT_‘Regina’ |  | GCCATCAAAGTTGAGGTTTCCAGCTCAAATCTTCATGCCTTTTCCAACCAAATATATATTGGGGAAAGAGTATATATAGACGAAGAAGGATTTGCTTAGG |  |
| PavFT_‘Lapins’ |  | GCCATCAAAGTTGAGGTTTCCAGCTCAAATCTTCATGCCTTTTCCAACCAAATATATATTGGGGAAAGAGTATATATAGACGAAGAAGGATTTGCTTAGG |  |
| PavFT_‘Garnet’ |  | GCCATCAAAGTTGAGGTTTCCAGCTCAAATCTTCATGCCTTTTCCAACCAAATATATATTGGGGAAAGAGTATATATAGACGAAGAAGGATTTGCTTAGG |  |
|  |  |  |  |
|  |  | ....|....|....|....|....|....|....|....|....|....|....|....|....|....|....|....|....|....|....|....| |  |
| ppa012320m |  | AAAAAGAAGAATGTTCATTCATTTATTCCTGGTTGTTTTCGGAATATCAGTTAAATGTTGGTCTTATACTTCTGATTGTGCCAACTTTTCAGGTTGGTTA |  |
| PRUAV011973 |  | ~~~~~~~~~~~~~~~~~~~~~~~~~~~~~~~~~~~~~~~~~~~~~~~~~~~~~~~~~~~~~~~~~~~~~~~~~~~~~~~~~~~~~~~~~~~~~~TGGTTA |  |
| PavFT_‘Regina’ |  | AAAAAGAAGAACGTTCATTCATTTATTCCTGGTTGTTTTCGGAATATCAGTTAAATGTTGGTCTTATACTTCTGATTGTGCCAACTTTTCAGGCTGGTTA |  |
| PavFT_‘Lapins’ |  | AAAAAGAAGAACGTTCATTCATTTATTCCTGGTTGTTTTCGGAATATCAGTTAAATGTTGGTCTTATACTTCTGATTGTGCCAACTTTTCAGGCTGGTTA |  |
| PavFT_‘Garnet’ |  | AAAAAGAAGAACGTTCATTCATTTATTCCTGGTTGTTTTCGGAATATCAGTTAAATGTTGGTCTTATACTTCTGATTGTGCCAACTTTTCAGGCTGGTTA |  |
|  |  |  |  |
|  |  | ....|....|....|....|....|....|....|....|....|....|....|....|....|....|....|....|....|....|....|....| |  |
| ppa012320m |  | CGGATATACCAGCAACAACGGCGGCAAGCTTTGGTGAGTAGTTCCTATTATATTCTAGTTAGGGTAATGGTAGGCTTAATTACCACATTTTTATACCACA |  |
| PRUAV011973 |  | CGGATATTCCAGCAACAACTGCGGCAAGCTTTGG~~~~~~~~~~~~~~~~~~~~~~~~~~~~~~~~~~~~~~~~~~~~~~~~~~~~~~~~~~~~~~~~~~ |  |
| PavFT_‘Regina’ |  | CGGATATTCCAGCAACAACTGCGGCAAGCTTTGGTGAGTAGTTTCTATTATATTATAGTTATGGTAA~~~~~~~~~~~~~~~~~~~~~~~~~~~~~~~~~ |  |
| PavFT_‘Lapins’ |  | CGGATATTCCAGCAACAACTGCGGCAAGCTTTGGTGAGTAGTTTCTATTATATTATAGTTATGGTAA~~~~~~~~~~~~~~~~~~~~~~~~~~~~~~~~~ |  |
| PavFT_‘Garnet’ |  | CGGATATTCCAGCAACAACTGCGGCAAGCTTTGGTGAGTAGTTTCTATTATATTATAGTTATGGTAA~~~~~~~~~~~~~~~~~~~~~~~~~~~~~~~~~ |  |
|  |  |  |  |
|  |  | ....|....|....|....|....|....|....|....|....|....|....|....|....|....|....|....|....|....|....|....| |  |
| ppa012320m |  | CCGTGTACCACCTGTCAAATAGAGATGGAGCCTACCAATACAATGGGGCCCACATCTATTAGAGAGGTTGTACATAATGTGGTATAAAAACGTGATAAAT |  |
| PRUAV011973 |  | ~~~~~~~~~~~~~~~~~~~~~~~~~~~~~~~~~~~~~~~~~~~~~~~~~~~~~~~~~~~~~~~~~~~~~~~~~~~~~~~~~~~~~~~~~~~~~~~~~~~~ |  |
| PavFT_‘Regina’ |  | ~~~~~~~~~~~~~~~~~~~~~~~~~~~~~~~~~~~~~~~~~~~~~~~~~~~~~~~~~~~~~~~~~~~~~~~~~~~~~~~~~~~~~~~~~~~~~~~~~~AT |  |
| PavFT_‘Lapins’ |  | ~~~~~~~~~~~~~~~~~~~~~~~~~~~~~~~~~~~~~~~~~~~~~~~~~~~~~~~~~~~~~~~~~~~~~~~~~~~~~~~~~~~~~~~~~~~~~~~~~~AT |  |
| PavFT_‘Garnet’ |  | ~~~~~~~~~~~~~~~~~~~~~~~~~~~~~~~~~~~~~~~~~~~~~~~~~~~~~~~~~~~~~~~~~~~~~~~~~~~~~~~~~~~~~~~~~~~~~~~~~~AT |  |
|  |  |  |  |
|  |  | ....|....|....|....|....|....|....|....|....|....|....|....|....|....|....|....|....|....|....|....| |  |
| ppa012320m |  | CTAGCATTTTCCTTCTAGTTATTGGGCATTTTTTCTTGGTTTTTTTTGGTGGAGGGGGGAATATTTCACTCATTACCATATAAGTATGAGCTCCTACATT |  |
| PRUAV011973 |  | ~~~~~~~~~~~~~~~~~~~~~~~~~~~~~~~~~~~~~~~~~~~~~~~~~~~~~~~~~~~~~~~~~~~~~~~~~~~~~~~~~~~~~~~~~~~~~~~~~~~~ |  |
| PavFT_‘Regina’ |  | CTAGCATTTTCTTTCTAGTTATTGGGGTTT~~~~~~~~~~~~~~~~~~~~~~~GGG~~~~~~~~~~~~~~~~~~ACCATATAAGTTGGAGCTTCTACATT |  |
| PavFT_‘Lapins’ |  | CTAGCATTTTCTTTCTAGTTATTGGGGTTT~~~~~~~~~~~~~~~~~~~~~~~GGG~~~~~~~~~~~~~~~~~~ACCATATAAGTTGGAGCTTCTACATT |  |
| PavFT_‘Garnet’ |  | CTAGCATTTTCTTTCTAGTTATTGGGGTTT~~~~~~~~~~~~~~~~~~~~~~~GGG~~~~~~~~~~~~~~~~~~ACCATATAAGTTGGAGCTTCTACATT |  |
|  |  |  |  |
|  |  | ....|....|....|....|....|....|....|....|....|....|....|....|....|....|....|....|....|....|....|....| |  |
| ppa012320m |  | CTTACCAATAGTAATGTGACAGGTGTGCATAAAATTTTCAAAGAAATGTTCGATTGGTCT~~~~AGTCTACAAA~GGCATAATTAATTAACATTTGATTA |  |
| PRUAV011973 |  | ~~~~~~~~~~~~~~~~~~~~~~~~~~~~~~~~~~~~~~~~~~~~~~~~~~~~~~~~~~~~~~~~~~~~~~~~~~~~~~~~~~~~~~~~~~~~~~~~~~~~ |  |
| PavFT_‘Regina’ |  | CCTCCCAATAGTAGTGTCACATGTGTGCATAAAATTTTCAAAGAAATGTCCAATTGGGCTCGCTAGTCTACAAAAGGCTTAATTAATTAACATTTGATTA |  |
| PavFT_‘Lapins’ |  | CCTCCCAATAGTAGTGTCACATGTGTGCATAAAATTTTCAAAGAAATGTCCAATTGGGCTCGCTAGTCTACAAAAGGCTTAATTAATTAACATTTGATTA |  |
| PavFT_‘Garnet’ |  | CCTCCCAATAGTAGTGTCACATGTGTGCATAAAATTTTCAAAGAAATGTCCAATTGGGCTCGCTAGTCTACAAAAGGCTTAATTAATTAACATTTGATTA |  |
|  |  |  |  |
|  |  | ....|....|....|....|....|....|....|....|....|....|....|....|....|....|....|....|....|....|....|....| |  |
| ppa012320m |  | TATTTTTGGATGCATGACCAATTTATAATGAAACCCCATATACGTACATTTATTGATGTAGGGCAAGAGATTGTGTGTTATGAAAGCCCACGGCCAACGG |  |
| PRUAV011973 |  | ~~~~~~~~~~~~~~~~~~~~~~~~~~~~~~~~~~~~~~~~~~~~~~~~~~~~~~~~~~~~~~GCAAGAGATTGTGTGTTATGAAAGCCCACGGCCAACGG |  |
| PavFT_‘Regina’ |  | TATTTTTGGATGCATGACCAATTTATAATGAAACCCCATATACGTACATTTATTGATGTAGGGCAAGAGATTGTGTGTTATGAAAGCCCACGGCCAACGG |  |
| PavFT_‘Lapins’ |  | TATTTTTGGATGCATGACCAATTTATAATGAAACCCCATATACGTACATTTATTGATGTAGGGCAAGAGATTGTGTGTTATGAAAGCCCACGGCCAACGG |  |
| PavFT_‘Garnet’ |  | TATTTTTGGATGCATGACCAATTTATAATGAAACCCCATATACGTACATTTATTGATGTAGGGCAAGAGATTGTGTGTTATGAAAGCCCACGGCCAACGG |  |
|  |  |  |  |
|  |  | ....|....|....|....|....|....|....|....|....|....|....|....|....|....|....|....|....|....|....|....| |  |
| ppa012320m |  | TGGGGATTCATCGCTTTGTTTTGGTGTTATTTCGGCAATTGGGTAGGCAAACAGTGTATGCTCCGGGGTGGCGCCAGAACTTTAATACTAGAGACTTTGC |  |
| PRUAV011973 |  | TGGGGATTCATCGCTTTGTTTTGGTGTTGTTTCGGCAATTGGGTAGGCAAACAGTGTATGCTCCGGGATGGCGCCAGAACTTTAATACAAGAGACTTTGC |  |
| PavFT_‘Regina’ |  | TGGGGATTCATCGCTTTGTTTTGGTGTTGTTTCGGCAATTGGGTAGGCAAACAGTGTATGCTCCGGGATGGCGCCAGAACTT |  |
| PavFT_‘Lapins’ |  | TGGGGATTCATCGCTTTGTTTTGGTGTTGTTTCGGCAATTGGGTAGGCAAACAGTGTATGCTCCGGGATGGCGCCAGAACTT |  |
| PavFT_‘Garnet’ |  | TGGGGATTCATCGCTTTGTTTTGGTGTTGTTTCGGCAATTGGGTAGGCAAACAGTGTATGCTCCGGGATGGCGCCAGAACTT |  |
|  |  |  |  |
|  |  | ....|....|....|....|....|....|....|....|....|....|....|....|....|....|....|....|....|....|....|....| |  |
| ppa012320m |  | GGAGCTTTACAATCTTGGATTACCGGTATCTGCCGTCTATTTTAACTGCCAAAGGGAGAGCGGCTCTGGAGGGAGGAGAAGATAATTAAGTTCGATATTA |  |
| PRUAV011973 |  | GGAGCTTTACAATCTTGGATTACCGGTAGCTGCCGTCTATTTTAATTGTCAAAGAGAAAGCGGCTCCGGAGGAAGGAGAAGATAATTAAGTTTGATATTA |  |
| PavFT_‘Regina’ |  |  |  |
| PavFT_‘Lapins’ |  |  |  |
| PavFT_‘Garnet’ |  |  |  |

***FYPP3***

|  |  | ....|....|....|....|....|....|....|....|....|....|....|....|....|....|....|....|....|....|....|....| |  |
| --- | --- | --- | --- |
| ppa009178m | 3621 | GAGGAAGTATGGAAATGCTAATGCATGGCGGTACTGTACAGATGTTTTCGACTATCTGACGCTTTCAGCAATTATAGATGGAACTGTAAACCTCCTCTCT |  |
| PRUAV002173 |  | GAGGAAGTATGGAAATGCTAATGCATGGCGGTACTGTACAGATGTTTTCGACTATCTGACGCTATCAGCAATTATAGATGGAACT~~~~~~~~~~~~~~~ |  |
| PavFYPP3_‘Regina’ |  | ~~~~~~~~~~~~~~~~~~~~~~~~~~~~~~~~~~~~~gcag~~~~ctt~GA~~TTCTGACGCTATCAGCAATTATAGATGGAACTGTAAACCTCCTCTCT |  |
| PavFYPP3_‘Lapins’ |  | ~~~~~~~~~~~~~~~~~~~~~~~~~~~~~~~~~~~~~ccag~~~~CTT~GAC~ATCTGACGCTATCAGCAATTATAGATGGAACTGTAAACCTCCTCTCT |  |
| PavFYPP3_‘Garnet’ |  | ~~~~~~~~~~~~~~~~~~~~~~~~~~~~~~~~~~~~~ggag~~~~cttTGAT~TTCTGACGCTATCAGCAATTATAGATGGAACTGTAAACCTCCTCTCT |  |
|  |  |  |  |
|  |  | ....|....|....|....|....|....|....|....|....|....|....|....|....|....|....|....|....|....|....|....| |  |
| ppa009178m |  | CCCTCTCTCTATGTATATGTCTGTGTGTTTGTGTTAGTATGTATCTCTTATCTGTGGTGTACTTTTTGATAGTAAATATGTTTATTTGGTTTGTCATCCC |  |
| PRUAV002173 |  | ~~~~~~~~~~~~~~~~~~~~~~~~~~~~~~~~~~~~~~~~~~~~~~~~~~~~~~~~~~~~~~~~~~~~~~~~~~~~~~~~~~~~~~~~~~~~~~~~~~~~ |  |
| PavFYPP3_‘Regina’ |  | CCCTCTCTCTATGTATATGTCTGTGTGTTTGTGTTAGTATGTATCTCTTATCTGTGGTGTACATTT~GATAGTGAATATGTTTATTTGGTTTGTCATCCC |  |
| PavFYPP3_‘Lapins’ |  | CCCTCTCTCTATGTATATGTCTGTGTGTTTGTGTTAGTATGTATCTCTTATCTGTGGTGTACATTT~GATAGTGAATATGTTTATTTGGTTTGTCATCCC |  |
| PavFYPP3_‘Garnet’ |  | CCCTCTCTCTATGTATATGTCTGTGTGTTTGTGTTAGTATGTATCTCTTATCTGTGGTGTACATTT~GATAGTGAATATGTTTATTTGGTTTGTCATCCC |  |
|  |  |  |  |
|  |  | ....|....|....|....|....|....|....|....|....|....|....|....|....|....|....|....|....|....|....|....| |  |
| ppa009178m |  | AGGTGCTATGTGTCCATGGTGGACTTTCTCCTGACATCCGAACAATTGATCAAGTAACCCCTTCTTGATATGCATTTTAACTTGTTGGTATATGTATGTA |  |
| PRUAV002173 |  | ~~GTGCTATGTGTCCATGGTGGACTTTCTCCTGACATCCGAACAATTGATCAA~~~~~~~~~~~~~~~~~~~~~~~~~~~~~~~~~~~~~~~~~~~~~~~ |  |
| PavFYPP3_‘Regina’ |  | AGGTGCTATGTGTCCATGGTGGACTTTCTCCTGACATCCGAACAATTGATCAAGTAACCCCTTCTTGATATGCATTT~AACTTGTTGATGTATGTTTGTA |  |
| PavFYPP3_‘Lapins’ |  | AGGTGCTATGTGTCCATGGTGGACTTTCTCCTGACATCCGAACAATTGATCAAGTAACCCCTTCTTGATATGCATTT~AACTTGTTGATGTATGTTTGTA |  |
| PavFYPP3_‘Garnet’ |  | AGGTGCTATGTGTCCATGGTGGACTTTCTCCTGACATCCGAACAATTGATCAAGTAACCCCTTCTTGATATGCATTT~AACTTGTTGATGTATGTTTGTA |  |
|  |  |  |  |
|  |  | ....|....|....|....|....|....|....|....|....|....|....|....|....|....|....|....|....|....|....|....| |  |
| ppa009178m |  | TTTATGTTTTTCATGCATGTTGCTACAG~TGCAATTTTGCATTAGACACTTTGCATGGAGGCTTCTTGCATGGATATGATTAACAGTTTCTTTGATTCTG |  |
| PRUAV002173 |  | ~~~~~~~~~~~~~~~~~~~~~~~~~~~~~~~~~~~~~~~~~~~~~~~~~~~~~~~~~~~~~~~~~~~~~~~~~~~~~~~~~~~~~~~~~~~~~~~~~~~~ |  |
| PavFYPP3_‘Regina’ |  | TTTATGTTTTTCATGCATGTTGCTACAGCTGCAATTTTGCATTAGCCACTTTGCATGGAGGCTTCTTGCATGGATATGATTAACAGTTTCTTAGATTCTG |  |
| PavFYPP3_‘Lapins’ |  | TTTATGTTTTTCATGCATGTTGCTACAGCTGCAATTTTGCATTAGCCACTTTGCATGGAGGCTTCTTGCATGGATATGATTAACAGTTTCTTAGATTCTG |  |
| PavFYPP3_‘Garnet’ |  | TTTATGTTTTTCATGCATGTTGCTACAGCTGCAATTTTGCATTAGCCACTTTGCATGGAGGCTTCTTGCATGGATATGATTAACAGTTTCTTAGATTCTG |  |
|  |  |  |  |
|  |  | ....|....|....|....|....|....|....|....|....|....|....|....|....|....|....|....|....|....|....|....| |  |
| ppa009178m |  | TACTTTTGCGTGTTAATCAATTGAAAGAAGCACTTATCCCGTGAATGTTAGTTTAGCTTTTCAAAGAAGCACTTATCCTATGGCCTTGTTCTCAAACAAG |  |
| PRUAV002173 |  | ~~~~~~~~~~~~~~~~~~~~~~~~~~~~~~~~~~~~~~~~~~~~~~~~~~~~~~~~~~~~~~~~~~~~~~~~~~~~~~~~~~~~~~~~~~~~~~~~~~~~ |  |
| PavFYPP3_‘Regina’ |  | TAATTTTGYGTGTTAATCAATTGAAAGAAGCACTTATCCCATGAATGTTAGTTTAGCTTTTCAAAGAAGGACTTATCCTATGGTCTCGTTCTCAAACAAG |  |
| PavFYPP3_‘Lapins’ |  | TAATTTTGYGTGTTAATCAATTGAAAGAAGCACTTATCCCATGAATGTTAGTTTAGCTTTTCAAAGAAGGACTTATCCTATGGTCTCGTTCTCAAACAAG |  |
| PavFYPP3_‘Garnet’ |  | TAATTTTGYGTGTTAATCAATTGAAAGAAGCACTTATCCCATGAATGTTAGTTTAGCTTTTCAAAGAAGGACTTATCCTATGGTCTCGTTCTCAAACAAG |  |
|  |  |  |  |
|  |  | ....|....|....|....|....|....|....|....|....|....|....|....|....|....|....|....|....|....|....|....| |  |
| ppa009178m |  | TTTAGCTTTTATTTTTTT~~AACTCAGTTCCCTCTATTTGAAGCACCTATACCCATGATATGAAAATGCTACCATTTGTATGA~TTATCTTATTTTTTTG |  |
| PRUAV002173 |  | ~~~~~~~~~~~~~~~~~~~~~~~~~~~~~~~~~~~~~~~~~~~~~~~~~~~~~~~~~~~~~~~~~~~~~~~~~~~~~~~~~~~~~~~~~~~~~~~~~~~~ |  |
| PavFYPP3_‘Regina’ |  | TTTAGCTTTTATTTTTTTTTAACTCAGTTCCCTCAATTTCAAGCACCTATACCCATGATATGATAATGCTACCATTTGCATGAATTATCTTTTTTTTTTG |  |
| PavFYPP3_‘Lapins’ |  | TTTAGCTTTTATTTTTTTTTAACTCAGTTCCCTCAATTTCAAGCACCTATACCCATGATATGATAATGCTACCATTTGCATGAATTATCTTTTTTTTTTG |  |
| PavFYPP3_‘Garnet’ |  | TTTAGCTTTTATTTTTTTTTAACTCAGTTCCCTCAATTTCAAGCACCTATACCCATGATATGATAATGCTACCATTTGCATGAATTATCTTTTTTTTTTG |  |
|  |  |  |  |
|  |  | ....|....|....|....|....|....|....|....|....|....|....|....|....|....|....|....|....|....|....|....| |  |
| ppa009178m |  | GGTTACAATTTGCCATT~~~~~~~~~~~~~TCCAGCATCATCATCACTGCACATGCTTTTTCC~~AAACAGATAAGGGTTATTGAGAGAAACTGTGAAAT |  |
| PRUAV002173 |  | ~~~~~~~~~~~~~~~~~~~~~~~~~~~~~~~~~~~~~~~~~~~~~~~~~~~~~~~~~~~~~~~~~~~~~~~ATAAGGGTTATTGAGAGAAACTGTGAAAT |  |
| PavFYPP3_‘Regina’ |  | GGTTACAATCTGCCATTATAATTTGCCATTTCCAGCGTCATCATCACTGCACATGCTTTTTCCTGTAACAAATAAGGGTTATTGAAAGAAACTGTGAAAT |  |
| PavFYPP3_‘Lapins’ |  | GGTTACAATCTGCCATTATAATTTGCCATTTCCAGCGTCATCATCACTGCACATGCTTTTTCCTGTAACAAATAAGGGTTATTGAAAGAAACTGTGAAAT |  |
| PavFYPP3_‘Garnet’ |  | GGTTACAATCTGCCATTATAATTTGCCATTTCCAGCGTCATCATCACTGCACATGCTTTTTCCTGTAACAAATAAGGGTTATTGAAAGAAACTGTGAAAT |  |
|  |  |  |  |
|  |  | ....|....|....|....|....|....|....|....|....|....|....|....|....|....|....|....|....|....|....|....| |  |
| ppa009178m |  | TCCACACGAGGGGCCATTTTGTGATCTCATGTGGAGTGATCCTGAAGATATTGAAACATGGGCAGTAAGTCCACGTGGAGCAGGTTGGCTTTTTGGATCC |  |
| PRUAV002173 |  | TCCACACGAGGGGCCATTTTGTGATCTCATGTGGAGTGATCCTGAAGATATTGAAACATGGGCAGTAAGTCCACGTGGAGCAGGTTGGCTTTTTGGATCC |  |
| PavFYPP3_‘Regina’ |  | TCAACtaaggggaaaa |  |
| PavFYPP3_‘Lapins’ |  | TCCCCcgaaggggaaa |  |
| PavFYPP3_‘Garnet’ |  | TCCCCCaaaggggaaaa |  |

***GA2ox***

|  |  | *....|....|....|....|....|....|....|....|....|....|....|....|....|....|....|....|....|....|....|....|* |  |
| --- | --- | --- | --- |
| ppa008414m | 301 | GGTGATGTTGGTTGGGTGGAGTACCTTCTTCTAACAGCCAACATAGAATCCAATTCCCAGAGATTTTTATCAGTTTTTGGACAGAACCCAGAAGAGTTCT |  |
| PRUAV012448 |  | ggtgatgttggttgggtggagtaccttcttctaacagccaacacagaatccaattcccagagatttttatcagtttttggacagaacccagaagagtttt |  |
| PavGA2ox_‘Regina’ |  | ~~~~~~~~~~~~~~~~~~~~~~~~~~~~~~~~~~~~~~~~~~~~~~~~~~~~~~~~~~~~~~~~~~~~~~~~~~~~~~~~~~~~~~~~~~~~~~~~~~~~ |  |
| PavGA2ox_‘Lapins’ |  | ~~~~~~~~~~~~~~~~~~~~~~~~~~~~~~~~~~~~~~~~~~~~~~~~~~~~~~~~~~~~~~~~~~~~~~~~~~~~~~~~~~~~~~~~~~~~~~~~~~~~ |  |
| PavGA2ox_‘Garnet’ |  | ~~~~~~~~~~~~~~~~~~~~~~~~~~~~~~~~~~~~~~~~~~~~~~~~~~~~~~~~~~~~~~~~~~~~~~~~~~~~~~~~~~~~~~~~~~~~~~~~~~~~ |  |
|  |  |  |  |
|  |  | ....|....|....|....|....|....|....|....|....|....|....|....|....|....|....|....|....|....|....|....| |  |
| ppa008414m |  | GGTATGCTATTCAAGCACAATCAATCAGAAGTTGGGAA~TTTTGATTCTTTTTTCCTTTTCTTTTCATGTCAAGTTCTTGATTAAAAAAATGTCTTCTTT |  |
| PRUAV012448 |  | g~~~~~~~~~~~~~~~~~~~~~~~~~~~~~~~~~~~~~~~~~~~~~~~~~~~~~~~~~~~~~~~~~~~~~~~~~~~~~~~~~~~~~~~~~~~~~~~~~~~ |  |
| PavGA2ox_‘Regina’ |  | ~~~~~~~~~~T~AGGC~CAATC~ATCAGAAGTT~GGAA~TTTTGATTCTTTTTTTCTTTTCTTTTCATGTCAAGTTCTTGATGAAAAAAACGTCTTCTTT |  |
| PavGA2ox_‘Lapins’ |  | ~~~~~~~~~~T~AGGCACAATC~ATCAGAAGTT~GGAAATTTTGATTCTTTTTTTCTTTTCTTTTCATGTCAAGTTCTTGATGAAAAAAACGTCTTCTTT |  |
| PavGA2ox_‘Garnet’ |  | ~~~~~~~~~~TCAGGCACAATC~ATCAGAAGTT~GGAA~TTTTGATTCTTTTTTTCTTTTCTTTTCATGTCAAGTTCTTGATGAAAAAAACGTCTTCTTT |  |
|  |  |  |  |
|  |  | ....|....|....|....|....|....|....|....|....|....|....|....|....|....|....|....|....|....|....|....| |  |
| ppa008414m |  | TGTCCTTGCAGTTCTGCTTTGAATGATTATATATCAGCTGTGAAGAAAATGGCATGTGAGATTCTTGAACTGATGGCTGAAGGATTAAAGATTCAACCAA |  |
| PRUAV012448 |  | ~~~~~~~~~~~ttctgctttgaatgattatatatcagctgtgaagaaaatgacatgtgagattcttgaactgatggctgaaggattaaagattcaaccaa |  |
| PavGA2ox_‘Regina’ |  | TGTCCTTGCAGTTCTGCTTTGAATGATTATATATCAGCTGTGAAGAAAATGACATGTGAGATTCTTGAACTGATGGCTGAAGGATTAAAGATTCAACCAA |  |
| PavGA2ox_‘Lapins’ |  | TGTCCTTGCAGTTCTGCTTTGAATGATTATATATCAGCTGTGAAGAAAATGACATGTGAGATTCTTGAACTGATGGCTGAAGGATTAAAGATTCAACCAA |  |
| PavGA2ox_‘Garnet’ |  | TGTCCTTGCAGTTCTGCTTTGAATGATTATATATCAGCTGTGAAGAAAATGACATGTGAGATTCTTGAACTGATGGCTGAAGGATTAAAGATTCAACCAA |  |
|  |  |  |  |
|  |  | ....|....|....|....|....|....|....|....|....|....|....|....|....|....|....|....|....|....|....|....| |  |
| ppa008414m |  | GGAATGTGTTCAGCAAGCTTTTGATGGATGAACAGAGTGACTCTTACTTCAGGTTAAATCACTACCCACCATGCCCAGAGCTT~CAAG~ATTTGAGTG~C |  |
| PRUAV012448 |  | ggaatgtgttcagcaagcttttgatggatgaacagagtgactcttgcttcaggttaaatcactacccaccatgtccagagcttacaagtatttgagtgac |  |
| PavGA2ox_‘Regina’ |  | GGAATGTGTTCAGCAAGCTTTTGATGGATGAACAGAGTGACTCTTGCTTCAGGTTAAATCACTACCCACCATGTCCAGAGCTT~CAAG~ATTTGAGTG~C |  |
| PavGA2ox_‘Lapins’ |  | GGAATGTGTTCAGCAAGCTTTTGATGGATGAACAGAGTGACTCTTGCTTCAGGTTAAATCACTACCCACCATGTCCAGAGCTT~CAAG~ATTTGAGTG~C |  |
| PavGA2ox_‘Garnet’ |  | GGAATGTGTTCAGCAAGCTTTTGATGGATGAACAGAGTGACTCTTGCTTCAGGTTAAATCACTACCCACCATGTCCAGAGCTT~CAAG~ATTTGAGTG~C |  |
|  |  |  |  |
|  |  | ....|....|....|....|....|....|....|....|....|....|....|....|....|....|....|....|....|....|....|....| |  |
| ppa008414m |  | CA~GAAATGT~GATTGGATTTGGAGCGCACACAGACCCACAAATC~~~ATCTCTGTGCTGAGATCCAACAACACATCTGGCCTCCAAATTTCATTGAGAG |  |
| PRUAV012448 |  | caagaaatgtagattggg |  |
| PavGA2ox_‘Regina’ |  | CA~GAAATGT~GATTGGATTTGGAGCGCACACAGACCC~ACAA~~ATCATCTCTGTGCTGAGATCCAACAACACATCTGGCCTCCAAATTTCATTGAGAG |  |
| PavGA2ox_‘Lapins’ |  | CA~GAAATGT~GATTGGATTTGGAGCGCACACAGACCC~ACAA~~ATCATCTCTGTGCTGAGATCCAACAACACATCTGGCCTCCAAATTTCATTGAGAG |  |
| PavGA2ox_‘Garnet’ |  | CA~GAAATGT~GATTGGATTTGGAGCGCACACAGACCC~ACAA~~ATCATCTCTGTGCTGAGATCCAACAACACATCTGGCCTCCAAATTTCATTGAGAG |  |
|  |  |  |  |
|  |  | ....|....|....|....|....|....|....|....|....|....|....|....|....|....|....|....|....|....|....|....| |  |
| ppa008414m |  | ATGGGAATTGGATTCCAGTCCCACCTGATCATAACTCCTTCTTCATCAATGTTGGTGACTCTTTGCAGGTACTGCTACTTTTTTTT~ATTCTTTTGGCTG |  |
| PRUAV012448 |  |  |  |
| PavGA2ox_‘Regina’ |  | ATGGAAATTGGATTCCAGTCCCACCTGATCATAACTCCTTCTTCATCAATGTTGGTGACTCTTTGCAGGTGCTGCTACTTTTTTTTGATTCTTTTGGCTG |  |
| PavGA2ox_‘Lapins’ |  | ATGGAAATTGGATTCCAGTCCCACCTGATCATAACTCCTTCTTCATCAATGTTGGTGACTCTTTGCAGGTGCTGCTACTTTTTTTTGATTCTTTTGGCTG |  |
| PavGA2ox_‘Garnet’ |  | ATGGAAATTGGATTCCAGTCCCACCTGATCATAACTCCTTCTTCATCAATGTTGGTGACTCTTTGCAGGTGCTGCTACTTTTTTTTGATTCTTTTGGCTG |  |
|  |  |  |  |
|  |  | ....|....|....|....|....|....|....|....|....|....|....|....|....|....|....|....|....|....|....|....| |  |
| ppa008414m |  | AAAAAAAAGAAACTGTTTAAACAAGTAAAAACAAAGATTCTAATTTGCAAATCAGTTCCCTGTAGAGGAAGATTCCTATTTCCATGTCAGTTCCCTATTA |  |
| PRUAV012448 |  |  |  |
| PavGA2ox_‘Regina’ |  | AAAAAAA~~~~~CTGATTA~~~~~~~~~~~ACTAAGATTCAAATTTGATTCTCATTTGCCTGTCAAGGACCAGTCCAATATCATTGCCATTTCCCTGTTA |  |
| PavGA2ox_‘Lapins’ |  | AAAAAAA~~~~~CTGTTTAAATAAGTAAAAACAAAGATTCTAATTTGCAAATCAGTTTCCTGTAGAGGAAGATTCCTATTTCCATGTCAGTTCCCTATTA |  |
| PavGA2ox_‘Garnet’ |  | AAAAAAA~~~~~CTGTTTAAATAAGTAAAAACAAAGATTCTAATTTGCAAATCAGTTTCCTGTAGAGGAAGATTCCTATTTCCATGTCAGTTCCCTATTA |  |
|  |  |  |  |
|  |  | ....|....|....|....|....|....|....|....|....|....|....|....|....|....|....|....|....|....|....|....| |  |
| ppa008414m |  | CCATGGATAAAATATGAATATA~~~~~~~~~~TTTGACTCAAAGAACATTCAACTGTTTAGTTTAGATAAACACCACATCCTTGTTTTTATCTTTCATTT |  |
| PRUAV012448 |  |  |  |
| PavGA2ox_‘Regina’ |  | CTTCGCATTAAATATGAATAAAATTTAATAATTTTGACTCATATATGATTCAAAAAATTCTCTTAGATTACCACCACATCAAAACCACAACCTATTATTT |  |
| PavGA2ox_‘Lapins’ |  | CCGTGGATAAGATATGAATATATATAAATATATTTGACTCAAAGAACATTCAACAGTTTCGTTTAGATAAACACCACATCCTAGTTTTTATCTTTTATTT |  |
| PavGA2ox_‘Garnet’ |  | CCGTGGATAAGATATGAATATATATAAATATATTTGACTCAAAGAACATTCAACAGTTTCGTTTAGATAAACACCACATCCTAGTTTTTATCTTTTATTT |  |
|  |  |  |  |
|  |  | ....|....|....|....|....|....|....|....|....|....|....|....|....|....|....|....|....|....|....|....| |  |
| ppa008414m |  | ATTTCTATTGGGTTTGTTCCAAGTTCTTCTGTTCTCTCATTATCATTCATATTAATCTAATGGGGGCCTTGGTGGGTTGGATTTGCAGGTTTTGACCAAT |  |
| PRUAV012448 |  |  |  |
| PavGA2ox_‘Regina’ |  | ATCTCTAATGTATTTGTACTGGGTTCGTTCGTTCTCTCTCTGTCCTCCCTATAACACTCATGTTAACCTTGTGGGGGTTGGATTTGGGTGGTATTACCAA |  |
| PavGA2ox_‘Lapins’ |  | ATTTCTATTGGGTTTGTTCTAAGTTCTTCTGTTCTCTCTCTATCATTCATATTAATCTAATGGGGGCCTTGGTGGGTTGGATTTGCAGGTTTTGACCAAT |  |
| PavGA2ox_‘Garnet’ |  | ATTTCTATTGGGTTTGTTCTAAGTTCTTCTGTTCTCTCTCTATCATTCATATTAATCTAATGGGGGCCTTGGTGGGTTGGATTTGCAGGTTTTGACCAAT |  |
|  |  |  |  |
|  |  | ....|....|....|....|....|....|....|....|....|....|....|....|....|....|....|....|....|....|....|....| |  |
| ppa008414m |  | GGGAGGTTCCAAAGTGTGAGGCACAGGGTTTTGGCCAATGGTTCAAAATCAAGAGTTTCAATGATTTATTTTGGGGGGCCACCCTTGAGTGAGAAAATAG |  |
| PRUAV012448 |  |  |  |
| PavGA2ox_‘Regina’ |  | GGTGAGACTCCAGAGAGTTTTCGAAAAAGAGGGGCAAAAAA |  |
| PavGA2ox_‘Lapins’ |  | GGGAGGTTCCAAAG~GTAAGAAGGCAAAAGAA |  |
| PavGA2ox_‘Garnet’ |  | GGGAGGTTCCAAA~TGTAGAGAGGCCACAGAGAA |  |

***GCR1***

|  |  | ....|....|....|....|....|....|....|....|....|....|....|....|....|....|....|....|....|....|....|....| |  |
| --- | --- | --- | --- |
| ppa008880m | 101 | CTCATTCGTCGGGTCGGGCTTCATAGTCCTCTGCTACGTTCTCTTCAAAGAGCTCCGCAAGTTCTCCTTCAAGCTCGTATTCTACCTCGCCCTCTCTGTA |  |
| PRUAV007411 |  | ctcattcgtcgggtcgggcttcatagtcctctgctacgttctcttcaaagagctccgcaagttctccttcaagctcgtcttctacctcgccctctct~~~ |  |
| PavGCR1_‘Regina’ |  | ~~~~~~~~~~~~~~~~~~~~~~~~~~~~~~~~~~~~~~~~~~~~~~~~~~~~~~~~ACGAG~TCT~CTTCA~GCTCGTCTTCTACCTCGCCCTCTCTGTA |  |
| PavGCR1_‘Lapins’ |  | ~~~~~~~~~~~~~~~~~~~~~~~~~~~~~~~~~~~~~~~~~~~~~~~~~~~~~~~AACAGAA~~T~CTTCA~GCTCGTCTTCTACCTCGCCCTCTCTGTA |  |
| PavGCR1_‘Garnet’ |  | ~~~~~~~~~~~~~~~~~~~~~~~~~~~~~~~~~~~~~~~~~~~~~~~~~~~~~~~~ACCGGCTCTCCTTCA~GCTCGTCTTCTACCTCGCCCTCTCTGTA |  |
|  |  |  |  |
|  |  | ....|....|....|....|....|....|....|....|....|....|....|....|....|....|....|....|....|....|....|....| |  |
| ppa008880m |  | TGTCTTCTCTCTCATTCTGTGAATTTTGCTTAAAATGATAAAATTGCGCCTGTTAGATCTAATT~~~~~~~~~~GCTCGATTGCCGGTGACGTGGTGTTT |  |
| PRUAV007411 |  | ~~~~~~~~~~~~~~~~~~~~~~~~~~~~~~~~~~~~~~~~~~~~~~~~~~~~~~~~~~~~~~~~~~~~~~~~~~~~~~~~~~~~~~~~~~~~~~~~~~~~ |  |
| PavGCR1_‘Regina’ |  | TGTCTTCTCTCTCATTCTGTGAATTTTGCTTAAAATGATAAAATTGCGTCTGTTA~~~~~~~TTAGATCTAATTGCTCGATTGCCGGTGACGTGGTGTTT |  |
| PavGCR1_‘Lapins’ |  | TGTCTTCTCTCTCATTCTGTGAATTTTGCTTAAAATGATAAAATTGCGTCTGTTA~~~~~~~TTAGATCTAATTGCTCGATTGCCGGTGACGTGGTGTTT |  |
| PavGCR1_‘Garnet’ |  | TGTCTTCTCTCTCATTCTGTGAATTTTGCTTAAAATGATAAAATTGCGTCTGTTA~~~~~~~TTAGATCTAATTGCTCGATTGCCGGTGACGTGGTGTTT |  |
|  |  |  |  |
|  |  | ....|....|....|....|....|....|....|....|....|....|....|....|....|....|....|....|....|....|....|....| |  |
| ppa008880m |  | TCTATTTCTGGAAACTTGAGCTTCTCTAAATACTTGGAAATTGAACTTAATCTATAGCAAATTGCATTTGATTACTTTAAAATTGTTAGAAAATTGTGCA |  |
| PRUAV007411 |  | ~~~~~~~~~~~~~~~~~~~~~~~~~~~~~~~~~~~~~~~~~~~~~~~~~~~~~~~~~~~~~~~~~~~~~~~~~~~~~~~~~~~~~~~~~~~~~~~~~~~~ |  |
| PavGCR1_‘Regina’ |  | TCTATTTCTGGACACTTGAGCTTCGCTAAATACTCGGAAATTGAACTTAATCTGTAGCAAATTGCATTTGATTACTTTAAAATTGTTAGAAAAATGTGCA |  |
| PavGCR1_‘Lapins’ |  | TCTATTTCTGGACACTTGAGCTTCGCTAAATACTCGGAAATTGAACTTAATCTGTAGCAAATTGCATTTGATTACTTTAAAATTGTTAGAAAAATGTGCA |  |
| PavGCR1_‘Garnet’ |  | TCTATTTCTGGACACTTGAGCTTCGCTAAATACTCGGAAATTGAACTTAATCTGTAGCAAATTGCATTTGATTACTTTAAAATTGTTAGAAAAATGTGCA |  |
|  |  |  |  |
|  |  | ....|....|....|....|....|....|....|....|....|....|....|....|....|....|....|....|....|....|....|....| |  |
| ppa008880m |  | ATTTCTTAGCTTTATGTAGTGGCTTTTACTTCGGTGACTTGAGCTTGAAAGATTCAACATAACTTTTCTAAACATTTAGGATTTAGGGCTTTGTTGTTCT |  |
| PRUAV007411 |  | ~~~~~~~~~~~~~~~~~~~~~~~~~~~~~~~~~~~~~~~~~~~~~~~~~~~~~~~~~~~~~~~~~~~~~~~~~~~~~~~~~~~~~~~~~~~~~~~~~~~~ |  |
| PavGCR1_‘Regina’ |  | ATTTCTTAGCTTTATGTAGTGGCTTTTACTTTGGTGACTTGAGCTTGAAAGATTCAACATAACTCTTCTAAACATTTAGGATTTAGGGCTTTGTTGTTCT |  |
| PavGCR1_‘Lapins’ |  | ATTTCTTAGCTTTATGTAGTGGCTTTTACTTTGGTGACTTGAGCTTGAAAGATTCAACATAACTCTTCTAAACATTTAGGATTTAGGGCTTTGTTGTTCT |  |
| PavGCR1_‘Garnet’ |  | ATTTCTTAGCTTTATGTAGTGGCTTTTACTTTGGTGACTTGAGCTTGAAAGATTCAACATAACTCTTCTAAACATTTAGGATTTAGGGCTTTGTTGTTCT |  |
|  |  |  |  |
|  |  | ....|....|....|....|....|....|....|....|....|....|....|....|....|....|....|....|....|....|....|....| |  |
| ppa008880m |  | TTAAGACTCGCTTCGTTCTAGTGATTAGCTTGAAGTTTGATTAAATTGGGAGCAAAAGAAAGCCATTTTATTGTTATTGTTACCGTCCACATGGTTAAGA |  |
| PRUAV007411 |  | ~~~~~~~~~~~~~~~~~~~~~~~~~~~~~~~~~~~~~~~~~~~~~~~~~~~~~~~~~~~~~~~~~~~~~~~~~~~~~~~~~~~~~~~~~~~~~~~~~~~~ |  |
| PavGCR1_‘Regina’ |  | TTAAGACTCGCTTCGTTCTAGTGTTTAGCTTAAAGTTTGATTAAATTGGGAACAGAAGAAAGCCATTTTATTGTTATTGTTACCGTCCACATGGTTAAGA |  |
| PavGCR1_‘Lapins’ |  | TTAAGACTCGCTTCGTTCTAGTGTTTAGCTTAAAGTTTGATTAAATTGGGAACAGAAGAAAGCCATTTTATTGTTATTGTTACCGTCCACATGGTTAAGA |  |
| PavGCR1_‘Garnet’ |  | TTAAGACTCGCTTCGTTCTAGTGTTTAGCTTAAAGTTTGATTAAATTGGGAACAGAAGAAAGCCATTTTATTGTTATTGTTACCGTCCACATGGTTAAGA |  |
|  |  |  |  |
|  |  | ....|....|....|....|....|....|....|....|....|....|....|....|....|....|....|....|....|....|....|....| |  |
| ppa008880m |  | ATATTTATTGCTTATTTTGTTATGCAGGATATGCTTTGTAGCTTCTTCAGCATGGTTGGGTAAGCTCGCTTCCCAGACACAAGTGCAGTCAGATAATATA |  |
| PRUAV007411 |  | ~~~~~~~~~~~~~~~~~~~~~~~~~~~gatatgctttgtagcttcttcagcatggttgg~~~~~~~~~~~~~~~~~~~~~~~~~~~~~~~~~~~~~~~~~ |  |
| PavGCR1_‘Regina’ |  | ATATTTATTGCTTATTTTGTTATGCAGGATATGCTTTGTAGCTTCTTCAGCATGGTTGGGTAAGCTCGCTTCCCAGACACAAGTGCAGTCAGATACTATA |  |
| PavGCR1_‘Lapins’ |  | ATATTTATTGCTTATTTTGTTATGCAGGATATGCTTTGTAGCTTCTTCAGCATGGTTGGGTAAGCTCGCTTCCCAGACACAAGTGCAGTCAGATACTATA |  |
| PavGCR1_‘Garnet’ |  | ATATTTATTGCTTATTTTGTTATGCAGGATATGCTTTGTAGCTTCTTCAGCATGGTTGGGTAAGCTCGCTTCCCAGACACAAGTGCAGTCAGATACTATA |  |
|  |  |  |  |
|  |  | ....|....|....|....|....|....|....|....|....|....|....|....|....|....|....|....|....|....|....|....| |  |
| ppa008880m |  | ACAAATTTATCTGATTCTGGCTTTTAAGTGATATAAGGGGAGTAATTTGCTCTTTTGTGCAGGGATCCATCCAGAGGATTTTTCTGTTACTCGCATGGCT |  |
| PRUAV007411 |  | ~~~~~~~~~~~~~~~~~~~~~~~~~~~~~~~~~~~~~~~~~~~~~~~~~~~~~~~~~~~~~~ggatccatccagaggatttttctgttactcgcatggct |  |
| PavGCR1_‘Regina’ |  | ACAAATTTATCTGATTCTGGCTTTTAAGTGATATAAGGGAAGTTAGTTGCTCTTTTGTGCAGGGATCCATCCAGAGGATTTTTCTGTTACTCGCATGGCT |  |
| PavGCR1_‘Lapins’ |  | ACAAATTTATCTGATTCTGGCTTTTAAGTGATATAAGGGAAGTTAGTTGCTCTTTTGTGCAGGGATCCATCCAGAGGATTTTTCTGTTACTCGCATGGCT |  |
| PavGCR1_‘Garnet’ |  | ACAAATTTATCTGATTCTGGCTTTTAAGTGATATAAGGGAAGTTAGTTGCTCTTTTGTGCAGGGATCCATCCAGAGGATTTTTCTGTTACTCGCATGGCT |  |
|  |  |  |  |
|  |  | ....|....|....|....|....|....|....|....|....|....|....|....|....|....|....|....|....|....|....|....| |  |
| ppa008880m |  | ATAGCACTCATTTCTTCTGTGTAGCATCTTTCCTATGGACTACCACGATTGCTTTTACCCTCCACCGCACGGTTGTTAGACACAAAACGGATGTTGAAGA |  |
| PRUAV007411 |  | atagcactcatttcttctgtgtagcatctttcctatggactaccacgattgcgtttaccctccaccgcaccgttgttagacacaaaacggatgttgaaga |  |
| PavGCR1_‘Regina’ |  | ATAGCACTCATTTCTTCTGTGTAGCATCTTTCCTATGGACTACCCAAATTGTCGCgtttta |  |
| PavGCR1_‘Lapins’ |  | ATAGCACTCATTTCTTCTGTGTAGCATCTTTCCTATGGACTACAGAAGGGGGTTTaa |  |
| PavGCR1_‘Garnet’ |  | ATAGCACTCATTTCTTCTGTGTAGCATCTTTCCTATGGACTACCCAAATTGGGGCGtaaaa |  |

***GH17***

|  |  | ....|....|....|....|....|....|....|....|....|....|....|....|....|....|....|....|....|....|....|....| |  |
| --- | --- | --- | --- |
| ppa007158m | 301 | ATGATGAAGAGCTTCTGGTTCTTTGCTCAGTTTCTTCACGTCTTCTTGGTCTTCTCCACTCCAATTGGTACCTTTCTTTTTTCTTTCATCAGATTGTTGT |  |
| PRUAV005464 |  | ATGATGAAGAGCTTCTGGTTCTTTGCTCAGTTTCTTCACGTCTTCTTGGTCTTCTCCACTCTAATT~~~~~~~~~~~~~~~~~~~~~~~~~~~~~~~~~~ |  |
| PavGH17_‘Regina’ |  | ~~~~~~~~~~~~~~~~~~~~~~~~~~~~~~~~~~~~~~~~~~~~~~~~~~~~~~~~~~~~~~~~~~~~~~~~~~~~~~~~~~~~~~~~~~~~~~~~~~~~ |  |
| PavGH17_‘Lapins’ |  | ~~~~~~~~~~~~~~~~~~~~~~~~~~~~~~~~~~~~~~~actcaag~~~~gggggGACTCTGATTCGATACCTTTCTTTTTTCTTTGATCAGATTGTTGT |  |
| PavGH17_‘Garnet’ |  | ~~~~~~~~~~~~~~~~~~~~~~~~~~~~~~~~~~~~~~~tgggggggttcggggcggactctgatCGATACCTTTCTTTTTTCTTTGATCAGATTGTTGT |  |
|  |  |  |  |
|  |  | ....|....|....|....|....|....|....|....|....|....|....|....|....|....|....|....|....|....|....|....| |  |
| ppa007158m |  | TATTCAATTGATTTGTGGAGTTGGGTTTTTATGGTTTTAACTGCTATCTGCTTGAATTTGTTGAATTGGATGGTTATAGAACAGTGTATATCTATTCTGT |  |
| PRUAV005464 |  | ~~~~~~~~~~~~~~~~~~~~~~~~~~~~~~~~~~~~~~~~~~~~~~~~~~~~~~~~~~~~~~~~~~~~~~~~~~~~~~~~~~~~~~~~~~~~~~~~~~~~ |  |
| PavGH17_‘Regina’ |  | ~~~~~~~~~~~~~~~~~~~~~~~~~~~~~~~~~~~~~~~~~~~~~~~~~~~~~~~atttgttgaattggatggttatagaacagtgtATATCTAGTCTGT |  |
| PavGH17_‘Lapins’ |  | TATTCAATTGATTTGTGGAGTTGGGTTTTTATGGTTTTAATTTCTATCTGCTTGAATTTGTTGAATTGGATGGTTATAGAACAGTGTATATCTATTCTGT |  |
| PavGH17_‘Garnet’ |  | TATTCAATTGATTTGTGGAGTTGGGTTTTTATGGTTTTAATTTCTATCTGCTTGAATTTGTTGAATTGGATGGTTATAGAACAGTGTATATCTATTCTGT |  |
|  |  |  |  |
|  |  | ....|....|....|....|....|....|....|....|....|....|....|....|....|....|....|....|....|....|....|....| |  |
| ppa007158m |  | GTGATTATTTACAGTGGGTAGTTGGTTTCTTGAAGAAATTGGGTTTAGATATTTCAGTGGTTATTGATTTTGATTGCTCTGTGATAGTTTCATATTAACA |  |
| PRUAV005464 |  | ~~~~~~~~~~~~~~~~~~~~~~~~~~~~~~~~~~~~~~~~~~~~~~~~~~~~~~~~~~~~~~~~~~~~~~~~~~~~~~~~~~~~~~~~~~~~~~~~~~~~ |  |
| PavGH17_‘Regina’ |  | GTGATYATTTATAGTGGGTAGTTGGTTTCTTGAAGAAATTGGGTTTAGATATTTCAGTGGTTATTGATTTTGATTGCTCTGTGATAGTTTCATATTAACA |  |
| PavGH17_‘Lapins’ |  | GTGATCATTTATAGTGGGTAGTTGGTTTCTTGAAGAAATTGGGTTTAGATATTTCAGTGGTTATTGATTTTGATTGCTCTGTGATAGTTTCATATTAACA |  |
| PavGH17_‘Garnet’ |  | GTGATCATTTATAGTGGGTAGTTGGTTTCTTGAAGAAATTGGGTTTAGATATTTCAGTGGTTATTGATTTTGATTGCTCTGTGATAGTTTCATATTAACA |  |
|  |  |  |  |
|  |  | ....|....|....|....|....|....|....|....|....|....|....|....|....|....|....|....|....|....|....|....| |  |
| ppa007158m |  | TTGGTCAATTTTGAGATTTGGGTATCTCTCTGTATCTGTTTTTAGGTGAGGATTGATTGGCTTTCTCCTTCATTGGTTATCTGAAGTTGTGTGTGTTTTT |  |
| PRUAV005464 |  | ~~~~~~~~~~~~~~~~~~~~~~~~~~~~~~~~~~~~~~~~~~~~~~~~~~~~~~~~~~~~~~~~~~~~~~~~~~~~~~~~~~~~~~~~~~~~~~~~~~~~ |  |
| PavGH17_‘Regina’ |  | TTGGTCAATCTTGAGAATTGGGTATCTCTCTGTATCTGTTTTTAGGTGAGGATTGTTTGGCTTTCTCCTTCATTGGTTATCTGAATT~GTGTGTGTGTTT |  |
| PavGH17_‘Lapins’ |  | TTGGTCAATCTTGAGAATTGGGTATCTCTCTGTATCTGTTTTTAGGTGAGGATTGTTTGGCTTTCTCCTTCATTGGTTATCTGAAGT~GTGTGTGTGTTT |  |
| PavGH17_‘Garnet’ |  | TTGGTCAATCTTGAGAATTGGGTATCTCTCTGTATCTGTTTTTAGGTGAGGATTGTTTGGCTTTCTCCTTCATTGGTTATCTGAAGT~GTGTGTGTGTTT |  |
|  |  |  |  |
|  |  | ....|....|....|....|....|....|....|....|....|....|....|....|....|....|....|....|....|....|....|....| |  |
| ppa007158m |  | TTTTTCCTTAGTGAAAGAGATTTGACTGAAGTTTCCGTGACTCTCCGCTAGATGCTGTAGTCTTTTCTCTTTCTGTTTTGTTCTTCTCAAATAGAATAAT |  |
| PRUAV005464 |  | ~~~~~~~~~~~~~~~~~~~~~~~~~~~~~~~~~~~~~~~~~~~~~~~~~~~~~~~~~~~~~~~~~~~~~~~~~~~~~~~~~~~~~~~~~~~~~~~~~~~~ |  |
| PavGH17_‘Regina’ |  | TTTTTCCTCAGTGAAAGAGATTTGACTGAAGTTTCTATGGCTCTCCGCCAAATGCTGTAGTCTTTTCTCTTTCTTTTTGGTTCTTCTCAAATAGAATAAT |  |
| PavGH17_‘Lapins’ |  | TTTTTCCTCAGTGAAAGAGATTTGACTGAAGTTTCTATGGCTCTCCGCCAGATGCTGTAGTCTTTTCTCTTTCTTTTTTGTTCTTCTCAAATAGAATAAT |  |
| PavGH17_‘Garnet’ |  | TTTTTCCTCAGTGAAAGAGATTTGACTGAAGTTTCTATGGCTCTCCGCCAGATGCTGTAGTCTTTTCTCTTTCTTTTTTGTTCTTCTCAAATAGAATAAT |  |
|  |  |  |  |
|  |  | ....|....|....|....|....|....|....|....|....|....|....|....|....|....|....|....|....|....|....|....| |  |
| ppa007158m |  | CTGTTTTCTTTATTATACAGGTTTATATAATTTTCCAGTTGATTACTCCTACTTTTCAAAGTGTACAGATGATACTACAGTCTTTTCAATGTTTAAGAAT |  |
| PRUAV005464 |  | ~~~~~~~~~~~~~~~~~~~~~~~~~~~~~~~~~~~~~~~~~~~~~~~~~~~~~~~~~~~~~~~~~~~~~~~~~~~~~~~~~~~~~~~~~~~~~~~~~~~~ |  |
| PavGH17_‘Regina’ |  | CTGTTTTCTTTATTATACAGGTTTACAAAATTTTCCAGTTGATTACTCCTACTTTTCAAAGTGTACAGATGATACTACAGTCTTTTCAATGTATAGGAAT |  |
| PavGH17_‘Lapins’ |  | CTGTTTTCTTTATTATACAGGTTTACAAAATTTTCCAGTTGATTACTCCTACTTTTCAAAGTGTACAGATGATACTACAGTCTTTTCAATGTATAGGAAT |  |
| PavGH17_‘Garnet’ |  | CTGTTTTCTTTATTATACAGGTTTACAAAATTTTCCAGTTGATTACTCCTACTTTTCAAAGTGTACAGATGATACTACAGTCTTTTCAATGTATAGGAAT |  |
|  |  |  |  |
|  |  | ....|....|....|....|....|....|....|....|....|....|....|....|....|....|....|....|....|....|....|....| |  |
| ppa007158m |  | CTTTGAAGATTAA~AAATAAAACAAAATCTACTTGTTTATGGTTATTGGGTATTATCACACGGTTTCATGAAAATTCAAAATGTTCAGTTT~AATGATTC |  |
| PRUAV005464 |  | ~~~~~~~~~~~~~~~~~~~~~~~~~~~~~~~~~~~~~~~~~~~~~~~~~~~~~~~~~~~~~~~~~~~~~~~~~~~~~~~~~~~~~~~~~~~~~~~~~~~~ |  |
| PavGH17_‘Regina’ |  | CTTTGAAGATTAA~AAATAAAACAAA~TCTACTtgtttatggttattggtttttatcacacggtttcatgaaaattcataatgttcatttttaatgattc |  |
| PavGH17_‘Lapins’ |  | CTTTGAAGATTAA~AAATAAAACAAAATCTACTTGTTTATGGTTATTGGGTTTTATCACACGGTTTCATGAAAATTCAAAATGTTCAGTTT~AATGATTC |  |
| PavGH17_‘Garnet’ |  | CTTTGAAGATTAAGAAATAAAACAAAATCTACTTGTTTATGGTTATTGGGTTTTATCACACGGTTTCATGAAAATTCAAAATGTTCAGTTT~AGTGATTC |  |
|  |  |  |  |
|  |  | ....|....|....|....|....|....|....|....|....|....|....|....|....|....|....|....|....|....|....|....| |  |
| ppa007158m |  | CATTTGAAGTGGTGTGCTTTCCTTGAAATTGTATAAACAGAAGAGATCTGTCTCCCTTCCCATTAAAAAAAGAAGATGGAGAGCCT~ATTTTACGGTGGA |  |
| PRUAV005464 |  | ~~~~~~~~~~~~~~~~~~~~~~~~~~~~~~~~~~~~~~~~~~~~~~~~~~~~~~~~~~~~~~~~~~~~~~~~~~~~~~~~~~~~~~~~~~~~~~~~~~~~ |  |
| PavGH17_‘Regina’ |  | catttgaagtggtgtgctttccttgaa~ttgtataaacagaaaacatctgcctcccttcccattaaaaaaagaatatggatagccttattttatggtgaa |  |
| PavGH17_‘Lapins’ |  | CATTTGAAGTGGTGTGCTTTCCTTGAA~TTGTATAAACAGAAGAGATCTGCCTCCCTTCCCATTAAAAAAAGAAGATGGAGAGCCT~ATTTTATGGTGGA |  |
| PavGH17_‘Garnet’ |  | CATTTGAAGTGGTGTGCTTTCCTTGAA~TTGGATAA~CAGAATagatctgcctcccttcccattaaaaaa~gaagatggatagcct~attttatggtgta |  |
|  |  |  |  |
|  |  | ....|....|....|....|....|....|....|....|....|....|....|....|....|....|....|....|....|....|....|....| |  |
| ppa007158m |  | ATTAAGCCCAAACTGGTTCTCTACGAATCTCTGTGGTTGGTTGACTGGAATAATTGCTATGCTAATTTGAG~TGTAAAATATGGC~ACATAATATGTTGC |  |
| PRUAV005464 |  | ~~~~~~~~~~~~~~~~~~~~~~~~~~~~~~~~~~~~~~~~~~~~~~~~~~~~~~~~~~~~~~~~~~~~~~~~~~~~~~~~~~~~~~~~~~~~~~~~~~~~ |  |
| PavGH17_‘Regina’ |  | at~aagcccaa~ctggttctctaccaatctctgtgcttggttgactggaacacttgctatgctaattagagatgtaaaatatggc~acataatatgttgc |  |
| PavGH17_‘Lapins’ |  | AT~AAGCCCAAACTGGTTCTCTACCAATCTCTGTGCTTGGTTGACTGGAATAATTGCTATGCTAATTTGAG~TGTAAAATATGGC~ACATAATATGTTGC |  |
| PavGH17_‘Garnet’ |  | at~a~gcccaaactggttctctaccaatccctgtgcttggttgactggaataattgctatgctaatctga~atggaaattatggccacataataggttgc |  |
|  |  |  |  |
|  |  | ....|....|....|....|....|....|....|....|....|....|....|....|....|....|....|....|....|....|....|....| |  |
| ppa007158m |  | CATTGTCTTT~CATTACCACATG~AACAAATGGTTGATGGTGCTGTTTGTGTTCTTTCC~CAGCC~TCT~GTGGCAGTACAAGCATTCACTGGAGCCTAT |  |
| PRUAV005464 |  | ~~~~~~~~~~~~~~~~~~~~~~~~~~~~~~~~~~~~~~~~~~~~~~~~~~~~~~~~~~~~~~GCC~TCT~GTGGCAGTACAAGCATTCACTGGAGCCTAT |  |
| PavGH17_‘Regina’ |  | cattgttttt~cattaccacttg~aacaaatggttgatggcgctgtttgtgtcctttccacagcc~tct~gtggcagtacac~cattcactggagcctat |  |
| PavGH17_‘Lapins’ |  | CATTGTTTTT~CATTACCACTTG~AACAAATGGTTGATGGTGCTGTTTGTGTTCTTTCC~CAGCC~TCT~GTGGCAGTACAAGCATTCACTGGAGCCTAT |  |
| PavGH17_‘Garnet’ |  | cattgttttttcataaccacttgcaacaa~tggt~gatgggcctgtt~gggttctttcc~~agccctcttgtgccgat~caagcattc~ctggagcctat |  |
|  |  |  |  |
|  |  | ....|....|....|....|....|....|....|....|....|....|....|....|....|....|....|....|....|....|....|....| |  |
| ppa007158m |  | GGAATAAATTATGGAAGAATTGCAGATAACATCCCTTCTCCTGATAAAGTTGCCACTCTTCTCAGAGCAGCAAAGATAAAGAATGTCAGGATATATGATG |  |
| PRUAV005464 |  | GGAATAAATTATGGAAGAATTGCAGATAACATCCCTTCTCCTGATAAAGTTGCTACTCTTCTCAGAGCAGCAAAGATAAAGAATGTCAGGATATATGATG |  |
| PavGH17_‘Regina’ |  | ggaacaatcacggaagactgcacataca~~~tcccttccactgataaattgctactctctcgaacagctaagatta~a~ctatcgccaagataatgaatg |  |
| PavGH17_‘Lapins’ |  | GGAATAAATTATGGAAGA~TTGCAGATAACATCCCTTCTCCTGATAAAGTTGCTACTCTTCTCAGAGCAGCAAAGATAACGATGTCAAGGATAtatgatg |  |
| PavGH17_‘Garnet’ |  | ggaataattatggagatgcgagaacat~~~~tccct~ctctggacagttgtcacctctctcgagcagcaagacatacatgtcagaaatatttatgcttga |  |
|  |  |  |  |
|  |  | ....|....|....|....|....|....|....|....|....|....|....|....|....|....|....|....|....|....|....|....| |  |
| ppa007158m |  | CTGATCACAGTGTTCTCAAGGCTTTTAGCGGGACTGGGCTTGATTTAGTGGTAGGACTTCCAAATGGATACCTGAAAGACATGAGTGCCAATGAGGACCA |  |
| PRUAV005464 |  | CTGATCACAGTGTTCTCAAGGCTTTTAGCGGGACTGGGCTTGATTTAGTGGTAGGACTTCCAAATGGATACCTGAAGGACATGAGTGCCAATGAGGACCA |  |
| PavGH17_‘Regina’ |  | ctgatcacagtggtcccaaggccttttatcgggacctaacttgatttatggtaggacttccaatggatacctggaaggacttgcaatgccaatgaggacc |  |
| PavGH17_‘Lapins’ |  | ctgatcacagtg~tctcaaggcctttagcgggactgggctgattagtgtagacttccaaatgctactgagacatgatgcatgagaccatgcatgaatggt |  |
| PavGH17_‘Garnet’ |  | tatccagttccctgtcttacgagacgtgtctgtattaggcgagctccaatggtacggagactattgcctaggactccgtatgtgtctaatggatctcgag |  |
|  |  |  |  |
|  |  | ....|....|....|....|....|....|....|....|....|....|....|....|....|....|....|....|....|....|....|....| |  |
| ppa007158m |  | TGCAATGGATTGGGTTAAAGAAAATGTGCAGGCGTTCCTTCCTGAGACACGAATCCGTGGGATTGCTGTGGGCAATGAAGTATTAGGTGGGACTGATTAT |  |
| PRUAV005464 |  | TGCAATGGATTGGGTTAAAGAAAATGTGCAGGCATTCCTTCCTGAAACACGCATTCGTGGGATTGCTGTGGGCAATGAAGTGTTAGGTGGGACCGATTAT |  |
| PavGH17_‘Regina’ |  | agagctatgggattgggttaaagaacaatggtgcggccattcttcctggaaccgcaatttctggcaattggcagatggccactctaatgcctaggcgtgg |  |
| PavGH17_‘Lapins’ |  | aagcaaatgtgcagcatcctactgatcacgcatcctgcatgctagcatgaggtagtgaacgattagaatgtgggggagctcttttttaac |  |
| PavGH17_‘Garnet’ |  | actcatctcggttggat |  |
|  |  |  |  |
|  |  | ....|....|....|....|....|....|....|....|....|....|....|....|....|....|....|....|....|....|....|....| |  |
| ppa007158m |  | GAATTGTGGGGAGCTCTGTTGGGTGCAGTTAAAAATATCTATAATGCAATAAAGGAGCTAGGATTAACTGATGTAGTTCAGATTACCACTGCCCATTCAC |  |
| PRUAV005464 |  | GAATTGTGGGGAGCTCTTTTGGGCGCAGTTAAAAATATCTATAATGCAATAAAGGAGCTAGGATTAACTGATGTAGTTCAGATTACCACTGCCCATTCAC |  |
| PavGH17_‘Regina’ |  | ttaccgaatattagaaatggtggggaagctccatttaagcgtgcacgacgtcgtaag |  |
| PavGH17_‘Lapins’ |  |  |  |
| PavGH17_‘Garnet’ |  |  |  |
|  |  |  |  |
|  |  | ....|....|....|....|....|....|....|....|....|....|....|....|....|....|....|....|....|....|....|....| |  |
| ppa007158m |  | AGGCTGTTTTTGCTAATTCCTTCCCTCCCTCTTCCTGTATATTCAGAGATAATGTTAAACAGCAATACATGAAGCCACTTTTGGAGTTCTTCTCAGAAAT |  |
| PRUAV005464 |  | AGGCTGTTTTTGCTAATTCCTTCCCTCCCTCTTCCTGTATATTCAGAGATAATGTTAAACAGCAATACATGAAGCCACTTTTGGAGTTCTTCTCAGAAAT |  |
| PavGH17_‘Regina’ |  |  |  |
| PavGH17_‘Lapins’ |  |  |  |
| PavGH17_‘Garnet’ |  |  |  |
|  |  |  |  |
|  |  | ....|....|....|....|....|....|....|....|....|....|....|....|....|....|....|....|....|....|....|....| |  |
| ppa007158m |  | CGGGTCTCCTTTCTGTTTAAATGCTTACCCATTCCTTGCCTACATGAGTGATCCGGAGAACATTGATATTAATTATGCTCTTTTCCAGAAAACTCAGGGG |  |
| PRUAV005464 |  | TGGGTCTCCTTTCTGTTTAAATGCTTACCCATTCCTTGCCTACATGAGTGATCCGGAGAACATTGATATTAATTATGCTCTTTTCCAGAAAACTCAGGGG |  |
| PavGH17_‘Regina’ |  |  |  |
| PavGH17_‘Lapins’ |  |  |  |
| PavGH17_‘Garnet’ |  |  |  |
|  |  |  |  |
|  |  | ....|....|....|....|....|....|....|....|....|....|....|....|....|....|....|....|....|....|....|....| |  |
| ppa007158m |  | ATTTCTGATCCAAAGACTGACCTTCATTATGATAACATGCTTGATGCTCAGATTGATGCAGCCTATGCAGCATTGGAAGATACTGGA~~~TTCAAAAAGA |  |
| PRUAV005464 |  | ATTTCTGATCCAAAGACTGACCTTCATTATGATAACATGCTTGATGCTCAGATTGATGCAGCCTATGCAGCATTGGAAGATGCTNNGGATTACAAAAAGA |  |
| PavGH17_‘Regina’ |  |  |  |
| PavGH17_‘Lapins’ |  |  |  |
| PavGH17_‘Garnet’ |  |  |  |

***GH17-39***

|  |  | ....|....|....|....|....|....|....|....|....|....|....|....|....|....|....|....|....|....|....|....| |  |
| --- | --- | --- | --- |
| ppa007999m | 1 | AATGTAGAGAGTGAGTTATTTATACAATTCCCTCTTCTTCATGGATTTCCCTACACAAGCTAGAACAGTTCCAAAAACTCTTATGGCTTCCATATTGCTG |  |
| PRUAV000077 |  | ~~GGTAGAGAGTGAGTTATTTATACAATTTCCTCTTCTTCATGGATTTCCCTACACAAGCTAGAACAGTTCCAAAAACTCTCATGGCTTCCATATTGCTG |  |
| PavGH17-39_‘Regina’ |  | ~~~~~~~~~~~~~~~~~~~~~~~~~~~~~~~~~~~~~~~~~~~~~~~~~~~~~~~~~~~~~~~~~~~~~~~~~~~~~~~~~~~~~~~~~~~~~caatgat |  |
| PavGH17-39_‘Lapins’ |  | ~~~~~~~~~~~~~~~~~~~~~~~~~~~~~~~~~~~~~~~~~~~~~~~~~~~~~~~~~~~~~~~~~~~~~~~~~~~~~~~~~~~~~~~~~~~~~~~ctaaa |  |
| PavGH17-39_‘Garnet’ |  | ~~~~~~~~~~~~~~~~~~~~~~~~~~~~~~~~~~~~~~~~~~~~~~~~~~~~~~~~~~~~~~~~~~~~~~~~~~~~~~~~~~~~~~~~~~~~~ggtcact |  |
|  |  |  |  |
|  |  | ....|....|....|....|....|....|....|....|....|....|....|....|....|....|....|....|....|....|....|....| |  |
| ppa007999m |  | CTACTTGTATTGTTGATGCCTGCCCTGCAAATAACAGGTCTCACTTTCTGCTTCTGAATTTATATATATATATAGATATATATATATATATATATATATA |  |
| PRUAV000077 |  | CTACTTGTATTGTTGATGCCTGCCCTGCAAATAACAGGT~~~~~~~~~~~~~~~~~~~~~~~~~~~~~~~~~~~~~~~~~~~~~~~~~~~~~~~~~~~~~ |  |
| PavGH17-39_‘Regina’ |  | ggtatgctgcgccatcgtggtatagctgcaaccaaggtggttatgctgcaaccaaggtgtctatgctgcgtgcaacggttgatgtgagcgttatttcttc |  |
| PavGH17-39_‘Lapins’ |  | atgtggtatgctgcgccaccgtggtatgctgcaccacgatggttttgctgcaaacacgtgttctatggtgggtgggtagttgatgtgaggttATTTCTTC |  |
| PavGH17-39_‘Garnet’ |  | gtggtatgctgcaccacggtggtatgctgcaaccaaggtggttatgctgcaaccaaggtgtcaatgctgcaaccaaggattgatgctgcgacaattcttc |  |
|  |  |  |  |
|  |  | ....|....|....|....|....|....|....|....|....|....|....|....|....|....|....|....|....|....|....|....| |  |
| ppa007999m |  | TGTCAT~~~~~CAAAGCAAA~~~~~~~~~~~~~~TATATTAAGTAATTCTGTTTCGTTATGTAGTTGAAATTATGATATAT~~~~~~~~~~~~~TACCAT |  |
| PRUAV000077 |  | ~~~~~~~~~~~~~~~~~~~~~~~~~~~~~~~~~~~~~~~~~~~~~~~~~~~~~~~~~~~~~~~~~~~~~~~~~~~~~~~~~~~~~~~~~~~~~~~~~~~~ |  |
| PavGH17-39_‘Regina’ |  | aatcatgtttacAGTACAAGAGTTTGAGGTAGATTATATTAAGTATTTCTGTTTCATTATATAGTTGAAATTATGATATATTCAAGCATATAAATACCAT |  |
| PavGH17-39_‘Lapins’ |  | AATCATGTTTACAGTACAAGAGTTTGAGGTAGATTATATTAAGTATTTCTGTTTCATTATATAGTTGAAATTATGATATATTCAAGCATATAAATACCAT |  |
| PavGH17-39_‘Garnet’ |  | aatcatgtttacaaaacaaGAGTTTGAGGTACATTATATTAAGTATTTCTGTTTCATTATATAGTTGAAATTATGATATATTCAAGCATATAWATACCAT |  |
|  |  |  |  |
|  |  | ....|....|....|....|....|....|....|....|....|....|....|....|....|....|....|....|....|....|....|....| |  |
| ppa007999m |  | GCAGAAAACTTCTGGTTTCTCTATCCAATTTTTATTTATTTTTATTTTAAAATACAAGCCATATTGGGGGAAGGGGGATTCGAACATAGGACCTTAGATG |  |
| PRUAV000077 |  | ~~~~~~~~~~~~~~~~~~~~~~~~~~~~~~~~~~~~~~~~~~~~~~~~~~~~~~~~~~~~~~~~~~~~~~~~~~~~~~~~~~~~~~~~~~~~~~~~~~~~ |  |
| PavGH17-39_‘Regina’ |  | GCAGAAAACTTCTGGTTTCTTTATCCAATTTTTATTT~~~~~~~~~~TAAAATACAAGCCATATTGGGGGAGGGGGGATTCGAACATAGAACCTCGGATG |  |
| PavGH17-39_‘Lapins’ |  | GCAGAAAACTTCTGGTTTCTTTATCCAATTTTTATTT~~~~~~~~~~TAAAATACAAGCCATATTGGGGGAGGGGGGATTCGAACATAGAACCTCGGATG |  |
| PavGH17-39_‘Garnet’ |  | GCAGAAAACTTCTGGTTTCTTTATCCAATTTTTATTT~~~~~~~~~~TAAAATACAAGCCATATTGGGGGAGGGGGGATTCGAACATAGAACCTCGGATG |  |
|  |  |  |  |
|  |  | ....|....|....|....|....|....|....|....|....|....|....|....|....|....|....|....|....|....|....|....| |  |
| ppa007999m |  | CAAGGGTGACTACTCTTAACCACTTGAGTTACCCGTCCCTTGCTTCTCTATCCAAATTCTGAATTAGAAGCATTTGGAGTTGTATGTAATTTCTTTGGTC |  |
| PRUAV000077 |  | ~~~~~~~~~~~~~~~~~~~~~~~~~~~~~~~~~~~~~~~~~~~~~~~~~~~~~~~~~~~~~~~~~~~~~~~~~~~~~~~~~~~~~~~~~~~~~~~~~~~~ |  |
| PavGH17-39_‘Regina’ |  | CAAGGGTGACTACTCTTAACCACTTGAGTTATATGTCCCTTGCTTCTCTATCCAAATTCTGAATTAGAAGCAACTGGAGTTGTATGTAATTTCATTGGTC |  |
| PavGH17-39_‘Lapins’ |  | CAAGGGWGACTACTCTTAACCACTTGAGTTATATGTCCCTTGCTTCTCTATCCAAATTCTGAATTAGAAGCAACTGGAGTTGTATGTAATTTCATTGGTC |  |
| PavGH17-39_‘Garnet’ |  | CAAGGGTGACTACTCTTAACCACTTGAGTTATATGTCCCTTGCTTCTCTATCCAAATTCTGAATTAGAAGCAACTGGAGTTGTATGTAATTTCATTGGTC |  |
|  |  |  |  |
|  |  | ....|....|....|....|....|....|....|....|....|....|....|....|....|....|....|....|....|....|....|....| |  |
| ppa007999m |  | ATTTCATGTATGAAATTAAAATTGTCATCCTTTAAAATATTCTTTAAGCAAGTTTTTGCTCTTGTTGCCAATCATGCTTTCACAAGCTGAAACTTTGATG |  |
| PRUAV000077 |  | ~~~~~~~~~~~~~~~~~~~~~~~~~~~~~~~~~~~~~~~~~~~~~~~~~~~~~~~~~~~~~~~~~~~~~~~~~~~~~~~~~~~~~~~~~~~~~~~~~~~~ |  |
| PavGH17-39_‘Regina’ |  | ATTTCATGTATGAAATTGAAATTGTCATCCTTTAAAACATTCTTTAAGCAAGTTGTTTCTCTTGTTGCCAATCATGCTTTCACAAACTGAAACTTTGATG |  |
| PavGH17-39_‘Lapins’ |  | ATTTCATGTATGAAATTGAAATTGTCATCCTTTAAAACATTCTTTAAGCAAGTTGTTTCTCTTGTTGCCAATCATGCTTTCACAAACTGAAACTTTGATG |  |
| PavGH17-39_‘Garnet’ |  | ATTTCATGTATGAAATTGAAATTGTCATCCTTTAAAACATTCTTTAAGCAAGTTGTTTCTCTTGTTGCCAATCATGCTTTCACAAACTGAAACTTTGATG |  |
|  |  |  |  |
|  |  | ....|....|....|....|....|....|....|....|....|....|....|....|....|....|....|....|....|....|....|....| |  |
| ppa007999m |  | CTTGTATTTCAGGTGCACAATCTGTTGGTGTTTGTTATGGACGAAATGGCAACAATTTACCATCCGAATCAGACGTCGTTGGCTTGTACAAAAGCAATGG |  |
| PRUAV000077 |  | ~~~~~~~~~~~~~~GCACAATCTGTTGGTGTTTGTTATGGACGAAATGGCAACAATTTACCATCCGAAACAGAAGTCGTTGAGTTGTACAAAAGCAATGG |  |
| PavGH17-39_‘Regina’ |  | CTTGTATTTCAGGTGCACAATCTGTTGGTGTTTGTTATGGACGAAATGGCAACAATTTACCATCCGAAACAGAAGTCGTTGAGTTGTACAAAAGCAATGG |  |
| PavGH17-39_‘Lapins’ |  | CTTGTATTTCAGGTGCACAATCTGTTGGTGTTTGTTATGGACGAAATGGCAACAATTTACCATCCGAAACAGAAGTCGTTGAGTTGTACAAAAGCAATGG |  |
| PavGH17-39_‘Garnet’ |  | CTTGTATTTCAGGTGCACAATCTGTCGGTGTTTGTTATGGACGAAATGGCAACAATTTACCATCCGAAACAGAAGTCGTTGACTTGTACAAAAGCAATGG |  |
|  |  |  |  |
|  |  | ....|....|....|....|....|....|....|....|....|....|....|....|....|....|....|....|....|....|....|....| |  |
| ppa007999m |  | CATTGGAAGAATGAGAATTTATGAACCCAATGATCCAACCTATCAAGCCCTTAAAGGTTCCAACATAGAACTCACCGTGACCATCCTCAAAAGCCAGCTT |  |
| PRUAV000077 |  | CATTGGAAGAATGAGAATTTATGAACCCAATGATCCAACCTATCAAGCCCTTAAAGGTTCCAACATAGAACTCACCGTGACCATCCTCAATAACCAGCTT |  |
| PavGH17-39_‘Regina’ |  | CATTGGAAGAATGAGAATTTATGAACCCAATGATCCAACCTATCAAGCCCTTAAAGGTTCCAACATAGAACTCACCGTGACCATCCTCAATAACCAGCTT |  |
| PavGH17-39_‘Lapins’ |  | CATTGGAAGAATGAGAATTTATGAACCCAATGATCCAACCTATCAAGCCCTTAAAGGTTCCAACATAGAACTCACCGTGACCATCCTCAATAACCAGCTT |  |
| PavGH17-39_‘Garnet’ |  | CATTGGAAGAATGAGAATTTATGAACCCAATGATCCAACCTATCAAGCCCTTAAAGGTTCCAACATAGAACTCACCGTGACCATCCTCAACAACCAGCTT |  |
|  |  |  |  |
|  |  | ....|....|....|....|....|....|....|....|....|....|....|....|....|....|....|....|....|....|....|....| |  |
| ppa007999m |  | CAAGGCCTCACTGATGCTGCTGCTGCAACAGATTGGGTCCAAAAGAATGTACAAGCCTACTCGCCTGACGTCAAGTTCAAATACATTGCGGTCGGGAACG |  |
| PRUAV000077 |  | CAAAGCCTCACTGATGCTGCTGCTGCAACAGATTGGGTCCAAAAGAATGTACAAGCCTACTTGCCTGACGTCAAGTTCAAATACATTGCGGTCGGGAACG |  |
| PavGH17-39_‘Regina’ |  | CAAAGCCTCACTGATGCTGCTGCTGCAACAGATTGGGTCCAAAAGAATGTACAAGCCTACTTGCCTGACGTCAAGTTCAAATACATTGCGGTCGGGAACG |  |
| PavGH17-39_‘Lapins’ |  | CAAAGCCTCACTGATGCTGCTGCTGCAACAGATTGGGTCCAAAAGAATGTACAAGCCTACTTGCCTGACGTCAAGTTCAAATACATTGCGGTCGGGAACG |  |
| PavGH17-39_‘Garnet’ |  | CAAAGCCTCACTGATGCTGCTGCTGCAACAGATTGGGTCCAAAAGAATGTACAAGCCTACTTGTCTGACGT~AAGTTCAAattcattgcggtcgggaacg |  |
|  |  |  |  |
|  |  | ....|....|....|....|....|....|....|....|....|....|....|....|....|....|....|....|....|....|....|....| |  |
| ppa007999m |  | AAGTACGCCCCACGGATCCAGAGACCCAGTATCTCCTCCCAGCCATCCAAAACATTCATAATGCAATTGTAGCAGCCAATCTGCAAGGCCAGATCAAAGT |  |
| PRUAV000077 |  | AAGTACACCCCGGCGATACAGTGACCCAGTATCTCTTCCCAGCCATCCAAAACATTTATAATGCAATTGTAGCAGCCAATCTGCAAGGCCAGATCAAAGT |  |
| PavGH17-39_‘Regina’ |  | AAGTACACCCCGGCGATACAGTGACCCAGTATCTCTTCCCAGCCATCCAAAACATTTATAATGCAATTGTAGCAGCCAATCTGCAAGGCCAGATCAAAGT |  |
| PavGH17-39_‘Lapins’ |  | AAGTACACCCCGGCGATACAGTGACCCAGTATCTCTTTCCCAGCCATCCAAAACATTTATAATGCAATTTGTAGCAGCCATtctgcaaggccagatcaaa |  |
| PavGH17-39_‘Garnet’ |  | aagtacactccggcgatacagtgacccaatatctcctccccgccctcacaaacatttataatgcaactgtaccaccatatctgcgcgctagatcatagtc |  |
|  |  |  |  |
|  |  | ....|....|....|....|....|....|....|....|....|....|....|....|....|....|....|....|....|....|....|....| |  |
| ppa007999m |  | CTCAACAGCAATTGACACAACCCTTGTGGACAATGCCTACCCTCCTTCAGCTGGAAAATATAGTGATGCTGCAAAGTCATTCATAACCCCAGTTATCAAC |  |
| PRUAV000077 |  | CTCAACAGCAATTGACACAACCCTTGTGGACAATGCCTTCCCTCCTTCAGCTGGAAAATATAGTGATGATGCAAAGTCATTCATAACCCCAGTTATCAAC |  |
| PavGH17-39_‘Regina’ |  | CTCAACAGCAATTGACACAACCCTTGTGGACAATGCCTTCCCTCCTTCAGCTGGGAAAATATAGTGATGATGCAAAGTCATTCATAACCCCAGTTATCAA |  |
| PavGH17-39_‘Lapins’ |  | gtctcaacagcaattgacacaaccctttgtggacaatgccttccctccttcagctggaaaatatagtgatgatgcaagtcatttcataaccccagttatc |  |
| PavGH17-39_‘Garnet’ |  | ttaacagctaataacaccacccat |  |
|  |  |  |  |
|  |  | ....|....|....|....|....|....|....|....|....|....|....|....|....|....|....|....|....|....|....|....| |  |
| ppa007999m |  | TTCCTAGCCAGCAATGGGGCCCCTCTTCTTGTCAATGTGTATCCTTACGTCAGCTACACTGAAAATCCTTCTCAAATAGACATTGCCTATGCCTTATTCA |  |
| PRUAV000077 |  | TTCCTAGCCAGCAATGGGGCCCCTCTTCTTGTCAATGTGTATCCTTACTTCAGCTACATTCTAAATACTGCTCAAATAGACCTTGCCTATGCCTTATTCA |  |
| PavGH17-39_‘Regina’ |  | CTTCCTAGCCAGCAATGGGGCCCCTCTTCTTGTCAATGTGTATCCTTACTTCAGCTACATTCTAAATACTGCTCAAATAGACCTTgcctatgccttattc |  |
| PavGH17-39_‘Lapins’ |  | acttctagccagcaatggggccccatcttcctggtcaatgtgtatccctacttccacctcctttctaaatactgctcaaatagaccttgcctaagcccta |  |
| PavGH17-39_‘Garnet’ |  |  |  |
|  |  |  |  |
|  |  | ....|....|....|....|....|....|....|....|....|....|....|....|....|....|....|....|....|....|....|....| |  |
| ppa007999m |  | CCTCACAAGGGATCACGACACCCGACGGTGTAAAGTACCAAAACCTGTTTGATGCTCTTTTGGATGCTCAGTACTCTGCTCTCGAGAAAGCCAATGCTCC |  |
| PRUAV000077 |  | CCTCAGATGGGATCACGACGCCCGACGGTGTAAAGTACCAAAACCTGTTTGATGCTCTTTTGGATGCTCAGTACTCGGCTCTTGAGAAAGCCAATGCTCC |  |
| PavGH17-39_‘Regina’ |  | acctcagatgggatcacgacgcccgacggtgtaaagtaccaaatctgttgatgcctcttttgatgcctcagtacctcggctctggaaagccatgcttcca |  |
| PavGH17-39_‘Lapins’ |  | ttccacctagatgggtacacgacccccgatcgtgtaaagaacccaacctgttcaagctctttttgaatgcctagtactcggtctttgaaaagccaatgcc |  |
| PavGH17-39_‘Garnet’ |  |  |  |
|  |  |  |  |
|  |  | ....|....|....|....|....|....|....|....|....|....|....|....|....|....|....|....|....|....|....|....| |  |
| ppa007999m |  | CAACGTGGAGATCGTCGTGTCGGAGAGTGGTTGGCCATCCGAAGGCAGTGATGCCGCAACCACTCAAAATGCACAAACATTCTACCAGAATTTGATCAAC |  |
| PRUAV000077 |  | CAACGTGGAGATCGTCGTGTCAGAGAGCGGTTGGCCATCCGAAGGTGGTAATGCTGCAACCCCTCAAAATGCACAAATATTCTACCAGAATTTGATCAAC |  |
| PavGH17-39_‘Regina’ |  | aacgtggaaatcgtcagtcgaaagcgattgcatccgagggggtgtaatgcctgcaccacggggctta |  |
| PavGH17-39_‘Lapins’ |  | tctccaccgtgaatacgctcggtacagagacgtgcctgtgactactcgaaagggtgtgaatggcttgcacccacaacg |  |
| PavGH17-39_‘Garnet’ |  |  |  |

***GH17-44***

|  |  | ....|....|....|....|....|....|....|....|....|....|....|....|....|....|....|....|....|....|....|....| |  |
| --- | --- | --- | --- |
| ppa024457m | 290 | GAAGTGAGCCCGGTTTATGGAGACACTACTAGGCTGGCTCAATTTGTCCTCCCCGCAATGAAGAACATATACAACGCAATCAGATCAGCTGGCCTTCAAG |  |
| PRUAV004835 |  | GAAGTGAGCCCGGTTTATGGAGACACTACTAGGCTGGCTCAGTTTGTCCTCCCCGCAATGAAGAACATATACAATGCAATCAGATCAGCTGGCCTTCAAG |  |
| PavGH17-44_‘Regina’ |  | ~~~~~~~~~~~~~~~~~~~~~~~~~~~~~~~~~~~~~~~~~~~~~~~~~~~~~~~~~~~~~gggagaATATACATGC~ATCAGATCAGCTGGCCTTCAAG |  |
| PavGH17-44_‘Lapins’ |  | ~~~~~~~~~~~~~~~~~~~~~~~~~~~~~~~~~~~~~~~~~~~~~~~~~~~~~~~~~~~~~ggggtaaTGTACATGC~ATCAGATCAGCTGGCCTTCAAG |  |
| PavGH17-44_‘Garnet’ |  | ~~~~~~~~~~~~~~~~~~~~~~~~~~~~~~~~~~~~~~~~~~~~~~~~~~~~~~aaggccctaaaagaagttcATGCAATCAGATCAGCTGGCCTTCAAG |  |
|  |  |  |  |
|  |  | ....|....|....|....|....|....|....|....|....|....|....|....|....|....|....|....|....|....|....|....| |  |
| ppa024457m |  | ACCAAATCAAGGTCTCAACTGCCATAGAGACTGGATTGATAGGCAACTCCTACCCTCCGTCACAAGGCGCTTTTCGCGGCGATGTGAGAGCTTATTTGGA |  |
| PRUAV004835 |  | ACCAAATCAAGGTCTCAACTGCCATAGAGACGGGATTGATAGGCAACTCCTACCCTCCGTCACAAGGAGCTTTTCGGGGCGATGTGAGAGCTTATTTGGA |  |
| PavGH17-44_‘Regina’ |  | ACCAAATCAAGGTCTCAACTGCCATAGAGACGGGATTGATAGGCAACTCCTACCCTCCGTCACAGGGAGCTTTTCGGGGCGATGTGAGAGCTTATTTGGA |  |
| PavGH17-44_‘Lapins’ |  | ACCAAATCAAGGTCTCAACTGCCATAGAGACGGGATTGATAGGCAACTCCTACCCTCCGTCACAGGGAGCTTTTCGGGACGATGTGAGAGCTTATTTGGA |  |
| PavGH17-44_‘Garnet’ |  | ACCAAATCAAGGTCTCAACTGCCATAGAGACGGGATTGATAGGCAACTCCTACCCTCCGTCACAAGGAGCTTTTCGGGGCGATGTGAGAGCTTATTTGGA |  |
|  |  |  |  |
|  |  | ....|....|....|....|....|....|....|....|....|....|....|....|....|....|....|....|....|....|....|....| |  |
| ppa024457m |  | CCCAATTATAGGGTTCTTGGTTTATGCCAAATCACCACTACTTGCTAACATCTATACATATTTTAGTTATATTGGAAATCCTAGAGATATTTCTCTTCCT |  |
| PRUAV004835 |  | CCCAATTATAGGGTTCTTGGTTTATGCCAAATCACCACTACTTCCTAACATCTATACATATTTTAGTTATATTGGAAATCCTAGAGATATTTCTCTTCCT |  |
| PavGH17-44_‘Regina’ |  | CCCAATTATAGGGTTCTTGGTTTATGCCAAATCACCACTACTTSCTAACATCTATACATATTTTAGTTATATTGGAAATCCTAGAGATATTTCTCTTCCT |  |
| PavGH17-44_‘Lapins’ |  | CCCAATTATAGGGTTCTTGGTTTATGCCAAATCACCACTACTTGCTAACATCTATACATATTTTAGTTATATTGGAAATCCTAGAGATATTTCTCTTCCT |  |
| PavGH17-44_‘Garnet’ |  | CCCAATTATAGGGTTCTTGGTTTATGCCAAATCACCACTACTTGCTAACATCTATACATATTTTAGTTATATTGGAAATCCTAGAGATATTTCTCTTCCT |  |
|  |  |  |  |
|  |  | ....|....|....|....|....|....|....|....|....|....|....|....|....|....|....|....|....|....|....|....| |  |
| ppa024457m |  | TATGCTTTGTTCACTTCACCGTCAGTTATGGCATGGGATGGTGATAAAGGGTACCAAAACCTGTTTGATGCAATGCTGGACGCTTTGTACTCTGCTGTTG |  |
| PRUAV004835 |  | TATGCTTTGTTCACTTCACCATCAGTTATGGCATGGGATGGTGATAAAGGGTACCAAAACCTGTTTGATGCAATGCTGGACGCTTTGTACTCGGCTGTTG |  |
| PavGH17-44_‘Regina’ |  | TATGCTTTGTTCACTTCACCATCAGTTATGGCATGGGATGGTGATAAAGGGTACCAAAACCTGTTTGATGCAATGCTGGATGCTTTGTACTCGGCTGTTG |  |
| PavGH17-44_‘Lapins’ |  | TATGCTTTGTTCACTTCACCATCAGTTATGGCATGGGATGGTGATAAAGGGTACCAAAACCTGTTTGATGCAATGCTGGATGCTTTGTACTCGGCTGTTG |  |
| PavGH17-44_‘Garnet’ |  | TATGCTTTGTTCACCTCACCATCAGTTATGGCATGGGATGGTGATAAAGGGTACCAAAACCTGTTTGATGCAATGCTGGACGCTTTGTACTCGGCTGTTG |  |
|  |  |  |  |
|  |  | ....|....|....|....|....|....|....|....|....|....|....|....|....|....|....|....|....|....|....|....| |  |
| ppa024457m |  | AGAGAGCTGGAGGAGGATCTTTGGAGGTTGTTGTGTCGGAAAGTGGGTGGCCTTCGGCAGGTGCGTTTGGGGCATCAACGGATAATGCGAGGACTTATTA |  |
| PRUAV004835 |  | AGAGAGCTGGAGGAGGCTCTTTGGAGGTTGTGGTGTCGGAAAGTGGGTGGCCTTCGGCAGGTGCGTTTGGGGCATCAACGGATAATGCGAGGAATTATTA |  |
| PavGH17-44_‘Regina’ |  | AGAGAGCTGGAGGAGGCTCTTTGGAGGTTGTGGTGTCGGAAAGTGGGTGGCCTTCGGCAGGTGCGTTTGGGGCATCAACGGATAATGCGAGGAATTATTA |  |
| PavGH17-44_‘Lapins’ |  | AGAGAGCTGGAGGAGGCTCTTTGGAGGTTGTGGTGTCGGAAAGTGGGTGGCCTTCGGCAGGTGCGTTTGGGGCATCAACGGATAATGCGAGGAATTATTA |  |
| PavGH17-44_‘Garnet’ |  | AGAGAGCTGGAGGAGGCTCTTTGGAGGTTGTGGTGTCGGAAAGTGGGTGGCCTTCGGCAGGTGCGTTTGGGGCATCAACGGATAATGCGAGGAATTATTA |  |
|  |  |  |  |
|  |  | ....|....|....|....|....|....|....|....|....|....|....|....|....|....|....|....|....|....|....|....| |  |
| ppa024457m |  | CTCGAATTTGATTCGGCATGTGAAA~GAGGGTACCCCAAAGAGACCTAAAAGATCCATAGAGACTTACTTGTTTGCCATGTTTGATGAGAATAACAAACT |  |
| PRUAV004835 |  | CTCGAATTTGATTTGGCATGTGAAA~GAGGGTACCCCAAAGAGACCTAAAAGATCCATAGAGACTTACTTGTTTGCCATGTTTGATGAGAATAACAAACT |  |
| PavGH17-44_‘Regina’ |  | CTCGAATTTGATTTGGCATGTGAAA~GAGGGTACCCCAAAGAGACCTAAAAGATCCATAGAGACTTACTTGTTTGCCATGTTTGATGAGAATAACAAACT |  |
| PavGH17-44_‘Lapins’ |  | CTCGAATTTGATTTGGCATGTGAAA~GAGGGTACCCCAAAGAGACCTAAAAGATCCATAGAGACTTACTTGTTTGCCATGTTTGATGAGAATAACAAACT |  |
| PavGH17-44_‘Garnet’ |  | CTCGAATTTGATTCGGCATGTGAAAAGAGGGTACCCCAAAGAGACCTAAAAGATCCATAGAGACTTACTTGTTTGCCATGTATGATGAGAATAACAAACT |  |
|  |  |  |  |
|  |  | ....|....|....|....|....|....|....|....|....|....|....|....|....|....|....|....|....|....|....|....| |  |
| ppa024457m |  | AGGTGAAGAAACAGAGAGACACTTTGGGGTGTTCTTCCCAACTAAAGAGCCAAAGTATAACCTCAATTTCGATACTTCCGCTGGGTATAATACTACAAAT |  |
| PRUAV004835 |  | GAGTGAAGAGACAGAGAGACACTTTGGGGTGTTCTTCCCAACTAAAGAGCCAAAGTATAACCTCAATTTCGGTACTTCGGCTGGGTATAATACTACAAAT |  |
| PavGH17-44_‘Regina’ |  | GAGTGAAGAGACAGAGAGACACTTTGGGGTGTTCTTCCCAACTAAAGAGCCAAAGTATAACCTCAATTTCGGTACTTCGGCTGGGTATAATACTACAAAT |  |
| PavGH17-44_‘Lapins’ |  | GGGTGAAGAGACAGAGAGACACTTTGGGGTGTTCTTCCCAACTAAAGAGCCAAAGTATAACCTCAATTTCGGTACTTCGGCTGGGTATAATACTACAAAT |  |
| PavGH17-44_‘Garnet’ |  | GGGTGAAGAGACAGAGAGACACTTTGGGGTGTTCTTCCCAACTAAAGAGCCAAAGTATAACCTCAATTTCGGTACTTCGGCTGGGTATAATACTACAAAT |  |
|  |  |  |  |
|  |  | ....|....|....|....|....|....|....|....|....|....|....|....|....|....|....|....|....|....|....|....| |  |
| ppa024457m |  | ACCCTTAACACTGACATGTAA |  |
| PRUAV004835 |  | ACCCTTAAAACTGACAAGTAATTACCGTTGGTTTCTTTCGATCCCCAAGTGCTGTTCTTAAAAATTAAAATAAAAGAAGTGAGCAGCAAACTCTTTATTT |  |
| PavGH17-44_‘Regina’ |  | ACCCTTAAAACTGACAAGTAATTACCGTTGGTTTCTTTCGATCCCCAAGTGCTGTTCTTAAAAATTAAAATAAAAGAAGTGAGCAGCAAACTCTTTATTT |  |
| PavGH17-44_‘Lapins’ |  | ACCCTTAAAACTGACAAGTAATTACCGTTGGTTTCTTTCGATCCCCAAGTGCTGTTCTTAAAAATTAAAATAAAAGAAGTGAGCAGCAAACTCTTTATTT |  |
| PavGH17-44_‘Garnet’ |  | ACCCTTAAAACTGACAGTAATTACCGTTGGTTTCTTTCGATCCCCAAGTGCTGTTCTTAAAATTAAAATAAAGAAGTGAGCAGCAAACTCTTTATTTATT |  |
|  |  |  |  |
|  |  | ....|....|....|....|....|....|....|....|....|....|....|....|....|....|....|....|....|....|....|....| |  |
| ppa024457m |  |  |  |
| PRUAV004835 |  | ATTTATTTTTATTTTATTTATATTTTTATCGCATGTGTAAGTGAGTTCTATTTCTTCTTTGTATGTAGAACATGTGGTCAGCAATACCGAAAGAAAATTC |  |
| PavGH17-44_‘Regina’ |  | ATTTATTTTTATTTTATTTATATTTTTATCGCATGTGTAAGTGAGTTCTATTTCTTCTTTGTATGTAGAACATGTGGTCAGCAATACCGAAAGAAAATTC |  |
| PavGH17-44_‘Lapins’ |  | ATTTATTTTTATTTTATTTATATTTTTATCGCATGTGTAAGTGAGTTCTATTTCTTCTTTGTATGTAGAACATGTGGTCAGCAATACCGAAAGAAAATTC |  |
| PavGH17-44_‘Garnet’ |  | TATTTTTATTTATTTATATTTTATCGCATGTGTAAGTGAGTTCTATTTCTtctttgtatgtagaacatgttgtcagccaataccgaaagaaaattccata |  |
|  |  |  |  |
|  |  | ....|....|....|....|....|....|....|....|....|....|....|....|....|....|....|....|....|....|....|....| |  |
| ppa024457m |  |  |  |
| PRUAV004835 |  | ATATCGATACTAGCAGCCCCTACAAGATCATCAATATCCACAAAGACCCAACCCAACTTACAAGATTATCAAAATTACAGAGAGACCCAAATCCAAATTA |  |
| PavGH17-44_‘Regina’ |  | ATATCGATACTAGCAGCCCCTACAAGATCATCAATATCCACAAGCCCCCCCCcccaaatcta |  |
| PavGH17-44_‘Lapins’ |  | ATATCGATACTAGCAGCCCCTACAAGATCATCAATATCCACAAGCCCACCCCccaacttaa |  |
| PavGH17-44_‘Garnet’ |  | ttcgatactagcagcccctacaagatcattcaattatcaccaaggacccaccccaactac |  |

***GH17-61***

|  |  | ....|....|....|....|....|....|....|....|....|....|....|....|....|....|....|....|....|....|....|....| |  |
| --- | --- | --- | --- |
| Ppa007079m | 1 | ~~~~~~~~~~~~~~~~~~~~~~~~~~~~~~~~~~~~~~~~~~~~~~~~~~~~~~~~~~~~~~~~~~~~TACGTAGTTCTGGGGATACAGGGGTAGTGGGG |  |
| PRUAV000448 |  | ATGCAAAGTGCACACTCCCATATTTTTATAAATGAAATCTTGGGCATTGTTAGGTGGCTAAAGTAGAGTACGTGGTTCTGGGGATACAGGGGTAGTGGGG |  |
| PavGH17-61_‘Regina’ |  | ~~~~~~~~~~~~~~~~~~~~~~~~~~~~~~~~~~~~~~~~~~~~~~~~~~~~~~~~~~~~~~~~~~~~~~~~~~~~~~~~~~~~~~~~~~~~~tcggggc |  |
| PavGH17-61_‘Lapins’ |  | ~~~~~~~~~~~~~~~~~~~~~~~~~~~~~~~~~~~~~~~~~~~~~~~~~~~~~~~~~~~~~~~~~~~~~~~~~~~~~~~~~~~~~~~~~~~~~atccggc |  |
| PavGH17-61_‘Garnet’ |  | ~~~~~~~~~~~~~~~~~~~~~~~~~~~~~~~~~~~~~~~~~~~~~~~~~~~~~~~~~~~~~~~~~~~~~~~~~~~~~~~~~~~~~~~~~~~~acaagggg |  |
|  |  |  |  |
|  |  | ....|....|....|....|....|....|....|....|....|....|....|....|....|....|....|....|....|....|....|....| |  |
| Ppa007079m |  | TG~~~~~~TTGGTGAAGGTGAAGGTGAAGGTGAAGGTGAAGGCCAAGATCGGATCAGTAATGGCAAGCTACACTGTCCTATTTAAAATTATTCTGGTCCT |  |
| PRUAV000448 |  | TGGTGGTGTTGGTGAAGTTGAAGGTGAAGGCCAAGGTGAAGGCCAAGATCGGATCAGTAATGGCAAGCTACACTGTCCTATTCAGAATTATTCTGGTCCT |  |
| PavGH17-61_‘Regina’ |  | agGTG~TGT~GGTGA~GTTGA~GGTGA~GGCCAAGGTGAAGGCCAAGATCGGATCAGTAATGGCAAGCTACACTGTCCTATTCAGAATTATTCTGGTCCT |  |
| PavGH17-61_‘Lapins’ |  | gGGTC~TGT~GGTGA~GTTGA~GGTGA~GGCCAAGGTGAAGGCCAAGATCGGATCAGTAATGGCAAGCTACACTGTCCTATTCAGAATTATTCTGGTCCT |  |
| PavGH17-61_‘Garnet’ |  | cgGTGGTGT~GGTGA~GTTGA~GGTGA~GGCCAAGGTGAAGGCCAAGATCGGATCAGTAATGGCAAGCTACACTGTCCTATTCAGAATTATTCTGGTCCT |  |
|  |  |  |  |
|  |  | ....|....|....|....|....|....|....|....|....|....|....|....|....|....|....|....|....|....|....|....| |  |
| Ppa007079m |  | TCTTAGTCTCTCAGGTTTGTTCTTTTTCAAACTACTTTCTTGCTTACCAGTTTGGTTCCGAAGAAAGTGCAGGAAAAGAAAAAGATGGTAATTTCATCTA |  |
| PRUAV000448 |  | TCTTAGTCTCTCAG~~~~~~~~~~~~~~~~~~~~~~~~~~~~~~~~~~~~~~~~~~~~~~~~~~~~~~~~~~~~~~~~~~~~~~~~~~~~~~~~~~~~~~ |  |
| PavGH17-61_‘Regina’ |  | TCTTAGTCTCTCAGGTTTGTTCTTTTTCAAACTATTTTCTTGCTTACCAGTTTGGTTCCCAAGAAAGTGCAGGAAAAGAAAAAGATGGTAATTTCAGCTA |  |
| PavGH17-61_‘Lapins’ |  | TCTTAGTCTCTCAGGTTTGTTCTTTTTCAAACTATTTTCTTGCTTACCAGTTTGGTTCCCAAGAAAGTGCAGGAAAAGAAAAAGATGGTAATTTCAGCTA |  |
| PavGH17-61_‘Garnet’ |  | TCTTAGTCTCTCAGGTTTGTTCTTTTTCAAACTATTTTCTTGCTTACCAGTTTGGTTCCCAAGAAAGTGCAGGAAAAGAAAAAGATGGTAATTTCAGCTA |  |
|  |  |  |  |
|  |  | ....|....|....|....|....|....|....|....|....|....|....|....|....|....|....|....|....|....|....|....| |  |
| Ppa007079m |  | AAAGCCTCAGATTTTCCTTCTGGGTCCTCTTAAACAAAATCTTCTCATCCGGGTCTTATATATCTCCTTTTTCGCTTTCTACCCACAAAAAGAAAAAAAA |  |
| PRUAV000448 |  | ~~~~~~~~~~~~~~~~~~~~~~~~~~~~~~~~~~~~~~~~~~~~~~~~~~~~~~~~~~~~~~~~~~~~~~~~~~~~~~~~~~~~~~~~~~~~~~~~~~~~ |  |
| PavGH17-61_‘Regina’ |  | AAAGCCTCAGATTTTCTTTCTGGGTCCTCTTAAACAAAATCTTCTCATCCGGGTCTTATATATCTCCTTTTTCGCTTTCTACCCACAAAAAGAACAAAAA |  |
| PavGH17-61_‘Lapins’ |  | AAAGCCTCAGATTTTCTTTCTGGGTCCTCTTAAACAAAATCTTCTCATCCGGGTCTTATATATCTCCTTTTTCGCTTTCTACCCACAAAAAGAACAAAAA |  |
| PavGH17-61_‘Garnet’ |  | AAAGCCTCAGATTTTCTTTCTGGGTCCTCTTAAACAAAATCTTCTCATCCGGGTCTTATATATCTCCTTTTTCGCTTTCTACCCACAAAAAGAACAAAAA |  |
|  |  |  |  |
|  |  | ....|....|....|....|....|....|....|....|....|....|....|....|....|....|....|....|....|....|....|....| |  |
| Ppa007079m |  | TATATATATCGAACACTTGAAATTTGAAACTCTGTTTTCTCTCCATCCAATTGAATTCAATCTGCTTTGCTATTTTCTGTTTTGAGATCAGTTTAGATTC |  |
| PRUAV000448 |  | ~~~~~~~~~~~~~~~~~~~~~~~~~~~~~~~~~~~~~~~~~~~~~~~~~~~~~~~~~~~~~~~~~~~~~~~~~~~~~~~~~~~~~~~~~~~~~~~~~~~~ |  |
| PavGH17-61_‘Regina’ |  | TATATATATCAGACACTTGAAATTTGAAACTCTGTTTTCTCTCCATCCAATTGAATTCAATCTTCTTTGCTATTTTCTGTTTTGAGATCAGTTCAGATTC |  |
| PavGH17-61_‘Lapins’ |  | TATATATATCAGACACTTGAAATTTGAAACTCTGTTTTCTCTYCATCCAATTGAATTCAATCTTCTTTGCTATTTTCTGTTTTGAGATCAGTTCAGATTC |  |
| PavGH17-61_‘Garnet’ |  | TATATATATCAGACACTTGAAATTTGAAACTCTGTTTTCTCTTCATCCAATTGAATTCAATCTTCTTTGCTATTTTCTGTTTTGAGATCAGTTCAGATTC |  |
|  |  |  |  |
|  |  | ....|....|....|....|....|....|....|....|....|....|....|....|....|....|....|....|....|....|....|....| |  |
| Ppa007079m |  | ATCAATCACTAATCTATATTGGGTTTTCATAAACTTAACAAGTATCTTCAATTTTACGTTAATTGCATTGGATTTAGGATCTGATTTTGCTCAAGATTTG |  |
| PRUAV000448 |  | ~~~~~~~~~~~~~~~~~~~~~~~~~~~~~~~~~~~~~~~~~~~~~~~~~~~~~~~~~~~~~~~~~~~~~~~~~~~~~~~~~~~~~~~~~~~~~~~~~~~~ |  |
| PavGH17-61_‘Regina’ |  | ATCAATCACTAATCTATACTGGGTTTTCATAAACTTAACAATTCTCTTCAATTTTACGTTAATTGCATTGGATTTAGGATCTGATTTTGCTCAAGATTTG |  |
| PavGH17-61_‘Lapins’ |  | ATCAATCACTAATCTATACTGGGTTTTCATAAACTTAACAATTCTCTTCAATTTTACGTTAATTGCATTGGATTTAGGATCTGATTTTGCTCAAGATTTG |  |
| PavGH17-61_‘Garnet’ |  | ATCAATCACTAATCTATACTGGGTTTTCATAAACTTAACAATTCTCTTCAATTTTACGTTAATTGCATTGGATTTAGGATCTGATTTTGCTCAAGATTTG |  |
|  |  |  |  |
|  |  | ....|....|....|....|....|....|....|....|....|....|....|....|....|....|....|....|....|....|....|....| |  |
| Ppa007079m |  | GTGCTGCAAACTTTACCAACTCTGTTCTCAACTCTGTTTCAGATTCAGCTGTTCATGTCCTCAGCCTGAGCTTCGGGATCAACTATGGACAAATAGCCAA |  |
| PRUAV000448 |  | ~~~~~~~~~~~~~~~~~~~~~~~~~~~~~~~~~~~~~~~~~~ATTCAGCTGTTCATGTCCTCAGCCTGAGCTTCGGGATCAACTATGGACAAATAGCCAA |  |
| PavGH17-61_‘Regina’ |  | GTGCGGCAAACTTTACCAACTCTGTTCTCAACTCTGTTTCAGATTCAGCTGTTCATGTCCTCAGCCTGAGCTTCGGGATCAACTATGGACAAATAGCCAA |  |
| PavGH17-61_‘Lapins’ |  | GTGCTGCAAACTTTACCAACTCTGTTCTCAACTCTGTTTCAGATTCAGCTGTTCATGTCCTCAGCCTGAGCTTCGGGATCAACTATGGACAAATAGCCAA |  |
| PavGH17-61_‘Garnet’ |  | GTGCGGCAAACTTTACCAACTCTGTTCTCAACTCTGTTTTAGATTCAGCTGTTCATGTCCTCAGCCTGAGCTTCGGGATCAACTATGGACAAATAGCCAA |  |
|  |  |  |  |
|  |  | ....|....|....|....|....|....|....|....|....|....|....|....|....|....|....|....|....|....|....|....| |  |
| Ppa007079m |  | CAACCTACCATCTCCCTCAAGAGTCTCCGTCCTTCTCCAAACTCTAAACGTCAGCAGAGTGAAACTCTACGACGCCGACCCGAACGTCCTCCAAGCATTC |  |
| PRUAV000448 |  | CAACCTACCATCTCCCTCAAGAGTCTCCGTCCTTCTCCAAACTCTAAACGTCAGCAGAGTGAAACTCTACGACGCCGACCCGAACGTCCTCCAAGCATTC |  |
| PavGH17-61_‘Regina’ |  | CAACCTACCATCTCCCTCAAGAGTCTCCGTCCTTCTCCAAACTCTAAACGTCAGCAGAGTGAAACTCTACGACGCCGACCCGAACGTCCTCCAAGCATTC |  |
| PavGH17-61_‘Lapins’ |  | CAACCTACCATCTCCCTCAAGAGTCTCCGTCCTTCTCCAAACTCTAAACGTCAGCAGAGTGAAACTCTACGACGCCGACCCGAACGTCCTCCAAGCATTC |  |
| PavGH17-61_‘Garnet’ |  | CAACCTACCATCTCCCTCAAGAGTCTCCGTCCTTCTCCAAACTCTAAACGTCAGCAGAGTGAAACTCTACGACGCCGACCCGAACGTCCTCCAAGCATTC |  |
|  |  |  |  |
|  |  | ....|....|....|....|....|....|....|....|....|....|....|....|....|....|....|....|....|....|....|....| |  |
| Ppa007079m |  | TCAAACTCACAAGTTGATTTCATCATAGGACTCGGCAACGAAAATTTGCAGAACATGAAGGATCCTCTCAAGGCCCAAGCTTGGATCCAGCAGCACGTCC |  |
| PRUAV000448 |  | TCAAACTCACAAGTTGATTTCATCATAGGACTCGGCAACGAAACTTTGCAGAACATGAAGGATCCTCTCAAGGCCCAAGCTTGGATCCAGCAGCACGTCC |  |
| PavGH17-61_‘Regina’ |  | TCAAACTCACAAGTTGATTTCATCATAGGACTCGGCAACGAAACTTTGCAGAACATGAAGGATCCTCTCAAGGCCCAAGCTTGGATCCAGCAGCACGTCC |  |
| PavGH17-61_‘Lapins’ |  | TCAAACTCACAAGTTGATTTCATCATAGGACTCGGCAACGAAACTTTGCAGAACATGAAGGATCCTCTCAAGGCCCAAGCTTGGATCCAGCAGCACGTCC |  |
| PavGH17-61_‘Garnet’ |  | TCAAACTCACAAGTTGATTTCATCATAGGACTCGGCAACGAAACTTTGCAGAACATGAAGGATCCTCTCAAGGCCCAAGCTTGGATCCAGCAGCACGTCC |  |
|  |  |  |  |
|  |  | ....|....|....|....|....|....|....|....|....|....|....|....|....|....|....|....|....|....|....|....| |  |
| Ppa007079m |  | AGCCTCACCTTCCCCAGACAAAAATCACCTGCATCACCGTGGGAAACGAAATCCTCGGCGGAAACGACACTCAGCTCATGTCGTATCTCCTCCCTGCAAT |  |
| PRUAV000448 |  | AGCCTCATCTTCCCCAGACAAAAATCACCTGCATCACCGTGGGAAACGAAATCCTCGGCGGAAGCGACGCTCAGCTCATGTCGTATCTCCTCCCTGCAAT |  |
| PavGH17-61_‘Regina’ |  | AGCCTCATCTTCCC~AGACAAAAATCACCTGCATCACCGTGGGAAACGAAATCCTCGGCGGAAGCGACGCTCAGCTCATGTCGTATCTCCTCCCTGCAAT |  |
| PavGH17-61_‘Lapins’ |  | AGCCTCATCTTCCCCAGACAAAAATCACCTGCATCACCGTGGGAAACGAAATCCTCGGCGGAAGCGACGCTCAGCTCATGTCGTATCTCCTCCCTGCAAT |  |
| PavGH17-61_‘Garnet’ |  | AGCCTCATCTTCCCCAGACAAAAATCACCTGCATCACCGTGGGAAACGAAATCCTCGGCGGAAGCGACGCTCAGCTCATGTCGTATCTCCTCCCTGCAAT |  |
|  |  |  |  |
|  |  | ....|....|....|....|....|....|....|....|....|....|....|....|....|....|....|....|....|....|....|....| |  |
| Ppa007079m |  | GCAATCTGTCTATAGAGCTCTTGTTGATCTTGGGCTCTCCAAGCAAGTTACTGTAACAACAGCACATTCTCTTACTATTTTGGGAAACTCCTACCCTCCT |  |
| PRUAV000448 |  | GCAATCTGTCTATGAAGCTCTTGTTGATCTTGGGCTCTCCAAGCAAGTTACTGTCACAACAGCACATTCTCTTACTATTTTGGGAGACTCCTACCCTCCT |  |
| PavGH17-61_‘Regina’ |  | GCAATCTGTCTATGAAGCTCTTGTTGATCTTGGGCTCTCCAAGCAAGTTACTGTCACAACAGCACATTCTCTTACTATTTTGGGAGACTCCTACCCTCCT |  |
| PavGH17-61_‘Lapins’ |  | GCAATCTGTCTATGAAGCTCTTGTTGATCTTGGGCTCTCCAAGCAAGTTACTGTCACAACAGCACATTCTCTTACTATTTTGGGAGACTCCTACCCTCCT |  |
| PavGH17-61_‘Garnet’ |  | GCAATCTGTCTATGAAGCTCTTGTTGATCTTGGGCTCTCCAAGCAAGTTACTGTCACAACAGCACATTCTCTTACTATTTTGGGAGACTCCTACCCTCCT |  |
|  |  |  |  |
|  |  | ....|....|....|....|....|....|....|....|....|....|....|....|....|....|....|....|....|....|....|....| |  |
| Ppa007079m |  | TCATCTGGGAGTTTTAAGCAAGATCTTGCTCAGTATATCCAGCCAATTCTCAGTTTCCATGC~ACAAGTTAATTCACCTTTTCTCATAAATGCATATCCA |  |
| PRUAV000448 |  | TCATCTGGGCGTTTTAGGCAAGATCTTGCTGAGTATATCCAGCCAATTCTCAGTTTTCTTGC~ACAAGTTAATTCACCTTTTCTCATAAATGCATATCCA |  |
| PavGH17-61_‘Regina’ |  | TCATCTGGGCGTTT~AGGCAAGATCTTGCTGAGTATATCCAGCCAATTCTCAGTTT~CTTGCcacaagttaattcaccttttctcataa~tgcatatcca |  |
| PavGH17-61_‘Lapins’ |  | TCATCTGGGCGTTTTAGGCA~GATCTtgctgagtatatccagccaat~ctcagttttcttgc~acaagttaattcaccttttctcataaatgcatatcca |  |
| PavGH17-61_‘Garnet’ |  | TCATCTGGGCGTTTTAGGCAAGATCTTGCTGAGTATATCCAGCCAATTCTCAGTTTTCTTGC~ACAAGTTAATTCACcttttctcataa~tgcatatcca |  |
|  |  |  |  |
|  |  | ....|....|....|....|....|....|....|....|....|....|....|....|....|....|....|....|....|....|....|....| |  |
| Ppa007079m |  | TATTTT~GCTTA~CAAGGACAACCCTGGAGAAGTTCCATTAGAGTATGTGCTTTTTCAGCCTAATTCTGGCATGGTTGATTCAGTTACCAATCT~GCACT |  |
| PRUAV000448 |  | TATTTT~GCTTA~CAAGGACAACCCTGGAGAAGTTCCATTAGAGTATGTGCTTTTTCAGCCTAATTCTGGCATGGTTGATTCAGTTACCAATCT~GCACT |  |
| PavGH17-61_‘Regina’ |  | tatttt~gcttaacaaggacaacc~tg~aga~gttc~attagagtatgtgctttttcagc~tagct~~~gcatg~~tgat~cagt~ac~a~tctggcact |  |
| PavGH17-61_‘Lapins’ |  | tatttt~gctta~ca~g~aca~ccctg~aga~gt~c~ataag~~tatgtgccttttcagc~ttattct~gcatg~atgat~cagt~ac~attctggcact |  |
| PavGH17-61_‘Garnet’ |  | tatttttgctta~ca~ggacaaccctggaga~gttccat~agagtatgtgctttt~cagcctagct~~~gcatg~~tgat~cagt~acca~tcttgcact |  |
|  |  |  |  |
|  |  | ....|....|....|....|....|....|....|....|....|....|....|....|....|....|....|....|....|....|....|....| |  |
| Ppa007079m |  | ATGA~CAACATGTTGGATGC~TC~AGA~TTGATGC~TGTTTATGC~TGCCATCAAGGCAATGGGGCATACCGATGT~CGAAGTGCGAATC~~~TCCGAGA |  |
| PRUAV000448 |  | ATGA~TAACATGTTGGATGC~TC~AGA~TTGATGC~TGTTTATGC~TGCCATCAAGGCAATGGGGCATACCGATGT~CGAAGTGCGAATC~~~TCCGAGA |  |
| PavGH17-61_‘Regina’ |  | atga~ta~catgt~gaatgcctc~agaat~gatgcctgtt~a~gcctgc~atca~gcca~tgag~catac~gatgttcgaaatgcgaatcct~tccgaga |  |
| PavGH17-61_‘Lapins’ |  | atga~taacatgt~g~atgc~tcgagaattgatgc~tgt~~a~gc~tgccatcgagc~aatgggccatac~gatgttcgaagtgcgaatcctctccgaaa |  |
| PavGH17-61_‘Garnet’ |  | atgaataacatgttg~atgcctc~agaat~gatgc~tgtt~atgc~tgc~atca~ggcaatgcgc~~tac~gatgt~cga~c~gccaatc~tctc~gaaa |  |
|  |  |  |  |
|  |  | ....|....|....|....|....|....|....|....|....|....|....|....|....|....|....|....|....|....|....|....| |  |
| Ppa007079m |  | C~~GGGT~TGG~CCTTCGAAGGGGGATCCGAATGAGGCCGGAGCCACGCCGGAGAATGCAGGGTTGTACAATGGGAATTTGATGAGGAAACTTGAAGAGA |  |
| PRUAV000448 |  | C~~GGGT~TGG~CCTTCGAAGGGGGATCCGAATGAGGCCGGAGCCACGCCGGAGAATGCAGGGTTGTACAATGGGAATTTGATGAGGAAACTTGAAGAGA |  |
| PavGH17-61_‘Regina’ |  | cacgg~t |  |
| PavGH17-61_‘Lapins’ |  | cactg |  |
| PavGH17-61_‘Garnet’ |  | cacgggtgtgggc |  |
|  |  |  |  |
|  |  | ....|....|....|....|....|....|....|....|....|....|....|....|....|....|....|....|....|....|....|....| |  |
| Ppa007079m |  | GGAAAGGAACTCCGGCTAAGCCTTCTGTTCCAATAGACATTTATGTTTTTGCACTTTTTAATGAGGATTTGAAGCCTGGCCCTGCATCAGAAAGGAATTA |  |
| PRUAV000448 |  | GGAAAGGAACTCCGGCTAAGCCTTCTGTTCCAATAGAGATGTATGTTTTTGCACTTTTTAATGAGGATTTGAAGCCTGGCCCTGCATCAGAGAGGAATTA |  |
| PavGH17-61_‘Regina’ |  |  |  |
| PavGH17-61_‘Lapins’ |  |  |  |
| PavGH17-61_‘Garnet’ |  |  |  |
|  |  |  |  |
|  |  | ....|....|....|....|....|....|....|....|....|....|....|....|....|....|....|....|....|....|....|....| |  |
| Ppa007079m |  | TGGGCTCTATTATCCTGATGGCACACCAGTTTATGATATTGGCTTCAAGGGTTATCTCCCTGAGCTAACCTTTACTGCCGACTCAAATAAAAATAATGTA |  |
| PRUAV000448 |  | TGGGCTCTATTATCCTGATGGCACACCAGTTTATGATATTGGCTTCAAGGGTTATCTCCCTCAGCTAACCTTTTCTGCCGACTCAAATAAAAATAATGT~ |  |
| PavGH17-61_‘Regina’ |  |  |  |
| PavGH17-61_‘Lapins’ |  |  |  |
| PavGH17-61_‘Garnet’ |  |  |  |
|  |  |  |  |
|  |  | ....|....|....|....|....|....|....|....|....|....|....|....|....|....|....|....|....|....|....|....| |  |
| Ppa007079m |  | AGTAAAAATAAAATATACCTTTTTGTTCATTTGTCTGTTTTATGATCTTGGGTTAGAGTTAATTTTGTGGTTTAATATCTAATGCTGTTCTGTTTTCTTG |  |
| PRUAV000448 |  | ~~~~~~~~~~~~~~~~~~~~~~~~~~~~~~~~~~~~~~~~~~~~~~~~~~~~~~~~~~~~~~~~~~~~~~~~~~~~~~~~~~~~~~~~~~~~~~~~~~~~ |  |
| PavGH17-61_‘Regina’ |  |  |  |
| PavGH17-61_‘Lapins’ |  |  |  |
| PavGH17-61_‘Garnet’ |  |  |  |
|  |  |  |  |
|  |  | ....|....|....|....|....|....|....|....|....|....|....|....|....|....|....|....|....|....|....|....| |  |
| Ppa007079m |  | CATGTTTTGCAGGTCTTGTCCATCTTCAACTTTCTGATCTTTCTTACTGTGTACTTGTTATTATCTGATTAATGCTCCTAAATTTTGGGCTCGATCCTAA |  |
| PRUAV000448 |  | ~~~~~~~~~~~~~~CCTCTCCATCTTCAACTTTCTGCTCTTTCTTATTGTGTACTTATTATTATCTGCTTAATGCTCCCAAATTTTGGGCTCGATCCTAA |  |
| PavGH17-61_‘Regina’ |  |  |  |
| PavGH17-61_‘Lapins’ |  |  |  |
| PavGH17-61_‘Garnet’ |  |  |  |
|  |  |  |  |
|  |  | ....|....|....|....|....|....|....|....|....|....|....|....|....|....|....|....|....|....|....|....| |  |
| Ppa007079m |  | ATCAATTGGTCAAAGAAGTGGTTCGCATTAAGTTCGTTCAAATGTTTAGGGACTTGGGAGCCAACATATGCATGAAGAATTCCTCAAGATTTTTCCAGGG |  |
| PRUAV000448 |  | ATCAATTGGT~~~~~~~~~~~~TCGCATTAAGTTCGTTCAAATGTCTGGGGACTTGGGAGCCAACATATGCATGAAGAATTCCTCAATATTTTTCCAGGG |  |
| PavGH17-61_‘Regina’ |  |  |  |
| PavGH17-61_‘Lapins’ |  |  |  |
| PavGH17-61_‘Garnet’ |  |  |  |

***GH17-101***

|  |  | ....|....|....|....|....|....|....|....|....|....|....|....|....|....|....|....|....|....|....|....| |  |
| --- | --- | --- | --- |
| ppa025430m | 111 | TCTTTCTCATCTCTATAAATTAAGGAGCAAGAGGCAGCCTTGAGAGACGTACACTTAGAATATTTCTTAGAAAATTGTATGTTTC~~~~TGACCATGACT |  |
| PRUAV000060 |  | ~~~~~~~~~~~~~~~~~~~~~~~~~~~~~~~~~~~~~~~~~~~~~GAGGCACACTTGCAATACTTCTTAGAATATCTATCTTTTCCTTATTATCATGGCT |  |
| PavGH17-101_‘Regina’ |  | ~~~~~~~~~~~~~~~~~~~~~~~~~~~~~~~~~~~gctggaggtcagtcgaacaccggtatttcgacatgcccacaccaaactcgagcatgcattctaga |  |
| PavGH17-101_‘Lapins’ |  | ~~~~~~~~~~~~~~~~~~~~~~~~~~~~~~~~~~~gctgaggtcagtcgacacacggtattcgagcgaatgccagtcacaaacttcaggacatgcatctt |  |
| PavGH17-101_‘Garnet’ |  | ~~~~~~~~~~~~~~~~~~~~~~~~~~~~~~~~~~~~~~~~~~~~~~~~~~~~~~~~~~~~~~~~~~~~~~aatgaccacaacaaacttcaggcatgcatt |  |
|  |  |  |  |
|  |  | ....|....|....|....|....|....|....|....|....|....|....|....|....|....|....|....|....|....|....|....| |  |
| ppa025430m |  | AAATCGAATTCGTCATCAGTTGGCAGATGCCTTTCTCTGATTTCTATAGTACTTCTACTTGGGCAGCTGGTGGTGGCTAGCTTGGCAACAAAACAACACA |  |
| PRUAV000060 |  | AAATCCAATT~~~CATCAGTTGGTA~~~~~~~~~~~~~~~TTTCGATAGTATTTCTACTTGGGCTGCTGATG~~~GCTAGCTTCGAAACAACAGGA~~~~ |  |
| PavGH17-101_‘Regina’ |  | taccgcaatatgtgagacagttagctaagggggtatatggttcgtctcgtctgagaatcgcgagagtttcccaatagagttgtgttaagggcagaggtag |  |
| PavGH17-101_‘Lapins’ |  | agatgcggccaatttgaacagttagccttaagtggtaatgtgttgcgtctggtatagattcgagagtctcccaatagagattggttaa~ggcagaggtag |  |
| PavGH17-101_‘Garnet’ |  | ctgtacggccaattgataccgttagccttaaggtgtaattggttcgtctcgctttagattcgagcctttcccatataggattgtttaaaggctatagtag |  |
|  |  |  |  |
|  |  | ....|....|....|....|....|....|....|....|....|....|....|....|....|....|....|....|....|....|....|....| |  |
| ppa025430m |  | CAGGTATGCATATATAATTGGCACTGCTGTCTGTCAATCTAACTGCCAATTTAGCTTTTGTTTTTCTAGCGTATGATTCATCTAACTCGTGATTAACGAA |  |
| PRUAV000060 |  | ~~~~~~~~~~~~~~~~~~~~~~~~~~~~~~~~~~~~~~~~~~~~~~~~~~~~~~~~~~~~~~~~~~~~~~~~~~~~~~~~~~~~~~~~~~~~~~~~~~~~ |  |
| PavGH17-101_‘Regina’ |  | aaga~ttatatctcagcctatcct~tgagatatatatgtagtgaaatacatatttgtcatatgtatcatacatgcgatcaatgggtgagattagaagTCT |  |
| PavGH17-101_‘Lapins’ |  | aagaattatatctcagc~tatc~tatgagatatatatgtagtgaaatacatatttgtcatatgtatcatacatgcgatcaatgggtgagattagaaGTCT |  |
| PavGH17-101_‘Garnet’ |  | aagatttatatctcagcatatcctttgagatatatatgtagtgaaatacatatttgtcatatgtatcatacatgcgatcaatgggtgagat~agaagtct |  |
|  |  |  |  |
|  |  | ....|....|....|....|....|....|....|....|....|....|....|....|....|....|....|....|....|....|....|....| |  |
| ppa025430m |  | AGCCAATGCACAACAAATAACGACATTTTTGTGTGTGTCTATTTTTAAAACATAAGGACCAAACTGTTCCATGCAACAAACCACGCATTCCTAATCAATC |  |
| PRUAV000060 |  | ~~~~~~~~~~~~~~~~~~~~~~~~~~~~~~~~~~~~~~~~~~~~~~~~~~~~~~~~~~~~~~~~~~~~~~~~~~~~~~~~~~~~~~~~~~~~~~~~~~~~ |  |
| PavGH17-101_‘Regina’ |  | AACTATGCTTAACATAAAATCTT~AATTAATGGAGATAAACCCAAAGAACTTTACATTGGCTGAAACAAATTCATACATTTTTTGAGCTCCTGTTTAAAA |  |
| PavGH17-101_‘Lapins’ |  | AACTATGCTTAACATAAAATCTTAAATTAATGGAGAGAAACCCAAAGAACTTTACATTGGCTGAAACAA~TTCATACATTTTTTGAGCTCCTGTTTAAAA |  |
| PavGH17-101_‘Garnet’ |  | aactatgcttaacataaaatctttAATTAATGGAGAGAAACCCAAAGAACTTTACATTGGCTGAAACAA~TTCATACATTTTTTGAGCTCCTGTTTAAAA |  |
|  |  |  |  |
|  |  | ....|....|....|....|....|....|....|....|....|....|....|....|....|....|....|....|....|....|....|....| |  |
| ppa025430m |  | TCACATAAGGACCAAATTGGTGTGTCTATTTAGCAATATAAATTCCTTTTTCAATGTAGGTGCTCCAGTTGGTGTATGTAATGGAATGGTTGGCGATGAC |  |
| PRUAV000060 |  | ~~~~~~~~~~~~~~~~~~~~~~~~~~~~~~~~~~~~~~~~~~~~~~~~~~~~~~~~~~~~~GCCCAAATTGGTGTATGTTATGGAATGCTTGGAGATCGT |  |
| PavGH17-101_‘Regina’ |  | TGATCCACATAGAAAATCGATCATAAAACCTCATTATAATTTCTTCTTCAATATTGCAGGAGCCCAAATTGGTGTATGTTATGGAATGCTTGGAGATCGT |  |
| PavGH17-101_‘Lapins’ |  | TGATCCACATAGAAAATCGATCATAAAACCTCATTATAATTTCTTCTTCAATATTGCAGGAGCCCAAATTGGTGTATGTTATGGAATGCTTGGAGATCGT |  |
| PavGH17-101_‘Garnet’ |  | TGATCCACATAGAAAATCGATCATAAAACCTCATTATAATTTCTTCTTCAATATTGCAGGAGCCCAAATTGGTGTATGTTATGGAATGCTTGGAGATCGT |  |
|  |  |  |  |
|  |  | ....|....|....|....|....|....|....|....|....|....|....|....|....|....|....|....|....|....|....|....| |  |
| ppa025430m |  | CTACCACCCCAAGCGGAAGTTGTTGCCCTCTACAAGACAAATAACATCCCAAGAATGCGACTTTATGATCCAAACCCAGCCGCTCTAGAAGCCCTTCGAG |  |
| PRUAV000060 |  | TTACCACCCCCATCAGAAGTCATTGCTCTGTACAAGCAAAATAACATCGGAAGAATGCGACTGTATGATCCAAACCAGGCTGCTCTAGCAGCCCTTAGAG |  |
| PavGH17-101_‘Regina’ |  | TTACCACCCCCATCAGAAGTCATTGCTCTGTACAAGCAAAATAACATCGGAAGAATGCGACTGTATGATCCAAACCAGGCTGCTCTAGCAGCCCTTAGAG |  |
| PavGH17-101_‘Lapins’ |  | TTACCACCCCCATCAGAAGTCATTGCTCTGTACAAGCAAAATAACATCGGAAGAATGCGACTGTATGATCCAAACCAGGCTGCTCTAGCAGCCCTTAGAG |  |
| PavGH17-101_‘Garnet’ |  | TTACCACCCCCATCAGAAGTCATTGCTCTGTACAAGCAAAATAACATCGGAAGAATGCGACTGTATGATCCAAACCAGGCTGCTCTAGCAGCCCTTAGAG |  |
|  |  |  |  |
|  |  | ....|....|....|....|....|....|....|....|....|....|....|....|....|....|....|....|....|....|....|....| |  |
| ppa025430m |  | GCTCCAATATCAAGCTCTTGCTAGGCGTACCAAATGAAAACCTTCAATACATTGCCTTAAGCCAAGCCAACGCAAATGCATGGGTCCAAAACAATGTGAG |  |
| PRUAV000060 |  | GCTCTAATATTGAGCTCATGCTAGGCGTTCCAAATGACAACCTTCAAAGCCTTGCCTCAAGCCAAGCCAATGCAAATACTTGGGTCCAAAATAATGTGAG |  |
| PavGH17-101_‘Regina’ |  | GCTCTAATATTGAGCTCATGCTAGGCGTTCCAAATGACAACCTTCAAAGCCTTGCCTCAAGCCAAGCCAATGCAAATACTTGGGTCCAAAATAATGTGAG |  |
| PavGH17-101_‘Lapins’ |  | GCTCTAATATTGAGCTCATGCTAGGCGTTCCAAATGACAACCTTCAAAGCCTTGCCTCAAGCCAAGCCAATGCAAATACTTGGGTCCAAAATAATGTGAG |  |
| PavGH17-101_‘Garnet’ |  | GCTCTAATATTGAGCTCATGCTAGGCGTTCCAAATGACAACCTTCAAAGCCTTGCCTCAAGCCAAGCCAATGCAAATACTTGGGTCCAAAATAATGTGAG |  |
|  |  |  |  |
|  |  | ....|....|....|....|....|....|....|....|....|....|....|....|....|....|....|....|....|....|....|....| |  |
| ppa025430m |  | AAACTATGCCAATGTGAAATTCAAGTACATTGCGGTAGGAAATGAAGTCAAGCCTTCAGACTCCTTTGCACAGTTTCTCGTCCCAGCCATGCGAAATATT |  |
| PRUAV000060 |  | AAACTATGGCAATGTAAAATTCAAATACATTGCGGTAGGAAATGAAGTCAAGCCCTCAGACTCCTTTGCACAATTTCTTGTTCCAGCCATGCAAAACATT |  |
| PavGH17-101_‘Regina’ |  | AAACTATGGCAATGTAAGATTCAAATACATTGCGGTAGGAAATGAAGTCAAGCCCTCAGACTCCTTTGCACAATTTCTTGTTCCAGCCATGCAAAACATT |  |
| PavGH17-101_‘Lapins’ |  | AAACTATGGCAATGTAAAATTCAAATACATTGCGGTAGGAAATGAAGTCAAGCCCTCAGACTCCTTTGCACAATTTCTTGTTCCAGCCATGCAAAACATT |  |
| PavGH17-101_‘Garnet’ |  | AAACTATGGCAATGTAAAATTCAAATACATTGCGGTAGGAAATGAAGTCAAGCCCTCAGACTCCTTTGCACAATTTCTTGTTCCAGCCATGCAAAACATT |  |
|  |  |  |  |
|  |  | ....|....|....|....|....|....|....|....|....|....|....|....|....|....|....|....|....|....|....|....| |  |
| ppa025430m |  | CAAGAGGCAATTTCTCTTGCTGGTCTTGCAAAGAAAATTAAAGTTTCGACAGCCATCGACACCGGAGTACTTGGAGAGACCTTTCCTCCTTCGATAGGCT |  |
| PRUAV000060 |  | CAGAACGCAATTTCCAGTGCTGGTTT~~~~~~GGGAATTAAAGTCTCCACTGCCATAGACACTGGAGTGCTTGGAAACTCCTTTCCTCCATCAAACGGAG |  |
| PavGH17-101_‘Regina’ |  | CAGAACGCAATTTCTAGTGCTGGTTT~~~~~~GGGAATTAAAGTTTCCACTGCCATAGACACTGGAGTGCTTGGAAAGTCCTTTCCTCCATCAAACGGAG |  |
| PavGH17-101_‘Lapins’ |  | CAGAACGCAATTTCTAGTGCTGGTTT~~~~~~GGGAATTAAAGTTTCCACTGCCATAGACACTGGAGTGCTTGGAAAGTCCTTTCCTCCATCGAACGGAG |  |
| PavGH17-101_‘Garnet’ |  | CAGAACGCAATTTCTAGTGCTGGTTT~~~~~~GGGAATTAAAGTTTCCACTGCCATAGACACTGGAGTGCTTGGAAACTCCTTTCCTCCATCAAACGGAG |  |
|  |  |  |  |
|  |  | ....|....|....|....|....|....|....|....|....|....|....|....|....|....|....|....|....|....|....|....| |  |
| ppa025430m |  | CATTCAAGTCTGAATATAACGCCCTTTTATATCCCATCATCCGCTTCCTAGTGAGCCACCAATCGCCATTGCTTGTTAACTTGTACCCTTATTTTGCTTA |  |
| PRUAV000060 |  | AGTTCAAGTCCGAATATGGAGCACTTTTGAACCCCATCATCCGCTTCCTAGTGAACAACAGATCGCCGTTGCTGGTTAATTTGTACCCTTATTTCAGCTA |  |
| PavGH17-101_‘Regina’ |  | AGTTCAAGTCCGAATATGGAGCACTTTTGAACCCCATCATCCGCTTCCTAGTGAACAACAGATCGCCGTTGCTGGTTAATTTGTACCCTTATTTCAGCTA |  |
| PavGH17-101_‘Lapins’ |  | AGTTCAAGTCCGAATATGGAGCACTTTTGAACCCCATCATCCGCTTCCTAGTGAACAACAGATCGCCGTTGCTGGTTAATTTGTACCCTTATTTCAGCTA |  |
| PavGH17-101_‘Garnet’ |  | AGTTCAAGTCCGAATATGGAGCACTTTTGAACCCAATCATCCGCTTCCTAGTGAACAACAGATCGCCGTTGCTGGTTAATTTGTACCCTTATTTCAGCTA |  |
|  |  |  |  |
|  |  | ....|....|....|....|....|....|....|....|....|....|....|....|....|....|....|....|....|....|....|....| |  |
| ppa025430m |  | CAGTGGCAACACTCAAGACATTCGTCTTGACTATGCTCTTTTCACAGCTCCATCAGTTGTGGTACAAGATGGGAACTTTGGTTACCGAAATCTTTTCGAT |  |
| PRUAV000060 |  | CAGCGGCAACACTCGTGACATTCGTCTAGATTATGCTCTTTTTACAGCTCCATCAGTTGTGGTACAAGATGGCCAACGTGGCTATCGTAATCTTTTCGAT |  |
| PavGH17-101_‘Regina’ |  | CAGCGGCAACACTCGTGACATTCGTCTAGATTATGCTCTTTTCACAGCTCCATCAGTTGTGGTACAAGATGGCCAACGTGGCTATCGTAATCTTTTCGAT |  |
| PavGH17-101_‘Lapins’ |  | CAGCGGCAACACTCGTGACATTCGTCTAGATTATGCTCTTTTCACAGCTCCATCAGTTGTGGTACAAGATGGCCAACGTGGCTATCGTAATCTTTTCGAT |  |
| PavGH17-101_‘Garnet’ |  | CAGCGGCAACACTCGTGACATTCGTCTAGATTATGCTCTTTTCACAGCTCCATCAGTTGTGGTACAAGATGGCCAACGTGGCTATCGTAATCTTTTCGAT |  |
|  |  |  |  |
|  |  | ....|....|....|....|....|....|....|....|....|....|....|....|....|....|....|....|....|....|....|....| |  |
| ppa025430m |  | GCCATGTTAGATGGTGTTTATGCTGCTCTTGAGAAGGCTGGTGGAGGGTCTTTGAAAGTTGTTATATCAGAGACTGGTTGGCCATCAGCTGCTGGAACAG |  |
| PRUAV000060 |  | GCCATTTTGGATGCTGTTTATGCCGCTCTTGAGAAGGCGGGCGGAGGGTCTTTGGAAATTGTCATATCCGAAAGTGGTTGGCCATCAGCTGGTGGAACGG |  |
| PavGH17-101_‘Regina’ |  | GCCATTTTGGAYGCTGTTTATGCCGCTCTTGAGAAGGCGGGCGGAGGGTCTTTGGAAATTGTCATATCCGAAAGTGGTTGGCCATCAGCTGGTGGAACGG |  |
| PavGH17-101_‘Lapins’ |  | GCCATTTTGGAYGCTGTTTATGCCGCTCTTGAGAAGGCGGGCGGAGGGTCTTTGGAAATTGTCATATCCGAAAGTGGTTGGCCATCAGCTGGTGGAACGG |  |
| PavGH17-101_‘Garnet’ |  | GCCATTTTGGAYGCTGTTTATGCCGCTCTTGAGAAGGCGGGCGGAGGGTCTTTGGAAATTGTCATATCCGAAAGTGGTTGGCCATCAGCTGGTGGAACGG |  |
|  |  |  |  |
|  |  | ....|....|....|....|....|....|....|....|....|....|....|....|....|....|....|....|....|....|....|....| |  |
| ppa025430m |  | CCACAACAATTGATAATGCAAGGACTTTTATATCAAATTTGATTCAACATGTGAAGGAAGGGACTCCAAGGAGGCCAGGAAGGCCCATAGAAACTTACAT |  |
| PRUAV000060 |  | CAACAACGATTGATAATGCAAGGACTTATAACGCAAATCTGATTCAACATGTGAAGGGAGGGACTCCAAGGAAGCCCGGAAGGGCCATAGAAACCTACAT |  |
| PavGH17-101_‘Regina’ |  | CAACAACGATTGATAATGCAAGGACTTATAACGCAAATCTGATTCAACATG~~GAAgg~cgtccgcc |  |
| PavGH17-101_‘Lapins’ |  | CAACAACGATTGATAATGCAAGGACTTATAACGCAAATCTGATTCAACATG~~GAAGGaggtgaatt |  |
| PavGH17-101_‘Garnet’ |  | CAACAACGATTGATAATGCAAGGACTTATAACGCAAATCTGATTCAACATG~~GAAGGGAgactatt |  |

***GI***

|  |  | 10 20 30 40 50 60 70 80 90 100 |  |
| --- | --- | --- | --- |
|  |  | ....|....|....|....|....|....|....|....|....|....|....|....|....|....|....|....|....|....|....|....| |  |
| Ppa000556m | 7951 | CTAAAAAGACAAAAAAACAGGGGCCGGTAGCAGCATTTGATTCTTATGTTCTGGCTGCTGTTTGTGCTCTTGCATGTGAGCTTCAGTTGTTCCCTTTGAT |  |
| PRUAV000817 |  | CTAAAAAGACAAAAAAACAGGGGCCTGTAGCAGCATTTGATTCTTATGTTCTGGCTGCTGTTTGTGCTCTTGCATGTGAGCTTCAGTTGTTCCCTTTGAT |  |
| PavGI_‘Regina’ |  | ~~~~~~~~~~~~~~~~~~~~~~~~~~~~agcttgcT~~~~~~~~~~~~~~~~~~CTGC~~~~TGTGCTCTTGCATGTGAGCTTCAGTTGTTCCCTTTGAT |  |
| PavGI_‘Lapins’ |  | ~~~~~~~~~~~~~~~~~~~~~~~~~~~~agtgtg~~~~~~~~~~~~~gT~~~~GCTGA~~~~TGTGCTCTTGCATGTGAGCTTCAGTTGTTCCCTTTGAT |  |
| PavGI_‘Garnet’ |  | ~~~~~~~~~~~~~~~~~~~~~~~~~~~~aagcGC~~~~~~~~~~~~~~~~~T~~CT~~~~~~TGTGCTCTTGCATGTGAGCTTCAGTTGTTCCCTTTGAT |  |
|  |  |  |  |
|  |  | ....|....|....|....|....|....|....|....|....|....|....|....|....|....|....|....|....|....|....|....| |  |
| Ppa000556m |  | TTCAAAGGGGATTAATCATGCTCACTCTAAAGATGCAAAAAATGTGGCAAAGCCTGCCAAAGAAAATGTATGTACTAATGAGTTTCGGAGTAGTGTTGA |  |
| PRUAV000817 |  | TTCGAAGGGGATTAATCGTGCTCACTCTAAAGATGCAAAAAATGTGGCAAAGCCTGCCAAAGAAAATGTATGTACTAATGAGTTTCGGAGTAGTGTTGA |  |
| PavGI_‘Regina’ |  | TTCGAAGGGGATTAATCGTGCTCACTCTAAAGATGCAAAAAATGTGGCAAAGCCTGCCAAAGAAAATGTATGTACTAATGAGTTTCGGAGTAGTGTTGA |  |
| PavGI_‘Lapins’ |  | TTCGAAGGGGATTAATCRTGCTCACTCTAAAGATGCAAAAAATGTGGCAAAGCCTGCCAAAGAAAATGTATGTACTAATGAGTTTCGGAGTAGTGTTGA |  |
| PavGI_‘Garnet’ |  | TTCGAAGGGGATTAATCRTGCTCACTCTAAAGATGCAAAAAATGTGGCAAAGCCTGCCAAAGAAAATGTATGTACTAATGAGTTTCGGAGTAGTGTTGA |  |
|  |  |  |  |
|  |  |  |  |
| Ppa000556m |  | CTCAGCAGTTTGTCACACACGCAGAATACTAGCCATTTTGGAGGCACTTTTCTTGCTGAAGCCATCTTCTGTTGGCACTTCGTGGAGTTACAGTTCAAAT |  |
| PRUAV000817 |  | CTCAGCAGTTTGTCACACACGCAGAATATTAGCCATTTTGGAGGCACTTTTCTTGCTGAAGCCATCTTCCATTGGCACTTCGTGGAGTTACAGTTCAAAT |  |
| PavGI_‘Regina’ |  | CTCAGCAGTTTGTCACACACGCAGAATATTAGCCATTTTGGAGGCACTTTTCTTGCTGAAGCCA |  |
| PavGI_‘Lapins’ |  | CTCAGCAGTTTGTCACACACGCAGAATATTAGCCATTTTGGAGGCACTTTTTTTGCTGAAGCCA |  |
| PavGI_‘Garnet’ |  | CTCAGCAGTTTGTCACACACGCAGAATATTAGCCATTTTGGAGGCACTTTTCTTGCTGAAGCCA |  |

***HYL1***

|  |  | ....|....|....|....|....|....|....|....|....|....|....|....|....|....|....|....|....|....|....|....| |  |
| --- | --- | --- | --- |
| ppa004305m | 1 | TTCTCATGGGTTTCTAGTTTTGAAAAGAAAAGAAAAAG~CCTTCAAAAATTTTAACCTTCTCTCTCTACCTATCAGACAAATCCAGGTACTCCGGCCAGA |  |
| PRUAV015329 |  | ~~~~~ATGGGTTTCTAGTTTTGAAAAGAAAAGAAAAAGACCTTAAAAAAATTTAACCTTCTCTCTCTACCTATCAGACAAATCCAGGTACTCCGGCCAGA |  |
| PavHYL1_‘Regina’ |  | ~~~~~~~~~~~~~~~~~~~~~~~~~~~~~~~~~~~~~~~~~~~~~~~~~~~~~~~~~~~~~~~~~~~~~tattatccaaaaaaaacctggtcctcccagc |  |
| PavHYL1_‘Lapins’ |  | ~~~~~~~~~~~~~~~~~~~~~~~~~~~~~~~~~~~~~~~~~~~~~~~~~~~~~~~~~~~~~~~~~~~~~~tTATCAGACCCAAAACCAGGTACTCCGGCC |  |
| PavHYL1_‘Garnet’ |  | ~~~~~~~~~~~~~~~~~~~~~~~~~~~~~~~~~~~~~~~~~~~~~~~~~~~~~~~~~~~~~~~~~~~~~tttttttcaaaaataacctggtcctcccagc |  |
|  |  |  |  |
|  |  | ....|....|....|....|....|....|....|....|....|....|....|....|....|....|....|....|....|....|....|....| |  |
| ppa004305m |  | AGACAAAACAGACAACCTCCAGGTATACTCTCTCCTTCTTTTCTCGCTCTCTGCTAGGGTTTAAATTTGAGATTCAATCAATTTCACTATATATGTCTCT |  |
| PRUAV015329 |  | AGACAAAACAGACAACATCCAG~~~~~~~~~~~~~~~~~~~~~~~~~~~~~~~~~~~~~~~~~~~~~~~~~~~~~~~~~~~~~~~~~~~~~~~~~~~~~~ |  |
| PavHYL1_‘Regina’ |  | agacgacacagccgacaacctccTGGTATTTTTTCTCCTTTTTTCGCTCTCTGCTAGGGTTTAAATTTGAGATTCAATCAATTTCACTATATATGTCTCT |  |
| PavHYL1_‘Lapins’ |  | AGAAGACAAAACAGACAACATCCAGGTACTCTCTCTCCTTTTTTCGCTCTCTGCTAGGGTTTAAATTTGAGATTCAATCAATTTCACTATATATGTCTCT |  |
| PavHYL1_‘Garnet’ |  | agacgacacagccgacatcctcctgGTCTTTTTTCTCCTTTTTTCGCTCTCTGCTAGGGTTTAAATTTGAGATTCAATCAATTTCACTATATATGTCTCT |  |
|  |  |  |  |
|  |  | ....|....|....|....|....|....|....|....|....|....|....|....|....|....|....|....|....|....|....|....| |  |
| ppa004305m |  | TCCTGGTTTTATTTCCTAATTTTCATGAACTGTTCGCTCCCCCGTTCTAAACCCAGTTTACACTTTATTGTTGTAATGAAAAAGTTTTTCATTTACAATA |  |
| PRUAV015329 |  | ~~~~~~~~~~~~~~~~~~~~~~~~~~~~~~~~~~~~~~~~~~~~~~~~~~~~~~~~~~~~~~~~~~~~~~~~~~~~~~~~~~~~~~~~~~~~~~~~~~~~ |  |
| PavHYL1_‘Regina’ |  | TCCTGGTTTTATTTCCTAATTTTCATGAACTGTTCGCTCCCCCGTTCTAAATCCAGTTTACACTTTATTGTTGTAATGAAAAGGCTTTTCATTTACAATA |  |
| PavHYL1_‘Lapins’ |  | TCCTGGTTTTATTTCCTAATTTTCATGAACTGTTCGCTCCCCCGTTCTAAATCCAGTTTACACTTTATTGTTGTAATGAAAAGGCTTTTCATTTTCAATA |  |
| PavHYL1_‘Garnet’ |  | TCCTGGTTTTATTTCCTAATTTTCATGAACTGTTCGCTCCCCCGTTCTAAATCCAGTTTACACTTTATTGTTGTAATGAAAAGGCTTTTCATTTACAATA |  |
|  |  |  |  |
|  |  | ....|....|....|....|....|....|....|....|....|....|....|....|....|....|....|....|....|....|....|....| |  |
| ppa004305m |  | TTTTCTTGCTTCTACATTTATGTTACTATTTTGATTCAAAATTCCTAGCTTCTACTCTGTGTGCTTTCGTGTGATGATTTCCACCACAAAACAAAGAGTT |  |
| PRUAV015329 |  | ~~~~~~~~~~~~~~~~~~~~~~~~~~~~~~~~~~~~~~~~~~~~~~~~~~~~~~~~~~~~~~~~~~~~~~~~~~~~~~~~~~~~~~~~~~~~~~~~~~~~ |  |
| PavHYL1_‘Regina’ |  | GTTTCTTGCTTCTACATTTATTTTACTATTTTGATTCAAAATTCCTAGCTTTTACTCTGTGTGCTATCTTGTGATGATTTCCACCACAAAACAAAGAGTT |  |
| PavHYL1_‘Lapins’ |  | GTTTCTTTCTTCTACATTTATTTTACTATTTTGATTCAAAATTCCTAGCTTTTACTCTGTGTGCTATCTTGTGATGATTTCCACCACAAAACAAAGAGTT |  |
| PavHYL1_‘Garnet’ |  | GTTTCTTGCTTCTACATTTATTTTACTATTTTGATTCAAAATTCCTAGCTTTTACTCTGTGTGCTATCTTGTGATGATTTCCACCACAAAACAAAGAGTT |  |
|  |  |  |  |
|  |  | ....|....|....|....|....|....|....|....|....|....|....|....|....|....|....|....|....|....|....|....| |  |
| ppa004305m |  | GGGCTTTAGTGATTATGCGAATAAAGTTTTCATTTTTAACTTTTCTCCTTTTTTTATATTAAATTTTCTTTTATGGGTTTGGAGTCATGGCTTCTACATT |  |
| PRUAV015329 |  | ~~~~~~~~~~~~~~~~~~~~~~~~~~~~~~~~~~~~~~~~~~~~~~~~~~~~~~~~~~~~~~~~~~~~~~~~~~~~~~~~~~~~~~~~~~~~~~~~~~~~ |  |
| PavHYL1_‘Regina’ |  | GGGCTTTAGTGATTATGCGAATAAAGTTTAAATTTTTAACTTTTCTCGTTTTTTTAAATTAAATTTTCGTTTATGGGTTTGGAGTCATGGCTTCTACATT |  |
| PavHYL1_‘Lapins’ |  | GGGCTTTAGTGATTATGCGAATAAAGTTTAAATTTTTAACTTTTCTCGTTTTTTTWAATTAAATTTTCGTTTATGGGTTTGGAGTCATGGCTTCTACATT |  |
| PavHYL1_‘Garnet’ |  | GGGCTTTAGTGATTATGCGAATAAAGTTTAAATTTTTAACTTTTCTCGTTTTTTTAAATTAAATTTTCGTTTATGGGTTTGGAGTCATGGCTTCTACATT |  |
|  |  |  |  |
|  |  | ....|....|....|....|....|....|....|....|....|....|....|....|....|....|....|....|....|....|....|....| |  |
| ppa004305m |  | TTAGTTTCTTATACTTCTAGCTACTCATAATTTTCTGATTATTTGTTTGTTTGATTTCCAGCACGAACAAAGAGCTGGGTTTCATACTGTTGTCAAGAAA |  |
| PRUAV015329 |  | ~~~~~~~~~~~~~~~~~~~~~~~~~~~~~~~~~~~~~~~~~~~~~~~~~~~~~~~~~~~~~C~CGAACAGAGAGCTGGGTTTCATTCTGTTTTCAAGAAA |  |
| PavHYL1_‘Regina’ |  | TTAGTTTGTTGTACTTCTAGCTACTCATAATTTTCTGATTATTTGTTTGTTTAATT~CCAGC~CGAACAGAGAGCTG~~TT~CATTCCG |  |
| PavHYL1_‘Lapins’ |  | TTAGTTTGTTGTACTTCTAGCTACTCATAATTTTCTGATTATTTGTTTGTTTAATT~CCAGC~CGAACAGAGAGCTGGTG~~CATTACT |  |
| PavHYL1_‘Garnet’ |  | TTAGTTTGTTGTACTTCTAGCTACTCATAATTTTCTGATTATTTGTTTGTTTAATT~CCAGC~CGAACAGAGAGCTGGTG~~CATTTCT |  |
|  |  |  |  |
|  |  | ....|....|....|....|....|....|....|....|....|....|....|....|....|....|....|....|....|....|....|....| |  |
| ppa004305m |  | CTGTCAGAGCTATGGCCACAAACGAAGGCTTTCAAGGTATTCACAATATTAATCCCTTTTGTTTCTCCTAAGTTTTTGTTTTTGTCTTGGTGCTATGTAT |  |
| PRUAV015329 |  | CTGTCAGAGCTATGGCCACAAACGAAGCCTTTCAAG~~~~~~~~~~~~~~~~~~~~~~~~~~~~~~~~~~~~~~~~~~~~~~~~~~~~~~~~~~~~~~~~ |  |
| PavHYL1_‘Regina’ |  |  |  |
| PavHYL1_‘Lapins’ |  |  |  |
| PavHYL1_‘Garnet’ |  |  |  |

***KS***

|  |  | ....|....|....|....|....|....|....|....|....|....|....|....|....|....|....|....|....|....|....|....| |  |
| --- | --- | --- | --- |
| ppa001902m | 1501 | TGATGCTGCCACCTTTGCAATGGCATTTCGGCTCTTACGTGTTAATGGATATGATGTTTCTGCAGGTATTTTCAGTTTAGGTGCATTGGTCTTCATGAGT |  |
| PRUAV034493 |  | TGATGCTGCCACCTGTGCGATGGCATTTCGGCTCTTACGTGTTAATGGATATGATGTTTCTGCAG~~~~~~~~~~~~~~~~~~~~~~~~~~~~~~~~~~~ |  |
| PavKS_‘Regina’ |  |  |  |
| PavKS_‘Lapins’ |  | ~~~~~~~~~~~~~~~~~~~~~~~~~~~~~~~~~~~~~~~~~~~~~~~~~~~~~~~~ggctgaggctttcggctcttacgtgtgcatttcggctccttacg |  |
| PavKS_‘Garnet’ |  | ~~~~~~~~~~~~~~~~~~~~~~~~~~~~~~~~~~~~~~~~~~~~~~~~~~~~~~~~~~~gtgCAG~TATTTTCACTTTAGGTGCGTTGGTCTTCATGAGT |  |
|  |  |  |  |
|  |  | ....|....|....|....|....|....|....|....|....|....|....|....|....|....|....|....|....|....|....|....| |  |
| ppa001902m |  | TTGTTTTTGGCAAAAAATATTTAATTGATTCATGCTGTCTTGTAGATCCATTAAGTCAATTTTCAGAAGATTGTTTCTTTAATTCCCTTGGAGGATATTT |  |
| PRUAV034493 |  | ~~~~~~~~~~~~~~~~~~~~~~~~~~~~~~~~~~~~~~~~~~~~~ATCCATTAAGTCAATTTTCAGAAGATTGTTTCTTTAATTCCCTTGGAGGATATTT |  |
| PavKS_‘Regina’ |  |  |  |
| PavKS_‘Lapins’ |  | tgtgcatttcggctcttacgtgtgcatttcggctcttacgtgtgcatttcggctcttacgtgtgcaattattgtcttacttgttcatttggaggATAATT |  |
| PavKS_‘Garnet’ |  | TTGTTTTTGACAAAAAATATTTAATTGATTCATGCTGTCTTGTAGATCCATTAAGTCAATTTTCAGAAGATTGTTTCTTTAATTCCCTTGGAGGATATTT |  |
|  |  |  |  |
|  |  | ....|....|....|....|....|....|....|....|....|....|....|....|....|....|....|....|....|....|....|....| |  |
| ppa001902m |  | GAAGGACATTGGTGCTGCCTTAGAATTGTTGAGGGCTTCAGAATTCATCATACATCCAGATGAATCAGTTATGGAGAAACAAAATTACTGGACAAGTCAT |  |
| PRUAV034493 |  | GAAGGACATTGGTGCTGCCTTAGAATTGTTGAGGGCTTCAGAATTCATCACACATCCAGATGAATCAGTTATGGAGAAACAAAATTACTGGACAAGCCAT |  |
| PavKS_‘Regina’ |  |  |  |
| PavKS_‘Lapins’ |  | GAAGGATTTTGGTGCTGCCTTAGAATTGTTGAGGGCTTCAGAATTCATCACACATCCAGATGAATCAGTTATGGAGAAACAAAATTACTGGACAAGCCAT |  |
| PavKS_‘Garnet’ |  | GAAGGACATTGGTGCTGCCTTAGAATTGTTGAGGGCTTCAGAATTCATCACACATCCAGATGAATCAGTTATGGAGAAACAAAATTACTGGACAAGCCAT |  |
|  |  |  |  |
|  |  | ....|....|....|....|....|....|....|....|....|....|....|....|....|....|....|....|....|....|....|....| |  |
| ppa001902m |  | TTTCTGAAACAGGAGTTATCAAATACTTTAGTTCAGGGTCATATATTCAATAAGCACATTGTCCTAGAGGTAGCTTGTTATGAGAACTCTTATCATATTG |  |
| PRUAV034493 |  | TTTCTGAAACAGGAGTTATCAAATACTTTAGTTCAGGGTCATATATTCAATAAGCACATTGTCCTAGAG~~~~~~~~~~~~~~~~~~~~~~~~~~~~~~~ |  |
| PavKS_‘Regina’ |  |  |  |
| PavKS_‘Lapins’ |  | TTTCTGAAACAGGAGTTATCAAATACTTTAGTTCAGGGTCATATATTCAATAAGCACATTGTCCTAGAGGTAACTTGTTATGAGAACTCT~ATCATATTG |  |
| PavKS_‘Garnet’ |  | TTTCTGAAACAGGAGTTATCAAATACTTTAGTTCAGGGTCATATATTCAATAAGCACATTGTCCTAGAGGTAACTTGTTATGAGAACTCT~ATCATATTG |  |
|  |  |  |  |
|  |  | ....|....|....|....|....|....|....|....|....|....|....|....|....|....|....|....|....|....|....|....| |  |
| ppa001902m |  | ATTTGGTGTTTTAGCTTTTTATAATCCCTAAAAATGCAACTTT~GATTGTTTTCAGGTGGAAGATGTTCTTAAATTTCCTTCCTATGCAAATTTGGGTCG |  |
| PRUAV034493 |  | ~~~~~~~~~~~~~~~~~~~~~~~~~~~~~~~~~~~~~~~~~~~~~~~~~~~~~~~~GTGGAAGATGTTCTTAAATTTCCTTCCTACGCAAATTTGGGTCG |  |
| PavKS_‘Regina’ |  |  |  |
| PavKS_‘Lapins’ |  | ATTTGGTGTTTTAGCTTTTTGTAATCCCTAAAAATGCAACTTTTGATTGTTTTCAGGTGGAAGATGTTCTTAAATTTCCTTCCTATGCAAATTTGGGTCG |  |
| PavKS_‘Garnet’ |  | ATTTGGTGTTTTAGCTTTTTGTAATCCCTAAAAATGCAACTTTTGATTGTTTTCAGGTGGAAGATGTTCTTAAATTTCCTTCCTATGCAAATTTGGGTCG |  |
|  |  |  |  |
|  |  | ....|....|....|....|....|....|....|....|....|....|....|....|....|....|....|....|....|....|....|....| |  |
| ppa001902m |  | GTTGTCAACCAGGAGGGCTATAAAATATTACAACACAGATAGTACAAGGATTTTAAAATCTTCTTACCGGTAAACCTCTAGTATTTTCTTCCACAGCCCC |  |
| PRUAV034493 |  | GTTGTCAACCAGGAGGGCTATAAAATATTACAACACAGATAGTACAAGGATTTTAAAATCTTCTTACCG~~~~~~~~~~~~~~~~~~~~~~~~~~~~~~~ |  |
| PavKS_‘Regina’ |  |  |  |
| PavKS_‘Lapins’ |  | GTTGTCAACCAGGAGGGCTATAAAATATTACAACACAGATAGTACAAGGATTTTAAAATCTTCTTACCGGTAAACCTCCAGTGTTTTCTTCCACAGCCCC |  |
| PavKS_‘Garnet’ |  | GTTGTCAACCAGGAGGGCTATAAAATATTACAACACAGATAGTACAAGGATTTTAAAATCTTCTTACCGGTAAACCTCCAGTGTTTTCTTCCACAGCCCC |  |
|  |  |  |  |
|  |  | ....|....|....|....|....|....|....|....|....|....|....|....|....|....|....|....|....|....|....|....| |  |
| ppa001902m |  | TGTTCTTCCTTTTCCTTTTTAAGAAGAAATTGATTTCTGTTGAGTTATAATAATGGACCAGTGTATATTACTTATTGATCGTTATGCTCTTCTTATGTTA |  |
| PRUAV034493 |  | ~~~~~~~~~~~~~~~~~~~~~~~~~~~~~~~~~~~~~~~~~~~~~~~~~~~~~~~~~~~~~~~~~~~~~~~~~~~~~~~~~~~~~~~~~~~~~~~~~~~~ |  |
| PavKS_‘Regina’ |  |  |  |
| PavKS_‘Lapins’ |  | TGTTCTTCCTTTTCCTTTTTAAGAAGAAATTGATTTCTGTTGAGTTATTATGACGGACCAGTGTATATTACTTATTGATCGTTGTGCTTTTCTTATGTTA |  |
| PavKS_‘Garnet’ |  | TGTTCTTCCTTTTCCTTTTTAAGAAGAAATTGATTTCTGTTGAGTTATTATGACGGACCAGTGTATATTACTTATTGATCGTTGTGCTTTTCTTATGTTA |  |
|  |  |  |  |
|  |  | ....|....|....|....|....|....|....|....|....|....|....|....|....|....|....|....|....|....|....|....| |  |
| ppa001902m |  | GTTGTTTGAATATTGGCAATGAAGATTTCCTAAAATTGGCAGTGGATGACTTCAATATTTGCCAATCTATACACCGTGAAGAACTCAACCATCTTGCAAG |  |
| PRUAV034493 |  | ~CTGTTTGAATATTGGCAATGAAGATTTCCTAAAATTGGCAGTGGATGACTTCAATATTTGCCAATCTATACACCGTGAAGAACTCAACCATCTTGCAAG |  |
| PavKS_‘Regina’ |  |  |  |
| PavKS_‘Lapins’ |  | GCTGTTTGAATATTGGCAATGAAGATTTCCTAAAATTGGCAGTGGATGACTTCAATTG |  |
| PavKS_‘Garnet’ |  | GCTGTTTGAATATTGGCAATGAAGATTTCCTAAAATTGGCATAAAGTGAGACTtcaaa |  |

***KSb***

|  |  | ....|....|....|....|....|....|....|....|....|....|....|....|....|....|....|....|....|....|....|....| |  |
| --- | --- | --- | --- |
| ppa018714m | 201 | TATCCTTGCACTAAAGCGGTGGAGTGTTGGTGAAGAACAAATTAACAAGGGTATATCTGTTGCTCTTGCTTCATGCTAAGTTTCCTTCATGTTTTACT~~ |  |
| PRUAV032409 |  | ~~TCCTTGCACTAAAGCGGTGGAGTGTTGGCGAAGAACAAATTAACAAGG~~~~~~~~~~~~~~~~~~~~~~~~~~~~~~~~~~~~~~~~~~~~~~~~~~ |  |
| PavKSb_‘Regina’ |  |  |  |
| PavKSb_‘Lapins’ |  | ~~~~~~~~~~~~~~~~~~~~~~~~~~~~~~~~~~~~~ca~~~taa~~agggtatatgtgttgctcttgcttc~tgcttactttccttcatgctgtactaa |  |
| PavKSb_‘Garnet’ |  | ~~~~~~~~~~~~~~~~~~~~~~~~~~~~~~~~~~~~~~aaa~~aa~aagg~tatatGTGTTGCTCTTGCTTCATGCTTAGTTTCCTTCATGCTGTACTAA |  |
|  |  |  |  |
|  |  | ....|....|....|....|....|....|....|....|....|....|....|....|....|....|....|....|....|....|....|....| |  |
| ppa018714m |  | ~~~ACGCCTCTCTTGTTCTTTTAGGGCTACATTTTATTGAATCAAATTTAGCTTTAGCTACCGATGAAGAGCAACAATCTCCTGTTGGATTTAATATAAT |  |
| PRUAV032409 |  | ~~~~~~~~~~~~~~~~~~~~~~~~GGCTACGTTTTATTGAATCAAATTTAGCTTCAGCTACCGATGAAGAGCAACAATCTCCTGTTGGATTTAATATAAT |  |
| PavKSb_‘Regina’ |  |  |  |
| PavKSb_‘Lapins’ |  | tgaacgcctctcttgTTCTTTTATGGCTACGTTTTATTGAATCAAATTTAGCTTCAGCTACCGATGAAGAGCAACGAGAGAaaact |  |
| PavKSb_‘Garnet’ |  | TGAACGCCTCTCTTGTTCTTTTAGGGCTACGTTTTATTGAATCAAATTTAGCTTCAGCTACCGATGAAGAGCAACGAGAGAAAA |  |

***KSc***

|  |  | ....|....|....|....|....|....|....|....|....|....|....|....|....|....|....|....|....|....|....|....| |  |
| --- | --- | --- | --- |
| ppa019543m | 2501 | CTTATGAGCACCTGTGGACGTCTTCTGAATGATATCCAAGGCTTCAAGGTAGACAATATGCCCACATTATTCTTTCGAAAAGAAAAACTAGTATCCAATT |  |
| PRUAV058967 |  | CTTATGAGCACTTGTGGCCGTCTTCTCAATGATATCCAAAGCTGTAAG~~~~~~~~~~~~~~~~~~~~~~~~~~~~~~~~~~~~~~~~~~~~~~~~~~~~ |  |
| PavKSc_‘Regina’ |  | ~~~~~~~~~~~~~~AGGGGTGAGCGTGTCCGCTCCTCTCTCTTTGAA~~~~~~~~~~~~~~~~~~~~~~~CAAAAACGAATGAAACAAACTAATAACCGG |  |
| PavKSc_‘Lapins’ |  | ~~~~~~~~~~~~~~GGGGTGCGCAGTGTCCACTCCTCTCTCTGTTGTG~~~~~~~~~~~~~~~~~~~~~~CAAAGACGAAGGAAACATACTAATAACCGG |  |
| PavKSc_‘Garnet’ |  | ~~~~~~~~~~~~~~GCGGTAGTCGCACTCCCC~CCTTCTCTTTGAA~~~~~~~~~~~~~~~~~~~~~~~~CAAAAACGAATGAAACAAACTAATAACCGG |  |
|  |  |  |  |
|  |  | ....|....|....|....|....|....|....|....|....|....|....|....|....|....|....|....|....|....|....|....| |  |
| ppa019543m |  | TGTTATCGTTAACTCATAACTCCTGGCTGTGTTGCAGAGGGATTCCGCTGAAGGGAAGCTAAATGCTCTATCATTGACCATGATTCATGGGAACGGTGTC |  |
| PRUAV058967 |  | ~~~~~~~~~~~~~~~~~~~~~~~~~~~~~~~~~~~~~AGGGAGTTAGAGCAAGGGAAGCTAAATGCTGTGTCATTGGCCGTGATTCATGGCGGTGGTATT |  |
| PavKSc_‘Regina’ |  | CCATCATATAATATCGATAACTCTGCGCTATTTGCAGAGGGAGTTAGAGCAAGGGAAGCTAAATGCTGTGTCATTGGCCGTGATTCATGGCGGTGGTATT |  |
| PavKSc_‘Lapins’ |  | CCATCATATAATATCGATAACTCTGCGCTATTTGCAGAGGGAGTTAGAGCAAGGGAAGCTAAATGCTGTGTCATTGGCCGTGATTCATGGCGGTGGTATT |  |
| PavKSc_‘Garnet’ |  | CCATCAGATAATATCGATAACTCTGCGCTATTTGCAGAGGGAGTTAGAGCAAGGGAAGCTAAATGCTGTGTCATTGGCCGTGATTCATGGCGGTGGTATT |  |
|  |  |  |  |
|  |  | ....|....|....|....|....|....|....|....|....|....|....|....|....|....|....|....|....|....|....|....| |  |
| ppa019543m |  | GTTACTGAAGAAGAGGCCATCAATGAGATAAATAGTATTATAAAAAGTAAGAGGGGAGAGCTGCTACGATTAGTTTTACAGGAGACGGGTGTTGAGAGTG |  |
| PRUAV058967 |  | GTTACTGAGGAAGATGCCATCAATGAGATTCAGAATGTTATAAACCGTAAGACAAAAGAACTGCTAAGATTAGTTGTGCTGGAGAAGGGAAGCATAGTT~ |  |
| PavKSc_‘Regina’ |  | GTTACTGAGGAAGATGCCATCAATGAGATTCAGAATGTTATAAACCGTAAGACAAAAGAACTGCTAAGATTAGTTGTGCTGGAGAAGGGAAGCATAGTT~ |  |
| PavKSc_‘Lapins’ |  | GTTACTGAGGAAGATGCCATCAATGAGATTCAGAATGTTATAAACCGTAAGACAAAAGAACTGCTAAGATTAGTTGTGCTGGAGAAGGGAAGCATAGTT~ |  |
| PavKSc_‘Garnet’ |  | GTTACTGAGGAAGATGCCATCAATGAGATTCAGAATGTTATAAACCGTAAGACAAAAGAACTGCTAAGATTAGTTGTGCTGGAGAAGGGAAGCATAGTT~ |  |
|  |  |  |  |
|  |  | ....|....|....|....|....|....|....|....|....|....|....|....|....|....|....|....|....|....|....|....| |  |
| ppa019543m |  | TTTGTCCTACATTGAAATGTTAAGGGACCTAGCCTAGGTTTATAAGGAGTTAGGCTACTCCCCCCATTGCCAATTGGTTTTGAGGTGGAACCTCAACTTC |  |
| PRUAV058967 |  | ~~~~~~~~~~~~~~~~~~~~~~~~~~~~~~~~~~~~~~~~~~~~~~~~~~~~~~~~~~~~~~~~~~~~~~~~~~~~~~~~~~~~~~~~~~~~~~~~~~~~ |  |
| PavKSc_‘Regina’ |  | ~~~~~~~~~~~~~~~~~~~~~~~~~~~~~~~~~~~~~~~~~~~~~~~~~~~~~~~~~~~~~~~~~~~~~~~~~~~~~~~~~~~~~~~~~~~~~~~~~~~~ |  |
| PavKSc_‘Lapins’ |  | ~~~~~~~~~~~~~~~~~~~~~~~~~~~~~~~~~~~~~~~~~~~~~~~~~~~~~~~~~~~~~~~~~~~~~~~~~~~~~~~~~~~~~~~~~~~~~~~~~~~~ |  |
| PavKSc_‘Garnet’ |  | ~~~~~~~~~~~~~~~~~~~~~~~~~~~~~~~~~~~~~~~~~~~~~~~~~~~~~~~~~~~~~~~~~~~~~~~~~~~~~~~~~~~~~~~~~~~~~~~~~~~~ |  |
|  |  |  |  |
|  |  | ....|....|....|....|....|....|....|....|....|....|....|....|....|....|....|....|....|....|....|....| |  |
| ppa019543m |  | CTTCATGGTATCAGAGCAAGGCTTCACGTGTTTGGCCCAATGGCCACACATTCTCCCATGTCATCCAATATGTGTTGTCCACGTTAGGCTTGAAAGTTCG |  |
| PRUAV058967 |  | ~~~~~~~~~~~~~~~~~~~~~~~~~~~~~~~~~~~~~~~~~~~~~~~~~~~~~~~~~~~~~~~~~~~~~~~~~~~~~~~~~~~~~~~~~~~~~~~~~~~~ |  |
| PavKSc_‘Regina’ |  | ~~~~~~~~~~~~~~~~~~~~~~~~~~~~~~~~~~~~~~~~~~~~~~~~~~~~~~~~~~~~~~~~~~~~~~~~~~~~~~~~~~~~~~~~~~~~~~~~~~~~ |  |
| PavKSc_‘Lapins’ |  | ~~~~~~~~~~~~~~~~~~~~~~~~~~~~~~~~~~~~~~~~~~~~~~~~~~~~~~~~~~~~~~~~~~~~~~~~~~~~~~~~~~~~~~~~~~~~~~~~~~~~ |  |
| PavKSc_‘Garnet’ |  | ~~~~~~~~~~~~~~~~~~~~~~~~~~~~~~~~~~~~~~~~~~~~~~~~~~~~~~~~~~~~~~~~~~~~~~~~~~~~~~~~~~~~~~~~~~~~~~~~~~~~ |  |
|  |  |  |  |
|  |  | ....|....|....|....|....|....|....|....|....|....|....|....|....|....|....|....|....|....|....|....| |  |
| ppa019543m |  | CCACACGTGCGGGGGCGTGTTGAGAGTGTTTGTCCCATATTGAAATGTTAAGGGACCTAGCCTAGGTTTATAAGGAGTTGGGCTACTCCCCCCATTGCCA |  |
| PRUAV058967 |  | ~~~~~~~~~~~~~~~~~~~~~~~~~~~~~~~~~~~~~~~~~~~~~~~~~~~~~~~~~~~~~~~~~~~~~~~~~~~~~~~~~~~~~~~~~~~~~~~~~~~~ |  |
| PavKSc_‘Regina’ |  | ~~~~~~~~~~~~~~~~~~~~~~~~~~~~~~~~~~~~~~~~~~~~~~~~~~~~~~~~~~~~~~~~~~~~~~~~~~~~~~~~~~~~~~~~~~~~~~~~~~~~ |  |
| PavKSc_‘Lapins’ |  | ~~~~~~~~~~~~~~~~~~~~~~~~~~~~~~~~~~~~~~~~~~~~~~~~~~~~~~~~~~~~~~~~~~~~~~~~~~~~~~~~~~~~~~~~~~~~~~~~~~~~ |  |
| PavKSc_‘Garnet’ |  | ~~~~~~~~~~~~~~~~~~~~~~~~~~~~~~~~~~~~~~~~~~~~~~~~~~~~~~~~~~~~~~~~~~~~~~~~~~~~~~~~~~~~~~~~~~~~~~~~~~~~ |  |
|  |  |  |  |
|  |  | ....|....|....|....|....|....|....|....|....|....|....|....|....|....|....|....|....|....|....|....| |  |
| ppa019543m |  | ATTGGTTTTGGGGTGGAACCTCAACTTCCTTCAATGGGTAGCATAGCCAAGGGCTTGCAAGGATTTGGTTTGGAACATGAGCAAAGTGCTCCACTTTTTT |  |
| PRUAV058967 |  | ~~~~~~~~~~~~~~~~~~~~~~~~~~~~~~~~~~~~~~~~~~~~~~CCAAGAGCATGCAAGAATTTGTTTTGGAACATGAACAAAGTGATCCACCTGTTT |  |
| PavKSc_‘Regina’ |  | ~~~~~~~~~~~~~~~~~~~~~~~~~~~~~~~~~~~~~~~~~~~~~~CCAAGAGCATGCAAGAATTTGAAA~AATTTGCAAAAATTTGACAACA |  |
| PavKSc_‘Lapins’ |  | ~~~~~~~~~~~~~~~~~~~~~~~~~~~~~~~~~~~~~~~~~~~~~~CCAAGAGCATGCAAGAATTTGATATAATTTGCAAGAATTTGACAAA |  |
| PavKSc_‘Garnet’ |  | ~~~~~~~~~~~~~~~~~~~~~~~~~~~~~~~~~~~~~~~~~~~~~~CCAAGAGCAT~CAAGAATTTGAAA~~AGCTGCCAAAATTTGATAAA |  |

***LDL1***

|  |  | ....|....|....|....|....|....|....|....|....|....|....|....|....|....|....|....|....|....|....|....| |  |
| --- | --- | --- | --- |
| ppa001504m | 1101 | TTCCTTAGGGACTGCACTCGAAGCTTTTCGACGTGCTTATAGTGTTGCCCAAGACCCGCAGGAGCGTATGCTCTTGGATTGGCATCTGGCTAACTTGGAA |  |
| PRUAV067054 |  | TTCCTTAGGGACTGCACTTGAAGCTTTTCGACGTGCTTATAGTGTTGCCCAAGACCCGCAGGAGCGTATGCTCTTGGATTGGCATCTGGCTAACTTGGAA |  |
| PavLDL1_‘Regina’ |  | ~~~~~~~~~~~~~~~~~~~~~~~~~~~~~~~~~~~~~~~~~~~~~~~~~~~~~~~~~~~~~~~~~~~~~~~~~~~~~~~~~~~~~~~~~~cggACTGGGA |  |
| PavLDL1_‘Lapins’ |  | ~~~~~~~~~~~~~~~~~~~~~~~~~~~~~~~~~~~~~~~~~~~~~~~~~~~~~~~~~~~~~~~~~~~~~~~~~~~~~~~~~~~~~~~~~~~~~~~~~~~~ |  |
| PavLDL1_‘Garnet’ |  | ~~~~~~~~~~~~~~~~~~~~~~~~~~~~~~~~~~~~~~~~~~~~~~~~~~~~~~~~~~~~~~~~~~~~~~~~~~~~~~~~~~~~~~~~~~ccgGCATGCA |  |
|  |  |  |  |
|  |  | ....|....|....|....|....|....|....|....|....|....|....|....|....|....|....|....|....|....|....|....| |  |
| ppa001504m |  | TATGCAAA~TGCTTCCTTAATGTCCAATTTGTCCATGGCCTATTGGGATCAGGATGATCCCTATGAGATGGGTGGTGACCATTGTTTTATTCCGGGAGGC |  |
| PRUAV067054 |  | TACGCAAA~TGCTTCCTTAATGTCCAATTTGTCCATGGCCTATTGG~ATCAGGATGATCCCTATGAGA |  |
| PavLDL1_‘Regina’ |  | T~CGCAA~~TGCTTCCTTAATGTCCAATTTGTCCATGGCCTATTGGGATCAGGATGATCCCTATGAGATGGGTGGTGACCATTGTTTTATTCCGGGAGGC |  |
| PavLDL1_‘Lapins’ |  | ~ACGCAAAATGCTTCCTTAATGTCCAATTTGTCCATGGCCTATTGGGATCAGGATGATCCCTATGAGATGGGTGGTGACCATTGTTTTATTCCGGGAGGC |  |
| PavLDL1_‘Garnet’ |  | ~ACGCAAA~TGCTTCCTTAATGTCCAATTTGTCCATGGCCTATTGGGATCAGGATGATCCCTATGAGATGGGTGGTGACCATTGTTTTATTCCGGGAGGC |  |
|  |  |  |  |
|  |  | ....|....|....|....|....|....|....|....|....|....|....|....|....|....|....|....|....|....|....|....| |  |
| ppa001504m |  | AATGAGACCTTCGTGCGATCCCTTTCAGAGGGCCTTCCAATCTTCTATGAAAGGACTGTGCAAAGTATTAGGTACGGCTCTGATGGGGTTTTGGTTTACG |  |
| PRUAV067054 |  |  |  |
| PavLDL1_‘Regina’ |  | AATGAGACCTTTGTGCGATCCCTTTCAGAGGGCCTTCCAATCTTCTATGAAAGGACTGTGCAAAGTATTAGGTACGGCTCTGATGGGATTTTGGTTTACG |  |
| PavLDL1_‘Lapins’ |  | AATGAGACCTTTGTGCGATCCCTTTCAGAGGGCCTTCCAATCTTCTATGAAAGGACTGTGCAAAGTATTAGGTACGGCTCTGATGGGATTTTGGTTTACG |  |
| PavLDL1_‘Garnet’ |  | AATGAGACCTTTGTGCGATCCCTTTCAGAGGGCCTTCCAATCTTCTATGAAAGGACTGTGCAAAGTATTAGGTACGGCTCTGATGGGATTTTGGTTTACG |  |
|  |  |  |  |
|  |  | ....|....|....|....|....|....|....|....|....|....|....|....|....|....|....|....|....|....|....|....| |  |
| ppa001504m |  | CAAATGGTCAGGAGTTTCGCGGGGACATGGTTCTTTGCACGGTCCCATTAGGTGTGCTTAAGAAGGGTTCCATTGAATTTGTCCCAGAGCTTCCACAAAG |  |
| PRUAV067054 |  |  |  |
| PavLDL1_‘Regina’ |  | CAAATGGTCAGGAGTTTCGCGGGGACATGGTTCTTTGCACGGTCCCATTAGGTGTGCTTAAGAAGGGTTCCATTGAATTTGTCCCAGAGCTTCCACAAAG |  |
| PavLDL1_‘Lapins’ |  | CAAATGGTCAGGAGTTTCGCGGGGACATGGTTCTTTGCACGGTCCCATTAGGTGTGCTTAAGAAGGGTTCCATTGAATTTGTCCCAGAGCTTCCACAAAG |  |
| PavLDL1_‘Garnet’ |  | CAAATGGTCAGGAGTTTCGCGGGGACATGGTTCTTTGCACGGTCCCATTAGGTGTGCTTAAGAAGGGTTCCATTGAATTTGTCCCAGAGCTTCCACAAAG |  |
|  |  |  |  |
|  |  | ....|....|....|....|....|....|....|....|....|....|....|....|....|....|....|....|....|....|....|....| |  |
| ppa001504m |  | GAAGAAAGATGCAATTCAGAGTTTAGGATTTGGATTATTGAATAAGGTTGCCATACTATTCCCTTATAATTTTTGGGGTGGAGATATTGATACTTTTGGG |  |
| PRUAV067054 |  |  |  |
| PavLDL1_‘Regina’ |  | GAAGAAAGATGCAATTCAGAGTTTAGGATTTGGATTATTGAATAAGGTTGCCATACTATTCCCTTATAATTTTTGGGGTGGAGATATTGATACTTTTGGG |  |
| PavLDL1_‘Lapins’ |  | GAAGAAAGATGCAATTCAGAGTTTAGGATTTGGATTATTGAATAAGGTTGCCATACTATTCCCTTATAATTTTTGGGGTGGAGATATTGATACTTTTGGG |  |
| PavLDL1_‘Garnet’ |  | GAAGAAAGATGCAATTCAGAGTTTAGGATTTGGATTATTGAATAAGGTTGCCATACTATTCCCTTATAATTTTTGGGGTGGAGATATTGATACTTTTGGG |  |
|  |  |  |  |
|  |  | ....|....|....|....|....|....|....|....|....|....|....|....|....|....|....|....|....|....|....|....| |  |
| ppa001504m |  | CATTTGACTGAGGATCCGAGTATGAGAGGTGAGTTCTTTCTGTTTTATAGCTACTCTTCTGTATCAGGAGGGCCTCTTCTGGTTGCTCTTGTTGCTGGAG |  |
| PRUAV067054 |  |  |  |
| PavLDL1_‘Regina’ |  | CATTTGACTGAGGATCCGAGCATGAGAGGTGAGTTCTTTCTGTTTTATAGCTACTCTTCTGTATCAGGAGGGCCTCTTCTGGTTGCTCTTGTTGCTGGAG |  |
| PavLDL1_‘Lapins’ |  | CATTTGACTGAGGATCCGAGCATGAGAGGTGAGTTCTTTCTGTTTTATAGCTACTCTTCTGTATCAGGAGGGCCTCTTCTGGTTGCTCTTGTTGCTGGAG |  |
| PavLDL1_‘Garnet’ |  | CATTTGACTGAGGATCCGAGCATGAGAGGTGAGTTCTTTCTGTTTTATAGCTACTCTTCTGTATCAGGAGGGCCTCTTCTGGTTGCTCTTGTTGCTGGAG |  |
|  |  |  |  |
|  |  | ....|....|....|....|....|....|....|....|....|....|....|....|....|....|....|....|....|....|....|....| |  |
| ppa001504m |  | ATGCTGCCATTAAGTTTGAGTTGATGTCTCCTGTGGAGTCTGTGAATAGGGTGTTGGAGATATTAAGGGGTATTTTCAACCCGAAAGGGATTGCTGTTCC |  |
| PRUAV067054 |  |  |  |
| PavLDL1_‘Regina’ |  | ATGCTGCCATTAAGTTTGAGTTGATGTCTCCTGTGAGTTCTGTGAA |  |
| PavLDL1_‘Lapins’ |  | ATGCTGCCATTAAGTTTGAGTTGATGTCTCCTGTGAATCCTGTGAAGA |  |
| PavLDL1_‘Garnet’ |  | ATGCTGCCATTAAGTTTGAGTTGATGTCTCCTGTGAGGTCCTGTGAA |  |

***LDL2***

|  |  | ....|....|....|....|....|....|....|....|....|....|....|....|....|....|....|....|....|....|....|....| |  |
| --- | --- | --- | --- |
| ppa001881m | 951 | TATAATGGATACATTAATTTTGGGGTTGCACCATCATTTGTGGCTAGTATGCCAGAGGAGGCAACCGAAGCGTCTGTCATTATTGTTGGTGCGGGACTTG |  |
| PRUAV023731 |  | tataatggatacattaattttggggttncaccatcatttgtggctaatgtgccagaggaggcaatcgaagggtctgtcattattgttggagcgggac |  |
| PavLDL2_‘Regina’ |  | ~~~~~~~~~~~~~~~~~~~~~~~~~~~~~~~~~~~~~~~~~~~~~~~~~~~~~~~~~~~~~~~~~~catcaggatggcgcatgccagaggatgctgggtt |  |
| PavLDL2_‘Lapins’ |  | ~~~~~~~~~~~~~~~~~~~~~~~~~~~~~~~~~~~~~~~~~~~~~~~~~~~~~~~~~~~~~~~~~~~ccaaagggaggtgtgt~~tggatcggATACTTG |  |
| PavLDL2_‘Garnet’ |  | ~~~~~~~~~~~~~~~~~~~~~~~~~~~~~~~~~~~~~~~~~~~~~~~~~~~~~~~~~~~~~~~~~~~gaatgagagcacagctatgccgaggaggcgggg |  |
|  |  |  |  |
|  |  | ....|....|....|....|....|....|....|....|....|....|....|....|....|....|....|....|....|....|....|....| |  |
| ppa001881m |  | CTGGGCTAGCGGCTGCAAGGCAGCTTCTGTCA~~~~~~TTGGGTTTCAAGGTGGCTGTTTTAGAAGGTAGGAATCGACCTGGTGGAAGAGTTTACACCCA |  |
| PRUAV023731 |  |  |  |
| PavLDL2_‘Regina’ |  | agcctaaggagggggc~~ggctgcca~~~~~~~~~~~~ttgggtttca~cgtggctgTTTTAAAAGGTAGGAATCGACCTGGTGGAAGAGTTTACACCCA |  |
| PavLDL2_‘Lapins’ |  | CTGGGCTAGCGGCTGCAAGGCAGCTTCTG~~TCA~~~~TTGGGTTTCAAGGTGGCTGTTTTAGAAGGTAGGAATCGACCTGGTGGAAGAGTTTACACCCA |  |
| PavLDL2_‘Garnet’ |  | tatgcctt~~~~~~aggaggcagcttctgccttaggAGTTGGG~~~~~~~GTGGCTGTTTTAGAAGGTAGGAATCGACCTGGTGGAAGAGTTTACACCCA |  |
|  |  |  |  |
|  |  | ....|....|....|....|....|....|....|....|....|....|....|....|....|....|....|....|....|....|....|....| |  |
| ppa001881m |  | AAAGATGGGGCAGGATGACAAATTTTCTGCAGTGGATCTTGGCGGCAGTGTCATTACTGGCATCCATGCTAATCCTCTTGGAGTTCTGGCCAGGCAACTT |  |
| PRUAV023731 |  |  |  |
| PavLDL2_‘Regina’ |  | AAAGATGGGGCAGGATGACAAATTTTCTGCAGTGGATCTTGGTGGCAGTGTCATTACTGGCATCCATGCTAATCCACTTGGAGTTCTGGCCAGGCAACTT |  |
| PavLDL2_‘Lapins’ |  | AAAGATGGGGCAGGATGACAAATTTTCTGCAGTGGATCTTGGTGGCAGTGTCATTACTGGCATCCATGCTAATCCACTTGGAGTTCTGGCCAGGCAACTT |  |
| PavLDL2_‘Garnet’ |  | AAAGATGGGGCAGGATGACAAATTTTCTGCAGTGGATCTTGGTGGCAGTGTCATTACTGGCATCCATGCTAATCCACTTGGAGTTCTGGCCAGGCAACTT |  |
|  |  |  |  |
|  |  | ....|....|....|....|....|....|....|....|....|....|....|....|....|....|....|....|....|....|....|....| |  |
| ppa001881m |  | TCCATTCCGCTTCATAAGGTCAGAGATAAGTGTCCTCTGTACAAGCCCGATGGGACACCTGTTGATAAGGACATTGATTCTAAGATTGAAGTCATCTTTA |  |
| PRUAV023731 |  |  |  |
| PavLDL2_‘Regina’ |  | TCTATTCCGCTTCATAAGGTCAGAGATAAGTGTCCTCTGTACAAGCCTGATGGGACACCTGTTGATAAGGACATTGATTCTAAGATTGAAGTCATCTTTA |  |
| PavLDL2_‘Lapins’ |  | TCTATTCCGCTTCATAAGGTCAGAGATAAGTGTCCTCTGTACAAGCCTGATGGGACACCTGTTGATAAGGACATTGATTCTAAGATTGAAGTCATCTTTA |  |
| PavLDL2_‘Garnet’ |  | TCTATTCCGCTTCATAAGGTCAGAGATAAGTGTCCTCTGTACAAGCCTGATGGGACACCTGTTGATAAGGACATTGATTCTAAGATTGAAGTCATCTTTA |  |
|  |  |  |  |
|  |  | ....|....|....|....|....|....|....|....|....|....|....|....|....|....|....|....|....|....|....|....| |  |
| ppa001881m |  | ATAAGTTGCTTGACAAAGTCATGGAACTCAGACAAACTATGGGTGGGTTTGGAAATGATGTATCTTTGGGTTCAGTTTTGGAGACACTGAGGCAGTTGTA |  |
| PRUAV023731 |  |  |  |
| PavLDL2_‘Regina’ |  | ATAAGTTGCTTGACAAAGTCATGGAACTCAGACAAACTATGGGTGGGTTTGGAAATGATATATCTTTGGGTTCAGTTTTGGAGACACTGAGGCAGTTGTA |  |
| PavLDL2_‘Lapins’ |  | ATAAGTTGCTTGACAAAGTCATGGAACTCAGACAAACTATGGGTGGGTTTGGAAATGATATATCTTTGGGTTCAGTTTTGGAGACACTGAGGCAGTTGTA |  |
| PavLDL2_‘Garnet’ |  | ATAAGTTGCTTGACAAAGTCATGGAACTCAGACAAACTATGGGTGGGTTTGGAAATGATATATCTTTGGGTTCAGTTTTGGAGACACTGAGGCAGTTGTA |  |
|  |  |  |  |
|  |  | ....|....|....|....|....|....|....|....|....|....|....|....|....|....|....|....|....|....|....|....| |  |
| ppa001881m |  | TGGTGTTGCTAGAAGTACTGAGGAGAGGCAACTTCTTGATTGGCATTTGGCAAATTTGGAATATGCAAATGCTGGATGTCTATCGAATCTCTCAGCTAAT |  |
| PRUAV023731 |  |  |  |
| PavLDL2_‘Regina’ |  | TGGTGTTGCTAGAAGTACTGAGGAGAGGCAACTTCTTGACTGGCATCTGGCAAATTTGGAATATGCAAATGCTGGATGTCTATCAAATCTCTCAGCTACT |  |
| PavLDL2_‘Lapins’ |  | TGGTGTTGCTAGAAGTACTGAGGAGAGGCAACTTCTTGACTGGCATCTGGCAAATTTGGAATATGCAAATGCTGGATGTCTATCAAATCTCTCAGCTACT |  |
| PavLDL2_‘Garnet’ |  | TGGTGTTGCTAGAAGTACTGAGGAGAGGCAACTTCTTGACTGGCATCTGGCAAATTTGGAATATGCAAATGCTGGATGTCTATCAAATCTCTCAGCTACT |  |
|  |  |  |  |
|  |  | ....|....|....|....|....|....|....|....|....|....|....|....|....|....|....|....|....|....|....|....| |  |
| ppa001881m |  | TATTGGGATCAGGATGATCCTTATGAGATGGGTGGGGACCACTGCTTTCTTGCTGGAGGTAATTGGAGATTGATAAAAGCATTGTGTGAAGGGGTTCCCA |  |
| PRUAV023731 |  |  |  |
| PavLDL2_‘Regina’ |  | TATTGGGATCAGGATGATCCTTATGAGATGGGTGGGG |  |
| PavLDL2_‘Lapins’ |  | TATTGGGATCAGGATGATCCTTATGAGTTGGGGTGGGG |  |
| PavLDL2_‘Garnet’ |  | TATTGGGATCAGGATGATCCTTATGAGATGGGGTGGGG |  |

***LHY***

|  |  | ....|....|....|....|....|....|....|....|....|....|....|....|....|....|....|....|....|....|....|....| |  |
| --- | --- | --- | --- |
| ppa001765m | 201 | CGAAGCCATTCGGATCCAGCTGCCACTGCCGGAAACTATGCCTTCTCCG~~~~~~~~~~~GTGAGTTGATTGAAGTTGCAGCCAGTCGCTGTCTTCGTCT |  |
| PRUAV000054 |  | CGAAGCAATTCGGATCCAGCTGCCATAGCCGGAAACTATGCCTTCTCCGGCGAGTGTCCGGTGAGTTGATTGAAGTTGAAGCCAGTCGCTGTCTTCGTCT |  |
| PavLHY_‘Regina’ |  | ~~~~~~~~~~~~~~~~~~~~~~~~~~~~~~~~~~~~~~~~~~~~~~~~~~~~~~~cgggccgagaatgaTGCAGCTTGATGCCAGTCGCTGTCTTCGTCT |  |
| PavLHY_‘Lapins’ |  | ~~~~~~~~~~~~~~~~~~~~~~~~~~~~~~~~~~~~~~~~~~~~~~~~~~~~~aagcataaagtattgatgcagCTTGATGCCAGTCGCTGTCTTCGTCT |  |
| PavLHY_‘Garnet’ |  | ~~~~~~~~~~~~~~~~~~~~~~~~~~~~~~~~~~~~~~~~~~~~~~~~~~~~~~acgcctgggtaatgatgCAGCTTGATGCCAGTCGCTGTCTTCGTCT |  |
|  |  |  |  |
|  |  | ....|....|....|....|....|....|....|....|....|....|....|....|....|....|....|....|....|....|....|....| |  |
| ppa001765m |  | CGAAAGCTCTCAGTCCACCATGGCTTTCTCAGAAGGTACGCTTTCGATTTCCTCAGGGATTGATTGGCCTTGTTCTTCTCCACTCTCCGTTTGGTTGCTG |  |
| PRUAV000054 |  | CGAAAGCTCTCTGTCCACCATGGCTTTCTCAGAAG~~~~~~~~~~~~~~~~~~~~~~~~~~~~~~~~~~~~~~~~~~~~~~~~~~~~~~~~~~~~~~~~~ |  |
| PavLHY_‘Regina’ |  | CGAAAGCTCTCTGTCCACCATGGMTTTCTCAGAAGGTACGCTTTCGATTTCCTCAGGGATTGATTGGCCTTGTTCTTCTCCACTCTCCGTTTGGTTGCTT |  |
| PavLHY_‘Lapins’ |  | CGAAAGCTCTCTGTCCACCATGGATTTCTCAGAAGGTACGCTTTCGATTTCCTCAGGGATTGATTGGCCTTGTTCTTCTCCACTCTCCGTTTGGTTGCTT |  |
| PavLHY_‘Garnet’ |  | CGAAAGCTCTCTGTCCACCATGGMTTTCTCAGAAGGTACGCTTTCGATTTCCTCAGGGATTGATTGGCCTTGTTCTTCTCCACTCTCCGTTTGGTTGCTT |  |
|  |  |  |  |
|  |  | ....|....|....|....|....|....|....|....|....|....|....|....|....|....|....|....|....|....|....|....| |  |
| ppa001765m |  | ATCAGAAAATTCAACGAGCCTAAAAAAAGTTCAAAATATAAATCTATACATGCTAAGGATGAAGAAGAGGACTGATAAAGTTCATCTCAGCTGAGCAAGT |  |
| PRUAV000054 |  | ~~~~~~~~~~~~~~~~~~~~~~~~~~~~~~~~~~~~~~~~~~~~~~~~~~~~~~~~~~~~~~~~~~~~~~~~~~~~~~~~~~~~~~~~~~~~~~~~~~~~ |  |
| PavLHY_‘Regina’ |  | ATCAGAAAATTCAACGAGC~TAAAAAAAGTTCAAAATATAAATCTATACATGCTAAGAATGAAGAAGAGGACTGATAAAGTTCATCTCAGCTGAGGAAGT |  |
| PavLHY_‘Lapins’ |  | ATCAGAAAATTCAACGAGC~TAAAAAAAGTTCAAAATATAAATCTATACATGCTAAGAATGAAGAAGAGGACTGATAAAGTTCATCTCAGCTGAGGAAGT |  |
| PavLHY_‘Garnet’ |  | ATCAGAAAATTCAACGAGC~TAAAAAAAGTTCAAAATATAAATCTATACATGCTAAGAATGAAGAAGAGGACTGATAAAGTTCATCTCAGCTGAGGAAGT |  |
|  |  |  |  |
|  |  | ....|....|....|....|....|....|....|....|....|....|....|....|....|....|....|....|....|....|....|....| |  |
| ppa001765m |  | GGTGGCGAAAGCTCATGTTTGGACAATGAAACTTAATTTTCTAAATTTTGTTGTTTTCATTTTCTCGGCAACCAAACAGATGGTTAAGATTGCAGAACAA |  |
| PRUAV000054 |  | ~~~~~~~~~~~~~~~~~~~~~~~~~~~~~~~~~~~~~~~~~~~~~~~~~~~~~~~~~~~~~~~~~~~~~~~~~~~~~~~~~~~~~~~~~~~~~~~~~~~~ |  |
| PavLHY_‘Regina’ |  | GGTGGCGAAAGCTCATGTTTGGACAATTAAACTTAATTTTCTAAATTTTGTTGTTTTCATTTTCTCGGCAACCAAACAGATGGTTAAGATTGCAGAACAA |  |
| PavLHY_‘Lapins’ |  | GGTGGCGAAAGCTCATGTTTGGACAATTAAACTTAATTTTCTAAATTTTGTTGTTTTCATTTTCTCGGCAACCAAACAGATGGTTAAGATTGCAGAACAA |  |
| PavLHY_‘Garnet’ |  | GGTGGCGAAAGCTCATGTTTGGACAATTAAACTTAATTTTCTAAATTTTGTTGTTTTCATTTTCTCGGCAACCAAACAGATGGTTAAGATTGCAGAACAA |  |
|  |  |  |  |
|  |  | ....|....|....|....|....|....|....|....|....|....|....|....|....|....|....|....|....|....|....|....| |  |
| ppa001765m |  | GGTCTGTCTGAGTCAGTAATAGAAATGGATGTTGTATACTGATCAAACTTTCTCTCTCTTCTTGTTCGCCTTTTTTCTTTTTTCGTAGTTAAAGGTTTTG |  |
| PRUAV000054 |  | ~~~~~~~~~~~~~~~~~~~~~~~~~~~~~~~~~~~~~~~~~~~~~~~~~~~~~~~~~~~~~~~~~~~~~~~~~~~~~~~~~~~~~~~~TTAAAGGTTTTG |  |
| PavLHY_‘Regina’ |  | GGTCTGTCTGAGTCAGTAATAGAAATGGATATTGTTTACTGATCAAACTTTCTCTCTCTTCTTGTTCGCCTTTTTTC~~~~~~~GTAGTTAAAGGTTTTG |  |
| PavLHY_‘Lapins’ |  | GGTCTGTCTGAGTCAGTAATAGAAATGGATATTGTTTACTGATCAAACTTTCTCTCTCTTCTTGTTCGCCTTTTTTC~~~~~~~GTAGTTAAAGGTTTTG |  |
| PavLHY_‘Garnet’ |  | GGTCTGTCTGAGTCAGTAATAGAAATGGATATTGTTTACTGATCAAACTTTCTCTCTCTTCTTGTTCGCCTTTTTTC~~~~~~~GTAGTTAAAGGTTTTG |  |
|  |  |  |  |
|  |  | ....|....|....|....|....|....|....|....|....|....|....|....|....|....|....|....|....|....|....|....| |  |
| ppa001765m |  | GCGTGTTCCTGTTCGTTTTGGTGAGGATTCTGAATAAGGAAATTTATTTTAGTTCTTTCGGTGACGCGTCTCTCTCTCTGTGATGCGCGGTGGAGTCTGA |  |
| PRUAV000054 |  | GCGTGTGCCGGTTCGTTTTG~~~~~~~~~~~~~~~~~~~~~~~~~~~~~~~~~~~~~~~~~~~~~~~~~~~~~~~~~~~~~~~~~~~~~~~~~~~~~~~~ |  |
| PavLHY_‘Regina’ |  | GCGTGTGCCGGTTCGTTTTGGTGAGGATTCTGAATAAGGAAATTTATTTTAGTTCTTTCGGTGACGCCTCTCTCTCTCTGTGATGCGCGGTGGAGTCTGA |  |
| PavLHY_‘Lapins’ |  | GCGTGTGCCGGTTCGTTTTGGTGAGGATTCTGAATAAGGAAATTTATTTTAGTTCTTTCGGTGACGCCTCTCTCTCTCTGTGATGCGCGGTGGAGTCTGA |  |
| PavLHY_‘Garnet’ |  | GCGTGTGCCGGTTCGTTTTGGTGAGGATTCTGAATAAGGAAATTTATTTTAGTTCTTTCGGTGACGCCTCTCTCTCTCTGTGATGCGCGGTGGAGTCTGA |  |
|  |  |  |  |
|  |  | ....|....|....|....|....|....|....|....|....|....|....|....|....|....|....|....|....|....|....|....| |  |
| ppa001765m |  | AATTTTCATAGATCAGTTCTGTTTTCGATTATTGGTTTATATAAGTATCATCTAGTTAGCTTCAGTGCATATGAAATTGGACTGCTTAATTCTGCTGCTA |  |
| PRUAV000054 |  | ~~~~~~~~~~~~~~~~~~~~~~~~~~~~~~~~~~~~~~~~~~~~~~~~~~~~~~~~~~~~~~~~~~~~~~~~~~~~~~~~~~~~~~~~~~~~~~~~~~~~ |  |
| PavLHY_‘Regina’ |  | AATTTTCATTAGAACAGTTCTGTTTTCGATTTTCG~~~~~GTTTCTATAAAGTATCGTCTAGTTAGCTTCAGTGCATATGAAATTGGACTGCTTAATTCT |  |
| PavLHY_‘Lapins’ |  | AATTTTCATTAGAACAGTTCTGTTTTCGATTTTCG~~~~~GTTTCTATAAAGTATCGTCTAGTTAGCTTCAGTGCATATGAAATTGGACTGCTTAATTCT |  |
| PavLHY_‘Garnet’ |  | AATTTTCATTAGAACAGTTCTGTTTTCGATTTTCG~~~~~GTTTCTATAAAGTATCGTCTAGTTAGCTTCAGTGCATATGAAATTGGACTGCTTAATTCT |  |
|  |  |  |  |
|  |  | ....|....|....|....|....|....|....|....|....|....|....|....|....|....|....|....|....|....|....|....| |  |
| ppa001765m |  | TAAAATCAGAGACGGATCTCTACCGATTGCTGTGGCTTGTTTTCTTTTTGCAGTAGCATCACCTTCTCAGCTCTTCTTCAGTAGGACAACCAAGACGGCT |  |
| PRUAV000054 |  | ~~~~~~~~~~~~~~~~~~~~~~~~~~~~~~~~~~~~~~~~~~~~~~~~~~~~~TAGCATCACCTTCTCAGCTCTTCTTTAGTAGGACAACCAAGACGGCT |  |
| PavLHY_‘Regina’ |  | GCTGCTATAAAATCAGAGACGGATCTCTACCGATTGCTGTGGCTTGTTTTCTTTTTGCAGTAGCATCACCTTCTCAGCTCTTCTTTAGTAGGACAACCAA |  |
| PavLHY_‘Lapins’ |  | GCTGCTATAAAATCAGAGACGGATCTCTACCGATTGCTGTGGCTTGTTTTCTTTTTGCAGTAGCATCACCTTCTCAGCTCTTCTTTAGTAGGACAACCAA |  |
| PavLHY_‘Garnet’ |  | GCTGCTATAAAATCAGAGACGGATCTCTACCGATTGCTGTGGCTTGTTTTCTTTTTGCAGTAGCATCACCTTCTCAGCTCTTCTTTAGTAGGACAACCAA |  |
|  |  |  |  |
|  |  | ....|....|....|....|....|....|....|....|....|....|....|....|....|....|....|....|....|....|....|....| |  |
| ppa001765m |  | TTACTCGTTATAAAGCTCGCTGCTTTCATAAATGAGAGAAGGTCCTTGTTCTATCCCTTGCTCGTCAGAGAGGATTTGAAGCAGCGCAAGCTGCGCAGGT |  |
| PRUAV000054 |  | TTACTCGTACTAAACCTCGCTGCTTTCACAAATGAGAGAAGGTTCTTGTTCTATCCGTTGCTCGTCAGAGAGGATTTGAAGCAGCGCAAGCTGCGCGGGT |  |
| PavLHY_‘Regina’ |  | GACGGCTTTACTCGTACTAAACCTCGCTGCTTTCACAAATGAGAGAAGGTTCTTGTTCTATCCGTTGCTCGTCAGAGAGGATTGAAGGCAGGCGGAGTtt |  |
| PavLHY_‘Lapins’ |  | GACGGCTTTACTCGTACTAAACCTCGCTGCTTTCACAAATGAGAGAAGGTTCTTGTTCTATCCGTTGCTCGTCAGAGAGGATTGAAGCCAGCGGGGGTta |  |
| PavLHY_‘Garnet’ |  | GACGGCTTTACTCGTACTAAACCTCGCTGCTTTCACAAATGAGAGAAGGTTCTTGTTCTATCCGTTGCTCGTCAGAGAGGATTGAGAGCCCACGGGaggt |  |
|  |  |  |  |
|  |  | ....|....|....|....|....|....|....|....|....|....|....|....|....|....|....|....|....|....|....|....| |  |
| ppa001765m |  | CCGGTAATGGACACACAGTCATCTGGAGAAGATTTGGTCATTAAGGTAACACAGATGGACTTTCTTTTGTTAAATTTGGAATTGCCTAGTGAGGTCTAAG |  |
| PRUAV000054 |  | CCGGTAATGGACACACAGTCATCCGGAGAAGATTTGGTCATTAAG~~~~~~~~~~~~~~~~~~~~~~~~~~~~~~~~~~~~~~~~~~~~~~~~~~~~~~~ |  |
| PavLHY_‘Regina’ |  | Agta |  |
| PavLHY_‘Lapins’ |  |  |  |
| PavLHY_‘Garnet’ |  | T |  |

***MBF1A***

|  |  | ....|....|....|....|....|....|....|....|....|....|....|....|....|....|....|....|....|....|....|....| |  |
| --- | --- | --- | --- |
| ppa013074m | 2201 | CTTTTCTACTATGTGGCTTTGTGCTTAAATGCCATCTGATTTTGACTTGTAGATGAACGTGTGCCAAGTGAGCTGAAGAAAGCTATTATGCAAGCTCGAA |  |
| PRUAV001900 |  | ~~~~~~~~~~~~~~~~~~~~~~~~~~~~~~~~~~~~~~~~~~~~~~~~~~~~ATGAACGTGTGCCAAGTGAGCTGAAGAAAGCTATTATGCAAGCTCGAA |  |
| PavMBF1A_‘Regina’ |  | ~~~~~~~~~~~~~~~~~~~~~~~~~~~~~~~~~~~~~~~~~~~~~~~~~~~~~~~~~~tttttccaaagggagtgaagaaagatattatgcacgctcgaa |  |
| PavMBF1A_‘Lapins’ |  | ~~~~~~~~~~~~~~~~~~~~~~~~~~~~~~~~~~~~~~~~~~~~~~~~~~~~~~~~~~tttccccaaagggggagaagaaagatattatgcaagctcgaa |  |
| PavMBF1A_‘Garnet’ |  | ~~~~~~~~~~~~~~~~~~~~~~~~~~~~~~~~~~~~~~~~~~~~~~~~~~~~~~~~~~~~tttccaagtgagatgaagaaagatattatgcaagctcgaa |  |
|  |  |  |  |
|  |  | ....|....|....|....|....|....|....|....|....|....|....|....|....|....|....|....|....|....|....|....| |  |
| ppa013074m |  | TGGATAAGAAGCTTACCCAGTCTCAGCTTGCTCAAGTAATTCTCTCTCTCTCTCTCTCTCTCTCTCACACACACACACACACACACACACACACACACAC |  |
| PRUAV001900 |  | TGGATAAGAAGCTTACCCAGTCTCAGCTTGCTCAA~~~~~~~~~~~~~~~~~~~~~~~~~~~~~~~~~~~~~~~~~~~~~~~~~~~~~~~~~~~~~~~~~ |  |
| PavMBF1A_‘Regina’ |  | tggataagaagcttacccactctcagCTTGCTCAAGTAATTCTCTCTCTCTCTCTCTCTCTCTCTCTCTCTCA~TCTCTCTCTCTCTCT~~~~~~~~~~~ |  |
| PavMBF1A_‘Lapins’ |  | tggataagaagcttacccactctcAGCTTGCTCAAGTAATTCTCTCTCTCTCTCTCTCTCTCTCTCTCTCTCA~TCTCTCTCTCTCTCT~~~~~~~~~~~ |  |
| PavMBF1A_‘Garnet’ |  | tggataagaagcttacccactctcaGCTTGCTCAAGTAATTCTCTCTCTCTCTCTCTCTCTCTCTCTCTCTCA~TCTCTCTCTCTCTCT~~~~~~~~~~~ |  |
|  |  |  |  |
|  |  | ....|....|....|....|....|....|....|....|....|....|....|....|....|....|....|....|....|....|....|....| |  |
| ppa013074m |  | GCACATACATGCTTGCGCGTGTGGGCCCATCTGTGTATAGTTAGTGATGATCCGGAGTTATTTGCAGATTATCAATGAGAAGCCTCAAGTGATCCAAGAG |  |
| PRUAV001900 |  | ~~~~~~~~~~~~~~~~~~~~~~~~~~~~~~~~~~~~~~~~~~~~~~~~~~~~~~~~~~~~~~~~~~~ATTATCAATGAGAAGCCTCAAGTGATCCAAGAG |  |
| PavMBF1A_‘Regina’ |  | ~~~~~~~~~~~~~~~~~~~~~~~~~CCCATCTGTGTATAGTTAGTGATGATCTGGAGTTATTTGCAGATTATCAATGAGAAGCCTCAAGTGATCCAAGAG |  |
| PavMBF1A_‘Lapins’ |  | ~~~~~~~~~~~~~~~~~~~~~~~~~CCCATCTGTGTATAGTTAGTGATGATCTGGAGTTATTTGCAGATTATCAATGAGAAGCCTCAAGTGATCCAAGAG |  |
| PavMBF1A_‘Garnet’ |  | ~~~~~~~~~~~~~~~~~~~~~~~~~CCCATCTGTGTATAGTTAGTGATGATCTGGAGTTATTTGCAGATTATCAATGAGAAGCCTCAAGTGATCCAAGAG |  |
|  |  |  |  |
|  |  | ....|....|....|....|....|....|....|....|....|....|....|....|....|....|....|....|....|....|....|....| |  |
| ppa013074m |  | TATGAATCTGGGAAGGCTATTCCAAATCAGCAGGTAATTACCAAGTTGGAGAGAGCTCTTGGAGCGAAACTGCGTGGAAAGAAATAAGTAATCCAGGACG |  |
| PRUAV001900 |  | TACGAATCTGGGAAGGCTATTCCAAATCAGCAGGTAATTACCAAGTTGGAGAGAGCTCTTGGAGCGAAACTGCGTGGAAAGAAATAAGTAATCCAGGATG |  |
| PavMBF1A_‘Regina’ |  | TACGAATCTGGGAAGGCTATTCCAAATCAGCAGGTAATTACCAAGTTGGAGAGAGCTCTTGGAGCGAAACTGCGTGGAAAGAAATAAGTAATCCAGGATG |  |
| PavMBF1A_‘Lapins’ |  | TACGAATCTGGGAAGGCTATTCCAAATCAGCAGGTAATTACCAAGTTGGAGAGAGCTCTTGGAGCGAAACTGCGTGGAAAGAAATAAGTAATCCAGGATG |  |
| PavMBF1A_‘Garnet’ |  | TACGAATCTGGGAAGGCTATTCCAAATCAGCAGGTAATTACCAAGTTGGAGAGAGCTCTTGGAGCGAAACTGCGTGGAAAGAAATAAGTAATCCAGGATG |  |
|  |  |  |  |
|  |  | ....|....|....|....|....|....|....|....|....|....|....|....|....|....|....|....|....|....|....|....| |  |
| ppa013074m |  | GAAAATGAATCTCAGCTGGGATTTATGATTTTAAGTTTGAACTCTGATGTATTTGCACTTAAATCTGTAGCCAAAGTTTGAACTAATATGCTGCCCCTCA |  |
| PRUAV001900 |  | GAAAACGAATCTCAGCTGGGGGTTATGATTTTAAGTTTGAACTCTGATGTATTTGCACTTAAATCTGTAGCTAAAGTTTGAACTAATATGCTGCACCTCA |  |
| PavMBF1A_‘Regina’ |  | GAAAACGAATCTCAGCTGGGGGTTATGATTTTAAGTTTGAACTCTGATGTATTTGCACTTAAATCTGTAGCTAAAGTT~GAACTAATATGCTGCACGTCA |  |
| PavMBF1A_‘Lapins’ |  | GAAAACGAATCTCAGCTGGGGGTTATGATTTTAAGTTTGAACTCTGATGTATTTGCACTTAAATCTGTAGCTAAAGTT~GAACTAATATGCTGCACCTCA |  |
| PavMBF1A_‘Garnet’ |  | GAAAACGAATCTCAGCTGGGGGTTATGATTTTAAGTTTGAACTCTGATGTATTTGCACTTAAATCTGTAGCTAAAGTT~GAACTAATATGCTGCACCTCA |  |
|  |  |  |  |
|  |  | ....|....|....|....|....|....|....|....|....|....|....|....|....|....|....|....|....|....|....|....| |  |
| ppa013074m |  | TTCTGTAGTTATCTTCCAAAGGTCGTGTGTTTGGTGCAGTAAGTACTTGTGTTTTCTAAGAATATTTCATCAAATGACAAGTTCGCAAACAATTTCAACT |  |
| PRUAV001900 |  | TTCTGTAGTTATCTTCCAAAGGTCGTGTGTTTGGTGCAGGAAGTACTTGTGTTTTCTAAGAATATTTCATCAAATGACGAGTTCGCAAACAATTTCAACT |  |
| PavMBF1A_‘Regina’ |  | Ttctgtatttatcttccaaagggcgtgggtttggtga |  |
| PavMBF1A_‘Lapins’ |  | TTCTGTATTTATCTTCCAAAGGGCGTGTGTTTGGTGA |  |
| PavMBF1A_‘Garnet’ |  | Ttctgtatatatcttccaaagggcgtgtgtttggtga |  |

***MBF1C***

|  |  | ....|....|....|....|....|....|....|....|....|....|....|....|....|....|....|....|....|....|....|....| |  |
| --- | --- | --- | --- |
| ppa013016m | 1 | ATCCGACTCTCTCTCTAGATCTCCAACGATGCCGAGCAGAAACCCAGGAGTGATAACCCAGGACTGGGAGCCGGTGGTCATCCACAAGTCCAGGCCCAAG |  |
| PRUAV029737 |  | ATCCGACTCTCTCTCTAGATCTCCGACGATGCCGAGCAGAAACCCAGGAGTGATAACCCAGGACTGGGAGCCGGTGGTCATCCACAAGTCCAGGCCCAAG |  |
| PavMBF1C_‘Regina’ |  | ~~~~~~~~~~~~~~~~~~~~~~~~~~~~~~~~~~~~~~~~~~~~~~~~~~~~~~~~~ccaaggaagCCA~~CGGTGGTCATCCACAAGTCCAGGCCCAAG |  |
| PavMBF1C_‘Lapins’ |  | ~~~~~~~~~~~~~~~~~~~~~~~~~~~~~~~~~~~~~~~~~~~~~~~~~~~~~~~~~ccag~~ctgg~TG~CGGTGGTCATCCACAAGTCCAGGCCCAAG |  |
| PavMBF1C_‘Garnet’ |  | ~~~~~~~~~~~~~~~~~~~~~~~~~~~~~~~~~~~~~~~~~~~~~~~~~~~~~~~~~~ctgggatgGTA~~CGGTGGTCATCCACAAGTCCAGGCCCAAG |  |
|  |  |  |  |
|  |  | ....|....|....|....|....|....|....|....|....|....|....|....|....|....|....|....|....|....|....|....| |  |
| ppa013016m |  | GGTCAAGACCTCCGCGACCCGAAGGCTGTGAACCAGGCGCTCCGGTCCGGCGCGCCGATCCAGACCATAAAGAAATTCGACGCGGGCTCCAACAAGAAGG |  |
| PRUAV029737 |  | GGTCAAGACCTCCGCGACCCGAAGGCTGTGAACCAGGCGCTCCGGTCCGGCGCGCCGATCCAGACCATCAAGAAGTTCGACGCGGGCTCCAACAAGAAGG |  |
| PavMBF1C_‘Regina’ |  | GGTCAAGACCTCCGCGACCCGAAGGCTGTGAACCAGGCGCTCCGGTCCGGCGCGCCGATCCAGACCATCAAGAAGTTCGACGCGGGCTCCAACAAGAAGG |  |
| PavMBF1C_‘Lapins’ |  | GGTCAAGACCTCCGCGACCCGAAGGCTGTGAACCAGGCGCTCCGGTCCGGCGCGCCGATCCAGACCATCAAGAAGTTCGACGCGGGCTCCAACAAGAAGG |  |
| PavMBF1C_‘Garnet’ |  | GGTCAAGACCTCCGCGACCCGAAGGCTGTGAACCAGGCGCTCCGGTCCGGCGCGCCGATCCAGACCATCAAGAAGTTCGACGCGGGCTCCAACAAGAAGG |  |
|  |  |  |  |
|  |  | ....|....|....|....|....|....|....|....|....|....|....|....|....|....|....|....|....|....|....|....| |  |
| ppa013016m |  | CAGCGCCGGTCGTCAGCGTGAAGAAGCTCGAGGAAGGGACCGAGCCGGCGGCGCTGGACCGGGTCTCGACCGATGTGAGGCAGGCCATACAGAAGGCGCG |  |
| PRUAV029737 |  | CAGCGCCGGTCTTCAGCGTGAAGAAGCTCGAGGAAGGGACCGAGCCGGCCGCGCTGGACCGGGTCTCGAACGATGTGAGGCAGGCCATACAGAAGGCTCG |  |
| PavMBF1C_‘Regina’ |  | CAGCGCCGGTCTTCAGCGTGAAGAAGCTCGAGGAAGGGACCGAGCCGGCCGCGCTGGACCGGGTCTCGARCGATGTGAGGCAGGCCATACAGAAGGCTCG |  |
| PavMBF1C_‘Lapins’ |  | CAGCGCCGGTCGTCAGCGTGAAGAAGCTCGAGGAAGGGACCGAGCCGGCCGCGCTGGACCGGGTCTCGAGCGATGTGAGGCAGGCCATACAGAAGGCTCG |  |
| PavMBF1C_‘Garnet’ |  | CAGCGCCGGTCGTCAGCGTGAAGAAGCTCGAGGAAGGGACCGAGCCGGCCGCGCTGGACCGGGTCTCGAGCGATGTGAGGCAGGCCATACAGAAGGCTCG |  |
|  |  |  |  |
|  |  | ....|....|....|....|....|....|....|....|....|....|....|....|....|....|....|....|....|....|....|....| |  |
| ppa013016m |  | TCTGGCGAAGAAGTTGAGCCAGGCCGACCTGGCCAAGCGGATCAACGAGCGGCCTCAGGTGGTTCAGGAGTACGAGAATGGCAAGGCGGTACCGAATCAG |  |
| PRUAV029737 |  | TCTGGCGAAGAAGTTGAGCCAGGCCGACCTGGCCAAGCGGATCAACGAGCGGCCTCAGGTGGTTCAGGAGTACGAGAATGGCAAGGCGGTACCGAATCAG |  |
| PavMBF1C_‘Regina’ |  | TCTGGCGAAGAAGTTGAGCCAGGCCGACCTGGCCAAGCGGATCAACGAGCGGCCTCAGGTGGTTCAGGAGTACGAGAATGGCAAGGCGGTACCGAATCAG |  |
| PavMBF1C_‘Lapins’ |  | TCTGGCGAAGAAGTTGAGCCAGGCCGACCTGGCCAAGCGGATCAACGAGCGGCCTCAGGTGGTTCAGGAGTACGAGAATGGCAAGGCGGTACCGAATCAG |  |
| PavMBF1C_‘Garnet’ |  | TCTGGCGAAGAAGTTGAGCCAGGCCGACCTGGCCAAGCGGATCAACGAGCGGCCTCAGGTGGTTCAGGAGTACGAGAATGGCAAGGCGGTACCGAATCAG |  |
|  |  |  |  |
|  |  | ....|....|....|....|....|....|....|....|....|....|....|....|....|....|....|....|....|....|....|....| |  |
| ppa013016m |  | GCCGTGCTCTCCAAGATGGAGAGGATTCTGGAAGTGAAGCTCAGGGGCAAAGTTGG~CAAATAAAAATAGTACTGTGAAAAGCTAAAAA~CGCT~GACAT |  |
| PRUAV029737 |  | GCCGTGCTCTCCAAGATGGAGAGGGTTCTGGAAGTGAAGCTCAGGGGCAAAGTTGGACAAATAAAATTAGTACTGTGAAAAGCTAAAAAACGCTAGACGT |  |
| PavMBF1C_‘Regina’ |  | GCCGTGCTCTCCAAGATGGAGAGGGTCCTTGGAAGTGA |  |
| PavMBF1C_‘Lapins’ |  | GCCGTGCTCTCTAAGATGGAGAGGTTCCTTGGAAGTGA |  |
| PavMBF1C_‘Garnet’ |  | GCCGTGCTCTCTAAGATGGAGAGGGTCCTTGGAAGTGA |  |

***MMP***

|  |  | ....|....|....|....|....|....|....|....|....|....|....|....|....|....|....|....|....|....|....|....| |  |
| --- | --- | --- | --- |
| ppa007227m | 400 | TCCCAATTTGCCCTCCAATTTCACCGACGACTTCGACGACGACCTCCAATCAGCTCTCAAAACCTACCAGAAGAACTTCAATCTCAACGTCACCGGCGAG |  |
| PRUAV015398 |  | TCCCAATTTGCCCTCCAATTTCACCGACGACTTCGACGACGACCTCCAATCAGCTCTCAGAACCTACCAGAAGAACTTCAATCTCAACATCACCGGCGAG |  |
| PRUAV026734 |  | ~~~~~~~~~~~~~~~~~~~~~~~~~~~~~~~~~~~~~~~~~~~~~~~~~~~~~~~~~~~~~~~~~~~~~~~~~~~~~~~~~~~~~~~~~~~~~~~~~~~~ |  |
| PavMMP_‘Regina’ |  | ~~~~~~~~~~~~~~~~~~~~~~~~~~~~~~~~~~~~~~~~~~~aggatcagctctcatagctacct~~~gaagaTCATCAATCTCAACGTCACCGGCGAG |  |
| PavMMP_‘Lapins’ |  | ~~~~~~~~~~~~~~~~~~~~~~~~~~~~~~~~~~~~~~~~~~~gtcaggctctcagacc~~tACCT~~~GAAGATCTTCAATCTCAACGTCACCGGCGAG |  |
| PavMMP_‘Garnet’ |  | ~~~~~~~~~~~~~~~~~~~~~~~~~~~~~~~~~~~~~~~~~~~taggctctcagagct~~~gaCCT~~~GAAGATCATCAATCTCAACATCACCGGCGAG |  |
|  |  |  |  |
|  |  | ....|....|....|....|....|....|....|....|....|....|....|....|....|....|....|....|....|....|....|....| |  |
| ppa007227m |  | CTAGACGAGCGCACCATCCAGCACCTCGTCAAGCGTCGATGCGGCAATCCCGATATAGTCAACGGCACCACCACCATGAATTCCGGCAAACCGACGTCGT |  |
| PRUAV015398 |  | CTAGACCAGCCCACCATCCAGCACCTCGTCAAGCCTCGATGCGGCAATCCCGATATAGTCAATGGCACCACGACCATGAATTCCGGCAAACCGACGTCGT |  |
| PRUAV026734 |  | ~~~~~~~~~~~~~~~~~~~~~~~~~~~~~~~~~~~~~~~~~~~~~~~~~~~~~~~~~~~~~~~~~~~~~~~~~~~~~~~~~~~~~~~~~~~~~~~~~~~~ |  |
| PavMMP_‘Regina’ |  | CTAGACCAGCCCACCATCCAGCACCTCGTCAAGCCTCGATGCGGCAATCCCGATATAGTCAATGGCACCACGACCATGAATTCCGGCAAACCGACGTCGT |  |
| PavMMP_‘Lapins’ |  | CTAGACCAGCCCACCATCCAGCACCTCGTCAAGCCTCGATGCGGCAATCCCGATATAGTCAATGGCACCACGACCATGAATTCCGGCAAACCGACGTCGT |  |
| PavMMP_‘Garnet’ |  | CTAGACCAGCCCACCATCCAGCACCTCGTCAAGCCTCGATGCGGCAATCCCGATATAGTCAATGGCACCACGACCATGAATTCCGGCAAACCGACGTCGT |  |
|  |  |  |  |
|  |  | ....|....|....|....|....|....|....|....|....|....|....|....|....|....|....|....|....|....|....|....| |  |
| ppa007227m |  | CTAACACGTCCAATCTCCACACGGTTGCTCACTACTCTACTTTCCCGGGGACGCCGGTCTGGCCGCCCAGCAGGCGCGACTTGACCTACGCATTCTTGCC |  |
| PRUAV015398 |  | CTAACACCTCCAATCTCCACACGGTT |  |
| PRUAV026734 |  | ~~~~~~~~~~~~~~~~~~~~~~~~~~~~~~~~~~~~~~~~~~~~~~~~~~~~~~~~~~~~~~~~~~~~~~~~~~~~~~~~~~~~~~~~~~~~~~~~~~~~ |  |
| PavMMP_‘Regina’ |  | CTAACACCTCCAATCTCCACACGGTTGCTCACTACTCTACTTTCCCGGGGACGCCGGTCTGGCCGCCGAGCCGGCTCGACTTGACCTACGCATTCTCGCC |  |
| PavMMP_‘Lapins’ |  | CTAACACCTCCAATCTCCACACGGTTGCTCACTACTCTACTTTCCCGGGGACGCCGGTCTGGCCGCCGAGCCGGCTCGACTTGACCTACGCATTCTCGCC |  |
| PavMMP_‘Garnet’ |  | CTAACACCTCCAATCTCCACACGGTTGCTCACTACTCTACTTTCCCGGGGACGCCGGTCTGGCCGCCGAGCCGGCTCGACTTGACCTACGCATTCTCGCC |  |
|  |  |  |  |
|  |  | ....|....|....|....|....|....|....|....|....|....|....|....|....|....|....|....|....|....|....|....| |  |
| ppa007227m |  | GGAGAACAAGCTTGCCGACGACGTCAAGGCAGTGTTTTTACGGGCCTTCCAGAGGTGGTCGGCAGCGACGCCGTTGAATTTCTCCGAGACGACGTCGTTC |  |
| PRUAV015398 |  |  |  |
| PRUAV026734 |  | ~~~~~~~~~~~~~~~~~~~~~~~~~~~~~~~~~~~~~~~~~~~~~~~~~~~~~~~~~~~~~~~~~~~~~~~~~~~~~~~~~~~~~~~~~~~~~~~~~~~~ |  |
| PavMMP_‘Regina’ |  | GGAGAACAATCTTTCCGACGAGACCAAGGCAGTGTTTTTACGGGCTTTCCAGCGGTGGTCGGCGGCGACGCCGTTGAATTTCTCCGAGACGACGTCGTTC |  |
| PavMMP_‘Lapins’ |  | GGAGAACAATCTTTCCGACGAGACCAAGGCAGTGTTTTTACGGGCTTTCCAGCGGTGGTCGGCGGCGACGCCGTTGAATTTCTCCGAGACGACGTCGTTC |  |
| PavMMP_‘Garnet’ |  | GGAGAACAATCTTTCCGACGAGACCAAGGCAGTGTTTTTACGGGCTTTCCAGCGGTGGTCGGCGGCGACGCCGTTGAATTTCTCCGAGACGACGTCGTTC |  |
|  |  |  |  |
|  |  | ....|....|....|....|....|....|....|....|....|....|....|....|....|....|....|....|....|....|....|....| |  |
| ppa007227m |  | TACACGGCGGATATCAAGATTGGGTTCTTCAGTGGCGATCACGGGGACGGAGAGCCGTTCGATGGGGTTTTGGGGACGCTGGCGCACGCGTTCTCGCCGC |  |
| PRUAV015398 |  |  |  |
| PRUAV026734 |  | ~~~~~~~~~~~~~~~~~~~~~~~~~~~~~~~~~~~~~~~~~~~~~~~~~~~~~~~~~~~~~~~~~~~~~~~~~~~~~~~~~~~GCACGCGTTCTCGCCGC |  |
| PavMMP_‘Regina’ |  | TACACGGCGGATATCAAGATTGGGTTCTTCAGAGGCGATCACGGGGACGGAGAGCCGTTCGATGGGGTTTTGGGGACGCTGGCGCACGCGTTCTCGCCGC |  |
| PavMMP_‘Lapins’ |  | TACACGGCGGATATCAAGATTGGGTTCTTCAGAGGCGATCACGGGGACGGAGAGCCGTTCGATGGGGTTTTGGGGACGCTGGCGCACGCGTTCTCGCCGC |  |
| PavMMP_‘Garnet’ |  | TACACGGCGGATATCAAGATTGGGTTCTTCAGAGGCGATCACGGGGACGGAGAGCCGTTCGATGGGGTTTTGGGGACGCTGGCGCACGCGTTCTCSCCGC |  |
|  |  |  |  |
|  |  | ....|....|....|....|....|....|....|....|....|....|....|....|....|....|....|....|....|....|....|....| |  |
| ppa007227m |  | CCAGCGGGAGGTTCCACTTGGACCTTGACGAGAACTGGGTGATCACGGGTGACATTAGCACGTCGTCCGTGACGTCAGCGGTCGACCTGGAGTCGGTGGC |  |
| PRUAV015398 |  |  |  |
| PRUAV026734 |  | CGAGCGGGAGGTTCCACTTGGACGCTGACGAGAACTGGGTGATCACGGGTGACATTAGCACGTCGTCCGTGACGTCAGCGGTCGACCTGGAGTCGGTGGC |  |
| PavMMP_‘Regina’ |  | CGAGCGGGAGGTTCCACTTGGACGCTGACGAGAACTGGGTGATCACGGGTGACATTAGCACGTCGTCCGTGACGTCAGCGGTCGACCTGGAGTCGGTGGC |  |
| PavMMP_‘Lapins’ |  | CGAGCGGGAGGTTCCACTTGGACGCTGACGAGAACTGGGTGATCACGGGTGACATTAGCACGTCGTCCGTGACGTCAGCGGTCGACCTGGAGTCGGTGGC |  |
| PavMMP_‘Garnet’ |  | CGAGCGGGAGGTTCCACTTGGACGCTGACGAGAACTGGGTGATCACGGGTGACATTAGCACGTCGTCCGTGACGTCAGCGGTCGACCTGGAGTCGGTGGC |  |
|  |  |  |  |
|  |  | ....|....|....|....|....|....|....|....|....|....|....|....|....|....|....|....|....|....|....|....| |  |
| ppa007227m |  | GGTTCACGAGATTGGGCATCTGCTGGGGTTGGGCCACTCGTCGGTTGAGGAGGCGATTATGTTCCCGACGATCTCGTCGCGGACGAAGAAAGTGGAGCTG |  |
| PRUAV015398 |  |  |  |
| PRUAV026734 |  | GGTTCACGAGATTGGGCATCTGCTGGGGTTGGGCCACTCGTCGGTTGAGGAGGCGATTATGTTCCCGACGATCTCGTCGCGGACGAAGAAAGTGGAACTG |  |
| PavMMP_‘Regina’ |  | GGTTCACGAGATTGGGCATCTGCTGGGGTTGGGCCACTCGTCGGTTGAGGAGGCGATTATGTTCCCGACGATCTCGTCGCGGACGAAGAAAGTGGAACTG |  |
| PavMMP_‘Lapins’ |  | GGTTCACGAGATTGGGCATCTGCTGGGGTTGGGCCACTCGTCGGTTGAGGAGGCGATTATGTTCCCGACGATCTCGTCGCGGACGAAGAAAGTGGAACTG |  |
| PavMMP_‘Garnet’ |  | GGTTCACGAGATTGGGCATCTGCTGGGGTTGGGCCACTCGTCGGTTGAGGAGGCGATTATGTTCCCGACGATCTCGTCGCGGACGAAGAAAGTGGAACTG |  |
|  |  |  |  |
|  |  | ....|....|....|....|....|....|....|....|....|....|....|....|....|....|....|....|....|....|....|....| |  |
| ppa007227m |  | GCGAGCGACGACGTTTTGGGGATTCAGTCGCTGTACGGCGCCAATCCCAGTTACGACGGTACGAGTGGTTCGTCTACGACGTCCACTCAAGCACGGGAAA |  |
| PRUAV015398 |  |  |  |
| PRUAV026734 |  | GCGAGCGACGACGTTTTGGGGATTCAGTCGCTGTACGGCGCCAATCCCAGTTACGACGGTCCGAGTGGTTCGTCTACGCCGTCCACTCAAGCACGGGAAA |  |
| PavMMP_‘Regina’ |  | GCGAGCGACGACGTTTTGGGGATTCAGTCGCTGTACGGCGCCAATCCCAGTTACGACGGTCCGAGTGGTTCGTCTACGCCGTCCACTCAAGCACGGGAAA |  |
| PavMMP_‘Lapins’ |  | GCGAGCGACGACGTTTTGGGGATTCAGTCGCTGTACGGCGCCAATCCCAGTTACGACGGTCCGAGTGGTTCGTCTACGCCGTCCACTCAAGCACGGGAA~ |  |
| PavMMP_‘Garnet’ |  | GCGAGCGACGACGTTTTGGGGATTCAGTCGCTGTACGGCGCCAATCCCAGTTACGACGGTCCGAGTGGTTCGTCTACGCCGTCCACTCAAGCACGGGAAA |  |
|  |  |  |  |
|  |  | ....|....|....|....|....|....|....|....|....|....|....|....|....|....|....|....|....|....|....|....| |  |
| ppa007227m |  | CGTCCGCTGCTGGGGACCACCTCACTTCCGCTCCAAGGTTGTGGGGCCTCAGTGCCTTGCTGGCCGTTGGATTTCTGTTATTGTCGTTTTAGTTTTTTCT |  |
| PRUAV015398 |  |  |  |
| PRUAV026734 |  | CGTCCGCTGCTGGGGACCACCTCACTTCCGCTCCAAGGTTGTGGGGCCTCAGTGCCTTGCTGGCCGTTGGATTTTTGTTATTGTCGTTTTAGTTTTTGTC |  |
| PavMMP_‘Regina’ |  | CGTCCGCTGCTGGGGACCACCTCACTTCCGCTCCAAGGTTGTGGGGCCTCAGTGCCTTGCTGGCCGTTGGATTTTTGTTATTGTCGTTTTAGTTTTTTGT |  |
| PavMMP_‘Lapins’ |  | CGTCCGCTGCTGGGGACCACCTCACTTCCGCTCCAAGGTTGTGGGGCCTCAGTGCCTTGCTGGCCGTTGGATTTTTGTTATTGTCGTTTTAGTTTTTTGT |  |
| PavMMP_‘Garnet’ |  | CGTCCSCTGCTGGGGACCACCTCACTTCCGCTCCAAGGTTGTGGGGCCTCAGTGCCTTGCTGGCCGTTGGATTTTTGTTATTGTCGTTTTAGTTTTTTGT |  |
|  |  |  |  |
|  |  | ....|....|....|....|....|....|....|....|....|....|....|....|....|....|....|....|....|....|....|....| |  |
| ppa007227m |  | CATATTGTTTGGTTTTATTGATTTTAAAGTTAGATTTAGCTTAGATTGTTTTATTATTTATAATTTTTTGGGGTAATTATTGGGGATTTGGCACTCTACT |  |
| PRUAV015398 |  |  |  |
| PRUAV026734 |  | TATTATTTTTTGGTTTTATTGTATTAAAGTTAGATTTAGCTTATATTGTTTTATTATTTATAATTTTTTGGGGTAATTATTGGGGATTTGGCACTCTACT |  |
| PavMMP_‘Regina’ |  | CATATTTTTTGGTTTTATTGATTAAAGTTAGATTAGCTTATATTGTTTTATTATTTATAATTTTTTGGGGTAATTATTGGTATATTgtgtcgacatctaa |  |
| PavMMP_‘Lapins’ |  | CATATTTTTTGGTTTTATTGATTAAAGTTAGATTAGCTTATATTGTTTTATTATTTATAATTTTTTGGGGTAATTATTGGTATGTtttgggcactcatca |  |
| PavMMP_‘Garnet’ |  | CATATTTTTTGGTTTTATTGATTAAAGTTAGATTAGCTTATATTGTTTTATTATTTATAATTATTTTATGAGTATTTttgtttatatgttgcgacgatca |  |
|  |  |  |  |
|  |  | ....|....|....|....|....|....|....|....|....|....|....|....|....|....|....|....|....|....|....|....| |  |
| ppa007227m |  | GACAGGCCATATCTTAC~~TTACTT~~~~~~~~GTATGATACTTATTTTAAATTTTATTTTTGTAGGATTTTTTTATTTTATTTTTGGTATTCAGATACA |  |
| PRUAV015398 |  |  |  |
| PRUAV026734 |  | GACAGGCCGTATCTTACAGTTACTTACTTACTTGTATGATTCT~~~GCCA~~~~~~~~~~~~GTAGGGTCTT~~~ATTTTATTTTTGGTATTCAGATACA |  |
| PavMMP_‘Regina’ |  | ca |  |
| PavMMP_‘Lapins’ |  |  |  |
| PavMMP_‘Garnet’ |  | cttggggcctcactaca |  |

***MSI1***

|  |  | ....|....|....|....|....|....|....|....|....|....|....|....|....|....|....|....|....|....|....|....| |  |
| --- | --- | --- | --- |
| ppa006205m | 401 | GCTACCATTGGAGGACGCAGAGAACGATGCTCGCCATTATGATGATGATCGAGCTGAGGTTGGCGGTTTTGGTTGCGCCAATGGCAAGGTAAGG~~CTTG |  |
| PRUAV005812 |  | GCTGCCATTAGAGGACGCAGAGAACGATGCTCGCCATTATGATGATGATCGAGCTGAGGTTGGCGGTTTTGGTTGCGCCAATGGCAAGGT~~~~~~~~~~ |  |
| PavMSI1_‘Regina’ |  | ~~~~~~~~~~~~~~~~~~~~~~~~~~~~~~~~~~~~~~~~~~~~~~~~~~~~~~~AGGATTGGCGGTTTTGGTTGCGCCAATGGCAAGGTAAGGGGCTTG |  |
| PavMSI1_‘Lapins’ |  | ~~~~~~~~~~~~~~~~~~~~~~~~~~~~~~~~~~~~~~~~~~~~~~~~~~~~AGGCGAAGTGGCGGTTTTGGTTGCGCCAATGGCAAGGTAAGGGGCTTG |  |
| PavMSI1_‘Garnet’ |  | ~~~~~~~~~~~~~~~~~~~~~~~~~~~~~~~~~~~~~~~~~~~~~~~~~~~~CTACGAGTTGGCGGTTTTGGTTGCGCCAATGGCAAGGTAAGGGGCTTG |  |
|  |  |  |  |
|  |  | ....|....|....|....|....|....|....|....|....|....|....|....|....|....|....|....|....|....|....|....| |  |
| ppa006205m |  | ATTTTGTGGTCTTAAGTTGTTGATTTTTCGGATAGGGT~~~~~~~~~~~~~~~~~~GGAATATAAGCAGTTCATACTGATGTGCTAAAGATTTCGAATTT |  |
| PRUAV005812 |  | ~~~~~~~~~~~~~~~~~~~~~~~~~~~~~~~~~~~~~~~~~~~~~~~~~~~~~~~~~~~~~~~~~~~~~~~~~~~~~~~~~~~~~~~~~~~~~~~~~~~~ |  |
| PavMSI1_‘Regina’ |  | GTTTTGTGGTCTCAAGTTGTTGATTTTTCGGATAGGGTTTAAGAATTTCATATTAAGGAATATAAGCAGTTCATACTGATGTGCTAAAGATTTCGAATTT |  |
| PavMSI1_‘Lapins’ |  | GTTTTGTGGTCTCAAGTTGTTGATTTTTCGGATAGGGTTTAAGAATTTCATATTAAGGAATATAAGCAGTTCATACTGATGTGCTAAAGATTTCGAATTT |  |
| PavMSI1_‘Garnet’ |  | GTTTTGTGGTCTCAAGTTGTTGATTTTTCGGATAGGGTTTAAGAATTTCATATTAAGGAATATAAGCAGTTCATACTGATGTGCTAAAGATTTCGAATTT |  |
|  |  |  |  |
|  |  | ....|....|....|....|....|....|....|....|....|....|....|....|....|....|....|....|....|....|....|....| |  |
| ppa006205m |  | GGGTATTTTGGAATGCTGGCCTGTTTGGTTGTAAAGATAGTGCTGGAAAATACAAGAAATTTTGAATTTTATTTATTTTTATACATTTTTATCAATTTAT |  |
| PRUAV005812 |  | ~~~~~~~~~~~~~~~~~~~~~~~~~~~~~~~~~~~~~~~~~~~~~~~~~~~~~~~~~~~~~~~~~~~~~~~~~~~~~~~~~~~~~~~~~~~~~~~~~~~~ |  |
| PavMSI1_‘Regina’ |  | GGGTATTTTGGAATGCTGGCCTGTTTCATTGTAAAGATAGTGCTGGACAATACAAGAAATTTTGAATTTTATTTATTTTTATACATTTTTATCAATTTAT |  |
| PavMSI1_‘Lapins’ |  | GGGTATTTTGGAATGCTGGCCTGTTTCATTGTAAAGATAGTGCTGGACAATACAAGAAATTTTGAATTTTATTTATTTTTATACATTTTTATCAATTTAT |  |
| PavMSI1_‘Garnet’ |  | GGGTATTTTGGAATGCTGGCCTGTTTCATTGTAAAGATAGTGCTGGACAATACAAGAAATTTTGAATTTTATTTATTTTTATACATTTTTATCAATTTAT |  |
|  |  |  |  |
|  |  | ....|....|....|....|....|....|....|....|....|....|....|....|....|....|....|....|....|....|....|....| |  |
| ppa006205m |  | TAGAAATAAGAAGTACTGGCACTTCAATCACATTGTAGAAACTTGAAAGAAAGTGGGAAATTTTT~GTTTTTTGTTTTTGTTTTGGTCATTCTAAGAATT |  |
| PRUAV005812 |  | ~~~~~~~~~~~~~~~~~~~~~~~~~~~~~~~~~~~~~~~~~~~~~~~~~~~~~~~~~~~~~~~~~~~~~~~~~~~~~~~~~~~~~~~~~~~~~~~~~~~~ |  |
| PavMSI1_‘Regina’ |  | TAGAAATAAGAAGTACTGGCACTTCAATCACATTGTAGAAACTTGAAAGAAAGTGGGAAATTTTTTGTTTT~~~~~~~~~~~~~GGTCATTCTAAAGATT |  |
| PavMSI1_‘Lapins’ |  | TAGAAATAAGAAGTACTGGCACTTCAATCACATTGTAGAAACTTGAAAGAAAGTGGGAAATTTTTTGTTTT~~~~~~~~~~~~~GGTCATTCTAAAGATT |  |
| PavMSI1_‘Garnet’ |  | TAGAAATAAGAAGTACTGGCACTTCAATCACATTGTAGAAACTTGAAAGAAAGTGGGAAATTTTTTGTTTT~~~~~~~~~~~~~GGTCATTCTAAAGATT |  |
|  |  |  |  |
|  |  | ....|....|....|....|....|....|....|....|....|....|....|....|....|....|....|....|....|....|....|....| |  |
| ppa006205m |  | TGATTTATAACAAAACATGTTCTGTGATAAGCACATAGCTTGAATTGAAGAAAGAATGAAATAGAAGGGATATTTGGTGTCTCCAATATGGAGAAAACAC |  |
| PRUAV005812 |  | ~~~~~~~~~~~~~~~~~~~~~~~~~~~~~~~~~~~~~~~~~~~~~~~~~~~~~~~~~~~~~~~~~~~~~~~~~~~~~~~~~~~~~~~~~~~~~~~~~~~~ |  |
| PavMSI1_‘Regina’ |  | TGATTTATACCAAAACATGTTCTATGATAAGTACATAGCTTGAATTGAAGAAAGAATGAAATAGAAGGGATATTTGGTGTCTCCAATATGGAGAAAACAC |  |
| PavMSI1_‘Lapins’ |  | TGATTTATACCAAAACATGTTCTATGATAAGTACATAGCTTGAATTGAAGAAAGAATGAAATAGAAGGGATATTTGGTGTCTCCAATATGGAGAAAACAC |  |
| PavMSI1_‘Garnet’ |  | TGATTTATACCAAAACATGTTCTATGATAAGTACATAGCTTGAATTGAAGAAAGAATGAAATAGAAGGGATATTTGGTGTCTCCAATATGGAGAAAACAC |  |
|  |  |  |  |
|  |  | ....|....|....|....|....|....|....|....|....|....|....|....|....|....|....|....|....|....|....|....| |  |
| ppa006205m |  | ACCAGCTTTTTCAATTGTGTCCCAATTTGACCCGATGAGGGAGGAATGCACTTACTTGAAGTAGAAAGTACTAACAGCTACCTAGAATCACTGAAACTAA |  |
| PRUAV005812 |  | ~~~~~~~~~~~~~~~~~~~~~~~~~~~~~~~~~~~~~~~~~~~~~~~~~~~~~~~~~~~~~~~~~~~~~~~~~~~~~~~~~~~~~~~~~~~~~~~~~~~~ |  |
| PavMSI1_‘Regina’ |  | ACCAGCTTTTTCAATTGTGTCCCAAGTTGACCTGATGAGGGAGGAATGCACTTACTTGAAGTAGAAAGTACTAACAGCTACCTAGAATCACTGAAACTAA |  |
| PavMSI1_‘Lapins’ |  | ACCAGCTTTTTCAATTGTGTCCCAAGTTGACCTGATGAGGGAGGAATGCACTTACTTGAAGTAGAAAGTACTAACAGCTACCTAGAATCACTGAAACTAA |  |
| PavMSI1_‘Garnet’ |  | ACCAGCTTTTTCAATTGTGTCCCAAGTTGACCTGATGAGGGAGGAATGCACTTACTTGAAGTAGAAAGTACTAACAGCTACCTAGAATCACTGAAACTAA |  |
|  |  |  |  |
|  |  | ....|....|....|....|....|....|....|....|....|....|....|....|....|....|....|....|....|....|....|....| |  |
| ppa006205m |  | CAACAATAATGTAATGAGGCAATTGGTTTATGCTCTGGTTTGGATTCATGAGCTGTATCGCTGACAGTATGGCACTTTAATAACTGAAGTAAAACACTGA |  |
| PRUAV005812 |  | ~~~~~~~~~~~~~~~~~~~~~~~~~~~~~~~~~~~~~~~~~~~~~~~~~~~~~~~~~~~~~~~~~~~~~~~~~~~~~~~~~~~~~~~~~~~~~~~~~~~~ |  |
| PavMSI1_‘Regina’ |  | CAACAATAATGTAATGAGGCAATTGGTTTGACCTCTGGTTTGGATTCATGAGCTGCATCGCTGACAGTATGGCACTTTAATAACTGAAGTAAAACACTGC |  |
| PavMSI1_‘Lapins’ |  | CAACAATAATGTAATGAGGCAATTGGTTTGACCTCTGGTTTGGATTCATGAGCTGCATCGCTGACAGTATGGCACTTTAATAACTGAAGTAAAACACTGC |  |
| PavMSI1_‘Garnet’ |  | CAACAATAATGTAATGAGGCAATTGGTTTGACCTCTGGTTTGGATTCATGAGCTGCATCGCTGACAGTATGGCACTTTAATAACTGAAGTAAAACACTGC |  |
|  |  |  |  |
|  |  | ....|....|....|....|....|....|....|....|....|....|....|....|....|....|....|....|....|....|....|....| |  |
| ppa006205m |  | CCCTTGCCGTATGCTGTAGTTGTTTCTATCATTCTATGTCTAAACGGGCTCAAGTATATGGTCTTAAATTTTCTTGCAGGTGCAAATAATCCAGCAAATA |  |
| PRUAV005812 |  | ~~~~~~~~~~~~~~~~~~~~~~~~~~~~~~~~~~~~~~~~~~~~~~~~~~~~~~~~~~~~~~~~~~~~~~~~~~~~~~~~~GCAAATAATCCAGCAAATA |  |
| PavMSI1_‘Regina’ |  | CCCTTGGCATATGCTGTAGTTGTTTCTATCATTCAATGTCTAAACGGGCTCAAGTATATGGTCTTAAATTTTCTTGCAGGTGCAAATAATCCAGCAAATA |  |
| PavMSI1_‘Lapins’ |  | CCCTTGGCATATGCTGTAGTTGTTTCTATCATTCAATGTCTAAACGGGCTCAAGTATATGGTCTTAAATTTTCTTGCAGGTGCAAATAATCCAGCAAATA |  |
| PavMSI1_‘Garnet’ |  | CCCTTGGCATATGCTGTAGTTGTTTCTATCATTCAATGTCTAAACGGGCTCAAGTATATGGTCTTAAATTTTCTTGCAGGTGCAAATAATCCAGCAAATA |  |
|  |  |  |  |
|  |  | ....|....|....|....|....|....|....|....|....|....|....|....|....|....|....|....|....|....|....|....| |  |
| ppa006205m |  | AATCATGATGGAGAGGTTAATCGAGCTCGTTATATGCCTCAGAATCCATTTATTATTGCGACAAAGACCGTCAATGCTGAAGTTTTTGTGTTCGATTATA |  |
| PRUAV005812 |  | AATCATGATGGAGAGGTTAATCGGGCTCGTTATATGCCTCAGAATCCATTTATTATTGCCACAAAGACCGTCAATGCTGAAGTTTTTGTGTTCGATTATA |  |
| PavMSI1_‘Regina’ |  | AATCATGATGGAGAGGTTAATCGGGCTCGTTATATGCCTCAGAATCCATTTATTATTGCAAAAAAACACGCtgcccaa |  |
| PavMSI1_‘Lapins’ |  | AATCATGATGGAGAGGTTAATCGGGCTCGTTATATGCCTCAGAATCCATTTATTATTGCAAAAAAACCCCaaaaa |  |
| PavMSI1_‘Garnet’ |  | AATCATGATGGAGAGGTTAATCGGGCTCGTTATATGCCTCAGAATCCATTTATTATTGCCAAAAAAACCCGtcaaa |  |

***NUA***

|  |  | ....|....|....|....|....|....|....|....|....|....|....|....|....|....|....|....|....|....|....|....| |  |
| --- | --- | --- | --- |
| Ppa000061m | 20401 | TTGATGTCGCTGAGTTGCCTCAAGGTTCAAATGAGGAAGCTGTTGGTGATACTGAGAAGGAAGAAATTGAGACTACGGGAGAGAAGGTTGAAGAGCCAAA |  |
| PRUAV002346 |  | ttgatgttgctgagttgcctcaaggttcaaatgaggaagctgttggtgatactgagaaggaagaaattgagactacaggagagaaggtggaagagccaaa |  |
| PavNUA_‘Regina’ |  | ~~~~~~~~~~~~~~~~~~~~~~~~~~~~~~~~~~cga~~~~~~tggTG~TACTGAGA~GGA~GAA~TTGAGACTACGGGAGAGAAGGTGGAAGAGCCAAA |  |
| PavNUA_‘Lapins’ |  | ~~~~~~~~~~~~~~~~~~~~~~~~~~~~~~~tgc~~~~gc~~~tggTG~TACTGAGA~GGA~GAA~TTGAGACTACGGGAGAGAAGGTGGAAGAGCCAAA |  |
| PavNUA_‘Garnet’ |  | ~~~~~~~~~~~~~~~~~~~~~~~~~~~~~~~~~ccga~~~~~tgGGTGA~ACTGAGA~GGA~GAA~TTGAGACTACGGGAGAGAAGGTGGAAGAGCCAAA |  |
|  |  |  |  |
|  |  | ....|....|....|....|....|....|....|....|....|....|....|....|....|....|....|....|....|....|....|....| |  |
| Ppa000061m |  | TGAACGACAGTTTGATGGTTCAAATCAAGTAGAATCACAACCCGACAAGCATATTGGTTTAGAAGAAAATGTTGATGGATCAGGTGGAACTGAGATGATG |  |
| PRUAV002346 |  | tgaacgacagtttgatggttcaaatcaagtagaatcacaacctgacaagcatattggtttagaagaaaatgttgatggatcaggtggaactgagatgatg |  |
| PavNUA_‘Regina’ |  | TGAACGACAGTTTGATGGTTCAAATCAAGTAGAATCACAACCTGACAAGCATATTGGTYTAGAAGAAAATGTTGATGGATCAGGTGGAACTGAGATGATG |  |
| PavNUA_‘Lapins’ |  | TGAACGACAGTTTGATGGTTCAAATCAAGTAGAATCACAACCTGACAAGCATATTGGTYTAGAAGAAAATGTTGATGGATCAGGTGGAACTGAGATGATG |  |
| PavNUA_‘Garnet’ |  | TGAACGACAGTTTGATGGTTCAAATCAAGTAGAATCACAACCTGACAAGCATATTGGTCTAGAAGAAAATGTTGATGGATCAGGTGGAACTGAGATGATG |  |
|  |  |  |  |
|  |  | ....|....|....|....|....|....|....|....|....|....|....|....|....|....|....|....|....|....|....|....| |  |
| Ppa000061m |  | TGTGATGATGGAGCCAAGGATCAGGTTGAGCTGGACAACCAACAGACCAATGAATTTGGAGGAGACAGAGAAGAGGGAGAGTTGGTCCCTGATGTTTCTG |  |
| PRUAV002346 |  | tgtgatgatggagccaaggatcaggttgagctggacaaccaacagtccaatgaatttggaggagacagagaagagggagagttggtccctgatgtttctg |  |
| PavNUA_‘Regina’ |  | TGTGATGATGGAGCCAAGGATCAGGTTGAGCTGGACAACCAACAGTCCAATGAATTTGGAGGAGACAGAGAAGAGGGAGAGTTGGTCCCTGATGTTTCTG |  |
| PavNUA_‘Lapins’ |  | TGTGATGATGGAGCCAAGGATCAGGTTGAGCTGGACAACCAACAGTCCAATGAATTTGGAGGAGACAGAGAAGAGGGAGAGTTGGTCCCTGATGTTTCTG |  |
| PavNUA_‘Garnet’ |  | TGTGATGATGGAGCCAAGGATCAGGTTGAGCTGGACAACCAACAGTCCAATGAATTTGGAGGAGACAGAGAAGAGGGAGAGTTGGTCCCTGATGTTTCTG |  |
|  |  |  |  |
|  |  | ....|....|....|....|....|....|....|....|....|....|....|....|....|....|....|....|....|....|....|....| |  |
| Ppa000061m |  | AGCTTGAAGGGGGTGATACAATTGGCAGTCCCGAAATAGGGGAAGGTCAACCTGAGCCTGTTGCAACTCCTGGGGCTTCCCCAGCCAGGGGCGATGATGA |  |
| PRUAV002346 |  | agcttgaaggggctgatacaattggcagtcctgaaataggggaaggtcaacctgagcccgttgcaactcctggggcttccccagccaggggcgatgatga |  |
| PavNUA_‘Regina’ |  | AGCTTGAAGGGGGTGATACAATTGGCAGTCCTGAAATAGGGGAAGGTCAACCTGAGCCTGTTGCAACTCCTGGGGCTTCCCCAGCCAGGGGTGATGATGA |  |
| PavNUA_‘Lapins’ |  | AGCTTGAAGGGGGTGATACAATTGGCAGTCCTGAAATAGGGGAAGGTCAACCTGAGCCCGTTGCAACTCCTGGGGCTTCCCCAGCCAGGGGCGATGATGA |  |
| PavNUA_‘Garnet’ |  | AGCTTGAAGGGGGTGATACAATTGGCAGTCCTGAAATAGGGGAAGGTCAACCTGAGCCTGTTGCAACTCCTGGGGCTTCCCCAGCCAGGGGTGATGATGA |  |
|  |  |  |  |
|  |  | ....|....|....|....|....|....|....|....|....|....|....|....|....|....|....|....|....|....|....|....| |  |
| Ppa000061m |  | AGGGGTTGCTGCTAGTTCTGTTGTGGATATTGGTGAGGTAAATTCTCCAGAGGTTCTAAATGATGATAAAAATGATGAAGTTGTAACTGAAGAAGCTGCC |  |
| PRUAV002346 |  | aggggttgctgctggttctgttgtggatattggtgaggtaaattctccggaggttctaaatgatgagaaaaatgatgaagttgtaactgaagaagctgcc |  |
| PavNUA_‘Regina’ |  | AGGGGTTGCTGCTGGTTCTGTTGTGGATATTGGTGAGGTAAAATTCTCCGGA |  |
| PavNUA_‘Lapins’ |  | AGGGGTTGCTGCTGGTTCTGTTGTGGATATTGGTGAGGAAAATTCTCCGGA~ |  |
| PavNUA_‘Garnet’ |  | AGGGGTTGCTGCTGGTTCTGTTGTAGATATTGGTGAGGAAAATTCTCCGGA~ |  |

***OTS1***

|  |  | ....|....|....|....|....|....|....|....|....|....|....|....|....|....|....|....|....|....|....|....| |  |
| --- | --- | --- | --- |
| ppa022300m | 1401 | GAACTATTTGGATCAAGAAGTCGCCGTTTCAGATATTCCTATTTCAGACAGTATATGGAACCAACTTCCCAATAAAATTGAAGAGAAAAAACTTGCGGTA |  |
| PRUAV030235 |  | ~~~~~~~~~~~~~~~~~~~~~~~~~~~~~~~~~~~~~~~~~~~~~AGACAGTATATGGAACCAACTTCCCAATAAAATTGAAGAGAAAAAACTTGCG~~~ |  |
| PavOTS1_‘Regina’ |  | ~~~~~~~~~~~~~~~~~~~~~~~~~~~~~~~~~~~~~~~~~~~~~~~~~~~~~~~~~~~~~~~~~~~~~~~aatggaagagg~~aaACTGTAGACAAGTA |  |
| PavOTS1_‘Lapins’ |  | ~~~~~~~~~~~~~~~~~~~~~~~~~~~~~~~~~~~~~~~~~~~~~~~~~~~~~~~~~~~~~~~~~~~~accagttgaggagtgcaaacTGTAGAAGAGTA |  |
| PavOTS1_‘Garnet’ |  | ~~~~~~~~~~~~~~~~~~~~~~~~~~~~~~~~~~~~~~~~~~~~~~~~~~~~~~~~~~~~~~~~~~~~~~~~~~~~~~~~~~~aaaactGTAGAAGAGTA |  |
|  |  |  |  |
|  |  | ....|....|....|....|....|....|....|....|....|....|....|....|....|....|....|....|....|....|....|....| |  |
| ppa022300m |  | CATTTTGTCCTCACATGCTTCAAATTTAAATGTTAAGATGCCATTAATACTATTTTGAACTGTTTTTATGCCCCATATTTCATATCCAGTTTGCATATAA |  |
| PRUAV030235 |  | ~~~~~~~~~~~~~~~~~~~~~~~~~~~~~~~~~~~~~~~~~~~~~~~~~~~~~~~~~~~~~~~~~~~~~~~~~~~~~~~~~~~~~~~~~~~~~~~~~~~~ |  |
| PavOTS1_‘Regina’ |  | CATTTTGTCCTCACATGCTTCAAATTTAAATGTTAAGATGCCATTAATACTATTTTGAACTGTTTTTATGCCCCATATTACATATCCAATTTGCATATAA |  |
| PavOTS1_‘Lapins’ |  | CATTTTGTCCTCACATGCTTCAAATTTAAATGTTAAGATGCCATTAATACTATTTTGAACTGTTTTTATGCCCCATATTACATATCCAATTTGCATATAA |  |
| PavOTS1_‘Garnet’ |  | CATTTTGTCCTCACATGCTTCAAATTTAAATGTTAAGATGCCATTAATACTATTTTGAACTGTTTTTATGCCCCATATTACATATCCAATTTGCATATAA |  |
|  |  |  |  |
|  |  | ....|....|....|....|....|....|....|....|....|....|....|....|....|....|....|....|....|....|....|....| |  |
| ppa022300m |  | TTATCAGAATTGCCATGAAATGTGCCCTATTCTTACTCCACAAAAGAC~~ATATTCTTGAGTTTTGTTCCTCCAAATACTTCTGTTTCACTTTGTGTGTT |  |
| PRUAV030235 |  | ~~~~~~~~~~~~~~~~~~~~~~~~~~~~~~~~~~~~~~~~~~~~~~~~~~~~~~~~~~~~~~~~~~~~~~~~~~~~~~~~~~~~~~~~~~~~~~~~~~~~ |  |
| PavOTS1_‘Regina’ |  | TTATCAGAATTGCCATGAAATATGCCTTATTCTTACTCCACGAAAGACACATATTCTTGAGTTTTGTTCCTCCAAATACTTCTGTTTCACTT~GTGTTTT |  |
| PavOTS1_‘Lapins’ |  | TTATCAGAATTGCCATGAAATATGCCTTATTCTTACTCCACGAAAGACACATATTCTTGAGTTTTGTTCCTCCAAATACTTCTGTTTCACTT~GTGTTTT |  |
| PavOTS1_‘Garnet’ |  | TTATCAGAATTGCCATGAAATATGCCTTATTCTTACTCCACGAAAGACACATATTCTTGAGTTTTGTTCCTCCAAATACTTCTGTTTCACTT~GTGTTTT |  |
|  |  |  |  |
|  |  | ....|....|....|....|....|....|....|....|....|....|....|....|....|....|....|....|....|....|....|....| |  |
| ppa022300m |  | CAACTTAATATGTTTGCATTAGAAGCCTTCTAGTTTTTGAACCAATTGAATCTGTTGACGTTGGACC~~~GATATTTAGATTGTTTTCTGGTTTCTGTGT |  |
| PRUAV030235 |  | ~~~~~~~~~~~~~~~~~~~~~~~~~~~~~~~~~~~~~~~~~~~~~~~~~~~~~~~~~~~~~~~~~~~~~~~~~~~~~~~~~~~~~~~~~~~~~~~~~~~~ |  |
| PavOTS1_‘Regina’ |  | CAACTTAAGATGTTTGCATTAGAAGCTTTCTAGTCTTTCAACCAATTGAATCTGTTGACGTTGGACGACCAATATTTAGATTGTTTTCTGGTTTCTGTGT |  |
| PavOTS1_‘Lapins’ |  | CAACTTAAGATGTTTGCATTAGAAGCTTTCTAGTCTTTCAACCAATTGAATCTGTTGACGTTGGACGACCAATATTTAGATTGTTTTCTGGTTTCTGTGT |  |
| PavOTS1_‘Garnet’ |  | CAACTTAAGATGTTTGCATTAGAAGCTTTCTAGTCTTTCAACCAATTGAATCTGTTGACGTTGGACGACCAATATTTAGATTGTTTTCTGGTTTCTGTGT |  |
|  |  |  |  |
|  |  | ....|....|....|....|....|....|....|....|....|....|....|....|....|....|....|....|....|....|....|....| |  |
| ppa022300m |  | GCAGTCTACAAGTATATCTAATTTCTGACAAGTGTTGTGTTAAAATTGTATAACATCATGAAGCTCTTAGAAATTTAGTGTGCCTTATAAGTTGAAATGG |  |
| PRUAV030235 |  | ~~~~~~~~~~~~~~~~~~~~~~~~~~~~~~~~~~~~~~~~~~~~~~~~~~~~~~~~~~~~~~~~~~~~~~~~~~~~~~~~~~~~~~~~~~~~~~~~~~~~ |  |
| PavOTS1_‘Regina’ |  | GCAGTCTACAAGTATATCTAATTTCTGACAAGTGTTGTGTTAAAATTGTATAACATCATAAAGCTCTTAGAAATTTAGTGTGCCTTGTAAGTTGGAATGG |  |
| PavOTS1_‘Lapins’ |  | GCAGTCTACAAGTATATCTAATTTCTGACAAGTGTTGTGTTAAAATTGTATAACATCATAAAGCTCTTAGAAATTTAGTGTGCCTTGTAAGTTGGAATGG |  |
| PavOTS1_‘Garnet’ |  | GCAGTCTACAAGTATATCTAATTTCTGACAAGTGTTGTGTTAAAATTGTATAACATCATAAAGCTCTTAGAAATTTAGTGTGCCTTGTAAGTTGGAATGG |  |
|  |  |  |  |
|  |  | ....|....|....|....|....|....|....|....|....|....|....|....|....|....|....|....|....|....|....|....| |  |
| ppa022300m |  | TTGGTAACTGTCGAATAGTAGCCAGCTATCACAAAATGAGGGGACTGTTGGGCTTGAGTGCGTAGCCTTACTTGGTTGGTTTGAAGCTTACCTCTTTTGG |  |
| PRUAV030235 |  | ~~~~~~~~~~~~~~~~~~~~~~~~~~~~~~~~~~~~~~~~~~~~~~~~~~~~~~~~~~~~~~~~~~~~~~~~~~~~~~~~~~~~~~~~~~~~~~~~~~~~ |  |
| PavOTS1_‘Regina’ |  | TTGGTAACTGTCGAATGGTACCCAGCTATCACAAAACGAGGGGACTGTTGGGCTTGAGTGTGTAGCCTTACTTGGTTGGTTTGAAGCTTACCTCTTTTGG |  |
| PavOTS1_‘Lapins’ |  | TTGGTAACTGTCGAATGGTACCCAGCTATCACAAAACGAGGGGACTGTTGGGCTTGAGTGTGTAGCCTTACTTGGTTGGTTTGAAGCTTACCTCTTTTGG |  |
| PavOTS1_‘Garnet’ |  | TTGGTAACTGTCGAATGGTACCCAGCTATCACAAAACGAGGGGACTGTTGGGCTTGAGTGTGTAGCCTTACTTGGTTGGTTTGAAGCTTACCTCTTTTGG |  |
|  |  |  |  |
|  |  | ....|....|....|....|....|....|....|....|....|....|....|....|....|....|....|....|....|....|....|....| |  |
| ppa022300m |  | TATCTCCTACCAGGTCCCCCAGCAGAGAAATGAGTATGATTGTGGTCTCTTTGTACTTTTCTTCATGGAGCGATTCATTGAAGAGGCTCCTCAAAGGCTG |  |
| PRUAV030235 |  | ~~~~~~~~~~~~~GTGCCCCAACAGAGAAATGAGTATGATTGTGGTCTCTTTGTACTTTTCTTCATGGAGCGATTCATTGAAGAGGCTCCTCAAAGGCTG |  |
| PavOTS1_‘Regina’ |  | TATCTCCTACCAGGTGCCCCAACAGAGAAATGAGTATGATTGTGGTCTCTTTGTACTTTTCTTCATGGAGCGATTCATTGAAGAGGCTCCTCAAAGGCTG |  |
| PavOTS1_‘Lapins’ |  | TATCTCCTACCAGGTGCCCCAACAGAGAAATGAGTATGATTGTGGTCTCTTTGTACTTTTCTTCATGGAGCGATTCATTGAAGAGGCTCCTCAAAGGCTG |  |
| PavOTS1_‘Garnet’ |  | TATCTCCTACCAGGTGCCCCAACAGAGAAATGAGTATGATTGTGGTCTCTTTGTACTTTTCTTCATGGAGCGATTCATTGAAGAGGCTCCTCAAAGGCTG |  |
|  |  |  |  |
|  |  | ....|....|....|....|....|....|....|....|....|....|....|....|....|....|....|....|....|....|....|....| |  |
| ppa022300m |  | CAAAGGAAAAATTTAGCAATGGTACAGGGTTCTTGTGTTCTCAACCTCTCCTTCCTTCTTAAAAAGAAAAGCAATGATCTTAAACATGTTTTTTTTTTCT |  |
| PRUAV030235 |  | CAAAGGAAAAATTTAGCAATG~~~~~~~~~~~~~~~~~~~~~~~~~~~~~~~~~~~~~~~~~~~~~~~~~~~~~~~~~~~~~~~~~~~~~~~~~~~~~~~ |  |
| PavOTS1_‘Regina’ |  | AAAAAGGAAAAAAA |  |
| PavOTS1_‘Lapins’ |  | CAAAGGAAAAAA |  |
| PavOTS1_‘Garnet’ |  | AAAAAGGAAAAAA |  |

***PHYC***

|  |  | ....|....|....|....|....|....|....|....|....|....|....|....|....|....|....|....|....|....|....|....| |  |
| --- | --- | --- | --- |
| ppa000506m | 1801 | GATGACAGGATACAGAGAGTGGATGAACTGCGAATTGTCACAAATGAAATGGTTCGACTTATTGAGACAGCTGCAGTTCCCATTTTGGCTGTTGATGCCT |  |
| PRUAV008814 |  | GATGACAGGATACAGAGAGTGGATGAACTGCGAATTGTCACAAATGAAATGGTTCGACTTATTGAGACAGCTGCAGTTCCCATTTTGGCTGTTGATGCCT |  |
| PavPHYC_‘Regina’ |  | ~~~~~~~~~~~~~~~~~~~~~~~~~~~~~~~~~~~~~~~~~~~~~~~~~~~~~~~~~~~~~~~~~~~~~~~~~~~~~~~~~~~~~~~~~~~~~~~~~~~~ |  |
| PavPHYC_‘Lapins’ |  | ~~~~~~~~~~~~~~~~~~~~~~~~~~~~~~~~~~~~~~~~~~~~~~~~~~~~~~~~~~~~~~~~~~~~~~~~~~~~~~~~~~~~~~~~~~~~~~~~~~~~ |  |
| PavPHYC_‘Garnet’ |  | ~~~~~~~~~~~~~~~~~~~~~~~~~~~~~~~~~~~~~~~~~~~~~~~~~~~~~~~~~~~~~~~~~~~~~~~~~~~~~~~~~~~~~~~~~~~~~~~~~~AA |  |
|  |  |  |  |
|  |  | ....|....|....|....|....|....|....|....|....|....|....|....|....|....|....|....|....|....|....|....| |  |
| ppa000506m |  | CTGGTAATATAAATGGGTGGAATACAAAAGCATCCGAACTAACAGAATTGGCTGTTGAGAAAGCCATTGGTATGCCATTGGTTGATGTTGTTGGGGATGA |  |
| PRUAV008814 |  | CTGGTAATATAAATGGGTGGAATACAAAAGCATCCGAACTAACAGAATTGGCTGTTGAGAAAGCCATTGGTATGCCATTGGTTGATGTTGTTGGGGATGA |  |
| PavPHYC_‘Regina’ |  | ~~~CAAGATCCGAATCGGTGGATACAAA~GCATCCGAACTAACAGAATTGGCTGTTGAGAAAGCCATTGGTATGCCATTGGTTGATGTTGTTGGGGATGA |  |
| PavPHYC_‘Lapins’ |  | AGGCCATGGTCACTGCTTTTGTTATATTAGCATCCGAACTAACAGAATTGGCTGTTGAGAAAGCCATTGGTATGCCATTGGTTGATGTTGTTGGGGATGA |  |
| PavPHYC_‘Garnet’ |  | AACGGGAATTGCAGGGTAGGTATACAAAAGCATCCGAACTAACAGAATTGGCTGTTGAGAAAGCCATTGGTATGCCATTGGTTGATGTTGTTGGGGATGA |  |
|  |  |  |  |
|  |  | ....|....|....|....|....|....|....|....|....|....|....|....|....|....|....|....|....|....|....|....| |  |
| ppa000506m |  | TTCAATCGAAGTGGTAAAGGACATGCTCTCATCTGCGCTACAAGGTGACACTACTATTCGTCCTCTGGGTCTGGATTGTTAAACCCATAATAACAAATTG |  |
| PRUAV008814 |  | TTCAATCGAAGTGGTAAAGGACATGCTTTCATCTGTGCTACAAG~~~~~~~~~~~~~~~~~~~~~~~~~~~~~~~~~~~~~~~~~~~~~~~~~~~~~~~~ |  |
| PavPHYC_‘Regina’ |  | TTCAATCGAAGTGGTAAAGGACATGCTTTCATCTGTGCTACAAGGTGACACTACTATTCGTACTCTGGGTCTGGATTGTTAAACCCGTTATAACAAATTG |  |
| PavPHYC_‘Lapins’ |  | TTCAATCGAAGTGGTAAAGGACATGCTTTCATCTGTGCTACAAGGTGACACTACTATTCGTACTCTGGGTCTGGATTGTTAAAACCGTTATAACAAATTG |  |
| PavPHYC_‘Garnet’ |  | TTCAATCGAAGTGGTAAAGGACATGCTTTCATCTGTGCTACAAGGTGACACTACTATTCGTACTCTGGGTCTGGATTGTTAAAACCGTTATAACAAATTG |  |
|  |  |  |  |
|  |  | ....|....|....|....|....|....|....|....|....|....|....|....|....|....|....|....|....|....|....|....| |  |
| ppa000506m |  | AAACCTGTTACTGACTGATTT~~~~~~~~~~GCCATGGAGTATAAGTAGACCAGAATAAGCGAAACAGACATGCTCAGAATAGTCTTTGGATGTTTGTTT |  |
| PRUAV008814 |  | ~~~~~~~~~~~~~~~~~~~~~~~~~~~~~~~~~~~~~~~~~~~~~~~~~~~~~~~~~~~~~~~~~~~~~~~~~~~~~~~~~~~~~~~~~~~~~~~~~~~~ |  |
| PavPHYC_‘Regina’ |  | AAACCTGTTGCTGACTGATTTTTGGTTATGTGCCATGGAATATAAGTAGACCAGAATA~GTGATTCAGACATGCTCAGAATAGTCTTTGGATGTTTGTTT |  |
| PavPHYC_‘Lapins’ |  | AAACCTGTTGCTGACTGATTTTTGGTTATGTGCCATGGAATATAAGTAGACCAGAATA~GTGATTCAGACATGCTCAGAATAGTCTTTGGATGTTTGTTT |  |
| PavPHYC_‘Garnet’ |  | AAACCTGTTGCTGACTGATTTTTGGTTATGTGCCATGGAATATAAGTAGACCAGAATA~GTGATTCAGACATGCTCAGAATAGTCTTTGGATGTTTGTTT |  |
|  |  |  |  |
|  |  | ....|....|....|....|....|....|....|....|....|....|....|....|....|....|....|....|....|....|....|....| |  |
| ppa000506m |  | ACCTGACCTATACTTGGTGATAGATCATTAATTGCAATGTGGTGTTCTAATTGTTAGGAGCAGCTTTTCTGGCCTGTCTACTTTGATATTCATACATTCT |  |
| PRUAV008814 |  | ~~~~~~~~~~~~~~~~~~~~~~~~~~~~~~~~~~~~~~~~~~~~~~~~~~~~~~~~~~~~~~~~~~~~~~~~~~~~~~~~~~~~~~~~~~~~~~~~~~~~ |  |
| PavPHYC_‘Regina’ |  | ACCTGACCTATACTTGGTGATAAATCATTAATTGCAATGTGGTGTTCTAATTGTTAGGAGCAGCTTTTCTGGGCTGTCTACTTTGATATTCAAACATTCT |  |
| PavPHYC_‘Lapins’ |  | ACCTGACCTATACTTGGTGATAAATCATTAATTGCAATGTGGTGTTCTAATTGTTAGGAGCAGCTTTTCTGGGCTGTCTACTTTGATATTCAAACATTCT |  |
| PavPHYC_‘Garnet’ |  | ACCTGACCTATACTTGGTGATAAATCATTAATTGCAATGTGGTGTTCTAATTGTTAGGAGCAGCTTTTCTGGGCTGTCTACTTTGATATTCAAACATTCT |  |
|  |  |  |  |
|  |  | ....|....|....|....|....|....|....|....|....|....|....|....|....|....|....|....|....|....|....|....| |  |
| ppa000506m |  | TTCAGACTTTGTGAATTTTCACAAAAATCAAAGCAGATGATTTGCACTTTTTCCTATGCTTAGGTTGTGTTTGGTTTATGGGTTGCTTCCTGACCCACAA |  |
| PRUAV008814 |  | ~~~~~~~~~~~~~~~~~~~~~~~~~~~~~~~~~~~~~~~~~~~~~~~~~~~~~~~~~~~~~~~~~~~~~~~~~~~~~~~~~~~~~~~~~~~~~~~~~~~~ |  |
| PavPHYC_‘Regina’ |  | TTCAGACTTTGTGAATTTTCACAAGAATCAAAGCAGATGATTTGCCCTTTTTCCTATGCTTTGGTTGTGTTTGGTTTATGGGTTGCTTCCTGACCCACAA |  |
| PavPHYC_‘Lapins’ |  | TTCAGACTTTGTGAATTTTCACAAGAATCAAAGCAGATGATTTGCCCTTTTTCCTATGCTTTGGTTGTGTTTGGTTTATGGGTTGCTTCCTGACCCACAA |  |
| PavPHYC_‘Garnet’ |  | TTCAGACTTTGTGAATTTTCACAAGAATCAAAGCAGATGATTTGCCCTTTTTCCTATGCTTTGGTTGTGTTTGGTTTATGGGTTGCTTCCTGACCCACAA |  |
|  |  |  |  |
|  |  | ....|....|....|....|....|....|....|....|....|....|....|....|....|....|....|....|....|....|....|....| |  |
| ppa000506m |  | CACACCCACTCTTCTACTTGTATGACACAATTATTTGGTTGCCTAATTAACAAATCCTAGAGATATAATTGTTTGGAATTAAATAGAGATACCTCTTCTG |  |
| PRUAV008814 |  | ~~~~~~~~~~~~~~~~~~~~~~~~~~~~~~~~~~~~~~~~~~~~~~~~~~~~~~~~~~~~~~~~~~~~~~~~~~~~~~~~~~~~~~~~~~~~~~~~~~~~ |  |
| PavPHYC_‘Regina’ |  | CACACCCACTCTTCTACTTGTATGACACAATTATTTGGTTGCCTAATTAACAAATCCTACAGATATAATTGTTTGGAATTAAATAGAGATACCTCTTCTG |  |
| PavPHYC_‘Lapins’ |  | CACACCCACTCTTCTACTTGTATGACACAATTATTTGGTTGCCTAATTAACAAATCCTACAGATATAATTGTTTGGAATTAAATAGAGATACCTCTTCTG |  |
| PavPHYC_‘Garnet’ |  | CACACCCACTCTTCTACTTGTATGACACAATTATTTGGTTGCCTAATTAACAAATCCTACAGATATAATTGTTTGGAATTAAATAGAGATACCTCTTCTG |  |
|  |  |  |  |
|  |  | ....|....|....|....|....|....|....|....|....|....|....|....|....|....|....|....|....|....|....|....| |  |
| ppa000506m |  | GATTCTTTTTGTTCCCATCTCATGCATGTCAAGTAAGTGGAAATTACAGTTATGCATGTAAAGTATGAAGTTTGTAGAACAAAGGCAGATATTTTATAAT |  |
| PRUAV008814 |  | ~~~~~~~~~~~~~~~~~~~~~~~~~~~~~~~~~~~~~~~~~~~~~~~~~~~~~~~~~~~~~~~~~~~~~~~~~~~~~~~~~~~~~~~~~~~~~~~~~~~~ |  |
| PavPHYC_‘Regina’ |  | GATTCTTTTTRTTTCCATCTCATGCATGTCAAGTAAGTGGAAATTACAGTTATGCATGTCAAGTATGAAGTTTGTAGAACAAAGGCAGATATTTTATAAT |  |
| PavPHYC_‘Lapins’ |  | GATTCTTTTTGTTTCCATCTCATGCATGTCAAGTAAGTGGAAATTACAGTTATGCATGTCAAGTATGAAGTTTGTAGAACAAAGGCAGATATTTTATAAT |  |
| PavPHYC_‘Garnet’ |  | GATTCTTTTTGTTTCCATCTCATGCATGTCAAGTAAGTGGAAATTACAGTTATGCATGTCAAGTATGAAGTTTGTAGAACAAAGGCAGATATTTTATAAT |  |
|  |  |  |  |
|  |  | ....|....|....|....|....|....|....|....|....|....|....|....|....|....|....|....|....|....|....|....| |  |
| ppa000506m |  | TGAGAAGTGTGCTTTTCTTTTACGTTACATACATGTTAGTCTTATTCATTTCTTGGATTCCATCTCTTTTTTCTTTTCTGCATTTTTTCCTTTATTTGAT |  |
| PRUAV008814 |  | ~~~~~~~~~~~~~~~~~~~~~~~~~~~~~~~~~~~~~~~~~~~~~~~~~~~~~~~~~~~~~~~~~~~~~~~~~~~~~~~~~~~~~~~~~~~~~~~~~~~~ |  |
| PavPHYC_‘Regina’ |  | TTAGAAGTGTGCTTTTCTTTTATGTTACATACATGTTAATCTTATTCATTTCTTGGATTCCATCTCTTTTTTCTTTTCTGTAATTTTTCCTTTATTTGAT |  |
| PavPHYC_‘Lapins’ |  | TTAGAAGTGTGCTTTTCTTTTATGTTACATACATGTTAATCTTATTCATTTCTTGGATTCCATCTCTTTTTTCTTTTCTGTAATTTTTCCTTTATTTGAT |  |
| PavPHYC_‘Garnet’ |  | TTAGAAGTGTGCTTTTCTTTTATGTTACATACATGTTAATCTTATTCATTTCTTGGATTCCATCTCTTTTTTCTTTTCTGTAATTTTTCCTTTATTTGAT |  |
|  |  |  |  |
|  |  | ....|....|....|....|....|....|....|....|....|....|....|....|....|....|....|....|....|....|....|....| |  |
| ppa000506m |  | TTGGTTGTATTTTTTGTTGTGTTGTTCCCTCTTCTGAGGTGATTGTAAATTGGCAGGTGTAGAAA~AGAAAAATGTTGAAATCAAATTGAAAACATTTGG |  |
| PRUAV008814 |  | ~~~~~~~~~~~~~~~~~~~~~~~~~~~~~~~~~~~~~~~~~~~~~~~~~~~~~~~~GTGTAGAAA~AGAAAAATGTTGAAATCAAATTGAAAATATTTGG |  |
| PavPHYC_‘Regina’ |  | TCGGTTGTATTTTTTCTTGTGTTGTTCCCTCTTCTGATGTGATTGTAAATTGGCAGGTGTAGAAA~AGAAAAATGTTGAAATCAAATTGAAAATATTTGG |  |
| PavPHYC_‘Lapins’ |  | TCGGTTGTATTTTTTCTTGTGTTGTTCCCTCTTCTGATGTGATTGTAAATTGGCAGGTGTAGAAATAGAAAAATGTTGAAATCAA~TTGAAA~TATTTGG |  |
| PavPHYC_‘Garnet’ |  | TCGGTTGTATTTTTTCTTGTGTTGTTCCCTCTTCTGATGTGATTGTAAATTGGCAGGTGTAGAAA~AGAAAAATGTTGAAATCAAATTGAAAATATTTGG |  |
|  |  |  |  |
|  |  | ....|....|....|....|....|....|....|....|....|....|....|....|....|....|....|....|....|....|....|....| |  |
| ppa000506m |  | TCGTCAAGAAAATGATAGTTTTGTAACCTTGGTGGTTAATGCATGTTGTAGCCGAGATATAAAGG~AAGATGTAGTTGGGGCTTGCTTTGTAAGCC~AAG |  |
| PRUAV008814 |  | CCGTCAAGAAAATGATAGTTTTGTAACCTTGGTGGTTAGTGCATGTTGTAGCCGAGATATAAAGG~AAGATGTAGTTGGGGCTTGCTTTGTAAGCC~AAG |  |
| PavPHYC_‘Regina’ |  | CCGTCAAGAAAATGATAGTTTTGTAACCTTGGTGGTTAGTGCATGTTGTAGCCGAGATATAAAGG~AAGATGTAGTTGGGGCTTGCTTTGTAAGCC~AAG |  |
| PavPHYC_‘Lapins’ |  | CCGTCAAGAAAATGATAGTTTTGTAACCTTGGTGGTTAGTGCATGTTGTAGCCGAGATATAAAGG~AAGATGTAGTTGGGGCTTGCTTTGTAAGCCCAAG |  |
| PavPHYC_‘Garnet’ |  | CCGTCAAGAAAATGATAGTTTTGTAACCTTGGTGGTTAGTGCATGTTGTAGCCGAGATATAAAGGGAAGATGTAGTTGGGGCTTGCTTTGTAAGCC~~AG |  |
|  |  |  |  |
|  |  | ....|....|....|....|....|....|....|....|....|....|....|....|....|....|....|....|....|....|....|....| |  |
| ppa000506m |  | ATCTT~ACAGGAGAGAAGTTGGGT~AT~GGACAAATAT~ACCCGTTT~GCTAGGTGATTATATTGGAATAGTGCGGAGCCCATCTGCACTGATCCCTCCT |  |
| PRUAV008814 |  | ATCTT~ACAGGAGAGAAGTTGGTT~AT~GGACAAATAT~ACCCGTTT~GCTAGGTGATTATATTGGAATAGTGCGGAGCCCATCTGCACTGATTCCTCCT |  |
| PavPHYC_‘Regina’ |  | ATCTT~ACAGGAGAGAAGTTGGTTTATTGGACAATTTACACCGGTTTGGC~AAA |  |
| PavPHYC_‘Lapins’ |  | ATCTTTACAGGAGAGAAGTTGGTT~AT~GGACAATTATACCCCGTTTTGC~AC |  |
| PavPHYC_‘Garnet’ |  | ATCTT~ACAGGAGAGAAGTTGGTT~AT~GGACAATAATACCCCGTTT~GCGATT |  |

***PIE1***

|  |  | ....|....|....|....|....|....|....|....|....|....|....|....|....|....|....|....|....|....|....|....| |  |
| --- | --- | --- | --- |
| ppa000063m | 8601 | AACACAAATCCCAAAATTTTTCTTTTCATATTGTCAACCCGTAGTGGGGGTGTTGGAATAAACCTAGTTGGGGCAGATACTGTTATCTTTTATGATAGTG |  |
| PRUAV020482 |  | aacacaaatcccaaaatttttcttttcatattgtcaacccgtagtgggggtgttggaataaacctagttggggcagatactgttatcttttatgatagtg |  |
| PavPIE1_‘Regina’ |  | ~~~~~~~~~~~~~~~~~~~~~~~~~~~~~~~~~~~~~~~~~~~~~~~~~~~~~~~~~~~~~~~~~~~~~aaggcactGACT~~~~TCTTTTATGATAGTG |  |
| PavPIE1_‘Lapins’ |  | ~~~~~~~~~~~~~~~~~~~~~~~~~~~~~~~~~~~~~~~~~~~~~~~~~~~~~~~~~~~~~~~~~~~~~gagg~ac~g~ct~~T~TCTTTT~TGATAGTG |  |
| PavPIE1_‘Garnet’ |  | ~~~~~~~~~~~~~~~~~~~~~~~~~~~~~~~~~~~~~~~~~~~~~~~~~~~~~~~~~~~~~~~~~~~~~acgg~actGACT~~~~TCTTTTATGATAGTG |  |
|  |  |  |  |
|  |  | ....|....|....|....|....|....|....|....|....|....|....|....|....|....|....|....|....|....|....|....| |  |
| ppa000063m |  | ACTGGAATCCTGCTATGGACCAACAAGCTCAAGATCGATGCCACCGTATTGGACAGACACGTGAAGTTCATATTTATCGGTTGATTAGTCAGAGCACCAT |  |
| PRUAV020482 |  | actggaatcctgctatggaccaacaagctcaagatcgatgccaccgtattggacagacacgtgaagttcatatttancggttgatcagtcagagcactat |  |
| PavPIE1_‘Regina’ |  | ACTGGAATCCTGCTATGGACCAACAAGCTCAAGATCGATGCCACCGTATTGGACAGACACGTGAAGTTCATATTTATCGGTTGATCAGTCATAGCACTAT |  |
| PavPIE1_‘Lapins’ |  | ACTGGAATCCTGCTATGGACCAACAAGCTCAAGATCGATGCCACCGTATTGGACAGACACGTGAAGTTCATATTTATCGGTTGATCAGTCAGAGCACTAT |  |
| PavPIE1_‘Garnet’ |  | ACTGGAATCCTGCTATGGACCAACAAGCTCAAGATCGATGCCACCGTATTGGACAGACACGTGAAGTTCATATTTATCGGTTGATCAGTCAGAGCACTAT |  |
|  |  |  |  |
|  |  | ....|....|....|....|....|....|....|....|....|....|....|....|....|....|....|....|....|....|....|....| |  |
| ppa000063m |  | TGAAGAAAATATCCTGAAGAAAGCAAATCAGAAGCGTGCGCTTGATGATCTAGTTATACAAAGTGGGGGATACAACACTGAATTCTTCAAAAAGCTTGAT |  |
| PRUAV020482 |  | tgaagaaaatatcctgaagaaagcaaatcagaagcgtgcacttgatgatctagttatacaaagtgggggatacaacactgaattcttcaaaaagcttgat |  |
| PavPIE1_‘Regina’ |  | TGAAGAAAATATCCTGAAGAAAGCAAATCAGAAGCGTGCACTTGATGATCTAGTTATACAAAGTGGGGGATACAACACTGAATTCTTCAAAAAGCTTGAT |  |
| PavPIE1_‘Lapins’ |  | TGAAGAAAATATCCTGAAGAAAGCAAATCAGAAGCGTGCACTTGATGATCTAGTTATACAAAGTGGGGGATACAACACTGAATTCTTCAAAAAGCTTGAT |  |
| PavPIE1_‘Garnet’ |  | TGAAGAAAATATCCTGAAGAAAGCAAATCAGAAGCGTGCACTTGATGATCTAGTTATACAAAGTGGGGGATACAACACTGAATTCTTCAAAAAGCTTGAT |  |
|  |  |  |  |
|  |  | ....|....|....|....|....|....|....|....|....|....|....|....|....|....|....|....|....|....|....|....| |  |
| ppa000063m |  | CCTATGGAGCTTTTCTCTGGTCATAGGGCACTTCCTGTTAAGAATACGCAAAAGGAGAAAAATCACAACACAACTGAGGTTTCTCTATCCAATGCTGATC |  |
| PRUAV020482 |  | cctatggagcttttctctggtcatagggcacttcctgttaagaatttgcaaaaggagaaaaatcacaacacaactgaggtttctctatcaaatgctgatc |  |
| PavPIE1_‘Regina’ |  | CCTATGGAGCTTTTCTCTGGTCATAGGGCACTTCCTGTTAAGAATTTGCAAAAGGAGAAAAATCACAACACAACTGAGGTTTCTCTATCAAATGCTGATC |  |
| PavPIE1_‘Lapins’ |  | CCTATGGAGCTTTTCTCTGGTCATAGGGCACTTCCTGTTAAGAATTTGCAAAAGGAGAAAAATCACAACACAACTGAGGTTTCTCTATCAAATGCTGATC |  |
| PavPIE1_‘Garnet’ |  | CCTATGGAGCTTTTCTCTGGTCATAGGGCACTTCCTGTTAAGAATTTGCAAAAGGAGAAAAATCACAACACAACTGAGGTTTCTCTATCAAATGCTGATC |  |
|  |  |  |  |
|  |  | ....|....|....|....|....|....|....|....|....|....|....|....|....|....|....|....|....|....|....|....| |  |
| ppa000063m |  | TGGAAGCTGCTCTAAAGCATGCAGAAGATGAAGCCGATTACATGGCATTAAAGAAAGTTGAGCAGGAGGAAGCTGTTGACAATCAAGAATTTACAGAAGA |  |
| PRUAV020482 |  | tcgaagctgctctaaagcatgcagaagatgaagctgattacatggcattaaagaaagttgagcaggaggaagctgttgacaatcaagaatttacagaaga |  |
| PavPIE1_‘Regina’ |  | TCGAAGCTGCTCTAAAGCATGCAGAAGATGAAGCTGATTACATGGCATTAAAGAAAGTTGAGCAGGAG~AAGCTGTTTGGTTTATTCCACACCCccaag |  |
| PavPIE1_‘Lapins’ |  | TCGAAGCTGCTCTAAAGCATGCAGAAGATGAAGCTGATTACATGGCATTAAAGAAAGTTGAGCAGGAGAAAGCTGTTAGGTTTATTCCCACCCCCCcaa |  |
| PavPIE1_‘Garnet’ |  | TCGAAGCTGCTCTAAAGCATGCAGAAGATGAAGCTGATTACATGGCATTAAAGAAAGTTGAGCAGGAG~AAGCTGTTTGGTTTATTCCAcacccccaa |  |

***SAR3***

|  |  | ....|....|....|....|....|....|....|....|....|....|....|....|....|....|....|....|....|....|....|....| |  |
| --- | --- | --- | --- |
| ppa000667m | 1901 | GTCGCAACATAGTTGATGCAGGTCTGTTGATGGGTAGGTCATTTCGAGTAGGATGGGGTCCAAATGGAACCCTAGTCCATGCTGGAACACCTGTAGGGAG |  |
| PRUAV007201 |  | gtcgcaacatagttgatgcaggtctgttgatgggtaggtcatttcgagtaggatggggtcctaatggaaccctagtccatgctggaacacctgtagggag |  |
| PavSAR3_‘Regina’ |  | ~~~~~~~~~~~~~~~~~~~~~~~~~~~~~~~~~~~~~~~~~~~~~~~~ttTGAGAGGAGTC~~TATGGACCCTAGTCCATGCTGGAACACCTGTAGGGAG |  |
| PavSAR3_‘Lapins’ |  | ~~~~~~~~~~~~~~~~~~~~~~~~~~~~~~~~~~~~~~~~~~~~~~~~tggcaagaaggGGTCTATGGATACTAGTCCATGCTGGAACACCTGTAGGGAG |  |
| PavSAR3_‘Garnet’ |  | ~~~~~~~~~~~~~~~~~~~~~~~~~~~~~~~~~~~~~~~~~~~~~~~~ttggaAGAGGGGTC~TATGTACCCTAGTCCATGCTGGAACACCTGTAGGGAG |  |
|  |  |  |  |
|  |  | ....|....|....|....|....|....|....|....|....|....|....|....|....|....|....|....|....|....|....|....| |  |
| ppa000667m |  | TACTGGTTCTCAAATGATGTTATCATCCACAATCAATTTAGAGAAGGTTGCTATTGACAATGTTGTTAGGGATGAAAA~~~TAACAAAGTTAGAGAGGAA |  |
| PRUAV007201 |  | tattggttctcaaatgatgttatcatccacaatcaatttagagaaggttgctattgacaatgttgttagggatgaaaanattaacaaagttagagaggaa |  |
| PavSAR3_‘Regina’ |  | TATTGGTTCTCAAATGATGTTATCATCCACAATCAATTTAGAGAAGGTTGCTATTGACAATGTTGTTAGGGATGAAAA~~~TAACAAAGTTAGAGAGGAA |  |
| PavSAR3_‘Lapins’ |  | TACTGGTTCTCAAATGATGTTATCATCCACAATCAATTTAGAGAAGGTTGCTATTGACAATGTTGTTAGGGATGAAAA~~~TAACAAAGTTAGAGAGGAA |  |
| PavSAR3_‘Garnet’ |  | TACTGGTTCTCAAATGATGTTATCATCCACAATCAATTTAGAGAAGGTTGCTATTGACAATGTTGTTAGGGATGAAAA~~~TAACAAAGTTAGAGAGGAA |  |
|  |  |  |  |
|  |  | ....|....|....|....|....|....|....|....|....|....|....|....|....|....|....|....|....|....|....|....| |  |
| ppa000667m |  | CTTATTGACACGGCTATTGATTCTCCACTAGATTTTCACATGGGATTACTTCATCAGACAGAAGAGATTGAAGTTGGATCCTTCAACCTAAGGCTGCAGA |  |
| PRUAV007201 |  | cttattgacatggctattgattctccactagattttcacaagggattacttcatcagacagaagagattgaagttggatcctttaacctaaggctgcaga |  |
| PavSAR3_‘Regina’ |  | CTTATTGACATGGCTATTGATTCTCCACTAGATTTTCACAAGGGATTACTTCATCAGACAGAAGAGATTGAAGTTGGATCCTTTAACCTAAGGCTGCAGA |  |
| PavSAR3_‘Lapins’ |  | CTTATTGACATGGCTATTGATTCTCCACTAGATTTTCACAWGGGATTACTTCATCAGACAGAAGAGATTGAAGTTGGATCCTTTAACCTAAGGCTGCAGA |  |
| PavSAR3_‘Garnet’ |  | CTTATTGACATGGCTATTGATTCTCCACTAGATTTTCACAWGGGATTACTTCATCAGACAGAAGAGATTGAAGTTGGATCCTTTAACCTAAGGCTGCAGA |  |
|  |  |  |  |
|  |  | ....|....|....|....|....|....|....|....|....|....|....|....|....|....|....|....|....|....|....|....| |  |
| ppa000667m |  | AGGTTGTCTCTAATCGCTTGATGCTTTCAGAGATTTGCAGGAGCTATGTAGATATTATTGAGAAGCAGCTGGAAGTTCCTAGGCTATCTTCCTCTGCTCG |  |
| PRUAV007201 |  | aggttgtctctaatcgcttgatgctatcagagatttgcaggagctatgtagagatcattgagaagcagctggaagttcctaggctatcttcctctgctcg |  |
| PavSAR3_‘Regina’ |  | AGGTTGTCTCTAATCGCTTGATGCTATCAGAGATTTGCAGGAGCTATGTAGAGATCATTGAGAAGCAGCTGGAAGTTCCTAGGCTATCTTCCTCTCTTCG |  |
| PavSAR3_‘Lapins’ |  | AGGTTGTCTCTAATCGCTTGATGCTATCAGAGATTTGCAGGAGCTATGTAGAGATCATTGAGAAGCAGCTGGAAGTTCCTAGGCTATCTTCCTCTGTTCG |  |
| PavSAR3_‘Garnet’ |  | AGGTTGTCTCTAATCGCTTGATGCTATCAGAGATTTGCAGGAGCTATGTAGAGATCATTGAGAAGCAGCTGGAAGTTCCTAGGCTATCTTCCTCTCTCCG |  |
|  |  |  |  |
|  |  | ....|....|....|....|....|....|....|....|....|....|....|....|....|....|....|....|....|....|....|....| |  |
| ppa000667m |  | TTTGGGT~TTGACACACCAAATAATGATTTGGGAACTGATAAAAGTTCTTTTTTCTGATAGGGAAAATGGTGGGAAAATGAAATCTTTGGGTGCTGATAA |  |
| PRUAV007201 |  | tttggttnttgacacaccaagtaatgatttgggaactgataaaagttcttttt~ctgatagggaaa~tggtgggaaaatgaaaactttgggtgctgataa |  |
| PavSAR3_‘Regina’ |  | TTTGGTA |  |
| PavSAR3_‘Lapins’ |  | TTTGGTA |  |
| PavSAR3_‘Garnet’ |  | TTTGGTTGCCATC |  |

***SPA1***

|  |  | ....|....|....|....|....|....|....|....|....|....|....|....|....|....|....|....|....|....|....|....| |  |
| --- | --- | --- | --- |
| ppa014569m | 2801 | TATACCAGTCCAGAGGAGCTCAATGAGAGTGGTTCCACACTTCCATCAAATGTCTATTGCCTTGGGGTTCTTCTTTTCGAGGTGAGAAGTTATTTTGAAG |  |
| PRUAV011454 |  | tataccagtccagaggagctcaatgagagagcttccacactttcatcaaatgtctattgccttggggttcttcttttcgag~~~~~~~~~~~~~~~~~~~ |  |
| PavSPA1_‘Regina’ |  | ~~~~~~~~~~~~~~~~~~~~~~~~~~~~~~~~~tc~~c~GTTTC~TCAAATGTCTATTGCCTTGGGGTTCTTCTTTTCGAGGTGAGAAGTTATTTTGAAG |  |
| PavSPA1_‘Lapins’ |  | ~~~~~~~~~~~~~~~~~~~~~~~~~~~~~~~~~tc~AC~GTTTC~TCAAATGTCTATTGCCTTGGGGTTCTTCTTTTCGAGGTGAGAAGTTATTTTGAAG |  |
| PavSPA1_‘Garnet’ |  | ~~~~~~~~~~~~~~~~~~~~~~~~~~~~~~~~~tC~AC~GTTTC~TCAA~TGTCTATTGCCTTGGGGTTCTTCTTTTCGAGGTGAGAAGTTATTTTGAAG |  |
|  |  |  |  |
|  |  | ....|....|....|....|....|....|....|....|....|....|....|....|....|....|....|....|....|....|....|....| |  |
| ppa014569m |  | TGTTTCCACAAGTTCTTATTATTTGAAAGTTAACTTGAACAACTTGTACATCTTGGCAGATGATTTTATTATATAATTTGGTTTGAACTAACATAACTTA |  |
| PRUAV011454 |  | ~~~~~~~~~~~~~~~~~~~~~~~~~~~~~~~~~~~~~~~~~~~~~~~~~~~~~~~~~~~~~~~~~~~~~~~~~~~~~~~~~~~~~~~~~~~~~~~~~~~~ |  |
| PavSPA1_‘Regina’ |  | TGTTTCCACAAGTTCTTATTATTTGAAAGTTAACTTAAACAACTTGTACTTCTTGGCAGATGATTTTATTATATAATTTGGTTTGAACTAACATAACTTA |  |
| PavSPA1_‘Lapins’ |  | TGTTTCCACAAGTTCTTATTATTTGAAAGTTAACTTAAACAACTTGTACTTCTTGGCAGATGATTTTATTATATAATTTGGTTTGAACTAACATAACTTA |  |
| PavSPA1_‘Garnet’ |  | TGTTTCCACAAGTTCTTATTATTTGAAAGTTAACTTAAACAACTTGTACTTCTTGGCAGATGATTTTATTATATAATTTGGTTTGAACTAACATAACTTA |  |
|  |  |  |  |
|  |  | ....|....|....|....|....|....|....|....|....|....|....|....|....|....|....|....|....|....|....|....| |  |
| ppa014569m |  | AGACTTGTTGTTCGCTTGTTATGACACTGTAAGTTAATTCTGAAATGATGAATAGGTAACATTCCTTGTGCTAAGTTCTGCTATCAAATTAATGTAAGAA |  |
| PRUAV011454 |  | ~~~~~~~~~~~~~~~~~~~~~~~~~~~~~~~~~~~~~~~~~~~~~~~~~~~~~~~~~~~~~~~~~~~~~~~~~~~~~~~~~~~~~~~~~~~~~~~~~~~~ |  |
| PavSPA1_‘Regina’ |  | AGACTTGTTGTTCGCTTGTTATGACACTGTAAGTTAATTTTGAAGTGATGAATAGGTAAAATTCCTTGTGCTAAGTTCTGCTATCAAATTAATGTAAGAA |  |
| PavSPA1_‘Lapins’ |  | AGACTTGTTGTTCGCTTGTTATGACACTGTAAGTTAATTTTGAAGTGATGAATAGGTAAAATTCCTTGTACTAAGTTCTGCTATCAAATTAATGTAAGAA |  |
| PavSPA1_‘Garnet’ |  | AGACTTGTTGTTCGCTTGTTATGACACTGTAAGTTAATTTTGAAGTGATGAATAGGTAAAATTCCTTGTACTAAGTTCTGCTATCAAATTAATGTAAGAA |  |
|  |  |  |  |
|  |  | ....|....|....|....|....|....|....|....|....|....|....|....|....|....|....|....|....|....|....|....| |  |
| ppa014569m |  | ATAGTTTGATCTCAAAACTGGAAAAATAACAGGGACTCAGTAGTGCCACTGAATGGCTCTTTATCCTTGTAATCAATCTTCGTTTGTCTTTATTTTATTT |  |
| PRUAV011454 |  | ~~~~~~~~~~~~~~~~~~~~~~~~~~~~~~~~~~~~~~~~~~~~~~~~~~~~~~~~~~~~~~~~~~~~~~~~~~~~~~~~~~~~~~~~~~~~~~~~~~~~ |  |
| PavSPA1_‘Regina’ |  | ATAGTTTGCTCCCAAAACTGGAAAAGTAACAGGGACTCAGTAGTGCCACTGAATGGCTCTTTATCCTTGTAATCAATCTTCGTTTGTCTTTCTTTTATTT |  |
| PavSPA1_‘Lapins’ |  | ATAGTTTGCTCCCAAAACTGGAAAAGTAACAGGGACTCAGTAGTGCCACTGAATGGCTCTTTATCCTTGTAATCAATCTTCGTTTGTCTTTCTTTTATTT |  |
| PavSPA1_‘Garnet’ |  | ATAGTTTGCTCCCAAAACTGGAAAAGTAACAGGGACTCAGTAGTGCCACTGAATGGCTCTTTATCCTTGTAATCAATCTTCGTTTGTCTTTCTTTTATTT |  |
|  |  |  |  |
|  |  | ....|....|....|....|....|....|....|....|....|....|....|....|....|....|....|....|....|....|....|....| |  |
| ppa014569m |  | TATTTT~~GAGTTCTAAAGTTCTATCCTTGGGGTTGGAGTAGAAAATCTATACATTTGACTTGTTTAGCTCGTTTATGTCAGTTGCTGTGCCGTTGTGAA |  |
| PRUAV011454 |  | ~~~~~~~~~~~~~~~~~~~~~~~~~~~~~~~~~~~~~~~~~~~~~~~~~~~~~~~~~~~~~~~~~~~~~~~~~~~~~~~~~~ttgctgtgccgttgtgaa |  |
| PavSPA1_‘Regina’ |  | TATTTTTTGAGTTCTAAAGTTCTATCCTTGGGGTTGGAGTAGAAAATCTGTACATTTGACTTGTTTAGCTCTTTTATGTCAGTTGCTGTGCCGTTGTGAA |  |
| PavSPA1_‘Lapins’ |  | TATTTTTTGAGTTCTAAAGTTCTATCCTTGGGGTTGGAGTAGAAAATCTGTACATTTGACTTGTTTAGCTCTTTTATGTCAGTTGCTGTGCCGTTGTGAA |  |
| PavSPA1_‘Garnet’ |  | TATTTTTTGAGTTCTAAAGTTCTATCCTTGGGGTTGGAGTAGAAAATCTGTACATTTGACTTGTTTAGCTCTTTTATGTCAGTTGCTGTGCCGTTGTGAA |  |
|  |  |  |  |
|  |  | ....|....|....|....|....|....|....|....|....|....|....|....|....|....|....|....|....|....|....|....| |  |
| ppa014569m |  | TCATGGGAGGTGCATTGCGCGGTGATGTTGGATTTGCACCATCGAATTCTGCCACCAAAGTTTCTTTCACAAAATCCGCTGGAAGCTGGCTTTTGTTTTT |  |
| PRUAV011454 |  | tcatgggaggtacattgcgcggtgatgttggatttgcaccatcgaattctgccaccagagtttctttcacaaaatccgctggaagctggcttttgttttt |  |
| PavSPA1_‘Regina’ |  | TCATGGGAGGTACATTGCGCGGTGATGTTGGATTTGCACCATCGAATTCTGCCACCAGAGTTTCTTTCACAAAAATCCGCTGA |  |
| PavSPA1_‘Lapins’ |  | TCATGGGAGGTACATTGCGCGGTGATGTTGGATTTGCACCATCGAATTCTGCCACCAGAGTTTCTTTCAAAAAATTTCGCCTGaa |  |
| PavSPA1_‘Garnet’ |  | TCATGGGAGGTACATTGCGCGGTGATGTTGGATTTGCACCATCGAATTCTGCCACCAGAGTTTCTTTCAAAAAATTTCCCTGGa |  |

***SUF4***

|  |  | ....|....|....|....|....|....|....|....|....|....|....|....|....|....|....|....|....|....|....|....| |  |
| --- | --- | --- | --- |
| ppa018474m | 1201 | AGAGTCAACGGATATTGAAATATATGGGATGCAAGGAATCCCACCTGATGTCTTGGCTGCACATTATGGAGAGGAAGGTAAAGGATCCTTACAAATATGA |  |
| PRUAV007354 |  | agagtcgacggatattgaaatatatgggatgcaaggaatcccacctgacgtcttggctgcacattatggagaggaag~~~~~~~~~~~~~~~~~~~~~~~ |  |
| PavSUF4_‘Regina’ |  | ~~~~~~~~~~~~~~~~~~~~~~~~~~~~~~~~~~~~tgtagctcgctgtgttttgGGT~C~CAT~ATGGAGAGGAAGGTAAAGGATCCTTACAAATATGA |  |
| PavSUF4_‘Lapins’ |  | ~~~~~~~~~~~~~~~~~~~~~~~~~~~~~~~~~~~~~~~~~~~~~~~~~~~~~~~~~~~~~~~~~~~~~~~~~~~~~~~~~~~~~~~~~~~~~~~~~~~~ |  |
| PavSUF4_‘Garnet’ |  | ~~~~~~~~~~~~~~~~~~~~~~~~~~~~~~~~~~~~ttgtgagttttttgtgGGGGCT~CACAT~ATGGAGAGGAAGGTGAAGGATCCTTACAAATATGA |  |
|  |  |  |  |
|  |  | ....|....|....|....|....|....|....|....|....|....|....|....|....|....|....|....|....|....|....|....| |  |
| ppa018474m |  | TGTTTATCCAGAGAATTTTCTTATTTTATGTTTAGGTTATGTTATCCCCATTGATATT~GATGATGTTTAAATTTTT~~ATTTTATTTAAATGATTAAAT |  |
| PRUAV007354 |  | ~~~~~~~~~~~~~~~~~~~~~~~~~~~~~~~~~~~~~~~~~~~~~~~~~~~~~~~~~~~~~~~~~~~~~~~~~~~~~~~~~~~~~~~~~~~~~~~~~~~~ |  |
| PavSUF4_‘Regina’ |  | TGTTTATCCAGAGAATTTTCTTATTTTATGTTTAGGTTATGTTATCCCCGTTGATATTTGGTGATGGTTAAATTTTTT~ATTTTATTTCAATGATTAAAT |  |
| PavSUF4_‘Lapins’ |  | ~~~~~~~~~~~~~~~~~~~~~~~~~~~~~~~~~~~~~~~~~~~~~~~~~~~~~~~~~~~~~~~~~~~~~~~~~~~~~~~~~~~~~~~~~~~~~~~~~~~~ |  |
| PavSUF4_‘Garnet’ |  | TGTTTATCCAGAGAATTTTCTTATTTTATGTTTAGGTTATGTTATCCCCGTTGATATTTGGTGATGGTTAAATTTTT~~ATTTTATT~CAATGATTAAAT |  |
|  |  |  |  |
|  |  | ....|....|....|....|....|....|....|....|....|....|....|....|....|....|....|....|....|....|....|....| |  |
| ppa018474m |  | TCAACTGTTTTATGCCGCCTTTGGGTTTGATTCACATTAAAGTTTCACCTGGATGTGCTGGCACATGCAAGTATACATAAACCGACCCATTTGAATCAAA |  |
| PRUAV007354 |  | ~~~~~~~~~~~~~~~~~~~~~~~~~~~~~~~~~~~~~~~~~~~~~~~~~~~~~~~~~~~~~~~~~~~~~~~~~~~~~~~~~~~~~~~~~~~~~~~~~~~~ |  |
| PavSUF4_‘Regina’ |  | TGAACTGTTTTATGCCTTCTTTGGGTTTGATTCACATTAA~GTTTCACCTGGATGTGCTGGTACATGCAAATATACATAAACCAACCCATTTGAATCAAA |  |
| PavSUF4_‘Lapins’ |  | ~~~~~~~~~~~~~~~~~~~~~~~~~~~~~~~~~~~~~~~~~~~~~~~~~~~~~~~~~~~~~~~~~~~~~~~~~~~~~~~~~~~atcc~ttttgaatcaaa |  |
| PavSUF4_‘Garnet’ |  | TGAACTGTTTTATGCCTTCTTTGGGTTTGATTCACATTAA~GTTTCACCTGGATGTGCTGGTACATGCAAATATACATAAACCAACCCATTTGAATCAAA |  |
|  |  |  |  |
|  |  | ....|....|....|....|....|....|....|....|....|....|....|....|....|....|....|....|....|....|....|....| |  |
| ppa018474m |  | TTTCCTTGAATAGTATAATGTATTAAATAAGATGCTCTATTGGAGTGTTCATAGCAACATCCATGTAATAGTTATTTGGGTATAAAAGCTTTGATGATTG |  |
| PRUAV007354 |  | ~~~~~~~~~~~~~~~~~~~~~~~~~~~~~~~~~~~~~~~~~~~~~~~~~~~~~~~~~~~~~~~~~~~~~~~~~~~~~~~~~~~~~~~~~~~~~~~~~~~~ |  |
| PavSUF4_‘Regina’ |  | TTTCCTTGAATAGTATAATGTATTAAATAAGGTGCTCTATTGGAGTGTTCATAGCAACATCCATGTAATAGTTATTTGGGTATAAAAGCTTTGATGATTG |  |
| PavSUF4_‘Lapins’ |  | tttccttgaatagtataatgtattaaataaggtgttgtgttggagtgttaataagaacatccatgtaataggtatcaaggaaattaaggattgaaatatg |  |
| PavSUF4_‘Garnet’ |  | TTTCCTTGAATAGTATAATGTATTAAATAAGGTGATCTATTGGAGTGTTCATAGCAACATCCATGTAATAGTTATTTGGGTATAAAAGCTTTGATGATTG |  |
|  |  |  |  |
|  |  | ....|....|....|....|....|....|....|....|....|....|....|....|....|....|....|....|....|....|....|....| |  |
| ppa018474m |  | TATTTGGTCCAGTTGGAAATTTTTAACATGTTGAAATTCTCCTGTTTTCATGGTTGAACCAATTTACTTGCAAGAGAATGCATCCACATATAAACTCATG |  |
| PRUAV007354 |  | ~~~~~~~~~~~~~~~~~~~~~~~~~~~~~~~~~~~~~~~~~~~~~~~~~~~~~~~~~~~~~~~~~~~~~~~~~~~~~~~~~~~~~~~~~~~~~~~~~~~~ |  |
| PavSUF4_‘Regina’ |  | TATTTGGTCCAGTTGGAAATTTTTAACATGTTGAAATTCTCCTGTTTTCATGGTTGAACCAATTTACTTGCAAGAGAATGCATCCACATATAAACTCATG |  |
| PavSUF4_‘Lapins’ |  | tatttggtgcagtcgaaaaaggataacaagttgaaattgtcttgttttcatggt~caaccaattta~aagcaagataatgcatatacgtataaa~~~~tt |  |
| PavSUF4_‘Garnet’ |  | TATTTGGTCCAGTTGGAAATTTTTAACATGTTGAAATTCTCCTGTTTTCATGGTTGAACCAATTTACTTGCAAGAGAATGCATCCACATATAAACTCATG |  |
|  |  |  |  |
|  |  | ....|....|....|....|....|....|....|....|....|....|....|....|....|....|....|....|....|....|....|....| |  |
| ppa018474m |  | CAAGTACACACTCATTAGGATGTCCTAATCTGTGCATAAGAATTCTGTATGAAATGTTTTGTCGTGGTTTGTCTTAAAGACTAATCGAACCAACAAAAAG |  |
| PRUAV007354 |  | ~~~~~~~~~~~~~~~~~~~~~~~~~~~~~~~~~~~~~~~~~~~~~~~~~~~~~~~~~~~~~~~~~~~~~~~~~~~~~~~~~~~~~~~~~~~~~~~~~~~~ |  |
| PavSUF4_‘Regina’ |  | CAAGTATACACTCATTCGGATGTCCTAATCTGTGCATAAGAATTCTGTATGAAATGTTTTGTCGTGGTTTGTCTTTAAGACTAATCGAACCAACAAAAAG |  |
| PavSUF4_‘Lapins’ |  | catgtacgtactcagtcagttggattaatttgtgtataagaattaattatgtaatgttttgtcgtggtttgtggt~AAGACTAATTGAACCAACAAAAAG |  |
| PavSUF4_‘Garnet’ |  | CAAGTATACACTCATTCGGATGTCCTAATCTGTGCATAAGAATTCTGTATGAAATGTTTTGTCGTGGTTTGTCTTTAAGACTAATTGAACCAACAAAAAG |  |
|  |  |  |  |
|  |  | ....|....|....|....|....|....|....|....|....|....|....|....|....|....|....|....|....|....|....|....| |  |
| ppa018474m |  | TGTACAGTACCTATCATTTTGTGTTGTTCATTTCATGTTATCTTGAAGTTATAGCATTAATCATATAAGTGGAGTTTATTTTCTAAATATTTTAATTTAT |  |
| PRUAV007354 |  | ~~~~~~~~~~~~~~~~~~~~~~~~~~~~~~~~~~~~~~~~~~~~~~~~~~~~~~~~~~~~~~~~~~~~~~~~~~~~~~~~~~~~~~~~~~~~~~~~~~~~ |  |
| PavSUF4_‘Regina’ |  | TGTACAGTACCTATCATTTTGTGTTGTTCATTTCATGTCCTCTTGAAGTTATAGCATTAATCATATAAGTTAAGTTTA~~~~~~~~~~~~~~~CATTTAT |  |
| PavSUF4_‘Lapins’ |  | TGTACAGTACCTATCATTTTGTGTTGTTCATTTCATGTCCTCTTGAAGTTATAGCATTAATCATATAAGTTAAGTTTA~~~~~~~~~~~~~~~CATATAT |  |
| PavSUF4_‘Garnet’ |  | TGTACAGTACCTATCATTTTGTGTTGTTCATTTCATGTCCTCTTGAAGTTATAGCATTAATCATATAAGTTAAGTTTA~~~~~~~~~~~~~~~AATTTAT |  |
|  |  |  |  |
|  |  | ....|....|....|....|....|....|....|....|....|....|....|....|....|....|....|....|....|....|....|....| |  |
| ppa018474m |  | TTGCAGATGAGGACGCTCCATCAAAAGTAGCTAAAGTGGACATCCCGTCAACCCAGTTTGTTGGTGGTATGGTGCCAGGTTCGATGGGGATTGGATATCC |  |
| PRUAV007354 |  | ~~~~~~atgaggacgctccatcaaaagtagctaaagtggacatcccatcaacccagtttgttggtggtatggtaccaggttcgatggggattggatatcc |  |
| PavSUF4_‘Regina’ |  | TTGCAGATGAGGACGCTCCATCAAAAGTAGCTAAAGTGGACATCCCATCAACCCAGTTTGTTGGTGGTATGGTACCAGGTTCGATGGGGATTGGATATCC |  |
| PavSUF4_‘Lapins’ |  | TTGCAGATGAGGACGCTCCATCAAAAGTAGCTAAAGTGGACATCCCRTCAACCCAGTTTGTTGGTGGTATGGTACCAGGTTCGATGGGGATTGGATATCC |  |
| PavSUF4_‘Garnet’ |  | TTGCAGATGAGGACGCTCCATCAAAAGTAGCTAAAGTGGACATCCCRTCAACCCAGTTTGTTGGTGGTATGGTACCAGGTTCGATGGGGATTGGATATCC |  |
|  |  |  |  |
|  |  | ....|....|....|....|....|....|....|....|....|....|....|....|....|....|....|....|....|....|....|....| |  |
| ppa018474m |  | TCCTCAACCAGCTTTGGGTGCAATCCGGCCAATGTATGTTTTCTGTGAGGTTTCTTGCATATCCTCCGGAACCAGCTTTCAAAAATTTGAAAACTATATT |  |
| PRUAV007354 |  | tccccaaccagctttgggtgcaatccggccaat~~~~~~~~~~~~~~~~~~~~~~~~~~~~~~~~~~~~~~~~~~~~~~~~~~~~~~~~~~~~~~~~~~~ |  |
| PavSUF4_‘Regina’ |  | TCCCCAACCAGCTTTGGGTGCAATCCGGCCAATGTATGTTTTCTGTGAGGTTTCTTGCATATCCTCCGCAAGCAGCTTTGTGAAATTTGAAAACTCTATT |  |
| PavSUF4_‘Lapins’ |  | TCCCCAACCAGCTTTGGGTGCAATCCGGCCAATGTATGTTTTCTGTGAGGTTTCTTGCATATCCTCCGCAAGCAGCTTTGTGAAATTTGAAAACTCTATT |  |
| PavSUF4_‘Garnet’ |  | TCCCCAACCAGCTTTGGGTGCAATCCGGCCAATGTATGTTTTCTGTGAGGTTTCTTGCATATCCTCCGCAAGCAGCTTTGTGAAATTTGAAAACTCTATT |  |
|  |  |  |  |
|  |  | ....|....|....|....|....|....|....|....|....|....|....|....|....|....|....|....|....|....|....|....| |  |
| ppa018474m |  | TTCTGTGTTACATA~~~~~TACCATTTACATAAACAACATGTGTGTATGTTTCTTTGGTTTTTGTTTGCACCAGTTACAGTCCTGCAGTTCCAATGCCTC |  |
| PRUAV007354 |  | ~~~~~~~~~~~~~~~~~~~~~~~~~~~~~~~~~~~~~~~~~~~~~~~~~~~~~~~~~~~~~~~~~~~~~~~~~~ttacagtcctgcagttccaatgcctc |  |
| PavSUF4_‘Regina’ |  | TTCTGTGTTACATACTGATTACCATTTACGTAAACAACTTATCTGTATGTTTCTTTGGTTTTTGTTTGCACCAGTTACAGTCCTGCAGTTCCAATGCCTC |  |
| PavSUF4_‘Lapins’ |  | TTCTGTGTTACATACTGATTACCATTTACGTAAACAACTTATCTGTATGTTTCTTTGGTTTTTGTTTGCACCAGTTACAGTCCTGCAGTTCCAATGCCTC |  |
| PavSUF4_‘Garnet’ |  | TTCTGTGTTACATACTGATTACCATTTACGTAAACAACTTATCTGTATGTTTCTTTGGTTTTTGTTTGCACCAGTTACAGTCCTGCAGTTCCAATGCCTC |  |
|  |  |  |  |
|  |  | ....|....|....|....|....|....|....|....|....|....|....|....|....|....|....|....|....|....|....|....| |  |
| ppa018474m |  | CAAATGCTTGGCAACCTCGTCCCCAGCCATGGTTTCCACAACTTCCAGCAGTCTCAATTCCTCCTCCTTCCTCATTGGGTTATGTGCCGCAGCCATTGTT |  |
| PRUAV007354 |  | caaatgcttggcaacctcgtccccagccatggtttccacaacttccctcagtctcaattcctcctccttcctcattgggttatgtgccgcagccattgtt |  |
| PavSUF4_‘Regina’ |  | CAAATGCTGCTCTCCCCcccc |  |
| PavSUF4_‘Lapins’ |  | CAAATGCTGCACTCGCCcacc |  |
| PavSUF4_‘Garnet’ |  | CAAATGCTGCATCTCCCcgcc |  |

***SVP***

|  |  | ....|....|....|....|....|....|....|....|....|....|....|....|....|....|....|....|....|....|....|....| |  |
| --- | --- | --- | --- |
| ppa011063m | 51 | GCAGGTGACCTTTTCCAAGCGGAGGAGAGGGCTTTTCAAGAAGGCTCAGGAGCTCTCCGTTCTCTGTGATGCAGATATTGCTCTTATCATCTTTTCTTCC |  |
| PRUAV004879 |  | gcaggtgaccttttccaagcggaggagagggcttttcaagaaggctcaggagctctccgttctctgtgatgcagatattgctcttatcatcttctcttcc |  |
| PavSVP_‘Regina’ |  | ~~~~~~~~~~~~~~~~~~~~~~~~~~~~~~~~~~~~~~~~~~~~~~~~~~~~~~~~~~~~~~~~~~~~~~~~~~~~~a~gcTGTTATC~~~TTCTCTTC~ |  |
| PavSVP_‘Lapins’ |  | ~~~~~~~~~~~~~~~~~~~~~~~~~~~~~~~~~~~~~~~~~~~~~~~~~~~~~~~~~~~~~~~~~~~~~~~~~~~~~~~aagCTTT~C~TCTTCTCTTC~ |  |
| PavSVP_‘Garnet’ |  | ~~~~~~~~~~~~~~~~~~~~~~~~~~~~~~~~~~~~~~~~~~~~~~~~~~~~~~~~~~~~~~~~~~~~~~~~~~~~~aagggCTACTC~~~TTCTCTTC~ |  |
|  |  |  |  |
|  |  | ....|....|....|....|....|....|....|....|....|....|....|....|....|....|....|....|....|....|....|....| |  |
| ppa011063m |  | ACTGGAAAGCTCTTTGAATACGCCAGCTCCAGGTCTCTCTATCTCAC~~AAACTCAAGCCCTCTATTTTTATTTTATTAT~~~~ATATATATATATATAT |  |
| PRUAV004879 |  | actggaaagctctttgaatacgccagctccag~~~~~~~~~~~~~~~~~~~~~~~~~~~~~~~~~~~~~~~~~~~~~~~~~~~~~~~~~~~~~~~~~~~~ |  |
| PavSVP_‘Regina’ |  | ACTGGAA~GCTCTTTGAATACGCCAGCTCCAGGTCTCTCTCTCTCTCACAAACTCAAGCCCACTATTTTT~~~TTATTATTTTTA~ATGTATATAT~T~T |  |
| PavSVP_‘Lapins’ |  | ACTGGAA~GCTCTTTGAATACGCCAGCTCCAGGTCTCTCTCTCTCTCACAAACTCAAGCCCACTATTTTT~~~TTATTATTTTTA~ATGTATATAT~T~T |  |
| PavSVP_‘Garnet’ |  | ACTGGAA~GCTCTTTGAATACGCCAGCTCCAGGTCTCTCTCTCTCTCACAAACTCAAGCCCACTATTTTT~~~TTATTATTTTTA~ATGTATATAT~T~T |  |
|  |  |  |  |
|  |  | ....|....|....|....|....|....|....|....|....|....|....|....|....|....|....|....|....|....|....|....| |  |
| ppa011063m |  | TTTATCATCCGTAAAAATAGATAGGCTTAAAATTTGAGGATACGGTTTTGTTTATTTTTTGGGGGGTGAAAAATGAGGAGGAGGAAGA~~~~~~~~~~~~ |  |
| PRUAV004879 |  | ~~~~~~~~~~~~~~~~~~~~~~~~~~~~~~~~~~~~~~~~~~~~~~~~~~~~~~~~~~~~~~~~~~~~~~~~~~~~~~~~~~~~~~~~~~~~~~~~~~~~ |  |
| PavSVP_‘Regina’ |  | TTTATCATCCGTAAAAATTGATAGGCTTAAAATTTGAGGGTT~GGTTGTGTTTATTTTATTGGGGGTGAAAAATGAGGARGAGGAGGAGGAGGAGGAGGA |  |
| PavSVP_‘Lapins’ |  | TTTATCATCCGTAAAAATTGATAGGCTTAAAATTTGAGGGTT~GGTTGTGTTTATTTTATTGGGGGTGAAAAATGAGGAAGAGGAGGAGGAGGAGGAGGA |  |
| PavSVP_‘Garnet’ |  | TTTATCATCCGTAAAAATTGATAGGCTTAAAATTTGAGGGTT~GGTTGTGTTTATTTTATTGGGGGTGAAAAATGAGGARGAGGAGGAGGAGGAGGAGGA |  |
|  |  |  |  |
|  |  | ....|....|....|....|....|....|....|....|....|....|....|....|....|....|....|....|....|....|....|....| |  |
| ppa011063m |  | ~~~~~ACC~ACGTCAATGGAAGTAGCTGCTCCGGTAGTCAAGCTATATTATAATAATATTAAGAGGGTGTATATATACATATATATATATATGTATATAT |  |
| PRUAV004879 |  | ~~~~~~~~~~~~~~~~~~~~~~~~~~~~~~~~~~~~~~~~~~~~~~~~~~~~~~~~~~~~~~~~~~~~~~~~~~~~~~~~~~~~~~~~~~~~~~~~~~~~ |  |
| PavSVP_‘Regina’ |  | GGAAGACCCACGTCAATGGAAGTGGCTGCTCAGCTAGTCAAGCTATATTATAATAATATTAAGAGGG~~TATATATA~~TATATA~A~~~~~~~~~~~~~ |  |
| PavSVP_‘Lapins’ |  | GGAAGACCCACGTCAATGGAAGTGGCTGCTCAGCTAGTCAAGCTATATTATAATAATATTAAGAGGG~~TATATATA~~TATATA~A~~~~~~~~~~~~~ |  |
| PavSVP_‘Garnet’ |  | GGAAGACCCACGTCAATGGAAGTGGCTGCTCAGCTAGTCAAGCTATATTATAATAATATTAAGAGGG~~TATATATA~~TATATA~A~~~~~~~~~~~~~ |  |
|  |  |  |  |
|  |  | ....|....|....|....|....|....|....|....|....|....|....|....|....|....|....|....|....|....|....|....| |  |
| ppa011063m |  | ATGTATGTGTGTATATAAATGTATATATAATAGGGTGAAGGGTCTGCAACTTGGATGTGGATGTTGTTTTCTG~GGATGAATATAATCATATAATATACA |  |
| PRUAV004879 |  | ~~~~~~~~~~~~~~~~~~~~~~~~~~~~~~~~~~~~~~~~~~~~~~~~~~~~~~~~~~~~~~~~~~~~~~~~~~~~~~~~~~~~~~~~~~~~~~~~~~~~ |  |
| PavSVP_‘Regina’ |  | ~~~~~~~~~~~~~~~~~~~~~~~~~~~~AATAGGGTGAAGGGTCTGCAACTTGGAT~~~~~TGTTGTTTTCTGTGGATGAATATAGTCATATAATATACA |  |
| PavSVP_‘Lapins’ |  | ~~~~~~~~~~~~~~~~~~~~~~~~~~~~AATAGGGTGAAGGGTCTGCAACTTGGAT~~~~~TGTTGTTTTCTGTGGATGAATATAGTCATATAATATACA |  |
| PavSVP_‘Garnet’ |  | ~~~~~~~~~~~~~~~~~~~~~~~~~~~~AATAGGGTGAAGGGTCTGCAACTTGGAT~~~~~TGTTGTTTTCTGTGGATGAATATAGTCATATAATATACA |  |
|  |  |  |  |
|  |  | ....|....|....|....|....|....|....|....|....|....|....|....|....|....|....|....|....|....|....|....| |  |
| ppa011063m |  | TAACAACAAACAAGGGATAAGACACTGTTCTTATTGGGGTCTAATCAGTTGCTGTTCGTATTGGCTACCGCCCATTTTTGACCATGGTTGGGTTTGTGAC |  |
| PRUAV004879 |  | ~~~~~~~~~~~~~~~~~~~~~~~~~~~~~~~~~~~~~~~~~~~~~~~~~~~~~~~~~~~~~~~~~~~~~~~~~~~~~~~~~~~~~~~~~~~~~~~~~~~~ |  |
| PavSVP_‘Regina’ |  | TAAAAACAAACAAGGGATAAGACACTGTTATTATTGGGGTCTAATCACTTGCTATTCTTATTGGATACCGCCCATTTT~GACCATGGTTGGGTTTGTGAT |  |
| PavSVP_‘Lapins’ |  | TAAAAACAAACAAGGGATAAGACACTGTTATTATTGGGGTCTAATCACTTGCTATTCTTATTGGATACCGCCCATTTT~GACCATGGTTGGGTTTGTGAT |  |
| PavSVP_‘Garnet’ |  | TAAAAACAAACAAGGGATAAGACACTGTTATTATTGGGGTCTAATCACTTGCTATTCTTATTGGATACCGCCCATTTT~GACCATGGTTGGGTTTGTGAT |  |
|  |  |  |  |
|  |  | ....|....|....|....|....|....|....|....|....|....|....|....|....|....|....|....|....|....|....|....| |  |
| ppa011063m |  | GCAAAAATGAACACCGAATACAAGTTTGACCAACCATGACTAAACAATTTACTACTTAGAAAGAAATCAAAACAGCCAAAATTAAGAAATTGCACCTTAA |  |
| PRUAV004879 |  | ~~~~~~~~~~~~~~~~~~~~~~~~~~~~~~~~~~~~~~~~~~~~~~~~~~~~~~~~~~~~~~~~~~~~~~~~~~~~~~~~~~~~~~~~~~~~~~~~~~~~ |  |
| PavSVP_‘Regina’ |  | GCAAAAATGAGCAACGTATACGAGTTTGACCAACCAAGACTAAACAATTTACCACC~ATAAAGAAATCAAAT~AGCCAAAATTAAGAAATTCCACCTAAA |  |
| PavSVP_‘Lapins’ |  | GCAAAAATGAGCAACGTATACGAGTTTGACCAACCAAGACTAAACAATTTACCACC~ATAAAGAAATCAAAT~AGCCAAAATTAAGAAATTCCACCTAAA |  |
| PavSVP_‘Garnet’ |  | GCAAAAATGAGCAACGTATACGAGTTTGACCAACCAAGACTAAACAATTTACCACC~ATAAAGAAATCAAAT~AGCCAAAATTAAGAAATTCCACCTAAA |  |
|  |  |  |  |
|  |  | ....|....|....|....|....|....|....|....|....|....|....|....|....|....|....|....|....|....|....|....| |  |
| ppa011063m |  | GGAGTTGATAAATTACAGCCATCTATTTTTCTTTTTCAAGTTTCCCTCTATTGGGTTTTGTAACAATGCTTCAAATTTGTTTGAGAAAATTTTGTTTCTG |  |
| PRUAV004879 |  | ~~~~~~~~~~~~~~~~~~~~~~~~~~~~~~~~~~~~~~~~~~~~~~~~~~~~~~~~~~~~~~~~~~~~~~~~~~~~~~~~~~~~~~~~~~~~~~~~~~~~ |  |
| PavSVP_‘Regina’ |  | GGAGTTGAACAATTACGGCCATCTATTTTTCTTTTTCAAGTTTCCCTCTATTGGGTTTTGTAACAATGCTTCAAATTTGTTTGAGAAAATTTTGTTTCTG |  |
| PavSVP_‘Lapins’ |  | GGAGTTGAACAATTACGGCCATCTATTTTTCTTTTTCAAGTTTCCCTCTATTGGGTTTTGTAACAATGCTTCAAATTTGTTTGAGAAAATTTTGTTTCTG |  |
| PavSVP_‘Garnet’ |  | GGAGTTGAACAATTACGGCCATCTATTTTTCTTTTTCAAGTTTCCCTCTATTGGGTTTTGTAACAATGCTTCAAATTTGTTTGAGAAAATTTTGTTTCTG |  |
|  |  |  |  |
|  |  | ....|....|....|....|....|....|....|....|....|....|....|....|....|....|....|....|....|....|....|....| |  |
| ppa011063m |  | TTAAAAACACGGTCATAGTGTTGTTAATTCACGTGTTATAACATAAATAAACGACAGGTAAATATTTTAAAATAAATTTGTCATATCATGCTATAGCGTC |  |
| PRUAV004879 |  | ~~~~~~~~~~~~~~~~~~~~~~~~~~~~~~~~~~~~~~~~~~~~~~~~~~~~~~~~~~~~~~~~~~~~~~~~~~~~~~~~~~~~~~~~~~~~~~~~~~~~ |  |
| PavSVP_‘Regina’ |  | TTAAAAACATGGCCATAATGTTGTTAATTCACGTGTTATAATATAAGTGAATGACGAGTAAATACTTTAAGATAAATTTGTCATATCATGCGACAGTGTC |  |
| PavSVP_‘Lapins’ |  | TTAAAAACATGGCCATAATGTTGTTAATTCACGTGTTATAATATAAGTGAATGACGAGTAAATACTTTAAGATAAATTTGTCATATCATGCGACAGTGTC |  |
| PavSVP_‘Garnet’ |  | TTAAAAACATGGCCATAATGTTGTTAATTCACGTGTTATAATATAAGTGAATGACGAGTAAATACTTTAAGATAAATTTGTCATATCATGCGACAGTGTC |  |
|  |  |  |  |
|  |  | ....|....|....|....|....|....|....|....|....|....|....|....|....|....|....|....|....|....|....|....| |  |
| ppa011063m |  | ACGATGACAACAAAAATTGCTCATATACTATATATGTAAGATTCTAGAGGGGAAAAACATTTTAAGTTTAAAA~GTGTAACAAGTTGCAGCAGCATCTTC |  |
| PRUAV004879 |  | ~~~~~~~~~~~~~~~~~~~~~~~~~~~~~~~~~~~~~~~~~~~~~~~~~~~~~~~~~~~~~~~~~~~~~~~~~~~~~~~~~~~~~~~~~~~~~~~~~~~~ |  |
| PavSVP_‘Regina’ |  | ACTATAACAACAAAAATTGCTCATATACTATATATGTAAGATTCTAGAGGGGAAAAACATTTTAAGTTTAAAAAGTGTAACAAGTTGCAGCAGCATCTTC |  |
| PavSVP_‘Lapins’ |  | ACTATAACAACAAAAATTGCTCATATACTATATATGTAAGATTCTAGAGGGGAAAAACATTTTAAGTTTAAAA~GTGTAACAAGTTGCAGCAGCATCTTC |  |
| PavSVP_‘Garnet’ |  | ACTATAACAACAAAAATTGCTCATATACTATATATGTAAGATTCTAGAGGGGAAAAACATTTTAAGTTTAAAA~GTGTAACAAGTTGCAGCAGCATCTTC |  |
|  |  |  |  |
|  |  | ....|....|....|....|....|....|....|....|....|....|....|....|....|....|....|....|....|....|....|....| |  |
| ppa011063m |  | GAAATGCTAAACCAAAAAGTTGCACTAAAATGTGAATAACATCGCTTTATGTCTTTTCCATTTTT~~~~~~~~~~~~~~ACTGCTATTTGCTTTGAAGCT |  |
| PRUAV004879 |  | ~~~~~~~~~~~~~~~~~~~~~~~~~~~~~~~~~~~~~~~~~~~~~~~~~~~~~~~~~~~~~~~~~~~~~~~~~~~~~~~~~~~~~~~~~~~~~~~~~~~~ |  |
| PavSVP_‘Regina’ |  | GAAATGCTAAACCAAAAAACTGCACTAAAATGTGAATAACATCTTTTTATGCCCTTTCCATTTTTGTTATTTTTTATTTACTGCTATTTGCTTTGAAGCT |  |
| PavSVP_‘Lapins’ |  | GAAATGCTAAACCAAAAAACTGCACTAAAATGTGAATAACATCTTTTTATGCCCTTTCCATTTTTGTTATTTTTTATTTACTGCTATTTGCTTTGAAGCT |  |
| PavSVP_‘Garnet’ |  | GAAATGCTAAACCAAAAAACTGCACTAAAATGTGAATAACATCTTTTTATGCCCTTTCCATTTTTGTTATTTTTTATTTACTGCTATTTGCTTTGAAGCT |  |
|  |  |  |  |
|  |  | ....|....|....|....|....|....|....|....|....|....|....|....|....|....|....|....|....|....|....|....| |  |
| ppa011063m |  | TTCTAGTTAACCAGCTAGTGAAGTGTATTATTTTTCTTTTCTGAAGCATGAA~GGAAATTCTAGAACGTCACAACTTGCACGCAAAGAATCTCTCGAAAA |  |
| PRUAV004879 |  | ~~~~~~~~~~~~~~~~~~~~~~~~~~~~~~~~~~~~~~~~~~~~~~catgaa~ggaaattctagaacgtcacaacttgcacgccaagaatctctcgaaaa |  |
| PavSVP_‘Regina’ |  | TTCTAGTTAACCAGCTAGTGACGTGTCTTGTTTTTCTCTTCTGAAGCATGAAAGGAAATTCTAGA~CGacacacaccctttttgcgagaa |  |
| PavSVP_‘Lapins’ |  | TTCTAGTTAACCAGCTAGTGACGTGTCTTGTTTTTCTCTTCTGAAGCATGAA~GGAAATTCTAGA~CGacaacacttttgttgggagaga |  |
| PavSVP_‘Garnet’ |  | TTCTAGTTAACCAGCTAGTGACGTGTCTTGTTTTTCTCTTCTGAAGCATGAAAGAAATTTctaga~cgcccacaaattcttggcagga |  |

***SWN***

|  |  | ....|....|....|....|....|....|....|....|....|....|....|....|....|....|....|....|....|....|....|....| |  |
| --- | --- | --- | --- |
| ppa001254m | 1 | ~~~~~~~~~~~~~~~~~~~~~~~~~~~~~~~~~~~ATGGTGTCCAAAGCTACAGACTCTGCAACTAAACTCAGAGTCAGTATCTCAAAACTTTACCTCTC |  |
| PRUAV013572 |  | ggagcgcacaggcacggcagatgagcaaaacagggatggtgtccaaagctacagactctgcaactaaactcaga~~~~~~~~~~~~~~~~~~~~~~~~~~ |  |
| PavSWN_‘Regina’ |  | ~~~~~~~~~~~~~~~~~~~~~~~~~~~~~~~~~~~~~~~~~~~~~~~~~~~~~~~~~~~~~~~~~~~~~~~~~~~~~~~~~~~~~~~~~~~~~~~~~~~~ |  |
| PavSWN_‘Lapins’ |  | ~~~~~~~~~~~~~~~~~~~~~~~~~~~~~~~~~~~~~~~~~~~~~~~~~~~~~~~~~~~~~~~~~~~~~~~~~~~~~~~~~~~~~~~~~~~~~~~~~~~~ |  |
| PavSWN_‘Garnet’ |  | ~~~~~~~~~~~~~~~~~~~~~~~~~~~~~~~~~~~~~~~~~~~~~~~~~~~~~~~~~~~~~~~~~~~~~~~~~~~~~~~~~~~~~~~~~~~~~~~~~~~~ |  |
|  |  |  |  |
|  |  | ....|....|....|....|....|....|....|....|....|....|....|....|....|....|....|....|....|....|....|....| |  |
| ppa001254m |  | GATCCTTGAATTCGTTATTTCTTCTCGCGATTTTGGCTCGTGTTTGCTTTTTAAATAAAAATTTCGATGCTTTTCATCATAATTGGAATTGAGATGATCA |  |
| PRUAV013572 |  | ~~~~~~~~~~~~~~~~~~~~~~~~~~~~~~~~~~~~~~~~~~~~~~~~~~~~~~~~~~~~~~~~~~~~~~~~~~~~~~~~~~~~~~~~~~~~~~~~~~~~ |  |
| PavSWN_‘Regina’ |  | ~~~~~~~~~~~~~~~~~~~~~~~~~~~~~~~~~~~~~~~~~~~~~~~~~~~~~~~~~~~~~~~~~~~~~~~~~~~~~~~~~~~~~~~~~~~~~~~~~~~~ |  |
| PavSWN_‘Lapins’ |  | ~~~~~~~~~~~~~~~~~~~~~~~~~~~~~~~~~~~~~~~~~~~~~~~~~~~~~~~~~~~~~~~~~~~~~~~~~~~~~~~~~~~~~~~~~~~~~~~~~~~~ |  |
| PavSWN_‘Garnet’ |  | ~~~~~~~~~~~~~~~~~~~~~~~~~~~~~~~~~~~~~~~~~~~~~~~~~~~~~~~~~~~~~~~~~~~~~~~~~~~~~~~~~~~~~~~~~~~~~~~~~~~~ |  |
|  |  |  |  |
|  |  | ....|....|....|....|....|....|....|....|....|....|....|....|....|....|....|....|....|....|....|....| |  |
| ppa001254m |  | AACAGGGATGAGAATTTATGTTTGGATTTTCAAATTGCAGTTTGGATATGAAAGAAATGAAAATTTTGAATTTAATGAGTTAGAAGTTAGAACTCATAGC |  |
| PRUAV013572 |  | ~~~~~~~~~~~~~~~~~~~~~~~~~~~~~~~~~~~~~~~~~~~~~~~~~~~~~~~~~~~~~~~~~~~~~~~~~~~~~~~~~~~~~~~~~~~~~~~~~~~~ |  |
| PavSWN_‘Regina’ |  | ~~~~~~~~~~~~~~~~~~~~~~~~~~~~~~~~~~~~~~~~~~~~~~~~~~~~~~~~~~~~~~~~~~~~~~~~~~~~~~~~~~~~~~~~~~~~~~~~~~~~ |  |
| PavSWN_‘Lapins’ |  | ~~~~~~~~~~~~~~~~~~~~~~~~~~~~~~~~~~~~~~~~~~~~~~~~~~~~~~~~~~~~~~~~~~~~~~~~~~~~~~~~~~~~~~~~~~~~~~~~~~~~ |  |
| PavSWN_‘Garnet’ |  | ~~~~~~~~~~~~~~~~~~~~~~~~~~~~~~~~~~~~~~~~~~~~~~~~~~~~~~~~~~~~~~~~~~~~~~~~~~~~~~~~~~~~~~~~~~~~~~~~~~~~ |  |
|  |  |  |  |
|  |  | ....|....|....|....|....|....|....|....|....|....|....|....|....|....|....|....|....|....|....|....| |  |
| ppa001254m |  | TTCTTGTAGTCATTAAGAAATATTTATAGAGAAATAGCTGTGGTTCTACTATTAATCTGCAGCCAAACAGGTGAACAAAATCTTATTATGTTTGATATGA |  |
| PRUAV013572 |  | ~~~~~~~~~~~~~~~~~~~~~~~~~~~~~~~~~~~~~~~~~~~~~~~~~~~~~~~~~~~~~~~~~~~~~~~~~~~~~~~~~~~~~~~~~~~~~~~~~~~~ |  |
| PavSWN_‘Regina’ |  | ~~~~~~~~~~~~~~~~~~~~~~~~~~~~~~~~~~~~~~~~~~~~~~~~~~~~~~~~~~~~~~~~~~~~~~~~~~~~~~~~~~~~~~~~~~~~~~~~~~~~ |  |
| PavSWN_‘Lapins’ |  | ~~~~~~~~~~~~~~~~~~~~~~~~~~~~~~~~~~~~~~~~~~~~~~~~~~~~~~~~~~~~~~~~~~~~~~~~~~~~~~~~~~~~~~~~~~~~~~~~~~~~ |  |
| PavSWN_‘Garnet’ |  | ~~~~~~~~~~~~~~~~~~~~~~~~~~~~~~~~~~~~~~~~~~~~~~~~~~~~~~~~~~~~~~~~~~~~~~~~~~~~~~~~~~~~~~~~~~~~~~~~~~~~ |  |
|  |  |  |  |
|  |  | ....|....|....|....|....|....|....|....|....|....|....|....|....|....|....|....|....|....|....|....| |  |
| ppa001254m |  | AATTCACGAATTTATATGCACTCTCTGATTTACGTGGGCAGAAATCACATGGAGAAGAGCCAAGTGATGGTGTTGGAAACTTGGAACATAAGATGCATCA |  |
| PRUAV013572 |  | ~~~~~~~~~~~~~~~~~~~~~~~~~~~~~~~~~~~~~~~~~aaaccacatggagaagagccaagtgatggtgttggaaacttggaacataagatgcatca |  |
| PavSWN_‘Regina’ |  | ~~~~~~~~~~~~~~~~~~~~~~~~~~~~~~~~~~~~~~~~~~~~~~~~~~~~~~~~~~~~~~~~~~~~~~~~~~~~~~~~~~~~~~~~~~~~~~~~~~~~ |  |
| PavSWN_‘Lapins’ |  | ~~~~~~~~~~~~~~~~~~~~~~~~~~~~~~~~~~~~~~~~~~~~~~~~~~~~~~~~~~~~~~~~~~~~~~~~~~~~~~~~~~~~~~~~~~~~~~~~~~~~ |  |
| PavSWN_‘Garnet’ |  | ~~~~~~~~~~~~~~~~~~~~~~~~~~~~~~~~~~~~~~~~~~~~~~~~~~~~~~~~~~~~~~~~~~~~~~~~~~~~~~~~~~~~~~~~~~~~~~~~~~~~ |  |
|  |  |  |  |
|  |  | ....|....|....|....|....|....|....|....|....|....|....|....|....|....|....|....|....|....|....|....| |  |
| ppa001254m |  | GCTTAAGAAGCAAATTCAAGCAGA~GAGAATGGTTTCAGTAAAAGTGAGTTTTCGCATATATATAATATGCACTATATAGGCGATAAACGATAAATGATG |  |
| PRUAV013572 |  | gcttaagaagcaaattcaagcagangagaatggtttcagtaaaa~~~~~~~~~~~~~~~~~~~~~~~~~~~~~~~~~~~~~~~~~~~~~~~~~~~~~~~~ |  |
| PavSWN_‘Regina’ |  | ~~~~~~~~~~~~~~~~~~~~~~~~~~~~~~~~~~~~~~~~~~~~~~~~~~~~~~~~~~~~~~~~~~~~~~~~~~~~~~~~~~~~~~~~~~~~~~~~~~~~ |  |
| PavSWN_‘Lapins’ |  | ~~~~~~~~~~~~~~~~~~~~~~~~~~~~~~~~~~~~~~~~~~~~~~~~~~~~~~~~~~~~~~~~~~~~~~~~~~~~~~~~~~~~~~~~~~~~~~~~~~~~ |  |
| PavSWN_‘Garnet’ |  | ~~~~~~~~~~~~~~~~~~~~~~~~~~~~~~~~~~~~~~~~~~~~~~~~~~~~~~~~~~~~~~~~~~~~~~~~~~~~~~~~~~~~~~~~~~~~~~~~~~~~ |  |
|  |  |  |  |
|  |  | ....|....|....|....|....|....|....|....|....|....|....|....|....|....|....|....|....|....|....|....| |  |
| ppa001254m |  | AACGATGGTAGCCAATGCGCAATTGACTCCTTCCTCGTGCTTATACTTTGCCCAGGAAAAAGTTGAGAAGAACAGGGAGAAGCTAGGAGGTTACATTTCG |  |
| PRUAV013572 |  | ~~~~~~~~~~~~~~~~~~~~~~~~~~~~~~~~~~~~~~~~~~~~~~~~~~~~~~~gaaaa~gttgagaagaacagggagaagctaggaggttacatatcg |  |
| PavSWN_‘Regina’ |  | ~~~~~~~~~~~~~~~~~~~~~~~~~~~~~~~~~~~~~~~~~~~~~~~~~~~~~~~~~~~~~~~~~~~~~~~~~~~~~~~~~~~~~~~~~~~~~~~~~~~~ |  |
| PavSWN_‘Lapins’ |  | ~~~~~~~~~~~~~~~~~~~~~~~~~~~~~~~~~~~~~~~~~~~~~~~~~~~~~~~~~~~~~~~~~~~~~~~~~~~~~~~~~~~~~~~~~~~~~~~~~~~~ |  |
| PavSWN_‘Garnet’ |  | ~~~~~~~~~~~~~~~~~~~~~~~~~~~~~~~~~~~~~~~~~~~~~~~~~~~~~~~~~~~~~~~~~~~~~~~~~~~~~~~~~~~~~~~~~~~~~~~~~~~~ |  |
|  |  |  |  |
|  |  | ....|....|....|....|....|....|....|....|....|....|....|....|....|....|....|....|....|....|....|....| |  |
| ppa001254m |  | CAAATTATATCAGCAACCTCAAGAGCGAATTCTACACTTCCAGAGAAAAATGGGAGCTTTAAATTGTTTCCTTCAAGAATTGAACAGCCTCTCTGCAAGT |  |
| PRUAV013572 |  | caaattatatcagcaacatcaagagcgaattctacagtctcagagaaaaatgggagctttaaattgtttccttcaagaattgaacagcctctctgcaagt |  |
| PavSWN_‘Regina’ |  | ~~~~~~~~~~~~~~~~~~~~~~~~~~~~~~~~~~~~~~~~~~~~~~~~~~~~~~~~~~~~~~~~~~~~~~~~~~~~~~~~~~~~~~~~~~~~~~~~~~~~ |  |
| PavSWN_‘Lapins’ |  | ~~~~~~~~~~~~~~~~~~~~~~~~~~~~~~~~~~~~~~~~~~~~~~~~~~~~~~~~~~~~~~~~~~~~~~~~~~~~~~~~~~~~~~~~~~~~~~~~~~~~ |  |
| PavSWN_‘Garnet’ |  | ~~~~~~~~~~~~~~~~~~~~~~~~~~~~~~~~~~~~~~~~~~~~~~~~~~~~~~~~~~~~~~~~~~~~~~~~~~~~~~~~~~~~~~~~~~~~~~~~~~~~ |  |
|  |  |  |  |
|  |  | ....|....|....|....|....|....|....|....|....|....|....|....|....|....|....|....|....|....|....|....| |  |
| ppa001254m |  | TCAGTGGATTTGGTCACGGATATGGTGATAAAGACTATATTAACAATCAAGAGGTGGTATTCTCATCAAGTACCAAGCTTCCATCTGCTGAAAATTTACC |  |
| PRUAV013572 |  | tcagtggatttgctcacggatatggtgataaagactatattaacaatcaagaggtggtattctcatcaagtaccaagcttccatctgctgaaaatttcca |  |
| PavSWN_‘Regina’ |  | ~~~~~~~~~~~~~~~~~~~~~~~~~~~~~~~~~~~~~~~~~~~~~~~~~~~~~~~~~~~~~~~~~~~~~~~~~~~~~~~~~~~~~~~~~~~~~~~~~~~~ |  |
| PavSWN_‘Lapins’ |  | ~~~~~~~~~~~~~~~~~~~~~~~~~~~~~~~~~~~~~~~~~~~~~~~~~~~~~~~~~~~~~~~~~~~~~~~~~~~~~~~~~~~~~~~~~~~~~~~~~~~~ |  |
| PavSWN_‘Garnet’ |  | ~~~~~~~~~~~~~~~~~~~~~~~~~~~~~~~~~~~~~~~~~~~~~~~~~~~~~~~~~~~~~~~~~~~~~~~~~~~~~~~~~~~~~~~~~~~~~~~~~~~~ |  |
|  |  |  |  |
|  |  | ....|....|....|....|....|....|....|....|....|....|....|....|....|....|....|....|....|....|....|....| |  |
| ppa001254m |  | ACCTTATACAACTTGGATATTCTTGGACAGGTTTGATATTCGTTTCTTTGAATCCTTTATGAGATTATGAAAAGAACGGTAGTTTTTTTTTAATTTTATA |  |
| PRUAV013572 |  | ccttatacaacttgngatattcttggacag~~~~~~~~~~~~~~~~~~~~~~~~~~~~~~~~~~~~~~~~~~~~~~~~~~~~~~~~~~~~~~~~~~~~~~ |  |
| PavSWN_‘Regina’ |  | ~~~~~~~~~~~~~~~~~~~~~~~~~~~~~~~~~~~~~~~~~~~~~~~~~~~~~~~~~~~~~~~~~~~~~~~~~~~~~~~~~~~~~~~~~~~~~~~~~~~~ |  |
| PavSWN_‘Lapins’ |  | ~~~~~~~~~~~~~~~~~~~~~~~~~~~~~~~~~~~~~~~~~~~~~~~~~~~~~~~~~~~~~~~~~~~~~~~~~~~~~~~~~~~~~~~~~~~~~~~~~~~~ |  |
| PavSWN_‘Garnet’ |  | ~~~~~~~~~~~~~~~~~~~~~~~~~~~~~~~~~~~~~~~~~~~~~~~~~~~~~~~~~~~~~~~~~~~~~~~~~~~~~~~~~~~~~~~~~~~~~~~~~~~~ |  |
|  |  |  |  |
|  |  | ....|....|....|....|....|....|....|....|....|....|....|....|....|....|....|....|....|....|....|....| |  |
| ppa001254m |  | TATCTTTAATCTGTGCAGTCCTTTTGTTTTTAATGCGAAAATGTTGTTGTTGTGTTTCTCCTATCTCAAATTTGTCTTTCACAGCACAGAAATCAGAGAA |  |
| PRUAV013572 |  | ~~~~~~~~~~~~~~~~~~~~~~~~~~~~~~~~~~~~~~~~~~~~~~~~~~~~~~~~~~~~~~~~~~~~~~~~~~~~~~~~~~~~~~~~~aaatcagagaa |  |
| PavSWN_‘Regina’ |  | ~~~~~~~~~~~~~~~~~~~~~~~~~~~~~~~~~~~~~~~~~~~~~~~~~~~~~~~~~~~~~~~~~~~~~~~~~~~~~~~~~~~~~~~~~~~~~~~~~~~~ |  |
| PavSWN_‘Lapins’ |  | ~~~~~~~~~~~~~~~~~~~~~~~~~~~~~~~~~~~~~~~~~~~~~~~~~~~~~~~~~~~~~~~~~~~~~~~~~~~~~~~~~~~~~~~~~~~~~~~~~~~~ |  |
| PavSWN_‘Garnet’ |  | ~~~~~~~~~~~~~~~~~~~~~~~~~~~~~~~~~~~~~~~~~~~~~~~~~~~~~~~~~~~~~~~~~~~~~~~~~~~~~~~~~~~~~~~~~~~~~~~~~~~~ |  |
|  |  |  |  |
|  |  | ....|....|....|....|....|....|....|....|....|....|....|....|....|....|....|....|....|....|....|....| |  |
| ppa001254m |  | TGGCTGACGACCAATCAGTAGTTGGGAGGAGGCGCATTTACTATGATAAAGATGGCACTGATGGCAGTGAAGCTCTTGTCTGCAGTGACACTGACGAAGA |  |
| PRUAV013572 |  | tggccgacgaccaatcagtagttgggaggagacgcatttactacgataaagatggcactgatggcagtgaagctcttgtctgcagtgacactgacgaaga |  |
| PavSWN_‘Regina’ |  | ~~~~~~~~~~~~~~~~~~~~~~~~~~~~~~~~~~~~~~~~~~~~~~~~acaatGGC~CTGATGGCAGTGA~GCTCTTGTCTGCAGTGACACTGACGAAGA |  |
| PavSWN_‘Lapins’ |  | ~~~~~~~~~~~~~~~~~~~~~~~~~~~~~~~~~~~~~~~~~~~~~~~~gcaATG~~ACTGATGGCAGTGA~GCTCTTGTCTGCAGTGACACTGACGAAGA |  |
| PavSWN_‘Garnet’ |  | ~~~~~~~~~~~~~~~~~~~~~~~~~~~~~~~~~~~~~~~~~~~~~~~acaaatGG~ACTGATGGCAGTGA~GCTCTTGTCTGCAGTGACACTGACGAAGA |  |
|  |  |  |  |
|  |  | ....|....|....|....|....|....|....|....|....|....|....|....|....|....|....|....|....|....|....|....| |  |
| ppa001254m |  | GATAGCAGAACCTGAAGAAGTGAAACATGAATTTACTGCAGGCGAAGATCGAATTATGTCGTAAGTTTTAAATGAGATATAGACT~~~~~~~~ATCACAA |  |
| PRUAV013572 |  | gatagcagaacccgaagaagt |  |
| PavSWN_‘Regina’ |  | GATAGCAGAACCCGAAGAAGTGAAACATGAATTTACTGCAGGCGAAGATCGAATTATGTCGTAAGTTTTAAATGAGATATAGACTTTTAGAGAATCACAA |  |
| PavSWN_‘Lapins’ |  | GATAGCAGAACCCGAAGAAGTGAAACATGAATTTACTGCAGGCGAAGATCGAATTATGTCGTAAGTTTTAAATGAGATATAGACTTTTAGAGAATCACAA |  |
| PavSWN_‘Garnet’ |  | GATAGCAGAACCCGAAGAAGTGAAACATGAATTTACTGCAGGCGAAGATCGAATTATGTCGTAAGTTTTAAATGAGATATAGACTTTTAGAGAATCACAA |  |
|  |  |  |  |
|  |  | ....|....|....|....|....|....|....|....|....|....|....|....|....|....|....|....|....|....|....|....| |  |
| ppa001254m |  | TGACTGTGCTGCTTATTAACTTGTCATAGTCCTGCTTTTCTTAGGACCAAGTGAAGGGTCTTACTTTTTCTGTGTGAATTTGTTAGGATGGCCTTTCAGG |  |
| PRUAV013572 |  |  |  |
[truncated: 43,153 more chars]
